# Is Antipsychotic Drug Use During Pregnancy Associated with Increased Malformation Rates and Worsening of Maternal and Infant Outcomes? A Systematic Review

**DOI:** 10.2174/1570159X22666240516151449

**Published:** 2024-05-17

**Authors:** Gabriele Sani, Tommaso Callovini, Ottavia Marianna Ferrara, Daniele Segatori, Stella Margoni, Alessio Simonetti, Francesco Maria Lisci, Giuseppe Marano, Alessia Fischetti, Georgios D. Kotzalidis, Federica Di Segni, Federica Fiaschè, Delfina Janiri, Lorenzo Moccia, Giovanni Manfredi, Alessandro Alcibiade, Caterina Brisi, Flavia Grisoni, Gianmarco Stella, Evelina Bernardi, Andrea Brugnami, Michele Ciliberto, Maria Chiara Spera, Romina Caso, Sara Rossi, Gianluca Boggio, Giulia Mastroeni, Francesca Abate, Eliana Conte, Anna Quintano, Lavinia De Chiara, Laura Monti, Giovanni Camardese, Lucio Rinaldi, Alexia E. Koukopoulos, Daniela Pia Rosaria Chieffo, Gloria Angeletti, Marianna Mazza

**Affiliations:** 1 Department of Psychiatry, Fondazione Policlinico Universitario Agostino Gemelli IRCCS, L.go Agostino Gemelli 8, 00168 Rome, Italy;; 2 Department of Neuroscience, Section of Psychiatry, Università Cattolica del Sacro Cuore, 00168 Rome, Italy;; 3 Department of Medicine and Surgery, University of Milano-Bicocca, Monza, Italy;; 4 Department of Neurosciences, Mental Health, and Sensory Organs (NESMOS), Sapienza University of Rome, Sant'Andrea Hospital, Via di Grottarossa 1035-1039, 00189 Rome, Italy;; 5 Psychiatry Residency Training Programme, Faculty of Medicine and Psychology, Sapienza University of Rome, Via di Grottarossa 1035-1039, 00189 Rome, Italy;; 6 ASL RM1, Presidio Ospedaliero San Filippo Neri, Servizio Psichiatrico di Diagnosi e Cura, Via Martinotti, 20, 00136 Rome, Italy;; 7 UOC Psichiatria, Day Hospital, Sant'Andrea Teaching Hospital, Sapienza University of Rome, Via di Grottarossa 1035-1039, 00189 Rome, Italy;; 8 Marina Militare Italiana (Italian Navy), Defense Ministry of Italy, Piazza della Marina, 4, 00196 Rome, Italy;; 9 Early Intervention Unit, ASL Roma 3, 00152 Rome, Italy;; 10 Struttura Residenziale Psichiatrica Samadi S.p.A., via di Grottarossa Km. 2.200, 00189 Rome, Italy and Centro Lucio Bini, Via Crescenzio 42, 00193 Rome, Italy;; 11 UOS Clinical Psychology, Clinical Government, Fondazione Policlinico Universitario Agostino Gemelli IRCCS, 00168 Rome, Italy;; 12 Azienda Ospedaliera Universitaria Policlinico Umberto I, Viale dell’Università 30, 00185 Rome, Italy and Centro Lucio Bini, Via Crescenzio 42, 00193 Rome, Italy;; 13 Women, Children and Public Health Department, Catholic University of the Sacred Heart, 00168 Rome, Italy

**Keywords:** Pregnancy, malformations, major, teratogenicity, lactation, antipsychotic drugs, second generation antipsychotics

## Abstract

There is much debate about continuing antipsychotic medication in patients who need it when they become pregnant because benefits must be weighed against potential teratogenic and malformation effects related to antipsychotics themselves. To address this, we conducted a systematic review on the PubMed, PsycINFO and CINHAL databases and the ClinicalTrials.gov register using the following strategy: (toxicity OR teratogenicity OR malformation* OR “birth defect*” OR “congenital abnormality” OR “congenital abnormalities” OR “brain changes” OR “behavioral abnormalities” OR “behavioral abnormalities”) AND antipsychotic* AND (pregnancy OR pregnant OR lactation OR delivery OR prenatal OR perinatal OR post-natal OR puerperium) on September 27, 2023. We found 38 studies to be eligible. The oldest was published in 1976, while most articles were recent. Most studies concluded that the antipsychotics, especially the second-generation antipsychotics, were devoid of teratogenic potential, while few studies were inconclusive and recommended replication. Most authoritative articles were from the Boston area, where large databases were implemented to study the malformation potential of psychiatric drugs. Other reliable databases are from Northern European registers. Overall conclusions are that antipsychotics are no more related to malformations than the disorders themselves; most studies recommend that there are no reasons to discontinue antipsychotic medications in pregnancy.

## 1 INTRODUCTION

Perinatal mental health has generated growing interest in recent years due to the increase in diagnoses of perinatal mental disorders and their impact on community health, particularly in young pregnant women [1]. Severe perinatal mental disorders can contribute to maternal mortality but also to adverse neonatal, infant and child outcomes [2].

Women with severe mental disorders, like bipolar disorder, schizoaffective disorder, other psychotic disorders or a severe major depressive episode, either bipolar or unipolar, are frequently exposed during their reproductive age to antipsychotic drugs (APs). In most instances, when they become pregnant, they continue to take APs during pregnancy and the postpartum period, thus affecting the developing foetus and the lactating newborn, respectively. It is known that APs can readily cross the placenta or be secreted in milk. Hence, their use carries potential risks during pregnancy and breastfeeding, such as congenital malformations, pregnancy and maternal outcomes, neonatal/infant risks, and developmental/long-term outcomes [3]. On the other hand, the perinatal period is associated with a high risk of new mental illness onset [4] or recurrence of recovered illness due to the hormonal turmoil and imbalance caused by pregnancy [5]. Severe perinatal mental illnesses are associated with considerable maternal and fetal/infant morbidity and mortality [6]. In assessing the benefit-to-risk ratio of peripartum medication use, the continuation of pharmacotherapy through the entire pregnancy and the postpartum appears to be protective in terms of symptom prevention or stability [7]. However, the risk of ongoing drug treatment for the mother or the foetus or newborn child cannot be neglected. The risk for every drug to assume during this very sensitive period of a woman’s life needs careful assessment.

In the majority of cases, the development of postpartum psychosis (a serious mental illness and a medical emergency consisting of symptoms like auditory hallucinations, delusions, and disorganization presenting within the first 4 weeks after delivery) occurs within 14 days from delivery [8]. Women with bipolar disorder [9] and schizophrenia [10] are particularly vulnerable to postpartum relapse and decompensation. In some cases, new-onset postpartum psychosis or mania may represent the first episode of a newly diagnosed bipolar disorder [11].

Since breastmilk is recommended as the exclusive source of nutrition for infants younger than six months due to the numerous health benefits for both infants and mothers [12], and considering that the risks of untreated psychosis are higher than the risks related to the use of antipsychotics, women taking APs often agree with their physicians to continue prescribed drug intake during breastfeeding. The treatment of severe mental illness in pregnant women represents a complex decision-making process that must include a careful evaluation of the potential risks and benefits of antipsychotic drugs.

Previous studies have outlined that there is no sufficient evidence to state that APs are major teratogens, but their use may be associated with greater metabolic risks for the mother and with impaired/altered growth in infants, who may be smaller or larger for gestational age or macrosomic when exposed to APs, but studies have not been consistent at this respect [13].

There is a need to clarify the issue of AP safety during the peripartum so as to inform clinical practice. The aim of this paper is to systematically review peer-reviewed published data regarding the safety of APs in pregnancy, puerperium, and lactation with a focus on the most commonly used antipsychotics.

## 2 MATERIALS AND METHODS

We searched three databases, including PubMed, PsycINFO/PsycLit, and CINAHL, using the following strategy: (toxicity OR teratogenicity OR malformation* OR “birth defect*” OR “congenital abnormality” OR “congenital abnormalities” OR “brain changes” OR “behavioral abnormalities” OR “behavioural abnormalities”) AND antipsychotic* AND (pregnancy OR pregnant OR lactation OR delivery OR prenatal OR perinatal OR post-natal OR puerperium). We also searched the ClinicalTrials.gov registry using the following strategy: Condition or disease: Pregnancy OR Pregnant; Other terms: Risk Factors OR Toxicity OR Teratogenesis; Risk Factors OR Toxicity OR Teratogenesis; Intervention/Treatment: Antipsychotic OR Antipsychotics OR Antipsychotic drugs OR Antipsychotic medications.

The search strategy was approved by all authors. All authors established eligibility criteria through Delphi rounds, in which they all met in person and discussed the issues of the review. T.C., D.S., F.M.L., G.D.K., F.D.S., F.F., A.A., A.Q., F.G., C.B., O.M.F., G.B., R.C., S.R., and Giul.Mas. Carried searches independently labelled each paper according to the inclusion/exclusion dichotomy and discussed eligibility according to our pre-established criteria. Each excluded paper received a label according to the reason for exclusion. When there were multiple reasons to exclude a study, we chose one (the main one was generally selected). Consensus was reached through Delphi rounds among all authors, which were repeated until all authors agreed on every issue raised. Delphi rounds were supervised by G.D.K., M.M., and G.S.. No more than two were needed to reach complete agreement among authors.

### 2.1 Inclusion Criteria

Eligibility depended on being a clinical study with results on antipsychotic(s) given during pregnancy and/or during lactation, reporting on neurodevelopmental outcomes or fetal toxicity (teratogenicity, malformations, obstetric complications impinging on fetal/newborn health *etc*.), physical outcomes at birth, mother outcomes, including psychiatric symptoms during the puerperium. Missing data, if not negligible, were imputed using the Effective Strategies for Handling Missing Values in Data Analysis (Updated 2023) by Nasima Tamboli, AnalyticsVidhya — Published on October 29, 2021 and Last Modified on July 14^th^, 2023, accessed on August 25, 2023 at https://www.analyticsvidhya.com/blog/2021/10/handling-missing-value/ and noticed on the risk-of-bias the attempts at addressing them in the various papers.

### 2.2 Exclusion Criteria

**Fig. (1):**
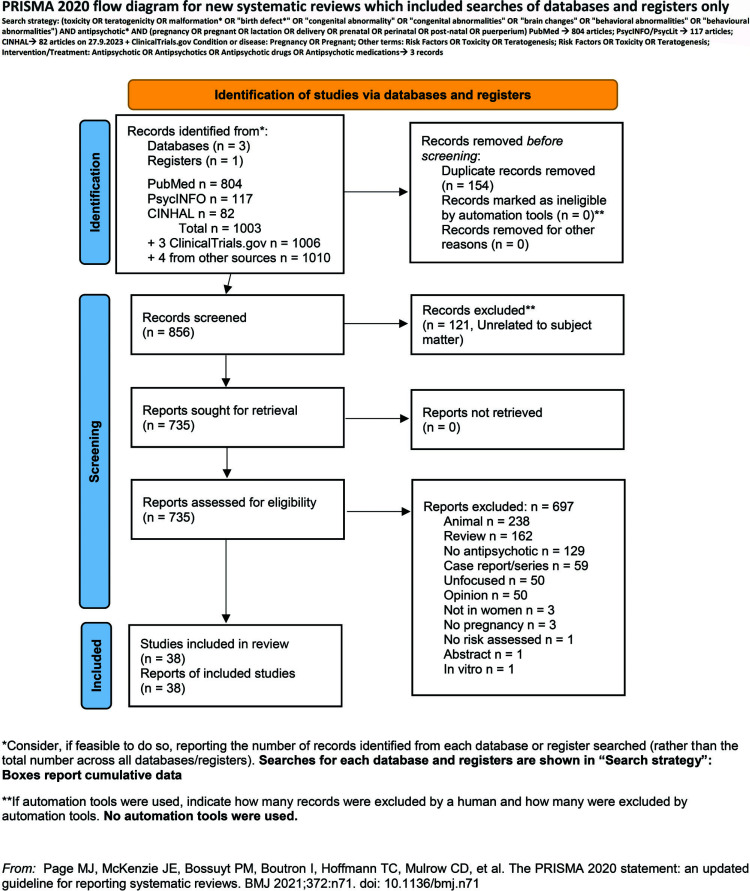
PRISMA flow diagram of our search (From Page *et al*., 2021 [14]).

Excluded were studies not performed on pregnant or lactating women (labeled Not in women or No pregnancy), those not assessing risk outcomes (No risk assessed) like basic science studies using animals or *in vitro* techniques (labeled respectively as Animal or *In vitro*), those not involving the administration of antipsychotics or pooling the data of antipsychotic administration with the data of other drug categories (labeled No antipsychotic), those assessing other outcomes with respect to what we aimed to investigate (labeled unfocused), studies emerging from our search but were unrelated to the object of our investigation (labeled Unrelated), case reports or series (termed Case), reviews, guidelines, and meta-analyses (labeled Review; however, we carefully searched their reference lists to identify studies that possibly eluded our search), editorials, hypotheses, models, opinions, letters to the editor or comments on published studies, collectively termed Opinion, and congress-meeting abstracts without sufficient data (labeled Abstract). We also did not include studies carried out on the same population (labeled as Same as#), but we kept such studies if they focused on different outcomes. Given that we searched multiple data sources, we started out with PubMed and the records of other databases that were identical to those of PubMed were labeled Duplicates and removed from the total count (Table **S1**, Fig. **1**).

To conduct our review, we adopted the Preferred Reporting Items for Systematic Reviews and Meta-Analyses (PRISMA) statement [14]. The checklist may be found in the supplemental online content (Table **S3**). To assess the risk-of-bias (RoB) of eligible studies, we used the Risk of Bias In Non-randomized Studies of Exposure (ROBINS-E) tool [15]. The results of the RoB are shown in the supplement, Table **S2**.

We did not register our review to PROSPERO due to problems encountered with their software. The site responsible for registering reviews has remained unresponsive to our solicitations. We did not prepare a review protocol, as we think it is excessive to adhere to all increasing impositions of international self-elected regulatory and bureaucratic agencies.

## 3 RESULTS

Our searches were carried out on the aforementioned databases, and the ClinicalTrials.gov register on the 27^th^ of September 2023 and produced 804 records on PubMed, 117 on PsycINFO, and 82 on CINAHL, while on ClinicalTrials.gov it yielded 3 records. Further, three studies were identified from other sources or review reference lists. Detailed results with reasons for exclusion are shown in Fig. (**1**) and Table **S1**. After removing duplicates, there remained 856 records. Of these, 38 were deemed eligible, hence 818 were excluded. Excluded articles were in decreasing order of number per reason Animal n = 238; Review n = 162; No antipsychotic n = 129; Unrelated n = 121; Case report/series n = 59; Unfocused n = 50; Opinion n = 50; Not in women n = 3; No pregnancy n = 3; No risk assessed n = 1; Abstract n = 1; and *In vitro* n = 1.

Records resulting from the search strategies spanned from December 15, 1955, to July 17, 2023, including papers ranging from February 1975 (accepted on July 17, 1974) to Apr 2, 2023 (accepted on March 28, 2023). Of the included studies, 31 were conducted in a single site but could involve the analysis of data from more than one database or country. The other 7 studies were multicentre, with the number of involved sites ranging from 2 to 20. Three of them were international (US-Canada, US-Northern European countries, and Northern European countries), and the other four were based in individual countries. The US contributed to most studies (n=12); in particular, Boston, Massachusetts, was an investigational site in 10 studies, of which 6 involved the Massachusetts General Hospital Group, which uses the purposely established National Pregnancy Registry for Atypical Antipsychotics (NPRAA) in the context of a wider project also involving registries for antidepressants, ADHD medications, and sedative/hypnotics and other sleep medications, and three used the medical insurance Medicaid database. Other US sites involved were based in California, Indiana, Florida, Ohio, Massachusetts (Lexington), Georgia, Illinois, Rhode Island, and Pennsylvania. Canada contributed to 4 studies (2 conjointly with the US), while France alone contributed to 3 studies, the United Kingdom to 2, Germany to 2, India to 2, Israel to 2, Australia to 2, and New Zealand, Japan, South Korea, and The Netherlands to 1 each; of the Northern countries which collaborated in common projects, Finland contributed to 4 studies (2 Finland alone), Denmark to 3 (1 alone), Sweden to 3 (1 alone), Iceland and Norway to 2 each. The sum of the sites exceeds the number of studies due to multiple author affiliations or to participation of more than one site in a study.

All 38 eligible studies were longitudinal, although some assessed data only once since most data were collected either prospectively or retrospectively (Table **1**) [16-54]. Of these, 25 were prospective, 11 were retrospective, and 2 were both retrospective and prospective [22, 38]. Most studies focused on major malformations as outcomes, on live birth rate (and/or death rate, abortions, spontaneous or elective, or stillbirth) and neurodevelopmental outcomes, including Apgar scores, intelligence quotient, fetal growth restriction, and newborn weight. Almost all required exposure to antipsychotics at least in the first trimester of pregnancy to compare with unexposed patients from the general population or from control groups with similar mental disorders to those for which the antipsychotics were prescribed in the experimental groups. Early studies (around the mid-late Seventies) were those with the most methodological flaws. One study was found at high risk of bias and another with some concerns; all other studies were at low risk of bias (Table **S2**). One study focused on the ability of thioridazine to reverse unexplained infertility [21], another on the possible teratogenicity of phenothiazines given as anti-nausea or antiemetic agents in women with no mental health problems [17], while only 2 studies observed infant-related outcomes with lactation [22, 27]. They both concluded that there are no infant-related harms associated with continued use of olanzapine during lactation. Of the 38 eligible articles, 22 found no increase in maternal or foetal/new-born risks [16, 17, 21, 22, 24, 27, 30, 31, 33, 36-38, 40, 44, 45, 47-53], 5 were cautious [19, 26, 28, 32, 54], 5 reported mixed results [20, 23, 25, 34, 35], pointing to APs not being related with adverse maternal/foetal/neonatal outcomes, but with some concerns for specific drugs, 1 found second generation antipsychotic (SGA) polytherapy to be associated with increased risks for maternal and new-born outcomes, while SGA monotherapy was associated with decreased risks [29]. There were 2 studies indicating some increase in adverse maternal and neonatal outcomes with SGAs in pregnancy [39, 42], and 2 other showing a definite increase of maternal and neonatal adverse outcomes, one involving olanzapine and musculoskeletal malformations [43] and another the use of long-acting injectable (LAI) formulations and obstetric complications [46]. Finally, 1 study did not draw any conclusion, as “the small exposed case counts limited the identification of associations between antipsychotic use and major congenital malformations” [44]. Usually, even in the most sceptical of these reports, addressing confounders like nicotine and alcohol use and other types of unhealthy lifestyle reduced the risks to figures similar to those of the general population. Summarizing the evidence, there were no increased risks of major malformations in children of women who used APs during their pregnancy, but rather, it is the very presence of psychotic disorders, like schizophrenia and bipolar disorder [16, 48] or the associated dysregulated lifestyle [49], that constitutes a risk factor for malformations.

## 4 DISCUSSION

In this systematic review, we found that the use of antipsychotic medications during pregnancy does not substantially increase the odds of adverse maternal or newborn outcomes. Most evidence was favorable for the use of antipsychotics throughout pregnancy and lactation (although the evidence regarding lactation is sparse), and even the reports pointing to the need to be cautious, they recommend not to discontinue the treatment if the condition needs it. All reports agree on the fact that a careful consideration of the risk-to-benefit ratio must precede a clinical decision to continue or discontinue the antipsychotic medication. Although the risks for the newborn may not approach zero, discontinuing the antipsychotic drug during pregnancy may even increase the occurrence of malformations. Women with a mental illness for which antipsychotic medication is needed may constitute a vulnerable population during pregnancy; the increased probability of adverse maternal and fetal/neonatal outcomes could be more related to the background psychopathology than the medication itself. Hence, discontinuation could not decrease the harm rate but either leave it unaffected or even increase it.

The eligible studies span from the pre-DSM-III era to the last few months. During this five-decade interval, many new antipsychotics were introduced, so the focus of attention of studies has consequently changed. In the early seventies, clozapine was introduced as another antipsychotic in some mid- and North-European countries, but in 1975, it was withdrawn from the market due to a number of occurrences of agranulocytosis [55]. During the late eighties, Herbert Meltzer, having noticed the particular clinical and receptor profile of clozapine (in particular, its higher affinity for the serotonin 5-HT_2_ receptor compared to its affinity for the dopamine D_2_ receptor), helped it to regain its status among antipsychotics and called it “atypical” [56], thus creating a split between neuroleptics, which were termed first-generation antipsychotics (FGAs) and the newer, second-generation antipsychotics (SGAs), consisting of any new drug being introduced after clozapine (which shared to a certain extent the high affinity for 5-HT_2_ receptors) plus other existing drugs that wished to join the SGA club (some years after being introduced, the substituted benzamide amisulpride was claimed to be an “atypical,” despite lacking any 5-HT_2_-inhibiting activity [57]). Since the introduction of SGAs, FGAs have been subjected to vexation from scientists and the pharma industry, mainly due to their cardiotoxic potential; suddenly discovered after some 50 years, they were consolidated in clinical practice. In the field of pregnancy, the FGA-SGA comparisons yielded no differences in their teratogenic potential [42, 46]. SGAs did not show a high potential for major congenital malformations [45, 47], but it cannot be ruled out that they are related with atrio-ventricular septal defects [28]. While they were shown to lower the risk of fetal complications when given in monotherapy, their use in polytherapy was related to adverse mother and child consequences [29]. Some SGAs impair glucose metabolism, and as of consequence, they may increase the likelihood of pregnancy complications [42].

The drugs focused upon were among phenothiazines chlorpromazine, prochlorperazine, thioridazine, levomepromazine, dixyrazine, fluphenazine, and perphenazine, the antihistaminic promethazine, the butyrophenones haloperidol, penfluridol, and melperone, among thioxanthenes flupenthixol, chlorprothixene, and zuclopenthixol, the diphenylbutylpiperidine pimozide, the substituted benzoamides amisulpride and L-sulpiride, whereas among SGAs, the benzodiazepines clozapine, olanzapine, zotepine, and quetiapine, among pyridopyrimidines risperidone and its metabolite paliperidone, and the piperazines ziprasidone, lurasidone, and aripiprazole. Some teratogenic potential is suspected for butyrophenones [20, 23], especially limb defects. Increased birth weight has been reported when mothers took olanzapine during pregnancy [26] and decreased newborn weight at birth when mothers took aripiprazole [39]. Weight gain and weight loss are reported respectively for olanzapine and aripiprazole in common clinical practice [58, 59]. It should be remembered that maternal weight affects the baby’s weight at birth [60], so it could be that drugs affecting the mother’s weight also affect the baby’s weight in the same direction they affect the mother’s weight.

The data we gathered here come from around the globe, from North America to the Far East to Australasia. Most contributions are from the North-America and Europe. Notable absences are Eastern and Southern Europe, near and middle-East, with the exception of Israel, South America, and Africa. Australia and New Zealand tend to have opposite results, with Australia being more negative towards the administration of antipsychotics during pregnancy [39, 46], while New Zealand had data favorable to their administration [36]. The Northern European countries, especially Denmark, have long-established databases of health-related data of their citizens to probe. Although Dutch nationwide hospital registries have been active since 1963, their data are anonymized and impossible to connect to cases [61]. The Danish National Patient Registry was established in 1977 [62] and was preceded by a similar Finnish registry (1969) and followed by Swedish (1987), Icelandic (1999), and Norwegian (2008) registries [61]. These registries united their forces to investigate the effects of drugs on pregnancy outcomes [48] and collaborated with a US insurance-related database [50]. These databases are likely to yield reliable results compared to individual studies that failed to collect sufficient numbers of cases and were generally underpowered. It should be emphasized that there have been methodological improvements through the years that allow data to become more reliable. More recent studies are likely to bring methodological improvements over the old ones. In 2008, the Massachusetts General Hospital (MGH) in Boston, US, established the NPRAA, a hospital-based registry modeled after the North American Antiepileptic Drug Pregnancy Registry, to collect safety data on fetal exposure to SGAs [63]. The NPRAA is now part of a larger effort, the National Pregnancy Registry for Psychiatric Medications, which includes other registries intended to collect safety data of mothers and children exposed to psychiatric drugs during pregnancy, like The National Pregnancy Registry for Antidepressants, The National Pregnancy Registry for ADHD Medications, and The National Pregnancy Registry for Sedative-Hypnotics and Other Sleep Medications. The way the MGH collects data enables the group to address confounders and minimize recall and selection biases [51]. The disadvantage with respect to the Nordic countries’ databases is that the NPRAA has no national coverage, but the sample under observation is well-known by the hospital staff, and this reduces evaluation errors. The comparisons regard a homogeneous sample and not a standard general population, which were the comparison groups in Australasian and other studies and are likely to miscalculate the absolute risk due to the fact that data in general populations are likely to change with time [64] and the comparison is not parallel, as in the NPRAA data. In the NPRAA, the comparisons are between women aged 18-45 years who were exposed at least during their first trimester of pregnancy to one or another SGA and those women who were not exposed to an SGA, *i.e*., a parallel group that confers statistical strength to the comparisons.

In summary, we may state with a fair margin of confidence that antipsychotic medication during pregnancy is not likely to increase significantly the odds for maternal or fetal/neonatal adverse outcomes and should not be withheld from patients needing them. However, considering the entire sample of the studies included here, the patients involved were not in sore need of antipsychotic treatment. Many were with unspecified mental illness, and some had ill-defined schizophrenia [16], the disorder for which antipsychotic medications are most indicated and that bears the highest risks for adverse pregnancy outcomes independently from medication; other women had other psychiatric diagnoses (for which antipsychotics are not recommended, *e.g*., depression [36, 40, 45, 47] or anxiety [36, 40, 45] or personality disorders [36], but it could be that these were comorbidities or that these specific patients required antipsychotics, we have to rely on visiting clinicians’ judgments), still, other women were with bipolar disorder [37, 40, 47, 48], a currently fashionable psychiatric diagnosis that has antipsychotics among its treatment options [65].

The treatment recommendations for pregnant women who need antipsychotic treatment that one may derive from our review are in line with those of other similarly aimed reviews. Despite most of them being quite distant in time and methodologically dissimilar (and with some pitfalls), they all reached similar conclusions, *i.e*., there are no sufficient data to conclude that administering antipsychotics in pregnant women during any trimester increases the risk for major congenital malformations [66-69]. All reviews agree that risk-to-benefit must be taken into account [67-69]. The best option when a patient is already taking antipsychotics is to continue treatment [68]. The evidence of potential teratogenic effects of antipsychotics is regarded as “conflicting and inconclusive” [70]. Since most studies do not fit quality requirements, their results may be regarded as inconclusive. Hence, women must be closely monitored [71]. The evidence of the effects of mental illness on birth outcomes is rarely taken into account, and results are inconclusive to conflicting [69]. We suspect that should the effects of mental illness on offspring be identified, some of the effects previously attributed to antipsychotic treatment would see their aetiology change their basis.

Still, another issue is polytherapy. Antipsychotic polytherapy, and which antipsychotics are combined in clinical practice, usually reflects a clinician’s therapeutic attitudes relating to a particular symptom pattern in a particular patient [72]. Polytherapy represents more than one-third of current clinical practice and shows a trend to increase [73]. While this practice is consolidated in routine clinical practice in nonpregnancy conditions, in pregnancy, it carries increased risks for adverse outcomes, while on the contrary, SGA monotherapy actually decreases the risks of fetal complications [29]. It is advisable to choose just one antipsychotic during pregnancy.

## 5 LIMITATIONS

Limitations regard both our search strategy *(mea culpa)* and the included studies *(sua culpa)*. We could not limit our search to specific disorders but obtained studies with heterogeneous diagnoses, sizes, and effect sizes. However, had we excluded all diagnoses for which antipsychotic medications are not recommended, we would not have obtained any findings to comment. We cannot control for sample overlap in studies by the same research groups and on the same database. It is certain that the two studies from the UK databases had sample overlap, but we decided to keep them both, as the two studies reported partially on different outcomes [33, 34], but it is also presumable that the NPRAA studies used different women for each antipsychotic they tested [31, 40, 44, 45, 51, 52], although it is not sure they used entirely different control groups of unexposed women. Given the extreme diagnostic heterogeneity, we were unable to perform a meta-analysis.

## CONCLUSION

We are at the beginning of reliable data collection for the health risks of antipsychotics in pregnant women and their children. The first data date back to the mid-seventies, but currently, the number of studies that deal with this subject is increasing (1975-2005: 8 studies, 2006-2015: 6 studies, 2016-2020: 12 studies, 2021: 3, 2022: 4, 2023: 5), thus reflecting the current interest in this subject. Most studies agree that there is no reason to worry about women who have been exposed to antipsychotics during the first trimester, nor is it recommended to discontinue their antipsychotic treatment if they really need it. Some concerns have been raised for haloperidol regarding limb malformations, for olanzapine regarding increased and for aripiprazole regarding decreased weight at birth of children exposed to the aforementioned drugs, but the evidence against the use of antipsychotics in pregnancy is not strong. The small increase in adverse pregnancy outcomes could be accounted for by the underlying psychiatric disorder.

## Figures and Tables

**Table 1:** Summary of included studies in chronological order, from older to newest.

**Study**	**Location ** **(*n* of Sites, ** **Databases)**	**Population**	**Design**	**Results**	**Conclusions/** **Observations**
Rieder *et al*., 1975 [16]	Bethesda, Maryland, USA (1, Collaborative Perinatal Study of the NINDS, of 12 centers selected only the Boston group)	Of 9,280 ♀ followed up since 1959 in Boston, 186 mothers reported she, her husband or both had a previous psychiatric hospitalization. Excluding 2 who underwent therapeutic abortion, there remained 184 whose psychiatric records were followed up from 11 years after first delivery (*x̄* age at delivery = 27.0 yrs). Patients were classified according to DSM-II-derived criteria in groups with schizophrenia, possible schizophrenia and non-schizophrenia and were matched to ctrl groups from the NINDS.	Observe any difference in fetal/neonatal birth rate and reason of death between index groups and ctrl.	The group of schizophrenias had 7 deaths in 93 births (7 deaths in 186 births in the ctrl group, *p =* 0.053), but there were more deaths for unknown causes among mothers with schizophrenia and in the combined psychiatric group than among ctrl (*p <* 0.01). Newborns of mothers in the ctrl group, when delivery ended in death, had identifiable causes of death, like obstetric complications. Slightly ↑ death rate in the group receiving phenothiazines, but individual drugs were not specified.	There is no evidence that drugs taken for schizophrenia are linked to a greater risk of premature fetus/infant death, but the sample was not sufficiently powered to detect drug effects; it appears that having schizophrenia could be related to ↑ odds of death for unknown cause.
Milkovich & van den Berg, 1976 [17]	Oakland-Berkley, California, USA (1)	11,481 pregnant ♀, of which 6,432 complained of vomiting/nausea who were subdivided into a medicated (n = 1981, *x̄* age = 27.0 yrs; receiving phenothiazine, prochlorperazine, meclizine, cyclizine, trimethobenzamide, and Bendectin [doxylamine/dicyclomine/ pyridoxine]) and an unmedicated group (n = 4,451, age *x̄* = 27.3 yrs)	Longitudinal, prospective, observational study to assess the impact of specific anti-nausea and anti-vomiting medications on fetal development; outcome: occurrence of congenital anomalies and perinatal deaths in newborns	Comparing the medicated group (n = 1,981) *vs.* the unmedicated group (n = 4,451), the rate of severe congenital anomalies did not differ in the two groups during follow-up (1.7% *vs.* 2.5% at 1 month; 2.5% *vs.* 2.2% at 1 year; 3.7% *vs.* 3.2% at 5 years). Comparing the medicated group (n = 1,981) *vs.* the unmedicated group (n = 4,451), the rate of perinatal deaths was not significantly different in the two groups (27.8% *vs.* 32.1%). Results did not change when considering the specific drugs separately. This is true also for congenital anomalies, with the exception of trimethobenzamide, which in the age category of 5 yrs, showed an excess number of SCA (*p* < 0.05)	No teratogenicity is associated with the phenothiazine derivatives. However, trimethobenzamide showed a trend to a potential association with severe congenital abnormalities. Many incongruences among the provided data; while there was no indication for Bendectin (Doxylamine/Dicyclomine/ Pyridoxine) an association with birth malformations, and this has been confirmed in a subsequent meta-analysis [18], the drug was withdrawn in 1983
Slone *et al*., 1977 [19]	Boston, Massachusetts, USA (1, Collaborative Perinatal cohort)	Total sample: 50,282 mother-child pairs. Among them, 3,675 were exposed to phenothiazine APs during pregnancy	Longitudinal, prospective cohort study assessing the impact of phenothiazine exposure during the first four lunar months of pregnancy on congenital malformations, perinatal mortality, birth weight, and IQ score	Congenital malformations rate was similar comparing heavy (n = 403, 6.2%) and intermediate exposure (n = 906, 7.6%) with non-exposure (n = 48,973, 6.4%). Comparing exposed (n = 1,309) to non-exposed (n = 48,973) per specific malformation, only cardiovascular malformations were borderline significantly associated with the exposed group (*p* ≈ 0.05). Comparing exposed and non-exposed groups, perinatal mortality rate (n = 3,056; 1.2% exposed *vs.* n = 38,281; 1.0% non-exposed), birth weight (n = 2860 exposed *vs.* n = 35,353 non-exposed), and *x̄* IQ scores (n = 2,141; *x̄* = 96.8 exposed *vs.* n = 26,217; *x̄* = 97.0 non-exposed) were similar	While a definitive conclusion regarding the non-teratogenic nature of phenothiazines cannot be drawn from the results, there is a lack of compelling evidence indicating fetal harm from the use of phenothiazines during pregnancy. Even after accounting for confounders, results did not change, but the detailed results were not reported. The age of mothers not reported
Godet & Marie-Cardine, 1991 [20]	Bron, Lyon, France (1, 2 French records of malformed children)	199 ♀ with schizophrenia delivering from 1984 to 1989 from the first record; 246 children with malformations from mothers exposed to APs during the 1^st^ trimester from the second record	Retrospective. Postal data collection. Self-reporting on AP use during 1^st^ trimester, 2^nd^ trimester, 3^rd^ trimester, and last month of pregnancy	2.5% malformations in children of ♀ with schizophrenia (No ↑ malformations). However, 4 malformations in 89 children exposed to at least one AP during 1^st^ trimester, of which 3 were in the 29 exposed to haloperidol. ↑ Prematurity (12.3% in the exposed sample *vs.* 5.6% in INSEE-data). No relationship between prematurity and perinatal death or prematurity and AP use (first record). No correlation between AP prescription and malformation type among 246 children with malformations (second record)	The use of APs during any trimester of pregnancy or during the last month is not associated with an increased risk of malformations. However, despite the statistical significance that could not be reached, haloperidol may carry a ↑ malformation potential than other APs, although prescription was not found to correlate with the type of malformation in actual cases of malformation.
Sharma & Sharma, 1992 [21]	New Delhi, India (1)	452 ♀ with unexplained infertility; 310 (*x̄* age = 23.4 yrs) pts put on thioridazine (discontinued on the 18^th^ day of the menses to avoid teratogenicity in case pregnancy occurred), 142 (*x̄* age = 24.5 yrs) pts put on plac	Longitudinal; 1-yr clinical trial to assess the effect of thioridazine on unexplained infertility	94 pts (30.2%) in the study group became pregnant in contrast to 22 (15.42%) in the ctrl group (*p* < 0.001). Comparing thioridazine *vs.* placebo, the incidence of abortions (4.25% *vs.* 9.09%), congenital malformations (2.16 *vs.* 4.54), perinatal mortality (5.31 *vs.* 9.09) and mode of delivery did not differ	Low-dose thioridazine is potentially safe and effective for unexplained infertility, likely due to anxiolytic properties, and not associated with teratogenic effects. Patients in the place were input on *Lactobacillus*, which may affect the microbiome.
Goldstein *et al*., 2000 [22]	Indianapolis, Indiana, USA (1, Lilly Worldwide Pharmacovigilance Safety Database)	34 olanzapine-exposed pregnant ♀; 23 assessed prospectively and 11 retrospectively	Longitudinal. Cohort-controlled (retrospective) and prospective survey exploring risks of olanzapine treatment during pregnancy	In the prospective sample, 80% normal birth, 13% spontaneous abortion, 5% stillbirth, 5% premature birth, 14% with perinatal complications, 0% post-natal complications and 0% major malformations. Proportions were similar to those of the retrospective sample. Two cases of lactation exposure in the retrospective group did not suggest infant risk	Olanzapine during pregnancy and breastfeeding shows no significant additional risk to the baby. However, using olanzapine during pregnancy requires balancing benefits against potential risks to the fetus. The mother’s age was not reported.
Diav-Citrin *et al*., 2005 [23]	Jerusalem, Israel (4)	215 pregnant ♀ with exposure to haloperidol or penfluridol compared to a control group of ♀ exposed to nonteratogenic agents. Offspring followed up between the neonatal period and 6 years	Longitudinal. Multicenter, prospective, controlled cohort study. Outcome: major malformations, ETOP rates, live births in AP-exposed *vs.* unexposed, % cesarean sections	In the exposed group, ↑ ETOP rate. No difference in the rate of major anomalies when the group was exposed in the 1^st^ trimester. ↓ GA, ↓ median BW 215 g, 2-fold ↑in the rate of preterm births,↓ 165 g in BW in the exposed group in full-term infants. ↑ proportion of cesarean sections (25.5% *vs.* 16.3%, *p* = 0.014) in the exposed group. No significant differences in the rate of miscarriages, ectopic pregnancies, or stillbirths	Butyrophenones do not represent a major teratogenic risk in humans. A possible association between limb defects and butyrophenones cannot be ruled out. In pregnancies with 1^st^-trimester exposure to butyrophenones, level II ultrasound is advised, with an emphasis on the limbs.
McKenna *et al*., 2005 [24]	Toronto, Ontario, Canada (1)	151 ♀ who took an atypical AP in the first trimester compared to 151 ♀ who were exposed to non-teratogenic agents	Prospective, controlled cohort study; outcomes: malformations, spontaneous abortions, obstetric and neonatal complications	Among exposed women, nominal ↑ rate of spontaneous abortions (14.5% *vs.* 8.6%, *p* = 0.15, ns), but no statistical significance. *x̄* GA and *x̄* BW not different between the groups (*p* ≈ 1; *p* = 0.38, respectively). The rates of major malformations were not statistically different (*p* ≈ 1). No statistical differences in the rates of complications during labor and in the rates of neonatal complications	Atypical APs do not appear to be associated with an increased risk for major malformations.
Reis & Källén, 2008 [25]	Linköping-Lund, Sweden (1)	958,729 ♀ with 973,767 infants. Among them, 2,908 ♀ reported the use of APs in early pregnancy (2,260 ♀ had used dixyrazine or prochlorperazine and 570 ♀ other APs). Distribution of mothers per age: <25 yr n = 153,671; 25-34 yrs n = 641,057; ≥ 35 yr n = 164,001. APs analyzed: chlorpromazine, levomepromazine, dixyrazine, fluphenazine, prochlorperazine, perphenazine, thioridazine, haloperidol, melperone, flupenthixol, chlorprothixene, zuclopenthixol, pimozide, clozapine, olanzapine, quetiapine, risperidone	Prospective, observational registry study evaluating fetal complications related to early-stage pregnancy use of FGAs and SGAs	Among the exposed group, a significant ↑ of congenital malformations was found compared to the non-exposed group (OR = 1.52, 95% CI 1.05 to 2.19), mainly regarding cardiovascular defects (A-V septum defects) without specificity for individual drugs. As regards mother involvement, 2-fold ↑ likelihood of GDM and 40% ↑ cesarean delivery compared to the non-exposed group	The use of APs during early pregnancy is not strongly correlated with a significant ↑ risk of congenital malformations. However, there exists a possibility of a moderate ↑ risk. The outcomes indicate that the moderate risks associated with AP use in pregnancy should not serve as a barrier to essential treatment for ♀ when considering potential benefits. APs do not differ from one another in their risk of congenital malformations.
Babu *et al*., 2010 [26]	Bengaluru, Karnataka, India (1)	37 pregnant ♀ (*x̄* age = 26.3 ± 4.98) treated with psychotropic medications. 12 ♀ exposed to olanzapine monotherapy (olanzapine), 12 to olanzapine with other psychotropic medications (olanzapine + other), and 13 to psychotropic medications other than olanzapine (other)	Longitudinal. Prospective, comparative study to examine the effect of olanzapine on infant birth weight. The study compared infants exposed to olanzapine alone, olanzapine + other, and other.	The mean weight in the olanzapine + other group was 3,008 g, olanzapine monotherapy 3,310 g and other group 2,921 g. Analysis of variance showed significant ↑ weight among infants in the olanzapine monotherapy group (*p* = 0.02) when compared with the other two groups. Moreover, *post-hoc* analysis showed significant ↑ birth weight among the olanzapine group (n = 12) compared with the olanzapine + other group (*p* = 0.037)	This study provides evidence suggesting a potential link between prenatal exposure to olanzapine and ↑ birth weight in newborns.
Gilad *et al*., 2011 [27]	Petah Tiqwa-Tel Aviv-Yaffo, Israel (1, Beilinson Teratology Information Service (BELTIS), Rabin Medical Center, Petah Tiqwa, Israel)	88 ♀, of whom 22 (*x̄* age = 31.3 ± 5.6) breastfed while taking olanzapine, 15 (*x̄* age = 30.7 ± 5.3) continued to take olanzapine but did not breastfeed, and 51 (age *x̄* = 31.9 ± 4.4) breastfeeding mothers while taking drug known to be safe during lactation	Longitudinal. A prospective, controlled observational study evaluating the impact of olanzapine exposure during lactation on infants	Comparing olanzapine-exposed breastfed infants to ctrl breastfed infants, both groups exhibited a comparable duration of breastfeeding and comparable rates of adverse outcomes. Nonetheless, the olanzapine-exposed breastfed group showed an ↑ incidence of early discontinuation of breastfeeding (5/22 *vs.* 0/51, *p* = 0.02). No congenital birth defects were identified among the 30 newborns who had been exposed to olanzapine *in utero*. Infants exposed to olanzapine showed significantly more neonatal symptoms than those not exposed to olanzapine (20% *vs.* 3.9%; *p* < 0.05). Withdrawal syndrome was observed in 3/30 (10%) infants.	No observed ↑ adverse long-term effects in breastfed infants exposed to olanzapine. Findings suggest that breastfeeding can continue for ♀ undergoing olanzapine treatment. Nevertheless, infants exposed to olanzapine should receive continued monitoring.
Habermann *et al*., 2013 [28]	Berlin-Freiburg, Germany (1 Teratology Information Service-Berlin)	1967 ♀ of whom 561 pregnant (age *x̄* = 32, IQR = 28-35) exposed to SGAs (olanzapine n = 187, quetiapine n = 185, clozapine n = 73, risperidone n = 64, aripiprazole n = 60, ziprasidone n = 37, amisulpride n = 16, zotepine n = 2), 284 (*x̄* age = 32, IQR = 27-36) exposed to FGAs (haloperidol n = 64, promethazine n = 86, and flupentixol n = 44) and 1122 (age *x̄* = 32, IQR = 27-36) using drugs known not to be harmful to the unborn (ctrl group)	Longitudinal, prospective, cohort study exploring infant risks related to SGA and FGA exposure during pregnancy	↑ Incidence of major malformation in the SGA group compared to the ctrl group (adjusted OR = 2.17; 95% CI: 1.20 to 3.91). Postnatal disorders occurred significantly more often in both SGA (15.6%) and FGA groups (21.6%) compared to ctrl (4.2%). The incidence of elective terminations of pregnancy was ↑ in the SGA (21%) and FGA (17%) groups, compared to ctrl (3%). Preterm birth and low birth weight were more common in the FGA group. Neonatal symptoms were more likely to occur in the SGA and FGA groups compared to the ctrl group.	The rates of malformations showed no difference between children prenatally exposed to SGAs or FGAs; however, the possibility of ↑ risk for A-V septal defects → first-trimester use of SGAs cannot be entirely dismissed. The ↑ incidence of major malformations in the SGA group may reflect a detection bias concerning A-V septal defects. Due to the ↑ risk of postnatal disorders in neonates exposed to SGAs or FGAs during the final week of gestation, it is advisable to schedule deliveries in medical facilities equipped with NICUs
Sadowski *et al*., 2013 [29]	Toronto, Ontario, Canada (1, Motherisk Program)	133 ♀ (age at conception in months *x̄* = 378.33 ± 61.79) exposed to SGAs (n = 37 with SGAs in monotherapy; n = 96 with SGA and other psychotropic drugs) and 133 matched HCs (age in months *x̄* = 683.61 ± 53.92)	Longitudinal, prospective, cohort study investigating the reproductive safety of SGAs	72% of pregnant ♀ received SGAs combined with other medications. Pregnancy outcomes for live births (n = 113 in the exposed group and n = 116 in the non-exposed group), excluding miscarriages and fetal deaths (which showed no significant difference between the two groups). Exposed ♀ displayed ↑ pre-pregnancy weights, ↑ associated comorbidities, and ↑ rates of instrumental deliveries. Moreover, ↑ % of neonates of SGA-exposed mothers were LGA (*p* = 0.022). Maternal weight gain during pregnancy did not differ significantly between exposed and HC groups, nor between the subgroups exposed to SGA mono- and poly-therapy. Neonates in the exposed group were more likely to be born prematurely (10.6% *vs.* 4.3%), frequently required NICU admissions (25.3% *vs.* 9.5%), and exhibited signs of poor neonatal adaptation (16.5% *vs.* 5.2%). While not reaching statistical significance, the rate of major malformations was approximately ×2½ ↑ in the exposed compared to the HC group (6.2% *vs.* 2.6%). Adverse neonatal outcomes were mainly observed in the poly-therapy group.	Polytherapy with SGA medications was linked to unfavorable pregnancy outcomes for both maternal health and the well-being of the child. On the other hand, exposure to SGA monotherapy during pregnancy seems to carry a ↓ risk for fetal complications. However, as the authors admitted, “The sample size necessary to define a twofold increase of malformation rates of 1-3% above the baseline was insufficient.”
Bellet *et al*., 2015 [30]	Saint-Etienne-Lyon-Paris, France (20, Terappel and Paris TIS databases)	86 ♀ exposed to aripiprazole from 4-10 GWs (*x̄* age = 31.8 ± 5.8 yrs [range 18-48 yrs]) *vs.* 172 unexposed ctrl (*x̄* age = 31.4 ± 5.4 [range 16-42 yrs]) (1:2 ratio, matched for age and GW) referred to Terappel (20 pharmacovigilance sites) and the Paris TIS between July 2004 and December 2011	Longitudinal. Observational prospective cohort; Primary outcome: MCM not related to a chromosomal defect or clinical genetic syndrome; Secondary: miscarriage, abortion, stillbirth, preterm birth, FGR, neonatal and maternal complications	Two major malformations each for the aripiprazole-exposed and unexposed groups (despite the rate was double in the former, there was no statistical significance; OR 2.30, 95% CI from 0.32 to 16.7); no difference in minor malformations (OR 2.03, 95% CI from 0.66 to 6.28). No differences in all secondary outcomes, save for Apgar scores at 1 and 5min, which were ↓ in aripiprazole-exposed newborns compared to unexposed (8.9 *vs.* 9.7 at 1 min and 9.7 *vs.* 9.9 at 5min, respectively) and preterm birth rate and FGR among the exposed group	Aripiprazole is not related to negative pregnancy and fetal/neonatal outcomes; however, the study was somewhat underpowered, with at least 200 pregnancies exposed during the first trimester to ensure a 3-fold risk ↑ compared to the general population.
Cohen *et al*., 2016 [31]	Boston, Massachusetts, USA (1, NPRAA)	303 ♀ aged 18-45 yrs (*x̄* age = 32.3 ± 5.1) were included; 214 first-trimester exposure to SGAs (*x̄* age = 31.9 ± 5.4), 89 unexposed (*x̄* age = 33.3 ± 4.07; *p* = 0.04)	Registry examination of records of women with psychiatric illness exposed and unexposed to SGAs; Outcome: risk of major malformations	There were 3 occurrences of major malformations in the SGA-exposed group compared to 1 in the unexposed. The absolute risk of major malformations was 1.4% for SGA-exposed and 1.1% for unexposed OR for exposed *vs.* unexposed infants = 1.25 (95% CI from 0.13 to 12.19. Risk estimate 1.4% (95% CI from 0.29 to 4.04) for exposed and 1.1% (95% CI from 0.0 to 6.10) for unexposed. SGA-exposed and unexposed did not differ in malformation risk.	SGA exposure in the first trimester is not related to major malformations. However, confounders have not been addressed. Relatively small sample and no general population comparison
Montastruc *et al*., 2016 [32]	Bordeaux, Gironde-Toulouse, Occitanie, France (1, VigiBase^®^ database {WHO}, investigated data from 123 countries)	Of 10,304,819 reports, 42,502 of which were related to ‘congenital, familial and genetic disorders’; 351,028 reports (3.4%) of AP exposure, of which 1235 (0.35%) related to events of ‘congenital, familial and genetic disorders’	Used data from the spontaneous reporting WHO database Vigibase^®^ (1967-2014) focusing on GI malformations and identifying SDRs; reports related to drug competitors (antiepileptics, antidepressants, antivirals) and movement disorders were removed.	Three SDRs identified, ‘palate disorders congenital’ (PRR 2.1, 95% CI from 1.6 to 2.9, *χ*^2^ = 30; n = 41), ‘esophageal disorders congenital’ (PRR 2.5, 95% CI from 1.3 to 4.7, *χ*^2^ = 11; n = 10) and ‘anorectal disorders congenital’ (PRR 3.0, 95% CI from 1.6 to 5.6, *χ*^2^ = 13; n = 11). No SDR detected for APs (PRR 0.8; *χ*^2^ = 17; n = 1235). For ‘palate disorders congenital’, phenothiazines were more represented (n = 19, 70%), *i.e*., prochlorperazine. n = 6, fluphenazine n = 4, and trifluoperazine n = 2, while among SGAs, olanzapine (n = 5), risperidone (n = 4), and quetiapine (n = 4) were the most represented. For ‘congenital esophageal disorders, ’ 7 were associated with phenothiazine and 9 with SGAs (4 risperidone or paliperidone). For the 11 ‘anorectal disorders congenital’, 5 were associated with a piperazinic phenothiazine, 4 with aripiprazole and 2 with risperidone.	This study found signals for congenital GI disorders, namely cleft palate, esophageal or anal malformations associated with AP use. The most suspected were piperazine phenothiazines, risperidone, and aripiprazole. The study did not address confounders, and there is suspicion of underreporting bias.
Petersen *et al*., 2016 [33]	London-Oxford, Oxon, UK (1, THN and CPRD databases)	670 ♀ who discontinued APs, *x̄* age *=* 30 ± 5.9; 416 ♀ who were prescribed APs in pregnancy, *x̄* age *=* 32 ± 5.8; 318,434 ♀ not prescribed APs, *x̄* age *=* 30 ± 5.9; mother-child cohort: 492 ♀ who discontinued APs, *x̄* age *=* 30 ± 5.7 (children, 253 ♂ [51.4%], 239 ♀ [48.6%]), 290 ♀ who were prescribed APs in pregnancy, *x̄* age *=* 32 ± 5.6 (children, 132 ♂ [45.5%], 158 ♀ [54.5%]), 210,966 ♀ not prescribed APs, *x̄* age *=* 30 ± 5.9 (children, 107,979 ♂ [51.2%], 102,987 ♀ [48.8%])	Retrospective cohort analysis of databases 1995-2012 of records of ♀ with psychosis and their children to observe their clinical course 18 months before pregnancy, during pregnancy and 15 months after delivery. Outcomes: risk factors for drug discontinuation, estimate absolute and RRs of adverse maternal and child outcomes of psychotropic treatment in pregnancy	♀ who were prescribed APs during pregnancy had ↑ odds for cesarean section than ♀ who were not prescribed APs (absolute risk 6.6, 95% CI from 2.5 to 10.8). Children of ♀ who were prescribed APs during pregnancy had ↑ odds for poor birth outcomes than children of ♀ who were not prescribed APs (absolute risk 6.3, 95% CI from 2.8 to 9.9) and compared to children of ♀ who discontinued APs (absolute risk 5.8, 95% CI from 1.8 to 9.8). RR for GDM was 0.43 (95% CI from 0.2 to 0.93; *p* < 0.032) for ♀ who were prescribed APs during pregnancy *vs.* ♀ who discontinued APs; RR for cesarean section was 1.36 (95% CI from 1.12 to 1.64; *p* < 0.001) for ♀ who were prescribed APs during pregnancy *vs.* ♀ who were not prescribed APs. Children of ♀ who were prescribed APs during pregnancy had ↑ RR for poor birth outcome of 2.19 (95% CI from 1.28 to 3.73; *p* < 0.003) *vs.* children of ♀ who discontinued APs and of 2.44 (95% CI from 1.71 to 3.47; *p* < 0.001) *vs.* children of ♀ who were not prescribed APs. The latter was no longer significant when the parameter was adjusted, while the former was confirmed. The HR for neurodevelopmental/behavioral disorders of children of ♀ who were prescribed APs during pregnancy was 1.58 ↑ *vs.* children of ♀ who were not prescribed APs (95% CI from 1.04 to 2.40; *p* < 0.031)	The study mainly focused on the discontinuation of psychoactive drugs, lithium, anticonvulsants, and APs. ♀ prescribed APs during pregnancy were at ↑ risk of cesarean delivery, poor children birth outcomes, and neurodevelopmental/behavioral disorders than ♀ not prescribed APs. Adjusting for health and lifestyle and concomitant medication, statistics were no longer significant. No differences between ♀ prescribed APs in pregnancy and those not prescribed for birth outcomes, including MCMs. Comparing ♀ prescribed APs in pregnancy to those who discontinued treatment before pregnancy, the former had ↓ a risk of developing GDM than ♀ discontinuing APs after adjusting for health, lifestyle and concomitant medication
Petersen *et al*., 2016 [34]	London-Oxford, Oxon, UK, (1, THN and CPRD databases)	670 ♀ who discontinued APs, *x̄* age *=* 30 ± 5.9; 416 ♀ who were prescribed APs in pregnancy, *x̄* age *=* 32 ± 5.8; 318,434 ♀ not prescribed APs, *x̄* age *=* 30 ± 5.9 (the same sample as in ref. [33])	Same method as above [33]; outcomes: pre-eclampsia, gestational hypertension AND diabetes, perinatal death, and cesarean section. Prematurity and low birth weight combined into one composite outcome measure called premature/low birth weight outcome and tremor, agitation, breathing, muscle tone problems, and low Apgar score combined into a second composite outcome called adverse birth outcomes	♀ who were prescribed APs in pregnancy were more likely to deliver by cesarean section (104 of 416, 25%) than ♀ not prescribed APs (58,532 of 318,434, 18%); however, adjusting for medication and health/lifestyle significance disappeared (adjusted RR 1.09, 95% CI from 0.92 to 1.30). Cesarean deliveries did not differ between ♀ prescribed and ♀ discontinuing APs during pregnancy, even after adjustment. ♀ prescribed APs during pregnancy were at ↓ risk of developing GDM than ♀ prescribed APs (adjusted RR 0.43, 95% CI from 0.20 to 0.93). Obesity associated with GDM (adjusted RR 5.49, 95% CI from 2.67 to 11.2). 10 of 290 (3.4%) ♀ who were prescribed APs in pregnancy delivered a child with MCM, compared to 2.2% of ♀ who discontinued APs and 2.1% ♀ not prescribed APs in pregnancy; however, associations were not significant.	♀ taking APs in pregnancy bears ↑ the risk of low birth weight/premature birth. These adverse outcomes are related rather than to AP use to health/lifestyle patterns, including obesity, nicotine, alcohol, and other substance use disorders, and concomitant medications. Rather than suggesting AP discontinuation in pregnancy, interventions should focus on improving health behaviors and lifestyle
-	-	-	-	Children of ♀ who were prescribed APs in pregnancy >2-fold more likely to have prematurity/low birth weight than children of ♀ of the other two groups; adjustment attenuated the effect. Furthermore, they had ↑ odds for adverse birth outcomes *vs.* the other two groups, but this disappeared after adjustments. Typical and atypical APs did not differ in risks	-
Huybrechts *et al*., 2016 [35]	Boston, Massachusetts, USA (1, Medicaid)	1,360,101 ♀ aged 12-55 yrs; 9,258 1^st^ trimester exposure to atypical APs (x̄ age = 25.39), 733 exposure to typical APs (x̄ = 26.96), and 1,331,910 untreated/ unexposed (x̄ = 24.01)	Longitudinal. Retrospective cohort analysis of Medicaid Analytic Extract database, which included data from January 1, 2000, to December 31, 2010. Outcomes: overall MCMs and cardiac malformations identified during the first 90 days after delivery	The prevalence of congenital malformations in infants not exposed to APs was 3.27% (95%CI from 32.4 to 33.0) compared with 4.45 (95%CI from 40.5 to 48.9) of exposed to atypical and 3.82 (95%CI from 26.6 to 54.7) of exposed to typical APs. Unadjusted analyses suggested ↑ the overall risk of malformations for atypical APs (RR = 1.36; 95%CI from 1.24 to 1.50) but not for typical APs (RR = 1.17; 95% CI, from 0.81 to 1.68). After adjustment for confounders, the RR was ↓ 1.05 (95%CI, from 0.96 to 1.16) for atypical APs and 0.90 (95% CI, from 0.62 to 1.31) for typical APs	The use of APs early in pregnancy generally does not mean ↑ the risk for overall MCMs or cardiac malformations, in particular. The small ↑ in the risk for malformations observed with risperidone requires additional study.
Hatters Friedman *et al*., 2016 [36]	Auckland, Northern Island, New Zealand (1)	Forty-five pregnant ♀ (*x̄* age = 30.6 ± 6.1) were prescribed atypical APs: 21 quetiapine, 19 olanzapine, 7 risperidone, 6 aripiprazole, and 1 clozapine. ⅖ (40%) were diagnosed with BD, and almost ⅓ (31%) with a primary psychotic disorder. Further, 22% were diagnosed with MD, 13% with anxiety, and 11% with personality disorders. Only ⅕ (20%) required psychiatric hospitalization during their pregnancy.	Longitudinal. Retrospective review, including all cases treated by Auckland Maternal Mental Health (MMH) Services in which atypical APs were prescribed during pregnancy from 2012 to 2014	There were 46 live births, including twins. There was a single stillbirth at the 21^st^ gestation week (with antenatal low-dose aripiprazole exposure and the death attributed to maternal infection). GDM was diagnosed in 13% (GDM frequency generally reported = 5-9%). The *x̄* BW of 3315 g among exposed pregnancies did not significantly differ from the national *x̄* BW of 3400 g. Three infants (7%) were LBW, 2 exposed to olanzapine and 1 to quetiapine. *x̄* GA = 38.8 weeks. All infants were born later than 35 weeks, with three (7%) being premature (before 37 weeks), each of whom had been exposed to olanzapine. This was within the normal range. 2 malformations (4% of exposed pregnancies against 5.4% overall prevalence of congenital anomalies in the New Zealand general population) were noted. Perinatal complications occurred in 9%, but only 2 infants required NICU admission. Most mothers (61%) subsequently breastfed their infants.	Rates of malformation after exposure to APs were similar to the general population rate despite the AP-exposed population experiencing multiple other risk factors for negative outcomes. The risk of malformations was not ↑ and provides more naturalistic evidence for the use of APs in pregnancy.
Shin *et al*., 2017 [37]	Seoul-Jeju, South Korea (1)	One hundred sixty-two levosulpiride-exposed pregnant ♀ (*x̄ age =* 32.0 ± 3.7) and 675 age-matched pregnant ♀ who were not exposed to any known teratogenic agents were recruited. The pregnant ♀ were inadvertently exposed to levosulpiride during the 1^st^ trimester (between 0.1 and 19.5 [*x̄*: 4.8] weeks’ gestation) *x̄ dose =* 75 mg (range: 25 to 100 mg/day; cumulative dose ranged from 0.23 to 231.82 mg/kg). In the exposed group, 124 (76.5%) ♀ used levosulpiride for GI, 21 (13.0%) for respiratory, 8 (4.9%) for dermatological, 5 (3.1%) for genitourinary, 3 (1.9%) for nervous system, and 1 (0.6%) for musculoskeletal disorders.	Longitudinal, prospective cohort study investigating pregnancy and neonatal outcomes of ♀ exposed to levosulpiride in early pregnancy	There were 15 miscarriages in the 162 levosulpiride-exposed pregnancies and 32 miscarriages in the non-exposed group (OR 1.747, 95%CI from 0.922 to 3.313, 9.3% *vs.* 5.5%, *p* = 0.084). No differences when comparing BW and length, head circumference at birth, Apgar scores at 1 and 5 min, NICU admission rate, transient tachypnea of the newborns or neonatal jaundice between exposed and unexposed newborns (*p* > .05). In the levosulpiride-exposed group, no cases of stillbirth. Major malformations at birth were not significant ↑ in the exposed group compared with ctrl (2.7% *vs.* 4.4%, *p* = 0.481)	Levosulpiride causes no significant adverse effects on pregnancy outcomes. Thus, it may not be a major teratogen in ♀ with nonpsychiatric conditions. However, the results of this study cannot be generalized to ♀ receiving levosulpiride for psychiatric conditions like bipolar disorder, schizophrenia and other psychoses.
Onken *et al*., 2018 [38]	Berlin, Germany (1, German Embryotox)	17 paliperidone-exposed pregnant ♀ (*x̄* age = 32.5 ± 7.3, range 18-41 yrs); 8 ♀ exposed during the 1^st^ trimester only, 7 ♀ during the entire pregnancy, and 2 ♀ during the 2^nd^ and/or 3^rd^ trimesters. 14 ♀ had psychotic disorders, 2 ♀ had mood disorders, and 1 ♀ unknown	Longitudinal, both retrospective and prospective (eventually, only prospectively collected data from January 2007 to June 2016 were analyzed). Data was collected by calling the participant 8 weeks after expected delivery or through mailed questionnaires reflecting the telephone structured interview (response rate 75%). Mother and child outcomes of ♀ exposure to paliperidone	14 ♀ with live-born children, 43% were > 35-yrs-old, 29% > 40-yrs-old. Fourteen pregnancies resulted in 15 live-born children (1 twin pair) without major malformations; minor issues: 3 children were for GA, 5 premature, possibly associated with smoking throughout pregnancy (11 pts smoked and 3 used alcohol during pregnancy). 7 pts (41.2%) took oral paliperidone 3-6 mg/day, and 10 (58.8%) took 50-150 mg/ 3-4 weeks LAI paliperidone i.m.. 4 pregnancies with complications: 1 ♀ HELLP syndrome at GW 31 (twin pregnancy), one child suffered IUGR and oligohydramnios, both cases ended in premature cesarean sections. 1 further child was delivered by cesarean section in GW 38 after oligohydramnios. In another pregnancy, abnormal uteroplacental blood flow and IUGR at GW 28 resulted in a small GA child born in GW 41. Child Welfare Services was involved in 5 cases. 1 ♀ overdosed due to psychosis (150 mg paliperidone + 4,000 mg amisulpride) at GW 32 and experienced QT prolongation; however, her child was born at GW 37 without major issues. Among the 17 ♀ enrolled, 6 were exposed to PAL throughout their entire pregnancies. There were 3 pregnancy losses (2 spontaneous abortions and 1 ETOPP). 4 pts stopped paliperidone within the 1^st^ trimester, and 2 started it after GW 14; 8 pts had no concurrent medication, 6 had nonteratogenic medications, and 3 had concomitant valproate (all ensuing in live births)	No child was born with a major congenital malformation. 1 premature child (born at GW 34) had congenital cryptorchidism and umbilical hernia, but these are common abnormalities in preterm infants. No evidence to suggest teratogenicity associated with paliperidone during pregnancy. However, there were no ctrl groups. The only comparisons were with the German general population.
Galbally *et al*., 2018 [39]	Murdoch-Subiaco, Western Australia-Heidelberg, Victoria, Australia (2)	26 pregnant (*x̄* age = 28.92 ± 5.84) treated with aripiprazole for SMI. 14 ♀ ceased aripiprazole after the 1^st^ trimester, *x̄* dose 17.98 mg ± 12.00); 12 ♀ continued aripiprazole during pregnancy *x̄* dose 19.77 mg ± 15.99)	Retrospective, multicentric study investigating the reproductive safety of aripiprazole	No differences between ♀ exposed to aripiprazole and the Australian population for GDM; GA at delivery and BW ↓ for ♀ using aripiprazole (37.3 wks and 3094 g) compared to the Australian population (38.7 wks and 3342 g);♀ who ceased aripiprazole during pregnancy had infants ↓ BW percentiles by GA (IQR: 9-72.5) compared with infants of ♀using aripiprazole during pregnancy (IQR: 33-90); the difference was not significant (Mann-Whitney U = 106.50, Fisher's exact *p* = .107); ♀ exposed to aripiprazole during pregnancy reported pregnancy hypertension (15.4%) compared to the Australian population (3.7%; Fisher's Exact *p* = .015); admission to SCN/NICU was ↑in our sample compared to the Australian rate (16%; Fisher's Exact *p* = .003); trend was ↓for ♀ not using aripiprazole during pregnancy	The use of aripiprazole in pregnancy was associated with an increased risk of ↓ BW, hypertension in pregnancy, shorter gestation, ↑ and rates of admission of the neonate than the expected Australian population; however, the small sample size underpowered the study.
Cohen *et al*., 2018 [40]	Boston, Massachusetts, USA (1, NPRAA)	357 ♀ aged 18-45 yrs; 152 1^st^-trimester exposure to quetiapine (*x̄* age = 32.3 ± 4.89) 205 unexposed to SGAs (*x̄* age = 33.2 ± 4.2)	Registry examination of records of women with psychiatric illness exposed to quetiapine and unexposed to SGAs; Outcome: risk of major malformations	The prevalence of major malformations in infants with first-trimester exposure to quetiapine (N = 155) was 1.29% (n = 3; 95%CI from 0.16 to 4.58); among infants unexposed to SGAs (N = 210), 1.43% (n = 3; 95% CI from 0.30 to 4.12). Unadjusted OR of major malformations quetiapine-exposed *vs.* SGA-unexposed = 0.90 (95% CI from 0.15 to 5.46; *p* = 0.91). Maternal BMI moved the OR downward (OR = 0.77, 95% CI from 0.12 to 4.88), while concomitant use of anticonvulsants moved it upward (OR = 1.36, 95% CI from 0.22 to 8.29). 1^st^-trimester nicotine use and diagnosis of depression also moved the OR upward but marginally	Mothers exposed to quetiapine were younger, more chronic and with a ↑ BMI at BL; furthermore, there were more primary diagnoses of bipolar disorder in the quetiapine-exposed groups and more diagnoses of anxiety and depressive disorders in the SGA-unexposed group. However, the authors addressed potential confounders. Overall, quetiapine carries a low potential for being related to major malformations.
Anderson *et al*., 2020 [41]	Atlanta, Georgia-Jacksonville, Florida, USA-Vancouver, British Columbia, Canada (10, NBDPS)	The total sample of mothers participating in NBDPS was 31,651 ♀ case-11,615 ♀ control pairs. Among them, 22,387 cases/11,470 ctrl were included; only 36 ♀ cases and 12 ♀ ctrl were exposed to atypical APs during early pregnancy; APs analyzed: quetiapine (case: 52.2%; control: 52.9%) aripiprazole (case: 23.9%; ctrl: 23.5%) olanzapine (case: 11.9%; ctrl: 17.6%), and risperidone (case: 13.4%; ctrl: 11.8%)	Longitudinal. A cohort-controlled survey exploring associations between atypical AP use in early pregnancy and specific birth defects	Elevated associations (cOR ≥ 2.0) between maternal AP use during early pregnancy and conotruncal defects (6 cases; cOR: 2.3, 95% CI from 0.9 to 6.1), tetralogy of Fallot (3 cases; cOR: 2.5, 95% CI from 0.7 to 8.8); associations between atypical APs use in early pregnancy and cleft palate (4 exposed cases; cOR: 2.5, 95% CI from 0.8 to 7.6), anorectal atresia/stenosis (3 cases; cOR: 2.8, 95% CI from 0.8 to 9.9), and gastroschisis (3 cases; cOR: 2.1, 95% CI from 0.6 to 7.3)	Small exposed case counts limited the identification of associations between individual AP use and specific birth defects.
Ellfolk *et al*., 2020 [42]	Helsinki-Turku, Finland (1, Finnish National Registries)	1,181,090 ♀ with a singleton pregnancy ending in delivery (1996-2016); ♀ divided into 3 groups: exposed to SGAs during pregnancy (n = 4,225), exposed to FGAs during pregnancy (n = 1,576), and unexposed (n = 21,125)	A population-based birth cohort study using national register data extracted from the Drugs and Pregnancy database in Finland, years 1996-2016, exploring associations between SGA use during pregnancy and ↑ risk of pregnancy and neonatal complications	Mothers exposed to SGAs: ↑ risk for GDM (adjusted OR 1.43; 95% CI from 1.25 to 1.65), cesarean section (OR 1.35; 95%CI from 1.18 to 1.53), being born LGA (OR 1.57; 95% CI from 1.14 to 2.16), and preterm birth (OR 1.29; 95%CI from 1.03 to 1.62) compared to unexposed. SGA-exposed infants ↑ neonatal complications; SGA-exposed mothers, ↑ risk of cesarean section (OR 1.25, 95% CI from 1.03 to 1.51) and LGA (OR 1.89, 95% CI from 1.20 to 2.99) compared to FGA mothers. No differences between SGAs and FGAs for neonatal complications	Prenatal exposure to SGAs is associated with ↑ a risk of pregnancy complications related to impaired glucose metabolism. Neonatal problems are common and occur similarly in SGA and FGA groups. Limitation: sample characteristics do not allow to confirm drug compliance
Ellfolk *et al*., 2021 [43]	Helsinki-Turku, Finland (1, Finnish Drugs and Pregnancy database)	1,273,987 pregnant, included singleton pregnancies ending in live or stillbirth or termination of pregnancy due to severe malformation (1996-2017); excluded pregnancies with exposure to known teratogens; ♀ divided into three groups: exposed to SGAs (quetiapine *n* = 2,618, olanzapine *n* = 413, risperidone *n* = 242, aripiprazole *n* = 220, and clozapine *n* = 106) (*n* = 3,478), exposed to FGAs (*n* = 1,030), and unexposed (no purchases of SGAs or FGAs during pregnancy, *n* = 22,540)	Longitudinal, retrospective, population-based birth cohort study using national register data extracted from the Drugs and Pregnancy database in Finland, years 1996-2017, exploring associations between SGA use during 1^st^ trimester of pregnancy and ↑ risk of MCM	SGA mothers ↑ are overweight than FGA or unexposed; SGA and FGA mothers ↑ tobacco smoke than unexposed. Pregestational and GDM ↑ in SGA mothers compared to FGA and unexposed. Of the individual SGAs, olanzapine ↑ risk of MCMs (odds ratio, OR 2.12; 95% CI from 1.19 to 3.76); olanzapine associated with ↑ risk of musculoskeletal malformations compared to unexposed (OR 3.71; 95% CI from 1.35 to 10.1); use of SGAs not associated with ↑ risk of organ-specific malformations compared to unexposed or to the FGA group	Olanzapine is associated with ↑ the risk of MCM and, specifically, musculoskeletal malformations; the musculoskeletal malformations recorded among olanzapine-exposed infants/fetuses were, and no specific malformation pattern was observed.
Freeman *et al*., 2021 [44]	Boston, Massachusetts-Cleveland, Ohio, USA-Toronto, Ontario, Canada (1, NPRAA)	1,906 pregnant ♀ (age range 18-45 yrs) with psychiatric diagnosis prospectively enrolled (n = 889 exposed to SGA during the 1^st^ trimester; n = 1,017 unexposed to SGAs). 1,311 ♀ completed the study: 621 pregnant ♀ had evaluable data and 1^st^ trimester SGA exposure (*x̄* age = 32.6 ± 5.14). Of the eligible 621 exposed ♀, 158 had 1^st^ trimester exposure to aripiprazole (*x̄ age =* 32.4 ± 5.49) *vs.* 690 pregnant ♀ with psychiatric diagnosis but no SGA exposure (*x̄ age =* 32.7 ± 4.19)	Longitudinal, prospective cohort study investigating the risk of major malformation in infants exposed during the 1^st^ trimester of pregnancy to aripiprazole compared to infants whose mothers had a psychiatric diagnosis but were not treated with atypical antipsychotic during pregnancy	Most women used aripiprazole during all three trimesters of pregnancy (N = 137, 86.7%). The prevalence of major malformation among the 1^st^ trimester exposed group [*n* infants = 163, including three sets of twins and one set of triplets; *n* major malformations = 7] was 4.29% (unadjusted OR: 2.21; 95% CI from 0.88 to 5.57; adjusted OR for potential confounders: 1.35; 95% CI from 0.43 to 4.20, ns); among ctrl [*n* infants = 704, including 14 sets of twins, *n* major malformations = 14] prevalence was 1.99%	Aripiprazole exposure in 1^st^ trimester of pregnancy does not substantially ↑ increase the risk of major malformations. Data are consistent with previously reported ones not showing a strong association between fetal exposure to SGAs and ↑ the rate of major malformations. Limitation: a relatively small number of participants were exposed to aripiprazole; however, this sample size is the largest in the literature referring to aripiprazole
Viguera *et al*., 2021 [45]	Boston, Massachusetts, USA (1, NPRAA)	1,906 pregnant ♀ with a psychiatric diagnosis prospectively enrolled (n = 889 exposed to SGA during 1^st^ trimester; n = 1,017 unexposed to SGAs). Among them, 1,311 ♀ completed the study: 621 ♀ with 1^st^ trimester SGA exposure (*x̄* age = 32.6 ± 5.14) and 690 ♀ with no SGA exposure (*x̄* age = 32.7 ± 4.19)	Longitudinal, prospective cohort study exploring infant risks related to SGA exposure during the 1^st^ trimester of pregnancy. Telephone interviews of participants at 3 time points: at enrollment, at 7 months of pregnancy and at 3 months post-partum). These three interviews assessed demographic characteristics, medication use and dosage changes, social habits, and clinical history. Neonatal outcome data were obtained through a systematic review of obstetrics, labor and delivery, and pediatric medical records.	Prevalence of major malformation in the 1^st^ trimester SGA exposed group [n infants = 640, including 17 twin pregnancies and 1 triplet pregnancy] was 2.50%, (n = 16, adjusted OR: 1.483; 95% CI from 0.625 to 3.517)]; among the unexposed group [n infants = 740, including 14 twin pregnancies) prevalence was 1.99% (n = 14)	SGA exposure in the 1^st^ trimester of pregnancy does not substantially ↑ increase the risk of major malformations. In order of frequency, APs used in the exposed group were quetiapine, aripiprazole and lurasidone. These results are consistent with CDC’s national rate of major malformations in the general population; however, the absolute risk in the unexposed group was lower than expected. The authors observed that some confounders, such as cigarette use, attenuated the odds ratio, while others, such as diagnosis of anxiety or depression and SSRI use, increased the unadjusted odds.
Nguyen *et al*., 2022 [46]	Rockingham, Western Australia, Australia (1)	36 ♀ in treatment with LAI APs during pregnancy. Included 38 pregnancies (*x̄* first antenatal exposure = 20.4 GWs), 35 of whom with a confirmed exposure during the 1° trimester. Regarding the specific LAI preparations used, 24 pregnancies were exposed to FGA LAIs (zuclopenthixol n = 12, flupenthixol n = 9, haloperidol n = 2, fluphenazine n = 1) and 14 to SGA LAIs (aripiprazole n = 8, risperidone n = 4, paliperidone n = 2)	A longitudinal, retrospective study investigating pregnancy, neonatal health, and psychosocial outcomes among pregnant women receiving LAI APs at a women’s and newborn hospital in Western Australia from 1999 to 2017. The current is an integral part of a broader study involving treatment and historical comparison of pregnant women diagnosed with schizophrenia over a span of 20 yr at King Edward Memorial Hospital (“Apps 20”)	5.7% of 1^st^-trimester exposures resulted in minor congenital malformations managed conservatively. Of 38 pregnancies, 36.8% underwent oGTT screening, revealing 50% GDM cases and 5.3% preexisting type 2 diabetes. *x̄ BW =* 3.18 kg ± 0.76, with GA = 38.25 weeks ± 2.19). Psychiatric admissions during pregnancy were 31.5%, while child protection involvement reached 71.1%. No major outcome differences between SGAs and FGAs, except for lower post-delivery contraception rates in the SGA group. Aboriginal and/or Torres Strait Islander Australian women (44.7%) had fewer partners (41.1% *vs.* 85.7%, *p* = 0.004), ↑ smoking rates (88.2% *vs.* 52.4%, *p* = 0.020), and ↑ psychiatric admissions (58.8% *vs.* 19.0%, *p* = 0.014). Their antenatal care attendance was ↓ (61.6% *vs.* 86.2%, *p* = 0.004), and child protection involvement ↑ (88.2% *vs.* 57.1%, *p* = 0.038)	Pregnant women treated with LAI APs exhibit ↑ rates of pregnancy/neonatal complications compared to the general population, with no differences between exposure to FGAs or SGAs. 5.7% of exposed pregnancies produced congenital malformations, compared to the 3-5% malformation rate in the general population. The prevalence of GDM was ↑ higher than in the general population; focusing on ♀ undergoing oGTTs, this rate further escalated. The likelihood of encountering psychosocial adversities was ↑. This resulted in ↑ a risk of psychiatric relapses and child protection involvement.
Yakuwa *et al*., 2022 [47]	Tokyo, Japan (1, JDIIP)	404 pregnant ♀ consulting JDIIP October 2005-December 2016 and exposed to SGAs (*x̄* age = 33 yrs, range 29-36 yrs), yielding 351 live births, 3 stillbirths, 34 spontaneous abortions, and 16 ETOP *vs.* 4,328 pregnant ♀ unexposed to SGAs (ctrl, *x̄* age = 32 yrs, range 29-35 yrs) yielding 3,899 live births, 18 stillbirths, 313 spontaneous abortions, and 98 ETOP (*p* = 073, ns)	Participants were asked to complete a questionnaire (with items on pregnancy outcome, date of delivery, GA at delivery, infant malformations, and height, weight, head circumference, and chest circumference at birth) 1 month after the expected delivery. Compared were ORs for major congenital malformations in 1^st^-trimester SGA-exposed children and ORs of children unexposed to SGAs and to teratogens. Outcomes: primary, live birth rate and major congenital malformations; secondary, ETOP rate	↓ response rates to the questionnaire in the SGA group (77.7%) *vs.* unexposed (84.2%), despite similar initial consent rates (87.7% SGA *vs.* 89.7% unexposed). Live birth rates were 86.9% SGA *vs.* 90.1% unexposed, *p* = 0.235, ns. ETOP ↑ in SGA (4% *vs.* 2.3% unexposed, OR SGA *vs.* unexposed = 1.61, 95% CI from 1.04 to 3.05, *p* = 0.036; *p* = 0.090 when adjusting for alcohol and nicotine use). SGA-exposed pregnant ♀ had ↑ BMI compared to unexposed (21.4 SGA *vs.* 20.0 unexposed, *p* < 0.001), smoked less than unexposed (73.8% SGA *vs.* 86.1% unexposed, *p* < 0.001; however, after knowing pregnancy status, 10.4% SGA-exposed ♀ continued smoking during pregnancy *vs.* 3.7% of unexposed), less unplanned pregnancies (n = 209 [51.7%] *vs.* 2,743 [63.4%], *p* < 0.001), ↑ diabetes mellitus than unexposed (3% SGA *vs.* 1.1% unexposed, *p* = 0.005) and ↑ psychiatric disorder rates (n = 396 [98%] *vs.* n = 1,176 [27.2%], *p* < 0.001; specifically: depression n = 131 [32.4%] *vs.* n = 454 [10.5%], schizophrenia n = 126 [31.2%] *vs.* n = 9 [0.2%], bipolar disorder n = 38 [9.4%] *vs.* n = 26 [0.6%]) than unexposed. There were 3 malformations in the SGA-exposed group (absolute risk 0.9) *vs.* 70 (absolute risk 1.8) in the unexposed group (*p* = 0.28, ns). Malformations were right hydronephrosis (quetiapine exposure in bipolar disorder pt who also took valproate and benzodiazepine and nonbenzodiazepine GABA_A_ agonists), spina bifida and hydrocephalus (quetiapine exposure in bipolar disorder pt who also took valproate and sertraline), and multiple heart, kidney, head and ear malformations (perospirone exposure in schizophrenia pt who also took trazodone). The SGA group was exposed to aripiprazole (n = 147), quetiapine (n = 91), olanzapine (n = 83), risperidone (n = 71), perospirone (n = 32), blonanserin (n = 24), paliperidone (n = 71), and >1 SGA (n = 45)	Despite lacking a JDIIP-non-contacting ctrl group that could have behaved differently and the fact that not all confounders were controlled for, the results indicate no malformation potential for SGAs compared to lack of exposure in pregnant ♀ consulting JDIIP.
Hálfdánarson *et al*., 2022 [48]	Reykjavik, Iceland-Oslo-Bergen, Norway-Stockholm, Sweden-Turku-Helsinki, Finland-Aarhus, Denmark (5, Medical Birth Registers	Four million three hundred twenty-four thousand eighty-six eligible children were included in the study cohort. Overall, 15,466 children (0.4%) were exposed to APs *in utero*, of whom 6,478 (41.9%) in the 1^st^ trimester only, 3,349 (21.7%) in the 2^nd^/3^rd^ trimesters only, and 5,639 (36.4%) throughout the entire pregnancy	Population-based cohort study on nationwide population data and health registers from five Nordic countries. The study population included all live-born singletons in the Medical Birth Registers of Denmark 1997-2017	15,466 (0.4%) of 4,324,086 singleton births were exposed to APs *in utero*. During a 10-year median f-u, 72,257 children with ADHD and 38,674 children with ASD were identified. Unadjusted HRs were ↑ for both outcomes but became not significant after adjustment: 1.10 (95% CI from 1.00 to 1.27) for ADHD and 1.12 (95% CI from 0.97 to 1.29) for ASD. Adjusted HRs remained consistent by trimester of exposure and AP type.	Findings suggest little or no increased risk of ADHD or ASD with exposure to APs *in utero*. The ↑ risk observed among children whose mothers had unspecified conditions is probably attributable to residual confounding.
-	Denmark 1997-2017, Finland 1996-2016, Iceland 2004-2017, Norway 2004-2017, and Sweden 2006-2016)	-	Finland 1996-2016, Iceland 2004-2017, Norway 2004-2017 and Sweden 2006-2016. Outcome: HRs for ASD and/or ADHD	Comparing *in-utero* exposure with pre-pregnancy use yielded HRs of 0.74 (95%CI from 0.62 to 0.87) for ADHD and 0.88 (95%CI from 0.70 to 1.10) for ASD. Siblings’ analyses yielded HRs of 1.14 (95%CI from 0.79 to 1.64) for ADHD and 1.34 (95%CI from 0.75 to 2.39) for ASD. Psychotic or bipolar disorder in the mother ↑ risk of ADHD or ASD in the child	Strong association between neurodevelopmental outcomes and maternal psychotic and bipolar disorders
Straub *et al*., 2022 [49]	Boston-Lexington, Massachusetts-Stanford, California-Chicago, Illinois-Pittsburgh, Pennsylvania-Providence, Rhode Island, USA (1, Medicaid; 2 cohorts: MAX and MarketScan)	MAX cohort: 2,034,883 ♀ with pregnancies unexposed to APs and 9,551 pregnancies with ≥1 AP prescribed, *x̄* age = 26.8 ± 6.1 yrs; 5356 (56.1%) identified themselves as White, 2,720 (28.5%) as Black, 500 (5.2%) as Hispanic/Latino, and 204 (2.1%) as Asian/Pacific Islander. MarketScan cohort: 1,306,408 ♀ unexposed to APs and 1,221 ♀ with ≥ 1 AP prescribed, *x̄* age = 33.1 ± 5.0 yrs; ethnicity not available	Birth cohort study using data from the Medicaid Analytic extract (MAX, 2000-2014). Large scale epidemiologic study nested in nationwide healthcare utilization databases, including broad information for controlling for confounders by treatment indication and associated factors. Outcome: to evaluate the association between *in-utero* exposure to AP drugs and NDDs in children	Most exposures were to atypical APs (≈90% in both cohorts and assessment periods), with quetiapine being the most commonly dispensed (≈40% of all exposures), → by aripiprazole (16%-23%). Most ♀ received only 1 type of AP during pregnancy. ♀ from the MAX cohort receiving any AP during pregnancy were more likely to be older (≥35 yrs) and White, to have psychiatric or neurologic conditions and other comorbidities, be prescribed other drugs during pregnancy, and have unhealthy lifestyle behavior like alcohol, nicotine, and other substance use disorders. SES data did not point to differences between AP-exposed and unexposed women. All risks found to be ↑ in most exposure-outcome contrasts were no longer significant after adjustments, with the possible exception of aripiprazole (RR 1.36, 95%CI from 1.14 to 1.63). Results consistent across sensitivity analyses	↑ risk of NDD seen in children born to women taking APs late in pregnancy is likely to be due to maternal characteristics and not to be causally related to prenatal AP exposure. Aripiprazole data need replication before accepting causality. The observed 2-fold ↑ NDD risk is more likely to be related to the mother’s mental illness than to *in utero* AP exposure; this underlies the need for closely monitoring the development course of children of ♀ with mental disorders
Huybrechts *et al*., 2023 [50]	Boston, Massachusetts, USA-Solna-Stockholm, Sweden-Oslo, Norway-Aarhus, Denmark-Reykjavik, Iceland-Turku-Helsinki, Finland (6, InPress)	The Nordic cohort included all pregnancies resulting in singleton live-born infants (4,531,396 pregnancies). The US cohort consisted of publicly insured mothers linked to their live-born infants (2,074,159 pregnancies). This cohort included 6,455,324 unexposed pregnancies (maternal age range across countries, 24-31 yrs), 21,751 pregnancies with atypical AP exposure (age range, 26-31 yrs), and 6,371 with typical AP exposure (age range, 27-32 yrs). Atypical APs were quetiapine (n = 11,065), aripiprazole (n = 4,523), and olanzapine (n = 3,110); typical APs were chlorpromazine (n = 1,348), dixyrazine (n = 1,086, Nordic countries only), and perphenazine (n = 1,054)	Retrospective cohort analysis of nationwide health registers (Denmark: 1997-2017; Finland: 1996-2016; Iceland: 2004-2017; Norway: 2005-2018; Sweden: 2006-2016) and of nationwide Medicaid Analytic eXtract (2000-2014) for the US. Outcome: Any MCM	The risk of having an infant diagnosed with any MCM was 2.7% (2.7-2.8%) (pooled absolute risk) among all unexposed women and 3.7% (95% CI, from 3.6% to 3.7%) when restricted to unexposed women with a mental health condition. The risk of any MCM was 4.3% (95% CI, from 4.1% to 4.6%) among atypical APs exposed and 3.1% (95% CI, from 2.7% to 3.5%) among typical APs exposed, with risks ranging from 1.8% (95% CI, from 1.1% to 2.5%) to 5.5% (95% CI, from 3.8% to 7.1%), depending on the specific AP drug. Risk estimates for olanzapine and oral clefts ranged from an adjusted RR of 1.1 (95% CI, 0.4-2.8), when restricted to monotherapy to 2.6 (95% CI, 1.2-5.4) when a diagnosis of a mental disorder was required for both exposed and unexposed groups. ↑ Risk of cleft palate with olanzapine, ↑ risk of gastroschisis, and other specific brain anomalies with atypical APs, and ↑ risk of cardiac malformations with chlorprothixene	*In utero,* AP exposure is not significantly associated with ↑ the risk of malformations. The observed ↑ risks of oral clefts associated with olanzapine, gastroschisis and other specific brain anomalies with atypical APs in general and of cardiac malformations with chlorprothixene need to be confirmed.
Cohen *et al*., 2023 [51]	Boston, Massachusetts, USA (1, NPRAA)	264 pregnant ♀ with 1st-trimester exposure to quetiapine (*x̄* age = 32.0 ± 4.80), 134 to lurasidone (*x̄* age = 33.2 ± 4.92), and 886 with no SGA exposure (ctrl, *x̄* age = 32.6 ± 4.18), with evaluable data, aged 18-45 yrs, with lifetime psychiatric history; f-u during pregnancy and postpartum to determine risk of major malformations in infants with 1^st^ trimester in utero exposure to SGAs	Longitudinal, prospective cohort study to explore 1^st^ trimester SGA exposure-related infant risks. Telephone interviews of ♀ at enrollment (any time during pregnancy), at 7 months of pregnancy and at 3 months post-partum). Interviews assessed demographic characteristics, medication use and dose changes, social habits, and clinical history. Neonatal outcome data was obtained through a systematic review of obstetrics, labor and delivery, and pediatric medical records. Malformations diagnosed by a dysmorphologist blind to study procedures.	Malformations in 5 of 273 1st trimester quetiapine-exposed infants (1.83% absolute risk; 95%CI from 0.60 to 4.22; OR 1.04 (95%CI from 0.38 to 2.85); in 3 of 137 1st trimester lurasidone-exposed infants (2.19% absolute risk; 95%CI from 0.45 to 6.27; OR 1.24 (95%CI from 0.36 to 4.32), and in 16 of 904 infants unexposed to SGAs (1.77% absolute risk; 95%CI from 1.01 to 2.86). After adjusting for the mother’s age, the absolute risk of lurasidone dropped to ×1.25 the odds of a malformation with unexposed infants (95%CI from 0.36 to 4.36). Malformations were with quetiapine: Pulmonary stenosis and dysplastic pulmonary valve (mother also took duloxetine, L-thyroxine, metformin and valaciclovir), Dandy-Walker malformation (pt also took venlafaxine and oxcarbazepine), right hydronephrosis, bilateral flat helices, overlapping toes on left foot, and shallow sacral dimple,	Data are preliminary due to small sizes; however, they indicate low teratogenic potential for both quetiapine and lurasidone. The healthy lifestyle adopted by ♀ endorsing the NPRAA may have kept absolute risks and unadjusted ORs low. One of the strengths of this study consists in its prospective design, which minimizes recall and selection biases.
-	-	-	Primary outcome: unadjusted ORs of major malformation with lurasidone- and quetiapine-exposure and infants unexposed to SGAs; confounders introduced in logistic regression one-by-one	cystic hygroma and alobar holoprosencephaly (but other also took lamotrigine and sertraline), and major arteries transposition (but the mother also took aripiprazole, bupropion and labetalol). With lurasidone: surgically-repaired hypospadias (but the mother also took zolpidem and chlorpromazine), Anencephaly (but the mother also took Li+, venlafaxine, nortriptyline, trazodone, and lorazepam), and lobar holoprosencephaly (but the mother also took nefazodone)	-
Viguera *et al*., 2023 [52]	Boston, Massachusetts, USA (1, NPRAA)	2,619 pregnant ♀ with a psychiatric diagnosis prospectively enrolled in the study (n = 1,010 exposed to SGAs during 1^st^ trimester; n = 1,609 unexposed to SGAs). Among them, 47 ♀ with 1^st^ trimester exposure to olanzapine (*x̄* age = 33.6 ± 4.56) and 1,1134 ♀ with no SGA exposure (*x̄* age = 32.5 ± 4.08) were enrolled	Longitudinal, prospective, cohort study exploring associations between olanzapine use during 1^st^ trimester of pregnancy and ↑ risk of major malformation	No major malformations occurred in the group with 1^st^-trimester exposure to olanzapine [n evaluable infants = 49, including 2 sets of twins] (Absolute Risk 0.00%; 95% CI, 0-7.25); n = 19 major malformation in the unexposed group [n evaluable infants = 1,134, including 22 sets of twins] (Absolute Risk: 1.64%; 95%CI from 0.99 to 2.55)	These preliminary data suggest that olanzapine exposure during the 1^st^ trimester is not associated with major malformations. However, the small sample size underpowered the study; furthermore, there were wide 95%CI variations around the potential malformation risk.
Schrijver *et al*., 2023 [53]	Rotterdam-Leiden-Amsterdam, The Netherlands (3)	Ninety-one children were born from mothers with SMI in 63 distinct families, comprising 22 families with 2 children each, 3 families with 3 children each, and 2 twin pairs. Children of mothers with schizophrenia or schizoaffective disorder aged 6 to 14 years exposed to ≥1 AP during pregnancy compared to 9 non-exposed children of mothers with postpartum psychiatric diagnoses. Exposure to other psychotropic drugs is not excluded	The study was nested within a cohort investigation focusing on neurodevelopmental functioning in children of mothers with SMI. This analysis explored the impact of *in-utero* exposure to Li^+^ or AP. Patients treated during pregnancy were recruited from Dutch perinatal psychiatry centers in Amsterdam.	The non-exposed group had more children exposed to Li^+^ (65% *vs.* 35%); the exposed group had ↑ co-treatment with ADs (65% *vs.* 19%). Parents' educational level was ↑ in the non-exposed group (63.5% *vs*. 17.6%). Linear regression analysis found no association between prenatal AP exposure and child IQ. No associations were observed for the NEPSY subtests. No statistically significant association with learning problems (OR 0.26, 95%CI from 0.03 to 2.11; *p* = 0.21) or psychiatric diagnoses (OR 0.78, 95%CI from 0.20 to 3.04; *p* = 0.72) in the exposed group	The first study explored cognitive and psychiatric functioning in school-aged children exposed to typical or atypical antipsychotics during gestation. The investigation revealed no adverse effects of AP exposure on neurodevelopmental outcomes, including IQ and neurocognitive domains. No association between AP exposure and the occurrence of learning problems or psychiatric disorders in children
Liu *et al*., 2023 [54]	Aarhus, Denmark (1, Danish National Registries)	503,158 singleton pregnancies with a fetus alive at the nuchal scan from GW 11, data on malformations 1 year after delivery and no genetic abnormalities	Population-based cohort study utilizing Danish national registers, adhering to the (RECORD-PE) guidelines from January 1, 2008, to December 31, 2017. APs use in the 1^st^ trimester was defined as receiving treatment from 30 days before pregnancy to 12 GWs. Data on AP use retrieved from the National Prescription Registry; prenatally diagnosed congenital malformations from the Fetal Medicine Database.	The prevalence of AP exposure during the first trimester was 0.2%, primarily to SGAs (69.3%) with quetiapine monotherapy as the most commonly prescribed AP; characteristics between exposed and unexposed pregnancies were well balanced. The prevalence of congenital malformations was 7.3% in AP-exposed pregnancies, 5.1% in unexposed, and 6.0% in discontinuers. The adjusted PR for major malformations was 1.23 (95% CI, 1.01-1.50) for AP exposure compared to unexposed pregnancies and 1.14 (95% CI, 0.88-1.48) compared to discontinuers. For cardiac malformations, the PRs were 1.18 (95% CI, 0.77-1.80) compared to unexposed pregnancies and 1.10 (95% CI, 0.65-1.52) compared to discontinuers	1^st^ trimester AP exposure was associated with ↑ the prevalence of major and cardiac malformations compared to unexposed pregnancies. However, these associations were influenced by confounders and were attenuated after propensity score adjustment; the risks moved towards null when compared to discontinuers and sibling analyses. These results suggest a minimal or negligible increased risk of major malformations due to AP use.

**Note:** Abbreviations used: AD(s), antidepressant(s) ; ADHD, attention deficit/hyperactivity disorder; APs, antipsychotics; ASD, autism spectrum disorders; A-V, atrial and/or ventricular; BL, baseline; BMI, body mass index; BW, birth weight; CDC, Centers for Disease Control (and Prevention); CI, confidence interval; cOR, crude odds ratio; CPRD, Clinical Practice Research Datalink; ctrl, control(s); ETOP, elective termination of pregnancy for any reason (patient’s or physician’s decision); ETOPP, elective termination of pregnancy for personal reasons; FGA(s), first-generation antipsychotic(s); FGR, fetal growth restriction; f-u, follow-up; GA, gestational age; GDM, Gestational Diabetes Mellitus; GI, gastrointestinal; GW(s), gestational week(s); HC(s), healthy control(s); HELLP, Hemolysis Elevated Liver enzymes Low Platelet count syndrome; HR, hazard ratio; InPress, International Pregnancy Safety Study Consortium; INSEE, Institut national de la statistique et des études économiques; IQ, Intelligence quotient; IQR, interquartile range; IUGR, IntraUterine Growth Retardation; JDIIP, Japan Drug Information Institute in Pregnancy; LAI, Long-acting Injectable; LGA, large for gestational age; Li^+^, lithium; MAX, Medicaid Analytic eXtract (MAX, 2000-2014); NBDPS, National Birth Defects Prevention Study; MCM(s), major congenital malformation(s); NDD(s), neurodevelopmental disorder(s); NEPSY, Developmental Neuropscyhological Assessment; NICU(s), neonatal intensive care unit(s); NINDS, National Institute of Neurological Diseases and Stroke; NPRAA, National Pregnancy Registry for Atypical Antipsychotics; ns, not (statistically) significant; oGTT, oral glucose tolerance test; OR, odds ratio; *p*, statistical probability; PR(s), prevalence ratio(s); PRR, proportional reporting ratio; pt(s), patient(s); RECORD-PE, RECORD Statement for Pharmacoepidemiology; RR, relative risk; SCA, severe congenital abnormalities; SDR(s), Signals of disproportionate reporting, *i.e*., PRR ≥2, χ2 ≥4, and cases n≥3; SES, socioeconomic status; SGA(s), second-generation antipsychotic(s); SMI, severe mental illness; THIN, The Health Improvement Network; TIS, Centrede Ré férencesurles Agents Té ratogènes; WHO, World Health Organization; x̄, mean; yr, years; χ2, Chi square; ×, for, per; ≈, about equal, not different; ♀, female, woman; ♂, male, man; ↓, decreased, lower; ↑, increased, higher, →, induced, followed.

## LIST OF ABBREVIATIONS

ADHD: Attention-deficit/hyperactivity Disorder
AP(s): Antipsychotic(s)
FGA(s): First-generation Antipsychotic(s)
LAI(s): Long-acting Injectable Formulation(s)
NPRAA: National Pregnancy Registry for Atypical Antipsychotics
PRISMA: Preferred Reporting Items for Systematic Reviews and Meta-Analyses statement
RoB: Risk-of-bias
ROBINS-E: Risk Of Bias In Non-randomized Studies of Exposure
SGA(s): Second-generation Antipsychotic(s)

## STANDARDS OF REPORTING

PRISMA guidelines and methodology were followed.

## SUPPLEMENTARY MATERIAL

Supplementary material is available on the publisher’s website along with the published article. The following supporting information can be downloaded at www.mdpi.com/xxx/s1, Table S1: List of records resulting from our search and Inclusion *vs*. Exclusion with reason; Table S2: Risk-of-Bias of included studies according to the Cochrane RoB tool 2.0; Table S3: PRISMA Checklist.
