# Supplementary material for: Is Antipsychotic Drug Use During Pregnancy Associated with Increased Malformation Rates and Worsening of Maternal and Infant Outcomes? A Systematic Review
Source: Curr Neuropharmacol. 2024 May 17;22(14):2402–21. doi: 10.2174/1570159X22666240516151449 (PMC11451318; doi:10.2174/1570159X22666240516151449)
Supplement: Supplementary file 1 [file CN-22-2402_SD1.pdf]

Supplementary Material

Is Antipsychotic Drug Use During Pregnancy Associated with Increased Malformation Rates and Worsening of Maternal and Infant Outcomes? A Systematic Review

Gabriele Sani[1,2], Tommaso Callovini[3], Ottavia Marianna Ferrara[2], Daniele Segatori[2], Stella Margoni[2], Alessio Simonetti[1,2], Francesco Maria Lisci[2], Giuseppe Marano[1,2], Alessia Fischetti[2], Georgios D. Kotzalidis[1,4,*], Federica Di Segni[5], Federica Fiaschè[6], Delfina Janiri[1,2], Lorenzo Moccia[1,2], Giovanni Manfredi[7], Alessandro Alcibiade[5,8], Caterina Brisi[2], Flavia Grisoni[2], Gianmarco Stella[2], Evelina Bernardi[2], Andrea Brugnami[2], Michele Ciliberto[2], Maria Chiara Spera[2], Romina Caso[2], Sara Rossi[2], Gianluca Boggio[2], Giulia Mastroeni[2], Francesca Abate[2], Eliana Conte[2,9], Anna Quintano[2], Lavinia De Chiara[10], Laura Monti[11], Giovanni Camardese[1,2], Lucio Rinaldi[1,2], Alexia E. Koukopoulos[12], Daniela Pia Rosaria Chieffo[11,13], Gloria Angeletti[4] and Marianna Mazza[1,2]

[1]Department of Psychiatry, Fondazione Policlinico Universitario Agostino Gemelli IRCCS, L.go Agostino Gemelli 8, 00168 Rome, Italy; [2]Department of Neuroscience, Section of Psychiatry, Università Cattolica del Sacro Cuore, 00168 Rome, Italy; [3]Department of Medicine and Surgery, University of Milano-Bicocca, Monza, Italy; [4]Department of Neurosciences, Mental Health, and Sensory Organs (NESMOS), Sapienza University of Rome, Sant'Andrea Hospital, Via di Grottarossa 1035-1039, 00189 Rome, Italy; [5]Psychiatry Residency Training Programme, Faculty of Medicine and Psychology, Sapienza University of Rome, Via di Grottarossa 1035-1039, 00189 Rome, Italy; [6]ASL RM1, Presidio Ospedaliero San Filippo Neri, Servizio Psichiatrico di Diagnosi e Cura, Via Martinotti, 20, 00136 Rome, Italy; [7]UOC Psichiatria, Day Hospital, Sant'Andrea Teaching Hospital, Sapienza University of Rome, Via di Grottarossa 1035-1039, 00189 Rome, Italy; [8]Marina Militare Italiana (Italian Navy), Defense Ministry of Italy, Piazza della Marina, 4, 00196 Rome, Italy; [9]Early Intervention Unit, ASL Roma 3, 00152 Rome, Italy; [10]Struttura Residenziale Psichiatrica Samadi S.p.A., via di Grottarossa Km. 2.200, 00189 Rome, Italy and Centro Lucio Bini, Via Crescenzio 42, 00193 Rome, Italy; [11]UOS Clinical Psychology, Clinical Government, Fondazione Policlinico Universitario Agostino Gemelli IRCCS, 00168 Rome, Italy; [12]Azienda Ospedaliera Universitaria Policlinico Umberto I, Viale dell'Università 30, 00185 Rome, Italy and Centro Lucio Bini, Via Crescenzio 42, 00193 Rome, Italy; [13]Women, Children and Public Health Department, Catholic University of the Sacred Heart, 00168 Rome, Italy

1875-6190/24 $65.00+.00 **© 2024 Bentham Science Publishers**

Table S1. Search strategy on PubMed, PsycINFO and CINAHL (duplicates excluded).
(Toxicity OR teratogenicity OR malformation* OR "birth defect*" OR "congenital abnormal*" OR "brain changes" OR "behavioral abnormalities") AND antipsychotic* AND (pregnancy OR pregnant OR lactation OR delivery OR prenatal OR perinatal OR postnatal OR puerperium) August 8, 2023

1	Torsello R, Palazzetti P. Studi sperimentali sulla ibernazione artificiale farmacologica nello stato gravido-puerperale. II. Studi sulla tossicità acuta e cronica del trattamento [Experimental studies of pharmacological artificial hibernation in pregnancy and puerperium. II. Studies of acute and chronic toxicity of the therapy]. Minerva Ginecol. 1955;7(23):824-6. Italian.	Animal
2	Velardo JT. Induction of pseudopregnancy in adult rats with trilafon, a highly potent tranquilizer of low toxicity. Fertil Steril. 1958;9(1):60-6. doi: 10.1016/s0015-0282(16)32947-8.	Animal
3	Trautner EM, Pennycuik PR, Morris RJ, Gershon S, Shankly KH. The effects of prolonged sub-toxic lithium ingestion on pregnancy in rats. Aust J Exp Biol Med Sci. 1958;36(4):305-21. doi: 10.1038/icb.1958.33.	Animal
4	Stojiljković S, Poleksić J, Milovanović D. Galaktoreja tokom terapije rezerpinom [Galactorrhea during the course of reserpine therapy]. Med Glas. 1959;13:339-42. Serbo-Croatian.	Case
5	Somlyo AP, Waye JD. Abnormal lactation. Report of a case induced by reserpine and a brief review of the subject. J Mt Sinai Hosp N Y. 1960;27:5-9.	Case
6	Lutz JF, Kearney PJ, Babuna C. Extrapyramidal effects due to perphenazine (trilafon): report of 3 cases of stiff-jaw sign. Am J Obstet Gynecol. 1960;79:296-8. doi: 10.1016/0002-9378(60)90191-5.	Case
7	Mackay EV. Progressive chlorpromazine jaundice during pregnancy. Med J Aust. 1960;47(1):209-12. doi: 10.5694/j.1326-5377.1960.tb105307.x.	Case
8	Neghandi DB, Shaper AG. Acute dystonic reaction to perphenazine. East Afr Med J. 1960;37:295.	Case
9	Py O, Mathieu P. Galactorrhée et troubles du cycle menstruel au cours de traitements neuroleptiques [Galactorrhea and disorders of the menstrual cycle in the course of neuroleptic treatments]. Presse Med (1893). 1960;68:765-6. French.	Case
10	Moretti O. Clorpromazina y aparición de galactorrea [Chlorpromazine and appearance of galactorrhea]. Sem Med. 1960;116:954-5. Spanish.	Case
11	Mckeever GE, Alfidi R. Tetanus-like dystonic reaction to triflupromazine hydrochloride. J Mich State Med Soc. 1960;59:1366-8.	Case
12	Fout LR, Kirk RC. Neck-face syndrome, A report of severe reactions to phenothiazine drugs in four patients. Ohio State Med J. 1961;57:405-6.	Case
13	Dede G, Ladu T, Pasolini F. A proposito di una complicazione in corso di terapia con reserpina [Apropos of a complication in the course of reserpine therapy]. Riv Sper Freniatr Med Leg Alien Ment. 1961;85:472-7. Italian.	Case
14	Love W, Peel EL. Chlorpromazine jaundice in pregnancy. J Obstet Gynaecol Br Commonw. 1961;68:628-33. doi: 10.1111/j.1471-0528.1961.tb02781.x.	Case
15	Barten. Clonische krampen na consumptie van een kleine dosis perfenazine (Trilafon) voor emesis gravidarum [Clonic cramps after consumption of a small dose of perphenazine (Trilafon) for emesis gravidarum]. Ned Tijdschr Verloskd Gynaecol. 1961;61:347-9. Dutch.	Case
16	Tachezy R, Vinařová M. Galaktorea v psychiatrické terapii [Galactorrhea in psychiatric therapy]. Act Nerv Super (Praha). 1962;4:235-6. Czech.	Case
17	Cortesi MC. Disturbi disendocrini in corso di trattamento prolungato con clorpromazina [Dysendocrine disorders in the course of prolonged treatment with chlorpromazine]. Rass Studi Psichiatr. 1962;51:220-38. Italian.	Case
18	Tachezy R. Galaktorea u mužů vyvolaná ovariálním hormonem a chlorpromazinem [Galactorrhea in a male induced by ovarian hormone and chlorpromazine]. Cas Lek Cesk. 1962;101:467-9. Czech.	Not in women
19	Willitts BK. Reserpine complications in obstetrical patients. J Indiana State Med Assoc. 1962;55:614-6.	No antipsychotic
20	Werboff J, Dembicki EL. Toxic effects of tranquilizers administered to gravid rats. J Neuropsychiatr. 1962;4:87-91.	Animal
21	Beghi Q. Uso psichiatrico di alcuni psicofarmaci in gravidanza [Psychiatric use of some psychopharmacological drugs in pregnancy]. Riv Sper Freniatr Med Leg Alien Ment. 1963;87:829-51. Italian.	Review
22	McQueen EG. Toxic effects of phenothiazine tranquillizers. N Z Med J. 1963;62:460-2.	Opinion
23	Delerue J. L'action tératogène des médicaments chez la femme enceinte [Teratogenic action of drugs in the pregnant woman]. J Sci Med Lille. 1963;81:497-505. French.	Review

24	Goldberg A. Therapeutic considerations in the diseases of porphyrin metabolism. S Afr J Lab Clin Med. 1963;14:249-55.	Unrelated
25	Hart PG, Holshuijsen N. Bacteriuele shock in het nageboortetijdperk nadat drie dagen van te voren de vruchtvliezen waren gebroken [Bacterial shock in the placental period 3 days after the rupture of the membranes]. Ned Tijdschr Verloskd Gynaecol. 1963;63:377-94. Dutch.	Case
26	Weicker H. Teratogene Substanzen? [Teratogenic substances?]. Med Klin. 1963;58:2032-7. German.	Opinion
27	West GB. Teratogenic activity of drugs. J Pharm Pharmacol. 1964;16:63-4. doi: 10.1111/j.2042-7158.1964.tb07381.x.	Opinion
28	O'Leary JL, O'Leary JA. Nonthalidomide ectromelia: Report of a case. Obstet Gynecol. 1964;23:17-20.	Case
29	Vivell O. Teratogene Substanzen? [Teratogenic substances?]. Med Klin. 1964;59:187. German.	Opinion
30	Cohlan SQ. Fetal and neonatal hazards from drugs administered during pregnancy. N Y State J Med. 1964;64:493-9.	Case
31	Goldman AS, Yakovac WC. Prevention of salicylate teratogenicity in immobilized rats by certain central nervous system depressants. Proc Soc Exp Biol Med. 1964;115:693-6. doi: 10.3181/00379727-115-29009.	Animal
32	Winberg J. Utredning roerande det eventuella sambandet mellan fosterskador och laekemedel. IV. Retrospektiv undersoekning roerande medicinkonsumtion hos moedrar till missbildade barn [Investigation on the possible relation of fetal injuries and drugs. IV. Retrospective study on drug consumption among mothers with malformed children]. Sven Lakartidn. 1964;61:890-902. Swedish.	Unfocused
33	Dowling HF, Lepper MH. Hepatic reactions to tetracycline. JAMA. 1964;188:307-9. doi: 10.1001/jama.1964.03060290111037.	No antipsychotic
34	Arena JM. Report from the Duke University Poison Control Center. Drug dangers (maternal medication) to the fetus. N C Med J. 1964;25:210-1.	Case
35	doering GK, hossfeld C. Über die Gefahren einer übertriebenen Medikamentenfurcht in der Schwangerschaft Untersuchungen über den Einfluss der Hypermesis gravidarum sowie einiger Antiemetika (Meclizin, Phenothiazinderivate) auf die Missbildungsrate [On the hazards of an exaggerated fear of drugs in pregnancy. Studies on the influence of hyperemesis gravidarum as well as various antiemetics (meclizine, phenothiazine derivatives) on the incidence of malformations]. Dtsch Med Wochenschr. 1964;89:1069-72. German. doi: 10.1055/s-0028-1111257.	Unfocused
36	Takacs I, Ruzicska G, Czoevek Z. A hirepin szülészeti alkalmazása [Obstetrical use of hirepine]. Orv Hetil. 1964;105:1319-21. Hungarian.	Case
37	Harley JD. Gross congenital malformations in siblings of children with glucose-6-phosphate dehydrogenase-deficient erythrocytes. Australas Ann Med. 1964;13:204-6. doi: 10.1111/imj.1964.13.3.204.	Unrelated
38	Brown RA, West GB. Effect of acetylsalicylic acid on foetal rats. J Pharm Pharmacol. 1964;16:563-5. doi: 10.1111/j.2042-7158.1964.tb07514.x.	Animal
39	Tuchmann-Duplessis H, Mercier-Parot L. Répercussions des neuroleptiques et des antitumoraux sur le développement prénatal [Repercussions of neuroleptic and antitumor agents on prenatal development]. Bull Schweiz Akad Med Wiss. 1964;20:490-526. French.	Review
40	Nelmans FA. Geneesmiddelen die aan een gravida niet mogen worden toegediend [Drugs which should not be given to a pregnant woman]. Ned Tijdschr Geneeskd. 1964;108:2271-6. Dutch.	Opinion
41	Ravina JH. Les thérapeutiques dangereuses chez la femme enceinte [Hazardous therapeutics in the pregnant woman]. Presse Med (1893). 1964;72:3057-9. French.	Opinion
42	Sinclair JG, Abreu BE. Transplacental effects of drugs in mice. Tex Rep Biol Med. 1965;23(4):849-53.	Animal
43	Dahl M, Sillanpaeae M. Aidille annettu reserpiini vaikean nenaen tukkoisuuden syynae vastasyntyneellae [Severe nasal obstruction in the newborn infant caused by transplacental reserpine]. Duodecim. 1965;81:309-10. Swedish.	No antipsychotic
44	Goldman AS, Yakovac WC. Teratogenic action in rats of reserpine alone and in combination with salicylate and immobilization. Proc Soc Exp Biol Med. 1965;118:857-62. doi: 10.3181/00379727-118-29990.	Animal
45	Gauthier J, Monnet P, Salle B. Malfaçons type ectromélie. Discussion sur le rôle tératogène de médications au cours de la grossesse [Ectromelic type defects. Discussion of the teratogenic role of medications during pregnancy]. Pediatrie. 1965;20(4):489-93. French.	Opinion
46	Lecyk M. The effect of hypothermia applied in the given stages of pregnancy on the number and form of vertebrae in the offspring of white mice. Experientia. 1965;21(8):452-3. doi: 10.1007/BF02150814.	Animal
47	Dacić Z. Beitrag zur medikamentösen Therapie der Fehl- und Frühgeburten [Contribution to the drug therapy of abortions and premature deliveries]. Zentralbl Gynakol. 1965;87(44):1514-9. German.	Case
48	Samorajski T, Ordy JM, Rolsten C. Prenatal chlorpromazine effects on liver enzymes, glycogen, and	Animal

	ultrastructure in mice offspring. Am J Pathol. 1965;47(5):803-31.	
49	Ordy JM, Samorajski T, Collins RL, Rolsten C. Prenatal chlorpromazine effects on liver, survival and behavior of mice offspring. J Pharmacol Exp Ther. 1966;151(1):110-25. PMID: 5902168.	Animal
50	Cretti A. Wie kann den Schwierigkeiten in der antihypertensiven Behandlung von Spätgestosen vorgebeugt werden? [How can the difficulties in the antihypertensive therapy of prolonged pregnancy be prevented?]. Zentralbl Gynäkol. 1966;88(24):770-7. German.	No antipsychotics
51	Cretti A. Skojarzone leczenie późnych zatruć ciazowych nowoczesnymi lekami [Combined treatment of late pregnancy toxemias with modern preparations]. Ginekol Pol. 1967;38(3):303-11. Polish.	Case
52	López-Llera M. Eclampsia 1963-1966. Evaluation of the treatment of 107 cases. J Obstet Gynaecol Br Commonw. 1967;74(3):379-84. doi: 10.1111/j.1471-0528.1967.tb03962.x.	Unfocused
53	Hoffeld DR, Webster RL, McNew J. Adverse effects on offspring of tranquilizing drugs during pregnancy. Nature. 1967;215(5097):182-3. doi: 10.1038/215182b0.	Animal
54	Moayer M. Phenothiazine im Tierexperiment und bei Hyperemesis gravidarum [Phenothiazine in animal experiments and in hyperemesis gravidarum]. Med Klin. 1967;62(29):1137-41. German.	Animal
55	Seay PH, Field WE. Toxicological studies on haloperidol. Int J Neuropsychiatry. 1967;3:Suppl 1:19-21.	Review
56	Blom van Assendelft PM, Dorhout Mees EJ, Hart PG. Vermindering van neonatale sterfte door behandeling van zwangeren lijkende aan essentiële hypertensie [Decrease in neonatal mortality through treatment of essential hypertension in pregnant women]. Ned Tijdschr Geneeskd. 1968;112(24):1115-8. Dutch.	Unrelated
57	Tucker RM, Hunt JC. Recent advances in the medical and surgical treatment of hypertension. Med Clin North Am. 1968;52(5):1227-36.	Unrelated
58	Hartmann-von Monakow K. Die spezielle Therapie der extrapyramidal- motorischen Syndrome [Specific therapy of extrapyramidal-motor syndromes]. Bibl Psychiatr Neurol. 1969;139:705-11. German.	No pregnancy
59	Vince DJ. Congenital malformations following phenothiazine administration during pregnancy. Can Med Assoc J. 1969;100(4):223.	Opinion
60	Pestel M. Médications modernes des nausées et des vomissements. Etude clinique et physiopathologique [New drugs for nausea and vomiting. A clinical and physiological study]. Presse Med (1893). 1969;77(25):921-3. French.	Unrelated
61	Fratta ID. Nicotinamide deficiency and thalidomide potential teratogenic disturbances in Long-Evans rats. Lab Anim Care. 1969;19(5):727-32.	Animal
62	Kornetsky C. Psychoactive drugs in the immature organism. Psychopharmacologia. 1970;17(2):105-36. doi: 10.1007/BF00402703.	Review
63	Vichi F, Pierleoni P. Effetti letali e teratogeni dell'aloperidolo in embroni di topo [Lethal and teratogenic effects of haloperidol in mouse embryos]. Riv Ital Stomatol. 1970;25(7):585-96. Italian.	Animal
64	Vernadakis A, Clark CV. Effects of prenatal administration of psychotropic drugs to rats on brain butylcholinesterase activity at birth. Brain Res. 1970;21(3):460-3. doi: 10.1016/0006-8993(70)90428-2.	Animal
65	Ullberg S, Lindquist NG, Sjòstrand SE. Accumulation of chorio-retinotoxic drugs in the foetal eye. Nature. 1970;227(5264):1257-8. doi: 10.1038/2271257a0.	No antipsychotic
66	Kamakhin AP, Leonov BV, Smol'nikova NM, Strekalova SN. Сравнительное изучение действия аминазина и фторацизина на ранний эмбриогенез мышей (in vitro и in vivo) [Comparative study of the effects of aminazine and fluoracizine on early embriogenensis in mice (in vitro and in vivo)]. Akush Ginekol (Mosk). 1971;47(3):52-5. Russian.	Animal
67	Farkas G, Farkas G Jr. Teratogene Wirkung von Hyperemesis gravidarum und der bei ihrer Behandlung üblichen Medikamente [Teratogenic effects of hyperemesis gravidarum and of the customary drugs used in its therapy]. Zentralbl Gynakol. 1971;93(10):325-30. German.	Unfocused
68	Zelson C, Rubio E, Wasserman E. Neonatal narcotic addiction: 10 year observation. Pediatrics. 1971;48(2):178-89.	No antipsychotic
69	Sharpe CJ, Shadbolt RS, Ashford A, Ross JW. Phenacylthioimidazolines and 3-aryl-5,6-dihydroimidazo(2,1-b)thiazoles with antidepressant activity. J Med Chem. 1971;14(10):977-82. doi: 10.1021/jm00292a023.	No antipsychotic
70	Aoki FY, Ruedy J. Severe lithium intoxication management without dialysis and report of a possible teratogenic effect of lithium. Can Med Assoc J. 1971;105(8):847-8.	No antipsychotic
71	Drugs and the unborn child. Dent Anaesth Sedat. 1972;1(1):5-8.	Opinion
72	Shapiro IuL, Vaĭntrub MIa, Grinberg KN, Zhurkov VS. Тератогенный и мутагенный эффект некоторых противоэпилептических и психотропных препаратов (обзор литературы) [Teratogenic and mutagenic effects of anticonvulsive and psychotropic drugs (review of the literature)]. Zh Nevropatol Psikhiatr Im S S	Review

	Korsakova. 1972;72(6):934-40. Russian.	
73	Beall JR. A teratogenic study of chlorpromazine, orphenadrine, perphenzaine, and LSD-25 in rats. Toxicol Appl Pharmacol. 1972;21(2):230-6. doi: 10.1016/0041-008x(72)90065-8.	Animal
74	Vernadakis A. Spontaneous seizures in rats treated with chlorpromazine during postnatal development. Experientia. 1972;28(2):173-4. doi: 10.1007/BF01935738.	Animal
75	Kiriushchenkov AP, Smol'nikova NM, Skosyreva AM. Влияние аминазина и хлорацизина на ранний эмбриогенез [The effect of aminazine and chlorazicin on early embryogenesis]. Akush Ginekol (Mosk). 1972;48(9):56-8. Russian.	Animal
76	Beliles RP. The influence of pregnancy on the acute toxicity of various compounds in mice. Toxicol Appl Pharmacol. 1972;23(4):537-40. doi: 10.1016/0041-008x(72)90094-4. PMID: 4674811.	Animal
77	Rao MS, Nair P, Pratap C. Induction of mutations by thioridazine hydrochloride, an active ingredient of Mellaril. Indian J Exp Biol. 1973;11(5):403-4.	Animal
78	Farkas G, Farkas G Jr. Stellt die Blutung in der Frühschwangerschaft und ihre medikamentöse Behandlung ein erhöhtes teratogenetisches Risiko dar [Proceedings: Is hemorrhage in early pregnancy and its drug therapy an increased risk of teratogenesis?]. Arch Gynäkol. 1973;214(1):81-2. German. doi: 10.1007/BF00671060.	Abstract
79	Givant Y, Shani J, Goldhaber G, Serebrenik R, Sulman FG. Pharmacology of three mammotropic butyrophenones in the rat. Arch Int Pharmacodyn Ther. 1973;205(2):317-27. PMID: 4797373.	Animal
80	Goldman AS. Developmental defects a final common pathway of teratogenicity? Clin Pediatr (Phila). 1973;12(11):627-8.	Opinion
81	Lemke H, Oettel M, Chemnitius KH. Pharmakologisch-endokrinologische Befunde bei der Prüfung von TURISYNCHRON) und SUISYNCHRON) im Tierexperiment. 2. Mitteilung: Toxikologische Befunde [Pharmacologic-endocrinological findings in animal experiments with TURISYNCHRON and SUISYNCHROM. 2. Toxicologic findings]. Arch Exp Veterinarmed. 1974;28(5):651-70. German.	Animal
82	Szabo KT, Brent RL. Letter Species differences in experimental teratogenesis by tranquillising agents. Lancet. 1974;1(7857):565. doi: 10.1016/s0140-6736(74)92749-4.	Opinion
83	Walker BE, Patterson A. Induction of cleft palate in mice by tranquilizers and barbiturates. Teratology. 1974;10(2):159-63. doi: 10.1002/tera.1420100212.	Animal
84	Gupta T, Sengupta K, Chatterjee A. Failure of clomiphene to interrupt gestation in rats treated with reserpine. J Reprod Fertil. 1974;41(2):379-83. doi: 10.1530/jrf.0.0410379.	Animal
85	Matsuyama SS, Jarvik LF. Cytogenetic effects of psychoactive drugs. Mod Probl Pharmacopsychiatry. 1975;10:99-132. doi: 10.1159/000397922.	Review
86	McCullar FW, Heggeness L. Limb malformations following maternal use of haloperidol. JAMA. 1975;231(1):62-4. doi: 10.1001/jama.231.1.62.	Case
87	Archer JD. Editorial Another possible teratogen? JAMA. 1975;231(1):69. doi: 10.1001/jama.231.1.69c.	Opinion
88	Rieder RO, Rosenthal D, Wender P, Blumenthal H. The offspring of schizophrenics. Fetal and neonatal deaths. Arch Gen Psychiatry. 1975;32(2):200-11. doi: 10.1001/archpsyc.1975.01760200064006.	**Included**
89	Black IB, Reis DJ. Ontogeny of the induction of tyrosine hydroxylase by reserpine in the superior cervical ganglion, nucleus locus coeruleus and adrenal gland. Brain Res. 1975;84(2):269-78. doi: 10.1016/0006-8993(75)90981-6.	Unrelated
90	Yanai J, Sze PY, Ginsburg BE. Effects of aminergic drugs and glutamic acid on audiogenic seizures induced by early exposure to ethanol. Epilepsia. 1975;16(1):67-71. doi: 10.1111/j.1528-1157.1975.tb04722.x.	Animal
91	Ho CK, Kaufman RL, McAlister WH. Congenital malformations. Cleft palate, congenital heart disease, absent tibiae, and polydactyly. Am J Dis Child. 1975;129(6):714-6. doi: 10.1001/archpedi.1975.02120430050014.	Case
92	Ershova VP. О влиянии хронических инъекций аминазина на оогенез и развитие потомства животных (белых мышей) [Influence of chronic injections of aminazin on the oogenesis and development of the progeny of animals (white mice)]. Farmakol Toksikol. 1975;38(4):473-6. Russian.	Animal
93	Ananth J. Congenital malformations with psychopharmacologic agents. Compr Psychiatry. 1975;16(5):437-45. doi: 10.1016/0010-440x(75)90033-4. PMID: 240643.	Review
94	Rumeau-Rouquette C, Goujard J, Huel G. Les médicaments du système nerveux sont-ils teratogenes? Bilan des recherches épidémiologiques [Are nervous system drugs teratogenic? Evaluation of epidemiologic studies]. Arch Fr Pediatr. 1976;33(1):5-10. French.	Review
95	Druga A. The effect of perphenazine treatment during the organogenesis in rats. Acta Biol Acad Sci Hung. 1976;27(1):15-23. PMID: 998109.	Animal
96	Coyle I, Wayner MJ, Singer G. Behavioral teratogenesis: a critical evaluation. Pharmacol Biochem Behav. 1976;4(2):191-200. doi: 10.1016/0091-3057(76)90014-9.	Review

97	Rise A. Neuroleptika [Neuroleptics]. Tidsskr Nor Laegeforen. 1976;96(7):446-7. Norwegian.	Opinion
98	Milkovich L, van den Berg BJ. An evaluation of the teratogenicity of certain antinauseant drugs. Am J Obstet Gynecol. 1976;125(2):244-8. doi: 10.1016/0002-9378(76)90601-3.	**Included**
99	Takeuchi K, Okabe S, Takagi K. Influence of pregnancy on the development of various gastric lesions in rats. Am J Dig Dis. 1976;21(10):853-8. doi: 10.1007/BF01072076.	Animal
100	Patel AJ, Béndek G, Balazs R. Do drugs acting on the nervous system affect cell proliferation in the developing brain. Lancet. 1977;1(8008):399-401.	Unfocused
101	Buravlev VM. Особенности влияние ин витро психофармакологических средств на эмбриональную тканы мозга плодов болних шизофрение матерей [In vitro effect of psychopharmacologic drugs on the embryonic brain tissue of the fetuses of schizophrenic mothers]. Zh Nevropatol Psikhiatr Im S S Korsakova. 1978;78(7):1070-5. Russian.	Unfocused
102	Marsboom R. Toxikologische Untersuchungen von Neuroleptika der Butyrophenonreihe und verwandter Substanzen [Toxicologic studies on butyrophenone neuroleptics and related substances]. Int Pharmacopsychiatry. 1978;13 Suppl 1:3-14. German.	Review
103	Airaksinen MM, Ho BT, An R, Taylor D. Major pharmacological effects of 6-methoxytetrahydro-beta-carboline, a drug elevating the tissue 5-hydroxytryptamine level. Arzneimittelforschung. 1978;28(1):42-6.	No antipsychotic
104	Singh S, Padmanabhan R. Teratogenic effects of chlorpromazine hydrochloride in rat foetuses. Indian J Med Res. 1978 Feb;67:300-9.	Animal
105	Singh S, Padmanabhan R. Prolongation of gestation & retarded postnatal growth & mortality induced by chlorpromazine in rats. Indian J Exp Biol. 1978;16(5):542-5.	Animal
106	Szczurek Z, Smigla K, Ciołkosz I, Bober A, Głab J, Trzeciak HI, Herman ZS. Obraz patomorfologiczny narzadów wewnetrznych szczurów po długotrwałym stosowaniu neuroleptyków [Pathomorphological changes in rat internal organs following long-term administration of neuroleptics]. Patol Pol. 1978;29(3):347-58. Polish.	Animal
107	Fukuhara K, Emi Y, Furukawa T, Fujii T, Iwanami K, Watanabe N, Tsubura Y. Toxicological and teratological studies of 2-chloro-11-(2-dimethylaminoethoxy)-dibenzo[b,f]thiepine (zotepine), a new neuroleptic drug. Arzneimittelforschung. 1979;29(10):1600-6.	Review
108	Lanza JP, Goude F, Lanza M. Effets d'une dose unique de quelques neuroleptiques à action prolongée sur le cycle estral et la glande mammaire de la ratte [Effect of a single dose of some long-acting neuroleptics on the estrus cycle and the mammary gland of the rat]. C R Seances Soc Biol Fil. 1979;173(4):797-806. French.	Animal
109	Singh S, Padmanabhan R. Effect of chlorpromazine on skeletogenesis. The result of maternal administration of the drug in experimental rats. Acta Orthop Scand. 1979;50(2):151-9. doi: 10.3109/17453677908989750.	Animal
110	Chou SM, Miike T, Payne WM, Davis GJ. Neuropathology of "spinning syndrome" induced by prenatal intoxication with a PCB in mice. Ann N Y Acad Sci. 1979;320:373-95. doi: 10.1111/j.1749-6632.1979.tb56619.x.	Animal
111	Jarvik ME. Necessary risks. N Engl J Med. 1979;300(23):1330. doi: 10.1056/NEJM197906073002309.	Opinion
112	Vorhees CV, Brunner RL, Butcher RE. Psychotropic drugs as behavioral teratogens. Science. 1979;205(4412):1220-5. doi: 10.1126/science.472738.	Animal
113	Koch M, Oettel M, Lauterbach H, Freund H. Induction of pituitary tumours and hyperprolactinemia in female rats by estrogens. The effect of apomorphine, reserpine and L-dopa. Arch Toxicol Suppl. 1980;4:89-91. doi: 10.1007/978-3-642-67729-8_24.	Animal
114	Roĭzin L. Многофакторность патогенеза патологических реакций, наблюдаемых при введении психотропных средств [Multifactorial nature of the pathogenesis of pathological reactions observed following the administration of psychotropic drugs]. Zh Nevropatol Psikhiatr Im S S Korsakova. 1980;80(7):1097-100. Russian.	Unfocused
115	Redmond GP, Hirshman MF. Postnatal growth retardation in rats produced by the phenothiazine perphenazine. Pediatr Pharmacol (New York). 1980;1(2):153-60.	Animal
116	Nurnberg HG. Treatment of mania in the last six months of pregnancy. Hosp Community Psychiatry. 1980;31(2):122-6. doi: 10.1176/ps.31.2.122.	Opinion
117	Druga A, Nyitray M, Szaszovszky E. Experimental teratogenicity of structurally similar compounds with or without piperazine-ring: a preliminary report. Pol J Pharmacol Pharm. 1980;32(2):199-204.	Animal
118	Singh S, Padmanabhan R. Placental changes in chlorpromazine induced teratogenesis in rats--a histochemical study. Indian J Exp Biol. 1980;18(4):344-50.	Animal
119	Robertson RT, Majka JA, Peter CP, Bokelman DL. Effects of prenatal exposure to chlorpromazine on postnatal development and behavior of rats. Toxicol Appl Pharmacol. 1980;53(3):541-9. doi: 10.1016/0041-008x(80)90367-1.	Animal

120	Papadimitriou K. Dehydrobenzperidol as part of a general anaesthetic technique at 30 weeks gestation. Anaesthesia. 1980;35(9):919-20. doi: 10.1111/j.1365-2044.1980.tb03957.x.	Case
121	Gropp C, Havemann K. Toxische Schädigungen durch zytostatische Arzneimittel [Toxic lesions caused by cytostatic drugs]. Internist (Berl). 1980;21(12):739-45. German.	Review
122	Agrawal AK, Squibb RE. Effects of acrylamide given during gestation on dopamine receptor binding in rat pups. Toxicol Lett. 1981;7(3):233-8. doi: 10.1016/0378-4274(81)90074-6.	Animal
123	Hays DP. Teratogenesis: a review of the basic principles with a discussion of selected agents: Part II. Drug Intell Clin Pharm. 1981;15(7-8):542-66. doi: 10.1177/1060028081015007-805.	Review
124	Leonard BE. Effect of psychotropic drugs administered to pregnant rats on the behaviour of the offspring. Neuropharmacology. 1981;20(12B):1237-42.	Animal
125	Kreek MJ, Hartman N. Chronic use of opioids and antipsychotic drugs: side effects, effects on endogenous opioids, and toxicity. Ann N Y Acad Sci. 1982;398:151-72. doi: 10.1111/j.1749-6632.1982.tb39489.x.	Review
126	Gill TS, Guram MS, Geber WF. Haloperidol teratogenicity in the fetal hamster. Dev Pharmacol Ther. 1982;4(1-2):1-5. doi: 10.1159/000457384. PMID: 7117084.	Animal
127	Goodman DR, James RC, Harbison RD. Placental toxicology. Food Chem Toxicol. 1982;20(1):123-8. doi: 10.1016/s0278-6915(82)80018-5.	Review
128	Donaldson GL, Bury RG. Multiple congenital abnormalities in a newborn boy associated with maternal use of fluphenazine enanthate and other drugs during pregnancy. Acta Paediatr Scand. 1982;71(2):335-8. doi: 10.1111/j.1651-2227.1982.tb09428.x.	Case
129	Henkler G, Klotzbach M, Koch H, Müller W, Richter J. Fortschritte auf dem Gebiet der Arzneimittelentwicklung. Teil 15 [Progress in the area of drug development. 15]. Pharmazie. 1982;37(11):753-65. German.	Review
130	Apter JT, Apter AS, Tyano S. Side Effects and toxicity of lithium. J Fam Pract. 1982;15(6):1101-6.	No antipsychotic
131	Rosengarten H, Friedman E, Friedhoff AJ. Sensitive periods for the effect of haloperidol on development of striatal dopamine receptors. Birth Defects 1983;19(4):511-3. PMID: 6871420.	Animal
132	Verdeal K, Ertürk E, Rose DP. Effects of reserpine administration on rat mammary tumors and uterine disease induced by N-nitrosomethylurea. Eur J Cancer Clin Oncol. 1983;19(6):825-34. doi: 10.1016/0277-5379(83)90015-9.	Animal
133	Arzamastsev EV, Mironova MI, Krepkova LV, Bortnikova VV, Kuznetsov IuV. Доклиническое изучение безвредности нового отечественного транквилизатора гендарина [Preclinical study of the safety of the new Soviet tranquilizer gindarin]. Farmakol Toksikol. 1983 Jul-Aug;46(4):107-12. Russian.	No antipsychotic
134	Lucchi L, Covelli V, Petkov VV, Spano PF, Trabucchi M. Effects of ethanol, given during pregnancy, on the offspring dopaminergic system. Pharmacol Biochem Behav. 1983 Oct;19(4):567-70. doi: 10.1016/0091-3057(83)90328-3.	No antipsychotic
135	Nurnberg HG, Prudic J. Guidelines for treatment of psychosis during pregnancy. Hosp Community Psychiatry. 1984 Jan;35(1):67-71. doi:10.1176/ps.35.1.67.	Review
136	Edlund MJ, Craig TJ. Antipsychotic drug use and birth defects: an epidemiologic reassessment. Compr Psychiatry. 1984;25(1):32-7. doi: 10.1016/0010-440x(84)90019-1. PMID: 6141893.	Review
137	Paragas MG. Lithium adverse reactions in psychiatric patients. Pharmacol Biochem Behav. 1984;21(Suppl 1):65-9. doi: 10.1016/0091-3057(84)90165-5.	No antipsychotic
138	Lucchi L, Covelli V, Spano PF, Trabucchi M. Acute ethanol administration during pregnancy: effects on central dopaminergic transmission in rat offspring. Neurobehav Toxicol Teratol. 1984;6(1):19-21.	Animal
139	Härnryd C, Bjerkenstedt L, Björk K, Gullberg B, Oxenstierna G, Sedvall G, Wiesel FA, Wik G, Aberg-Wistedt A. Clinical evaluation of sulpiride in schizophrenic patients--a double-blind comparison with chlorpromazine. Acta Psychiatr Scand Suppl. 1984;311:7-30. doi: 10.1111/j.1600-0447.1984.tb06856.x.	Unrelated
140	DeHaven DL, Krigman MR, Gaynor JJ, Mailman RB. The effects of lead administration during development on lithium-induced polydipsia and dopaminergic function. Brain Res. 1984 Apr 16;297(2):297-304. doi: 10.1016/0006-8993(84)90570-5.	No antipsychotic
141	Cuomo V, Ambrosi L, Annau Z, Cagiano R, Brunello N, Racagni G. Behavioural and neurochemical changes in offspring of rats exposed to methyl mercury during gestation. Neurobehav Toxicol Teratol. 1984 May-Jun;6(3):249-54.	Animal
142	Imai S, Tauchi K, Huang KJ, Takeshima T, Sudo T. ラットにおけるブロムペリドールの催奇形性研究 [Teratogenicity study on bromperidol in rats]. J Toxicol Sci. 1984;9(Suppl 1):109-26. Japanese. doi: 10.2131/jts.9.supplementi_109.	Animal

143	Ross RK, Paganini-Hill A, Krailo MD, Gerkins VR, Henderson BE, Pike MC. Effects of reserpine on prolactin levels and incidence of breast cancer in postmenopausal women. Cancer Res. 1984;44(7):3106-8.	Unfocused
144	McKeon TW, Lorden JF, Oltmans GA, Beales M, Walkley SU. Decreased catalepsy response to haloperidol in the genetically dystonic (dt) rat. Brain Res. 1984;308(1):89-96. doi: 10.1016/0006-8993(84)90920-x.	Animal
145	Buelke-Sam J, Kimmel GL, Webb PJ, Slikker W Jr, Newport GD, Nelson CJ, Kimmel CA. Postnatal toxicity following prenatal reserpine exposure in rats: effects of dose and dosing schedule. Fundam Appl Toxicol. 1984;4(6):983-91.	Animal
146	Rodríguez González MD, Friman Pérez M. Teratogenic effect of trifluoperazine in rats and mice. Acta Biol Hung. 1985;36(3-4):233-7.	Animal
147	Watanabe T, Matsuhashi K, Takayama S. 出生前に自律神経系に作用する薬剤を投与されたラットの出生後の神経行動発達に関する研究 [Study on the postnatal neuro-behavioral development in rats treated prenatally with drugs acting on the autonomic nervous systems]. Nihon Yakurigaku Zasshi. 1985;85(2):79-90. Japanese. doi: 10.1254/fpj.85.79.	Animal
148	Itsubo M, Kameda H. 中毒性肝炎の定義と分類 [Definition and classification of toxic hepatitis]. Nihon Rinsho. 1985;43(6):1103-7. Japanese.	Unrelated
149	Breese GR, Napier TC, Mueller RA. Dopamine agonist-induced locomotor activity in rats treated with 6-hydroxydopamine at differing ages: functional supersensitivity of D-1 dopamine receptors in neonatally lesioned rats. J Pharmacol Exp Ther. 1985;234(2):447-55.	Animal
150	Cuomo V, Cagiano R, Renna G, Serinelli A, Brunello N, Racagni G. Comparative evaluation of the behavioural consequences of prenatal and early postnatal exposure to haloperidol in rats. Neurobehav Toxicol Teratol. 1985;7(5):489-92.	Animal
151	Sanberg PR, Pevsner J, Autuono PG, Coyle JT. Fetal methylazoxymethanol acetate-induced lesions cause reductions in dopamine receptor-mediated catalepsy and stereotypy. Neuropharmacology. 1985;24(11):1057-62. doi: 10.1016/0028-3908(85)90191-1.	No antipsychotic
152	Dawson R Jr, Callahan MF, Annau Z. Hypothalamic monoamine metabolism in mice: evaluation of drug challenges and neurotoxic insult. Pharmacology. 1986;32(1):25-37. doi: 10.1159/000138149.	Animal
153	Lancaster FE, Selvanayagam PF, Hsu LL. Lactational ethanol exposure: brain enzymes and [3H]spiroperidol binding. Int J Dev Neurosci. 1986;4(2):151-60. doi: 10.1016/0736-5748(86)90040-7.	No antipsychotic
154	Kola I, Folb PI. Chlorpromazine inhibits the mitotic index, cell number, and formation of mouse blastocysts, and delays implantation of CBA mouse embryos. J Reprod Fertil. 1986;76(2):527-36. doi: 10.1530/jrf.0.0760527.	Animal
155	Robinson GE, Stewart DE, Flak E. The rational use of psychotropic drugs in pregnancy and postpartum. Can J Psychiatry. 1986;31(3):183-90. doi: 10.1177/070674378603100301.	Review
156	Pauli RM, Pettersen BJ. Is reserpine a human teratogen? J Med Genet. 1986;23(3):267-8. doi: 10.1136/jmg.23.3.267.	Case
157	Ali SF, Buelke-Sam J, Slikker W Jr. Prenatal reserpine exposure in rats decreases caudate nucleus dopamine receptor binding in female offspring. Toxicol Lett. 1986;31(3):195-201. doi: 10.1016/0378-4274(86)90126-8.	Animal
158	Nishie K, Cole RJ, Dorner JW. Effects of cyclopiazonic acid on the contractility of organs with smooth muscles, and on frog ventricles. Res Commun Chem Pathol Pharmacol. 1986;53(1):23-37.	Animal
159	Leathem AM. Safety and efficacy of antiemetics used to treat nausea and vomiting in pregnancy. Clin Pharm. 1986;5(8):660-8.	No antipsychotic
160	Takagi S, Alleva FR, Seth PK, Balazs T. Delayed development of reproductive functions and alteration of dopamine receptor binding in hypothalamus of rats exposed prenatally to phenytoin and phenobarbital. Toxicol Lett. 1986;34(1):107-13. doi: 10.1016/0378-4274(86)90152-9.	Animal
161	Baldwin J, Ridings J. Teratogenicity in rats of two dopaminergic agonists. Toxicology. 1986;42(2-3):291-302. doi: 10.1016/0300-483x(86)90017-x.	Animal
162	Harmon JR, Kimmel GL, Webb PJ, Delongchamp RR. Effect of prenatal reserpine exposure on development of the postnatal rat heart. Teratog Carcinog Mutagen. 1987;7(4):347-55. doi: 10.1002/tcm.1770070403.	Animal
163	Elia J, Katz IR, Simpson GM. Teratogenicity of psychotherapeutic medications. Psychopharmacol Bull. 1987;23(4):531-86.	Review
164	Obasaju MF, Wiley LM, Miller L, Samuels SJ, Chang RJ, Overstreet JW. Reproductive effects of chlorpromazine exposure to female mice: cell proliferation disadvantage revealed by the Chimera Embryo Assay. Reprod Toxicol. 1987;1(1):17-23. doi: 10.1016/0890-6238(87)90067-0.	Animal
165	Seth PK, Alleva FR, Takagi S, Yen-Koo HC, Balazs T. Brain neurotransmitter receptor alterations in offspring of rats exposed to phenobarbital, phenytoin or their combination during pregnancy. Neurotoxicology. 1987;8(1):45-53.	Animal

166	Gailis L, Dumas L, Page M. Ambiguous effect of chlorpromazine on doxorubicin activity against P388D1 tumours in mice. Eur J Cancer Clin Oncol. 1988;24(2):169-73. doi: 10.1016/0277-5379(88)90248-9.	Animal
167	Saillenfait AM, Vannier B. Methodological proposal in behavioural teratogenicity testing: assessment of propoxyphene, chlorpromazine, and vitamin A as positive controls. Teratology. 1988;37(3):185-99. doi: 10.1002/tera.1420370303.	Animal
168	Yu JF, Yang YS, Wang WY, Xiong GX, Chen MS. Mutagenicity and teratogenicity of chlorpromazine and scopolamine. Chin Med J (Engl). 1988;101(5):339-45.	Animal
169	Salim AS. Protection by procaine hydrochloride against reserpine-induced acute gastric mucosal injury in the rat: implications for stress-induced injury. J Pharm Sci. 1988;77(7):582-5. doi: 10.1002/jps.2600770707.	Animal
170	Czeizel A. Reserpine is not a human teratogen. J Med Genet. 1988;25(11):787. doi: 10.1136/jmg.25.11.787.	Opinion
171	Nabeshima T, Hiramatsu M, Yamaguchi K, Kasugai M, Ishizaki K, Kawashima K, Itoh K, Ogawa S, Katoh A, Furukawa H, et al. Effects of prenatal administration of phencyclidine on the learning and memory processes of rat offspring. J Pharmacobiodyn. 1988;11(12):816-23. doi: 10.1248/bpb1978.11.816.	Animal
172	Scalzo FM, Newport GD, Gough BJ, Ali SF, Holson RR. Chronic prenatal haloperidol exposure: lack of effect on presynaptic dopamine autoreceptors. Neurotoxicology. 1989;10(3):485-90.	Animal
173	Ali SF, Ahmad G, Slikker W Jr, Bondy SC. Effects of gestational exposure to phencyclidine: distribution and neurochemical alterations in maternal and fetal brain. Neurotoxicology. 1989;10(3):383-92.	No antipsychotic
174	Fung YK, Reed JA, Lau YS. Prenatal cocaine exposure fails to modify neurobehavioral responses and the striatal dopaminergic system in newborn rats. Gen Pharmacol. 1989;20(5):689-93. doi: 10.1016/0306-3623(89)90108-0.	Animal
175	Sitland-Marken PA, Rickman LA, Wells BG, Mabie WC. Pharmacologic management of acute mania in pregnancy. J Clin Psychopharmacol. 1989;9(2):78-87.	Review
176	Fung YK, Lau YS. Effects of prenatal nicotine exposure on rat striatal dopaminergic and nicotinic systems. Pharmacol Biochem Behav. 1989;33(1):1-6. doi: 10.1016/0091-3057(89)90419-x.	Animal
177	Buelke-Sam J, Ali SF, Kimmel GL, Slikker W Jr, Newport GD, Harmon JR. Postnatal function following prenatal reserpine exposure in rats: neurobehavioral toxicity. Neurotoxicol Teratol. 1989;11(5):515-22. doi: 10.1016/0892-0362(89)90028-7.	Animal
178	Sullivan-Jones P, Kimmel CA, Kimmel GL. Prenatal reserpine exposure alters cardiovascular parameters in rat offspring. Fundam Appl Toxicol. 1989;13(4):652-61.	Animal
179	Scalzo FM, Holson RR, Gough BJ, Ali SF. Neurochemical effects of prenatal haloperidol exposure. Pharmacol Biochem Behav. 1989;34(4):721-5. doi: 10.1016/0091-3057(89)90265-7.	Animal
180	Scalzo FM, Ali SF, Holson RR. Behavioral effects of prenatal haloperidol exposure. Pharmacol Biochem Behav. 1989;34(4):727-31. doi: 10.1016/0091-3057(89)90266-9.	Animal
181	Almasio P, Bortolini M, Pagliaro L, Coltorti M. Role of S-adenosyl-L- methionine in the treatment of intrahepatic cholestasis. Drugs. 1990;40(Suppl 3):111-23. doi: 10.2165/00003495-199000403-00011.	Unrelated
182	Saigo K. Inhibitory effect of chlorpromazine on rat reproduction: a test of administration for nine weeks before breeding. Reprod Toxicol. 1990;4(1):29-36. doi: 10.1016/0890-6238(90)90076-8.	Animal
183	Cagiano R, De Salvia MA, Renna G, Tortella E, Braghiroli D, Parenti C, Zanoli P, Baraldi M, Annau Z, Cuomo V. Evidence that exposure to methyl mercury during gestation induces behavioral and neurochemical changes in offspring of rats. Neurotoxicol Teratol. 1990;12(1):23-8. doi: 10.1016/0892-0362(90)90108-o.	Animal
184	Johansson P. Methylazoxymethanol (MAM)-induced brain lesion and oral dyskinesia in rats. Psychopharmacology (Berl). 1990;100(1):72-6. doi:ì 10.1007/BF02245793.	Animal
185	Parant M. Possible mediators in endotoxin-induced abortion. Res Immunol. 1990;141(2):164-8. doi: 10.1016/0923-2494(90)90137-n. PMID: 2202030.	Review
186	Yokoya H. Inhibitory effect of chlorpromazine on rat reproduction: a two week administration test before mating. J Toxicol Sci. 1990;15(1):15-28. doi: 10.2131/jts.15.15.	Animal
187	Watanabe T, Matsuhashi K, Takayama S. Placental and blood-brain barrier transfer following prenatal and postnatal exposures to neuroactive drugs: relationship with partition coefficient and behavioral teratogenesis. Toxicol Appl Pharmacol. 1990;105(1):66-77. doi: 10.1016/0041-008x(90)90359-3.	Animal
188	Hara K. 精神障害および神経障害 [Psychiatric and nervous disorders]. Nihon Sanka Fujinka Gakkai Zasshi. 1990;42(8):847-53. Japanese.	Review
189	Watanabe T, Matsuhashi K, Takayama S. ラットにおける出生前および出生後のクロルプロマジン曝露後の行動催奇形性および胎盤、乳、および血液脳関門への移行 [Behavioral teratogenesis and placental, milk, and blood-brain barrier transfer following prenatal and postnatal exposures to chlorpromazine in rats]. Yakubutsu Seishin Kodo. 1990;10(3):351-62. Japanese.	Animal

190	Kristofová A, Ujházy E, Bezek S, Balonová T, Nosál R. Stobadine toxicity and transplacental movement. Arch Toxicol. 1991; Suppl. 14:280-3. doi: 10.1007/978-3-642-74936-0_60.	Animal
191	Balonová T, Zeljenková D, Durisová M, Nosál R, Jakubovský J, Líska J, Stolc S. Reproductive toxicity studies with cis-(-)-2,3,4,4a,5,9b-hexahydro-2,8-dimethyl-1H-pyrido-[4,3-b]indole dipalmitate in rats. Arzneimittelforschung. 1991;41(1):1-5.	Animal
192	Arcas Cruz R, Figueras Aloy J, Vilanova Juanola JM, Comas Mastmitja L, Jiménez González R, Cruz Hernández M. Recién nacido de madre adicta a las drogas: aspectos maternos, perinatológicos, neonatales y síndrome de abstinencia [Newborn infant of drug-addicted mother: maternal, perinatal, neonatal aspects, and neonatal abstinence syndrome]. An Esp Pediatr. 1991;34(2):123-7. Spanish.	No antipsychotic
193	Brusés JL, Berninsone PM, Ojea SI, Azcurra JM. The circling training rat model as a behavioral teratology test. Pharmacol Biochem Behav. 1991;38(4):739-45. doi: 10.1016/0091-3057(91)90235-t.	Animal
194	Van Gent CM, Sandberg LB, Boucek RJ. Haloperidol administration to rats during pregnancy induces permanent alterations in serum lipoprotein patterns of progeny. J Clin Psychopharmacol. 1991;11(2):113-5.	Animal
195	Ireland FA, Loch WE, Worthy K, Anthony RV. Effects of bromocriptine and perphenazine on prolactin and progesterone concentrations in pregnant pony mares during late gestation. J Reprod Fertil. 1991;92(1):179-86. doi: 10.1530/jrf.0.0920179.	Animal
196	Ali SF, Chang LW, Slikker W Jr. Biogenic amines as biomarkers for neurotoxicity. Biomed Environ Sci. 1991;4(1-2):207-16.	Review
197	Kostrzewa RM, Gong L. Supersensitized D1 receptors mediate enhanced oral activity after neonatal 6-OHDA. Pharmacol Biochem Behav. 1991;39(3):677-82. doi: 10.1016/0091-3057(91)90146-s.	Animal
198	Banerjee J, Ghosh P, Mitra S, Ghosh N, Bhattacharya S. Inhibition of human fetal brain acetylcholinesterase: marker effect of neurotoxicity. J Toxicol Environ Health. 1991;33(3):283-90. doi: 10.1080/15287399109531527.	Unrelated
199	Godet PF, Marie-Cardine M. Neuroleptiques, schizophrénie et grossesse. Étude épidémiologique et tératologique [Neuroleptics, schizophrenia and pregnancy. Epidemiological and teratologic study]. Encéphale. 1991;17(6):543-7. French.	**Included**
200	Balonová T, Ujházy E, Nosál R, Durisová M, Jakubovský J, Líska J. Effect of the cardioprotective agent stobadine on reproduction in rats. Bratisl Lek Listy. 1991;92(12):603-8.	Animal
201	Schwabe R, Thiel R, Chahoud I, Neubert D. Effects of a single haloperidol application to neonatal and early postnatal rats on the neurotransmitter content in the corpus striatum. Arch Toxicol. 1992;66(8):573-9. doi: 10.1007/BF01973388.	Animal
202	Sharma JB, Sharma S. Role of thioridazine in unexplained infertility. Int J Gynaecol Obstet. 1992;37(1):37-41. doi: 10.1016/0020-7292(92)90975-o.	**Included**
203	Ujházy E, Balonová T, Vargová T, Jansák J, Derková L. Teratological study of stobadin after single and repeated administration in rats. Teratog Carcinog Mutagen. 1992;12(5):211-21. doi: 10.1002/tcm.1770120504.	Animal
204	Savasta M, Mennicken F, Chritin M, Abrous DN, Feuerstein C, Le Moal M, Herman JP. Intrastriatal dopamine-rich implants reverse the changes in dopamine D2 receptor densities caused by 6-hydroxydopamine lesion of the nigrostriatal pathway in rats: an autoradiographic study. Neuroscience. 1992;46(3):729-38. doi: 10.1016/0306-4522(92)90159-y.	Animal
205	Mattsson R, Mattsson A, Hansson I, Holmdahl R, Rook GA, Whyte A. Increased levels of prolactin during, but not after, the immunisation with rat collagen II enhances the course of arthritis in DBA/1 mice. Autoimmunity. 1992;11(3):163-70. doi: 10.3109/08916939209035151.	Animal
206	Shurtz-Swirski R, Cohen Y, Barnea ER. Patterns of secretion of human chorionic gonadotrophin by superfused placental explants and the embryo-- placental relationship following maternal use of medications. Hum Reprod. 1992;7(3):300-4. doi: 10.1093/oxfordjournals.humrep.a137639.	Unfocused
207	Williams R, Ali SF, Scalzo FM, Soliman K, Holson RR. Prenatal haloperidol exposure: effects on brain weights and caudate neurotransmitter levels in rats. Brain Res Bull. 1992;29(3-4):449-58. doi: 10.1016/0361-9230(92)90082-9.	Animal
208	Sullivan-Jones P, Hansen DK, Sheehan DM, Holson RR. The effect of teratogens on maternal corticosterone levels and cleft incidence in A/J mice. J Craniofac Genet Dev Biol. 1992;12(4):183-9.	Animal
209	de Cuypere G, Rombaut P, van Moffaert M. Congenital malformations caused by psychotropic drugs in pregnancy. Acta Neuropsychiatr. 1992;4(4):77-85. doi: 10.1017/S0924270800034128.	Review
210	Archer T. Behavioural retardation in the neuropathology of mental retardation. APMIS. 1993; Suppl. 40:35-56.	Animal
211	Scalzo FM, Ali SF, Holson RR, Williams RL. Haloperidol effects on the developing dopamine system: conflicting results and implications for neurobehavioral teratology research. Ann Ist Super Sanità. 1993;29(1):139-46.	Animal

212	Cagiano R, De Salvia MA, Giustino A, Siro Brigiani G, Cuomo V. Behavioral and neurochemical changes produced in rats by developmental treatments with psychotropic drugs. Ann Ist Super Sanità. 1993;29(1):175-7.	Animal
213	Lipska BK, Jaskiw GE, Weinberger DR. Postpubertal emergence of hyperresponsiveness to stress and to amphetamine after neonatal excitotoxic hippocampal damage: a potential animal model of schizophrenia. Neuropsychopharmacology. 1993;9(1):67-75. doi: 10.1038/npp.1993.44.	Animal
214	Kodavanti PR, Mundy WR, Tilson HA, Harry GJ. Effects of selected neuroactive chemicals on calcium transporting systems in rat cerebellum and on survival of cerebellar granule cells. Fundam Appl Toxicol. 1993;21(3):308-16. doi: 10.1006/faat.1993.1103.	Animal
215	Henderson MG, McMillen BA. Changes in dopamine, serotonin and their metabolites in discrete brain areas of rat offspring after in utero exposure to cocaine or related drugs. Teratology. 1993;48(5):421-30. doi: 10.1002/tera.1420480506.	Animal
216	Silberstein SD. Headaches and women: treatment of the pregnant and lactating migraineur. Headache. 1993;33(10):533-40. doi: 10.1111/j.1526-4610.1993.hed3310533.x.	Review
217	Goldberg HL. Psychotropic drugs in pregnancy and lactation. Int J Psychiatry Med. 1994;24(2):129-47. doi: 10.2190/2BF1-0718-WE7F-A9F7.	Review
218	Lipska BK, Weinberger DR. Subchronic treatment with haloperidol and clozapine in rats with neonatal excitotoxic hippocampal damage. Neuropsychopharmacology. 1994;10(3):199-205. doi: 10.1038/npp.1994.22.	Animal
219	Holson RR, Webb PJ, Grafton TF, Hansen DK. Prenatal neuroleptic exposure and growth stunting in the rat: an in vivo and in vitro examination of sensitive periods and possible mechanisms. Teratology. 1994;50(2):125-36. doi: 10.1002/tera.1420500207.	Animal
220	Altshuler LL, Szuba MP. Course of psychiatric disorders in pregnancy. Dilemmas in pharmacologic management. Neurol Clin. 1994;12(3):613-35.	Review
221	Bond GR, Van Zee A. Overdosage of misoprostol in pregnancy. Am J Obstet Gynecol. 1994;171(2):561-2. doi: 10.1016/0002-9378(94)90302-6.	No antipsychotic
222	Bloomquist J, King E, Wright A, Mytilineou C, Kimura K, Castagnoli K, Castagnoli N Jr. 1-Methyl-4-phenylpyridinium-like neurotoxicity of a pyridinium metabolite derived from haloperidol: cell culture and neurotransmitter uptake studies. J Pharmacol Exp Ther. 1994;270(2):822-30. PMID: 8071874.	Unrelated
223	Merlob P, Stahl B, Maltz E. Is fluphenazine a teratogen? Am J Med Genet. 1994;52(2):231-2. doi: 10.1002/ajmg.1320520221.	Case
224	Ujházy E, Dubovický M, Balonová T, Jansák J, Zeljenková D. Teratological assessment of stobadine after single and repeated administration in mice. J Appl Toxicol. 1994;14(5):357-63. doi: 10.1002/jat.2550140507.	Animal
225	Tarantino R, Bishop E, Chen FC, Iqbal K, Malick AW. N-methyl-2-pyrrolidone as a cosolvent: relationship of cosolvent effect with solute polarity and the presence of proton-donating groups on model drug compounds. J Pharm Sci. 1994;83(9):1213-6. doi: 10.1002/jps.2600830905.	Unrelated
226	Ujházy E, Navarová J, Líska J, Balonová T, Vargová T. Determination of selective biochemical parameters in pregnant and lactating rats after stobadine administration. Methods Find Exp Clin Pharmacol. 1994;16(8):569-73.	Animal
227	Pons G, Rey E, Matheson I. Excretion of psychoactive drugs into breast milk. Pharmacokinetic principles and recommendations. Clin Pharmacokinet. 1994;27(4):270-89. doi: 10.2165/00003088-199427040-00003.	Review
228	Gross ME, Clifford CA, Hardy DA. Excitement in an elephant after intravenous administration of atropine. J Am Vet Med Assoc. 1994;205(10):1437-8.	Animal
229	Henck JW, Petrere JA, Anderson JA. Developmental neurotoxicity of CI-943: a novel antipsychotic. Neurotoxicol Teratol. 1995;17(1):13-24. doi: 10.1016/0892-0362(94)00054-h.	Animal
230	Ujházy E, Faberová V, Dubovický M, Zemánek M, Jansák J, Dedík L, Durisová M. Transplacentárny prechod stobadínu u králikov v rôznych stádiach gravidity--aproximácia farmakokinetickým modelom [Transplacental transfer of stobadine in rabbits in various stages of pregnancy--an approximation using a pharmacokinetic model]. Cesk Fysiol. 1995;44(1):15-7. Slovak.	Animal
231	Imanishi M, Yoneyama M, Takagi S, Takeuchi M. Collaborative work to determine an optimal administration period and optimal parameters for detection of effects on male fertility in rats--male reproductive toxicity study of haloperidol. J Toxicol Sci. 1995;20(3):297-307. doi: 10.2131/jts.20.297.	Animal
232	Handal M, Matheson I, Bechensteen AG, Lindemann R. Antipsykotika og gravide. En kasuistikk [Antipsychotic agents and pregnant women. A case report]. Tidsskr Nor Laegeforen. 1995 Aug 30;115(20):2539-40. Norwegian.	Case
233	Sharma SK, Herrera ER, Sidawi JE, Leveno KJ. The pregnant patient with an intracranial arteriovenous malformation. Cesarean or vaginal delivery using regional or general anesthesia? Reg Anesth. 1995;20(5):455-8	Unrelated
234	Chorvatovicová D, Ujházy E. Transplacental effect of stobadine on cyclophosphamide induced micronucleus	Animal

	frequency in mice. Mutagenesis. 1995;10(6):531-4. doi: 10.1093/mutage/10.6.531.	
235	Zhang J, Wang L, Pitts DK. Prenatal haloperidol reduces the number of active midbrain dopamine neurons in rat offspring. Neurotoxicol Teratol. 1996;18(1):49-57. doi: 10.1016/0892-0362(95)02023-3.	Animal
236	Dubovický M, Kovacovský P, Rychlík I, Ujházy E, Gajdosík A. Effect of long-term administration of stobadine to rats on selective variables of spontaneous behaviour of their offspring. Gen Physiol Biophys. 1996;15(2):181-6.	Animal
237	Abdel-Hamid HA, Abdel-Rahman MS, Abdel-Rahman SA. Teratogenic effect of diphenylhydantoin and/or fluphenazine in mice. J Appl Toxicol. 1996;16(3):221-5. doi: 10.1002/(SICI)1099-1263(199605)16:3<221::AID-JAT336>3.0.CO;2-Q.	Animal
238	Besch TK, Ruble DL, Gibbs PH, Pitt ML. Steady-state minute volume determination by body-only plethysmography in juvenile rhesus monkeys. Lab Anim Sci. 1996;46(5):539-44. PMID: 8905587.	Animal
239	Benson KA, Ali SF, Wilson MC. The effects of prenatal cocaine exposure on dopaminergic challenge and receptor binding in Wistar rats. Ann N Y Acad Sci. 1996;801:289-300. doi: 10.1111/j.1749-6632.1996.tb17449.x.	Animal
240	Dubovický M, Ujházy E, Kovacovský P, Rychlík I, Kalnovicová T, Navarová J, Turcáni P, Durisová M, Gajdosík A. Effect of long-term administration of stobadine on exploratory behaviour and on striatal levels of dopamine and serotonin in rats and their offspring. J Appl Toxicol. 1997;17(1):63-70. doi: 10.1002/(sici)1099-1263(199701)17:1<63::aid-jat396>3.0.co;2-m.	Animal
241	Yang X, Kulkarni AP. Oxidation of phenothiazines by human term placental peroxidase in non-smokers. Teratog Carcinog Mutagen. 1997;17(3):139-51.	Unfocused
242	Goldaber KG. Psychotropics. Semin Perinatol. 1997;21(2):154-9. doi:10.1016/s0146-0005(97)80059-6.	Review
243	Chisholm CA, Kuller JA. A guide to the safety of CNS-active agents during breastfeeding. Drug Saf. 1997;17(2):127-42. doi: 10.2165/00002018-199717020-00005.	Review
244	Pinkofsky HB. Psychosis during pregnancy: treatment considerations. Ann Psychiatry. 1997;9(3):175-9. doi: 10.1023/a:1026234125565.	Case
245	Loupe PS, Schroeder SR, Tessel RE. Effects of neuroleptic and anticonvulsant drugs on repeated acquisition learning in microencephalic and normal rats. Exp Clin Psychopharmacol. 1997;5(4):323-33. doi: 10.1037//1064-1297.5.4.323.	Animal
246	García-Gil L, De Miguel R, Muñoz RM, Cebeira M, Villanua MA, Ramos JA, Fernández-Ruiz JJ. Perinatal delta(9)-tetrahydrocannabinol exposure alters the responsiveness of hypothalamic dopaminergic neurons to dopamine-acting drugs in adult rats. Neurotoxicol Teratol. 1997;19(6):477-87. doi: 10.1016/s0892-0362(97)00048-2.	Animal
247	Hannigan JH, Hackett JA, Tilak J, Subramanian MG. Sulpiride-induced increases in serum prolactin levels in female rats exposed prenatally to alcohol. Alcohol. 1997;14(6):585-92. doi: 10.1016/s0741-8329(97)00053-0.	Animal
248	Cohen LS, Rosenbaum JF. Psychotropic drug use during pregnancy: weighing the risks. J Clin Psychiatry. 1998;59(Suppl 2):18-28.	Review
249	Bollweg G, Sparber SB. Relationships between midembryonic 5-HT2 agonist and/or antagonist exposure and detour learning by chickens. Pharmacol Biochem Behav. 1998;60(1):47-53. doi: 10.1016/s0091-3057(97)00555-8.	Animal
250	Poeggeler B, Rassoulpour A, Guidetti P, Wu HQ, Schwarcz R. Dopaminergic control of kynurenate levels and N-methyl-D-aspartate toxicity in the developing rat striatum. Dev Neurosci. 1998;20(2-3):146-53. doi: 10.1159/000017309.	Animal
251	Cantalamessa F, Barili P, Cavagna R, Sabbatini M, Tenore G, Amenta F. Influence of neonatal treatment with the pyrethroid insecticide cypermethrin on the development of dopamine receptors in the rat kidney. Mech Ageing Dev. 1998;103(2):165-78. doi: 10.1016/s0047-6374(98)00039-6.	Animal
252	Schrott LM, Getty ME, Wacnik PW, Sparber SB. Open-field and LPS-induced sickness behavior in young chickens: effects of embryonic cocaine and/or ritanserin. Pharmacol Biochem Behav. 1998;61(1):9-17. doi: 10.1016/s0091-3057(98)00013-6.	Animal
253	Singh KP, Jaiswal AK, Singh M, Bhattacharya SK. Behavioural alterations in rats induced by single prenatal exposure of haloperidol. Indian J Exp Biol. 1998;36(11):1102-7.	Animal
254	Yoshida K, Smith B, Kumar R. Psychotropic drugs in mothers' milk: a comprehensive review of assay methods, pharmacokinetics and of safety of breast-feeding. J Psychopharmacol. 1999;13(1):64-80. doi: 10.1177/026988119901300108.	Review
255	Bortolozzi AA, Duffard RO, Evangelista de Duffard AM. Behavioral alterations induced in rats by a pre- and postnatal exposure to 2,4-dichlorophenoxyacetic acid. Neurotoxicol Teratol. 1999;21(4):451-65. doi: 10.1016/s0892-0362(98)00059-2.	Animal

256	Takashima H, Tsujihata M, Kishikawa M, Freed WJ. Bromocriptine protects dopaminergic neurons from levodopa-induced toxicity by stimulating D(2)receptors. Exp Neurol. 1999;159(1):98-104. doi: 10.1006/exnr.1999.7122.	Unrelated
257	Schrott LM, Sweeney WA, Bodensteiner KE, Sparber SB. Late embryonic ritanserin exposure fails to alter normal responses to immune system stimulation in young chicks. Pharmacol Biochem Behav. 1999;64(1):81-8. doi:10.1016/s0091-3057(99)00096-9.	Animal
258	Ujházy E, Dubovický M, Soltés L, Faberová V, Zemánek M, Gajdosík A. Placental transfer of stobadine in rabbits. Life Sci. 1999;65(18-19):2011-4. doi: 10.1016/s0024-3205(99)00467-1.	Animal
259	Reiff-Eldridge R, Heffner CR, Ephross SA, Tennis PS, White AD, Andrews EB. Monitoring pregnancy outcomes after prenatal drug exposure through prospective pregnancy registries: a pharmaceutical company commitment. Am J Obstet Gynecol. 2000;182(1 Pt 1):159-63. doi: 10.1016/s0002-9378(00)70506-0.	No antipsychotic
260	Dubovický M, Ujházy E, Kovacovský P, Rychlík I, Navarová J, Jansák J.Antioxidant stobadine and neurobehavioural development of the rat offspring. Gen Physiol Biophys. 1999;18(Spec No):41-7.	Animal
261	Navarová J, Ujházy E, Dubovický M. Protective effect of the antioxidant stobadine against cyclophosphamide and irradiation induced oxidative stress. Gen Physiol Biophys. 1999;18(Spec No):112-9.	Unrelated
262	Ujházy E, Dubovický M, Balonová T, Jansák J. Teratological study of the antioxidant stobadine in rats. Gen Physiol Biophys. 1999;18(Spec No):171-6.	Animal
263.	Chaudron LH, Jefferson JW. Mood stabilizers during breastfeeding: a review.J Clin Psychiatry. 2000;61(2):79-90. doi: 10.4088/jcp.v61n0202.	Review
264	Goldstein DJ, Corbin LA, Fung MC. Olanzapine-exposed pregnancies and lactation: early experience. J Clin Psychopharmacol. 2000;20(4):399-403.doi: 10.1097/00004714-200008000-00002.	**Included**
265	Pinkofsky HB. Effects of antipsychotics on the unborn child: what is known and how should this influence prescribing? Paediatr Drugs. 2000;2(2):83-90. doi: 10.2165/00148581-200002020-00001.	Review
266	Parmeggiani L, Belmonte A, Ferrari AR, Perucca E, Guerrini R. Add-on lamotrigine treatment in children and young adults with severe partial epilepsy:an open, prospective, long-term study. J Child Neurol. 2000;15(10):671-4.doi: 10.1177/088307380001501006.	Unrelated
267	Koren G. Misrepresentation and miscommunication of teratogenic risk of drugs; analysis of three highly publicized international cases. Reprod Toxicol.2001;15(1):1-3. doi: 10.1016/s0890-6238(00)00123-4.	Case
268	Rasmussen EB, Newland MC. Developmental exposure to methylmercury alters behavioral sensitivity to D-amphetamine and pentobarbital in adult rats. Neurotoxicol Teratol. 2001;23(1):45-55. doi:10.1016/s0892-0362(00)00112-4.	Animal
269	Iqbal MM, Gundlapalli SP, Ryan WG, Ryals T, Passman TE. Effects of antimanic mood-stabilizing drugs on fetuses, neonates, and nursing infants. South Med J. 2001;94(3):304-22.	Review
270	Ujházy E, Dubovický M, Faberová V, Zemánek M, Soltés L, Gajdosík A, Eybl V.Placental transfer of the antioxidant stobadine at different gestational stages in rabbits. Methods Find Exp Clin Pharmacol. 2000;22(9):683-8. doi:10.1358/mf.2000.22.9.802284.	Animal
271	Marchi NS, Azoubel R, Tognola WA. Teratogenic effects of lamotrigine on rat fetal brain: a morphometric study. Arq Neuropsiquiatr. 2001;59(2-B):362-4.doi: 10.1590/s0004-282x2001000300010.	Animal
272	Retamal P, Cantillano V. Tratamiento de la enfermedad bipolar durante el embarazo y puerperio. Caso clínico [Treatment of bipolar disorder during pregnancy and puerperium period. A case report]. Rev Med Chil. 2001;129(5):556-60. Spanish.	Case
273	Koren G. Maternal obesity and risk of neural tube defects. Can Fam Physician. 2001;47:1385, 1387.	No antipsychotic
274	Singh KP, Singh M. Effect of single prenatal haloperidol exposure on hippocampus and striatum of developing rat brain. Indian J Exp Biol. 2001;39(3):223-9.	Animal
275	Su KP, Shen WW, Huang SY. Omega-3 fatty acids as a psychotherapeutic agent for a pregnant schizophrenic patient. Eur Neuropsychopharmacol. 2001;11(4):295-9. doi: 10.1016/s0924-977x(01)00098-0.	Case
276	Schrott LM, Sparber SB. Embryonic "binge" cocaine exposure alters neural-immune and neural-endocrine interactions in young chickens: involvement of serotonin(2) receptors. Brain Res Dev Brain Res. 2001;130(1):99-107. doi:10.1016/s0165-3806(01)00217-6.	Animal
277	Doehaerd S. Les médicaments neuropsychiatriques pendant la grossesse [Neuropsychiatric drugs during pregnancy]. Rev Med Brux. 2001 Sep;22(4):A264-6.French. PMID: 11680186.	Review
278	Rocha JB, Rocha LK, Emanuelli T, Pereira ME. Effect of mercuric chloride and lead acetate treatment during the second stage of rapid post-natal brain growth on the behavioral response to chlorpromazine and on delta-ALA-D activity in weaning rats. Toxicol Lett. 2001;125(1-3):143-50. doi:10.1016/s0378-4274(01)00435-0.	Animal

279	Wang C, McInnis J, Ross-Sanchez M, Shinnick-Gallagher P, Wiley JL, Johnson KM. Long-term behavioral and neurodegenerative effects of perinatal phencyclidine administration: implications for schizophrenia. Neuroscience.2001;107(4):535-50. doi: 10.1016/s0306-4522(01)00384-0.	Animal
280	Dominguez M, Díaz Obregón MC, Bhathal H, Santiago R. Epilepsia y embarazo [Epilepsy and pregnancy]. Rev Neurol. 2001;33(12):1179-85. Spanish.	Review
281	Borrell J, Vela JM, Arévalo-Martin A, Molina-Holgado E, Guaza C. Prenatal immune challenge disrupts sensorimotor gating in adult rats. Implications for the etiopathogenesis of schizophrenia. Neuropsychopharmacology. 2002;26(2):204-15. doi: 10.1016/S0893-133X(01)00360-8.	Animal
282	Yan QS. Reduced serotonin release and serotonin uptake sites in the rat nucleus accumbens and striatum after prenatal cocaine exposure. Brain Res. 2002;929(1):59-69. doi: 10.1016/s0006-8993(01)03378-9.	Animal
283	Beghi E, Annegers JF; Collaborative Group for the Pregnancy Registries in Epilepsy. Pregnancy registries in epilepsy. Epilepsia. 2001;42(11):1422-5.doi: 10.1046/j.1528-1157.2001.11201.x.	Unfocused
284	Baldessarini RJ, Tondo L, Hennen J, Viguera AC. Is lithium still worth using? An update of selected recent research. Harv Rev Psychiatry. 2002;10(2):59-75.	Review
285	Tottori K, Nakai M, Uwahodo Y, Miwa T, Yamada S, Oshiro Y, Kikuchi T, Altar CA. Attenuation of scopolamine-induced and age-associated memory impairments by the sigma and 5-hydroxytryptamine(1A) receptor agonist OPC-14523 (1-[3-[4-(3-chlorophenyl)-1-piperazinyl]propyl]-5-methoxy-3,4-dihydro-2[1H]-quinolinonemonomethanesulfonate). J Pharmacol Exp Ther. 2002;301(1):249-57. doi:10.1124/jpet.301.1.249.	Unrelated
286	Ernst CL, Goldberg JF. The reproductive safety profile of mood stabilizers, atypical antipsychotics, and broad-spectrum psychotropics. J Clin Psychiatry.2002;63(Suppl 4):42-55.	Review
287	Pfuhlmann B, Stoeber G, Beckmann H. Postpartum psychoses: prognosis, risk factors, and treatment. Curr Psychiatry Rep. 2002;4(3):185-90. doi:10.1007/s11920-002-0025-6.	Review
288	Gil-ad I, Shtaif B, Shiloh R, Weizman A. Evaluation of the neurotoxic activity of typical and atypical neuroleptics: relevance to iatrogenic extrapyramidal symptoms. Cell Mol Neurobiol. 2001;21(6):705-16. doi:10.1023/a:1015152021192.	Animal
289	Bushnell PJ, Moser VC, MacPhail RC, Oshiro WM, Derr-Yellin EC, Phillips PM,Kodavanti PR. Neurobehavioral assessments of rats perinatally exposed to a commercial mixture of polychlorinated biphenyls. Toxicol Sci. 2002;68(1):109-20. doi: 10.1093/toxsci/68.1.109.	Animal
290	Viguera AC, Cohen LS, Baldessarini RJ, Nonacs R. Managing bipolar disorder during pregnancy: weighing the risks and benefits. Can J Psychiatry. 2002;47(5):426-36. doi: 10.1177/070674370204700503.	Review
291	Bortolozzi A, Duffard R, Antonelli M, Evangelista de Duffard AM. Increased sensitivity in dopamine D2-like brain receptors from 2,4-dichlorophenoxyacetic acid (2,4-D)-exposed and amphetamine-challenged rats. Ann N Y Acad Sci. 2002;965:314-23. doi: 10.1111/j.1749-6632.2002.tb04173.x.	Animal
292	Singh KP, Singh M. Effect of prenatal haloperidol exposure on behavioral alterations in rats. Neurotoxicol Teratol. 2002;24(4):497-502. doi:10.1016/s0892-0362(02)00189-7.	Animal
293	Gabay MP. Galactogogues: medications that induce lactation. J Hum Lact.2002;18(3):274-9. doi: 10.1177/089033440201800311. PMID: 12192964.	Review
294	Ward RK, Zamorski MA. Benefits and risks of psychiatric medications during pregnancy. Am Fam Physician. 2002;66(4):629-36.	Review
295	Schrott LM, Baumgart MI, Zhang X, Sparber SB. Prenatal opiate withdrawal activates the chick embryo hypothalamic pituitary-adrenal axis and dilates vitelline blood vessels via serotonin(2) receptors. J Pharmacol Exp Ther. 2002;303(1):257-64. doi: 10.1124/jpet.102.037044.	Animal
296	Tennis P, Eldridge RR; International Lamotrigine Pregnancy Registry Scientific Advisory Committee. Preliminary results on pregnancy outcomes in women using lamotrigine. Epilepsia. 2002;43(10):1161-7. doi:10.1046/j.1528-1157.2002.45901.x.	No antipsychotic
297	Cissoko H, Jonville-Béra AP, Autret-Leca E. Exposition in utero aux nouveaux antiépileptiques: issue de grossesse de 12 patientes traitées [New antiepileptic drugs in pregnancy: outcome of 12 exposed pregnancies]. Thérapie.2002;57(4):397-401. French.	No antipsychotic
298	Koren G. Comment on antiepileptics and antipsychotics. Teratology. 2002;66(6):273; author reply 274. doi: 10.1002/tera.10115. PMID: 12486757.	Opinion
299	Petris MJ, Smith K, Lee J, Thiele DJ. Copper-stimulated endocytosis and degradation of the human copper transporter, hCtr1. J Biol Chem. 2003;278(11):9639-46. doi: 10.1074/jbc.M209455200. Epub 2002 Dec 25.	Unrelated
300	Patton SW, Misri S, Corral MR, Perry KF, Kuan AJ. Antipsychotic medication during pregnancy and lactation in women with schizophrenia: evaluating the risk.Can J Psychiatry. 2002;47(10):959-65. doi: 10.1177/070674370204701008.	Review

301	Suzuki T, Mizuo K, Nakazawa H, Funae Y, Fushiki S, Fukushima S, Shirai T,Narita M. Prenatal and neonatal exposure to bisphenol-A enhances the central dopamine D1 receptor-mediated action in mice: enhancement of the methamphetamine-induced abuse state. Neuroscience. 2003;117(3):639-44. doi:10.1016/s0306-4522(02)00935-1.	Animal
302	Nordeng H, Spigset O. Bruk av antipsykotika ved graviditet og amming [Use of antipsychotics during pregnancy and lactation]. Tidsskr Nor Laegeforen. 2003;123(15):2033-5. Norwegian.	Review
303	Harwood AJ. Neurodevelopment and mood stabilizers. Curr Mol Med. 2003;3(5):472-82. doi: 10.2174/1566524033479672.	Review
304	Vajda FJ, O'Brien TJ, Hitchcock A, Graham J, Lander C. The Australian registry of anti-epileptic drugs in pregnancy: experience after 30 months. J Clin Neurosci. 2003;10(5):543-9. doi: 10.1016/s0967-5868(03)00158-9.	No antipsychotic
305	Schwarz A, Górniak SL, Bernardi MM, Dagli ML, Spinosa HS. Effects of Ipomoea carnea aqueous fraction intake by dams during pregnancy on the physical and neurobehavioral development of rat offspring. Neurotoxicol Teratol. 2003;25(5):615-26. doi: 10.1016/s0892-0362(03)00078-3.	Animal
306	Nonacs R, Cohen LS. Assessment and treatment of depression during pregnancy: an update. Psychiatr Clin North Am. 2003;26(3):547-62. doi: 10.1016/s0193-953x(03)00046-7.	Review
307	Nguyen HN, Lalonde P. Clozapine et grossesse [Clozapine and pregnancy].Encéphale. 2003;29(2):119-24. French.	Review
308	Bhardwaj SK, Beaudry G, Quirion R, Levesque D, Srivastava LK. Neonatal ventral hippocampus lesion leads to reductions in nerve growth factor inducible-B mRNA in the prefrontal cortex and increased amphetamine response in the nucleus accumbens and dorsal striatum. Neuroscience. 2003;122(3):669-76.doi: 10.1016/j.neuroscience.2003.08.016.	Animal
309	Francis F, Marty-Detraves C, Poincloux R, Baricault L, Fournier D, Paquereau L. Fungal lectin, XCL, is internalized via clathrin-dependent endocytosis and facilitates uptake of other molecules. Eur J Cell Biol. 2003;82(10):515-22. doi: 10.1078/0171-9335-00338.	Unrelated
310	Sabers A, Dam M, A-Rogvi-Hansen B, Boas J, Sidenius P, Laue Friis M, Alving J, Dahl M, Ankerhus J, Mouritzen Dam A. Epilepsy and pregnancy: lamotrigine as main drug used. Acta Neurol Scand. 2004;109(1):9-13. doi:10.1034/j.1600-0404.2003.00200.x.	No antipsychotic
311	Navarová J, Ujházy E, Líska J, Dubovický M. Determination of selective biochemical variables in pregnant and lactating mice after stobadine administration. Methods Find Exp Clin Pharmacol. 2003;25(9):717-21.	Animal
312	Padmanabhan R, Abdulrazzaq YM, Bastaki SM, Shafiullah M, Chandranath SI.Experimental studies on reproductive toxicologic effects of lamotrigine in mice.Birth Defects Res B Dev Reprod Toxicol. 2003;68(5):428-38. doi:10.1002/bdrb.10042. PMID: 14745993.	Animal
313	Yonkers KA, Wisner KL, Stowe Z, Leibenluft E, Cohen L, Miller L, Manber R,Viguera A, Suppes T, Altshuler L. Management of bipolar disorder during pregnancy and the postpartum period. Am J Psychiatry. 2004;161(4):608-20.doi: 10.1176/appi.ajp.161.4.608.	Review
314	Ujházy E, Mach M, Dubovický M, Navarová J, Soltés L, Juránek I, Brucknerová I, Zeman M. Effect of melatonin and stobadine on maternal and embryofoetal toxicity in rats due to intrauterine hypoxia induced by phenytoin administration. Cent Eur J Public Health. 2004;12(Suppl):S83-6.	Animal
315	Gentile S. Clinical utilization of atypical antipsychotics in pregnancy and lactation. Ann Pharmacother. 2004;38(7-8):1265-71. doi: 10.1345/aph.1D485.	Review
316	Dodd S, Berk M. The pharmacology of bipolar disorder during pregnancy and breastfeeding. Expert Opin Drug Saf. 2004;3(3):221-9. doi: 10.1517/eods.3.3.221.31074.	Review
317	Wolansky MJ, Soiza-Reilly M, Fossati M, Azcurra JM. Postnatal haloperidol eliminates the deficit in circling behavior produced by prenatal exposure to the same drug. Neurotoxicol Teratol. 2004;26(4):561-9. doi:10.1016/j.ntt.2004.04.006.	Animal
318	Tohmi M, Tsuda N, Watanabe Y, Kakita A, Nawa H. Perinatal inflammatory cytokine challenge results in distinct neurobehavioral alterations in rats: implication in psychiatric disorders of developmental origin. Neurosci Res. 2004;50(1):67-75. doi:10.1016/j.neures.2004.05.010.	Animal
319	Forrester MB, Stanley SK. Exposures and treatments among women of childbearing age and pregnant women reported to Texas poison centers. Vet HumToxicol. 2004;46(4):210-2.	No antipsychotic
320	de Haan GJ, Edelbroek P, Segers J, Engelsman M, Lindhout D, Dévilé-Notschaele M, Augustijn P. Gestation-induced changes in lamotrigine pharmacokinetics: a monotherapy study. Neurology. 2004;63(3):571-3. doi:10.1212/01.wnl.0000133213.10244.fd	No antipsychotic
321	Daoud AS, Bataineh H, Otoom S, Abdul-Zahra E. The effect of Vigabatrin,Lamotrigine and Gabapentin on the fertility, weights, sex hormones and biochemical profiles of male rats. Neuro Endocrinol Lett. 2004;25(3):178-83.	Animal

322	Yablonsky-Alter E, Gashi E, Lidsky TI, Wang HY, Banerjee SP. Clozapine protection against gestational cocaine-induced neurochemical abnormalities. JPharmacol Exp Ther. 2005;312(1):297-302. doi: 10.1124/jpet.104.074062.	Animal
323	Tomson T, Perucca E, Battino D. Navigating toward fetal and maternal health: the challenge of treating epilepsy in pregnancy. Epilepsia. 2004;45(10):1171-5. doi: 10.1111/j.0013-9580.2004.15104.x.	No antipsychotic
324	Smith MS, Evatt ML. Movement disorders in pregnancy. Neurol Clin. 2004 Nov;22(4):783-98. doi: 10.1016/j.ncl.2004.06.005.	Review
325	Allison SK. Psychotropic medication in pregnancy: ethical aspects and clinical management. J Perinat Neonatal Nurs. 2004;18(3):194-205. doi:10.1097/00005237-200407000-00003.	Review
326	Sladden M, Mortimer N, Chave T. Toxic epidermal caused by lamotrigine. Aust Fam Physician. 2004;33(10):829-30.	Case
327	Tavakoli-Nezhad M, Pitts DK. Postnatal inorganic lead exposure reduces midbrain dopaminergic impulse flow and decreases dopamine D1 receptor sensitivity in nucleus accumbens neurons. J Pharmacol Exp Ther. 2005;312(3):1280-8. doi: 10.1124/jpet.104.076166.	Animal
328	Karlov VA. Стратегия и тактика терапии эпилепсии сегодня [Therapy of epilepsy: current strategy and tactics]. Zh Nevrol Psikhiatr Im S S Korsakova.2004;104(8):28-34. Russian.	Review
329	Kawashima H, Iida Y, Kitamura Y, Saji H. Binding of 4-(4-chlorophenyl)-1-[4-(4-fluorophenyl)-4-oxobutyl]pyridinium ion (HPP+), a metabolite of haloperidol, to synthetic melanin: implications for the dopaminergic neurotoxicity of HPP+. Neurotox Res. 2004;6(7-8):535-42. doi:10.1007/BF03033449.	Animal
330	Zuckerman L, Weiner I. Maternal immune activation leads to behavioral and pharmacological changes in the adult offspring. J Psychiatr Res. 2005;39(3):311-23. doi: 10.1016/j.jpsychires.2004.08.008.	Animal
331	Curtis V. Women are not the same as men: specific clinical issues for female patients with bipolar disorder. Bipolar Disord. 2005;7 Suppl 1:16-24.doi: 10.1111/j.1399-5618.2005.00190.x.	Review
332	Diav-Citrin O, Shechtman S, Ornoy S, Arnon J, Schaefer C, Garbis H,Clementi M, Ornoy A. Safety of haloperidol and penfluridol in pregnancy: a multicenter, prospective, controlled study. J Clin Psychiatry. 2005;66(3):317-22. doi: 10.4088/jcp.v66n0307.	**Included**
333	Penovich P, Gaily E. What can we say to women of reproductive age with epilepsy? Neurology. 2005;64(6):938-9. doi: 10.1212/01.WNL.0000154464.36610.91. Erratum in: Neurology. 2005;64(11):1991.	Opinion
334	Cunnington M, Tennis P; International Lamotrigine Pregnancy Registry Scientific Advisory Committee. Lamotrigine and the risk of malformations in pregnancy. Neurology. 2005;64(6):955-60. doi:10.1212/01.WNL.0000154515.94346.89.	No antipsychotic
335	Mazaira S. Efectos de los psicofármacos en el feto y el recién nacido.Consecuencias del tratamiento de los trastornos psiquiátricos durante el embarazo y la lactancia [Effects of psychiatric drugs on the fetus and newbornchildren. Consequences of the treatment of psychiatric disorders during pregnancy and lactation]. Vertex. 2005;16(59):35-42. Spanish.	Review
336	McKenna K, Koren G, Tetelbaum M, Wilton L, Shakir S, Diav-Citrin O, Levinson A, Zipursky RB, Einarson A. Pregnancy outcome of women using atypical antipsychotic drugs: a prospective comparative study. J Clin Psychiatry. 2005;66(4):444-9; quiz 546. doi: 10.4088/jcp.v66n0406.	**Included**
337	Drug treatments for bipolar disorder: 2--maintenance, prevention and special situations. Drug Ther Bull. 2005;43(5):33-7. doi: 10.1136/dtb.2005.43533.	Review
338	Nielsen GL, Nørgard B, Puho E, Rothman KJ, Sørensen HT, Czeizel AE. Risk of specific congenital abnormalities in offspring of women with diabetes. Diabet Med. 2005;22(6):693-6. doi: 10.1111/j.1464-5491.2005.01477.x.	Unfocused
339	Trixler M, Gáti A, Fekete S, Tényi T. Use of antipsychotics in the management of schizophrenia during pregnancy. Drugs. 2005;65(9):1193-206. doi: 10.2165/00003495-200565090-00002.	Review
340	Jain AE, Lacy T. Psychotropic drugs in pregnancy and lactation. J Psychiatr Pract. 2005;11(3):177-91. doi: 10.1097/00131746-200505000-00005.	Review
341	Richtand NM, Taylor B, Welge JA, Ahlbrand R, Ostrander MM, Burr J, Hayes S, Coolen LM, Pritchard LM, Logue A, Herman JP, McNamara RK. Risperidone pretreatment prevents elevated locomotor activity following neonatal hippocampal lesions. Neuropsychopharmacology. 2006 Jan;31(1):77-89. doi: 10.1038/sj.npp.1300791.	Animal
342	Gao YD, Xiong DS, Xu YF, Peng H, Shao XF, Yang CZ, Zhu ZP. [Construction and expression of anti-CD3/ anti-Pgp Diabody]. Sheng Wu Gong Cheng Xue Bao. 2003;19(4):444-9. Chinese. PMID: 15969062.	Unrelated
343	Lambert T, Brennan A, Castle D, Kelly DL, Conley RR. Perception of depot antipsychotics by mental health professionals. J Psychiatr Pract. 2003 May;9(3):252-60. doi: 10.1097/00131746-200305000-00011. PMID: 15985940.	Unrelated

344	Freeman MP, Gelenberg AJ. Bipolar disorder in women: reproductive events and treatment considerations. Acta Psychiatr Scand. 2005 Aug;112(2):88-96. doi: 10.1111/j.1600-0447.2005.00526.x.	Review
345	Li M, Budin R, Fleming AS, Kapur S. Effects of novel antipsychotics, amisulpiride and aripiprazole, on maternal behavior in rats. Psychopharmacology (Berl). 2005;181(3):600-10. doi: 10.1007/s00213-005-0091-7.	Animal
346	Carneiro LM, Diógenes JP, Vasconcelos SM, Aragão GF, Noronha EC, Gomes PB,Viana GS. Behavioral and neurochemical effects on rat offspring after prenatal exposure to ethanol. Neurotoxicol Teratol. 2005;27(4):585-92. doi:10.1016/j.ntt.2005.06.006	Animal.
347	Eberhard-Gran M, Eskild A, Opjordsmoen S. Treating mood disorders during pregnancy: safety considerations. Drug Saf. 2005;28(8):695-706. doi:10.2165/00002018-200528080-00004.	Review
348	Iqbal MM, Aneja A, Rahman A, Megna J, Freemont W, Shiplo M, Nihilani N, Lee K. The potential risks of commonly prescribed antipsychotics: during pregnancy and lactation. Psychiatry (Edgmont). 2005;2(8):36-44.	Review
349	Bhat R, Chari G, Rao R. Effects of prenatal cocaine, morphine, or both on postnatal opioid (mu) receptor development. Life Sci. 2006;78(13):1478-82. doi: 10.1016/j.lfs.2005.07.023.	Animal
350	Howard LM. Atypical antipsychotic use during the first trimester of pregnancy may not increase major malformations. Evid Based Ment Health. 2005Nov;8(4):115. doi: 10.1136/ebmh.8.4.115.	Opinion
351	Gille G, Radad K, Reichmann H, Rausch WD. Synergistic effect of alpha-dihydroergocryptine and L-dopa or dopamine on dopaminergic neurons in primary culture. J Neural Transm (Vienna). 2006;113(9):1107-18. doi:10.1007/s00702-005-0369-2.	Unrelated
352	Crosignani PG. Current treatment issues in female hyperprolactinaemia. Eur J Obstet Gynecol Reprod Biol. 2006;125(2):152-64. doi: 10.1016/j.ejogrb.2005.10.005.	Review
353	Levin ED, Christopher NC. Effects of clozapine on memory function in the rat neonatal hippocampal lesion model of schizophrenia. Prog Neuropsychopharmacol Biol Psychiatry. 2006;30(2):223-9. doi:10.1016/j.pnpbp.2005.10.018. Epub 2005 Dec 13	Animal.
354	Májeková M, Koprda V, Bohácik L, Bohov P, Hadgraft J, Bezáková Z, Májek P.Skin permeation of acyl derivatives of stobadine. Drug Deliv. 2006;13(1):51-4. doi: 10.1080/10717540500313729.	Unrelated
355	Hauser WA, Tomson T. Lamotrigine and the risk of malformations inpregnancy. Neurology. 2006;66(1):153-4; author reply 153-4. doi:10.1212/01.wnl.0000203710.88804.29.	Opinion
356	Penschuck S, Flagstad P, Didriksen M, Leist M, Michael-Titus AT. Decrease in parvalbumin-expressing neurons in the hippocampus and increased phencyclidine-induced locomotor activity in the rat methylazoxymethanol (MAM) model of schizophrenia. Eur J Neurosci. 2006;23(1):279-84. doi:10.1111/j.1460-9568.2005.04536.x.	Animal
357	Brodie MJ. Major congenital malformations and antiepileptic drugs:prospective observations. J Neurol Neurosurg Psychiatry. 2006;77(2):145.doi: 10.1136/jnnp.2005.079376.	Opinion
358	Moldzio R, Radad K, Duvigneau JC, Kranner B, Krewenka C, Piskernik C,Rausch WD. Glutamate-induced cell death and formation of radicals can be reduced by lisuride in mesencephalic primary cell culture. J Neural Transm (Vienna).2006;113(9):1095-105. doi: 10.1007/s00702-005-0394-1.	Animal
359	Arora M, Praharaj SK. Meningocele and ankyloblepharon following in uteroexposure to olanzapine. Eur Psychiatry. 2006;21(5):345-6. doi:10.1016/j.eurpsy.2006.01.014.	Case
360	Höcht C, Opezzo JA, Taira CA. Applicability of reverse microdialysis in pharmacological and toxicological studies. J Pharmacol Toxicol Methods. 2007;55(1):3-15. doi: 10.1016/j.vascn.2006.02.007.	Review
361	Gentile S. Prophylactic treatment of bipolar disorder in pregnancy and breastfeeding: focus on emerging mood stabilizers. Bipolar Disord. 2006;8(3):207-20. doi: 10.1111/j.1399-5618.2006.00295.x.	Review
362	Mendhekar DN, Sunder KR, Andrade C. Aripiprazole use in a pregnant schizoaffective woman. Bipolar Disord. 2006;8(3):299-300. doi: 10.1111/j.1399-5618.2006.00316.x.	Case
363	Mahadik SP, Pillai A, Joshi S, Foster A. Prevention of oxidative stress-mediated neuropathology and improved clinical outcome by adjunctive use of a combination of antioxidants and omega-3 fatty acids in schizophrenia. Int Rev Psychiatry. 2006;18(2):119-31. doi: 10.1080/09540260600581993.	Review
364	Fredriksson A, Archer T. Subchronic administration of haloperidol influences the functional deficits of postnatal iron administration in mice. Neurotox Res. 2006;9(4):305-12. doi: 10.1007/BF03033321.	Animal
365	Grover S, Avasthi A, Sharma Y. Psychotropics in pregnancy: weighing the risks. Indian J Med Res. 2006;123(4):497-512.	Review
366	Meador KJ, Baker GA, Finnell RH, Kalayjian LA, Liporace JD, Loring DW, Mawer G, Pennell PB, Smith JC, Wolff MC; NEAD Study Group. In utero antiepileptic drug exposure: fetal death and malformations. Neurology. 2006;67(3):407-12. doi: 10.1212/01.wnl.0000227919.81208.b2.	Unfocused
367	Even C, Dorocant ES, Thuile J, Kalck-stern M, Guelfi JD. Grossesse, allaitement et thymorégulateurs: eléments de décisions et règles pour la pratique [Pregnancy, breast feeding and mood stabilisers: review and	Review

	recommendations for practice]. Encéphale. 2006;32(2 Pt 1):224-30. French. doi: 10.1016/s0013-7006(06)76148-6.	
368	Pennell PB. 2005 AES annual course: evidence used to treat women with epilepsy. Epilepsia. 2006;47 Suppl 1:46-53. doi: 10.1111/j.1528-1167.2006.00660.x.	Review
369	Malaspina D. Schizophrenia: a neurodevelopmental or a neurodegenerativedisorder. J Clin Psychiatry. 2006;67(8):e07. doi: 10.4088/jcp.0806e07.	Not in women
370	Kim SW, Kim KM, Kim JM, Shin IS, Shin HY, Yang SJ, Yoon JS. Use of long-acting injectable risperidone before and throughout pregnancy in schizophrenia.Prog Neuropsychopharmacol Biol Psychiatry. 2007;31(2):543-5. doi:10.1016/j.pnpbp.2006.09.017.	Case
371	Pae CU. Potential role of lymphotoxin-alpha (tumor necrosis factor-beta) in the development of schizophrenia. Med Hypotheses. 2007;68(6):1359-62. doi:10.1016/j.mehy.2006.10.023.	Unrelated
372	Ujházy E, Schmidtová M, Dubovický M, Navarova J, Brucknerová I, Mach M. Neurobehavioural changes in rats after neonatal anoxia: effect of antioxidant stobadine pretreatment. Neuro Endocrinol Lett. 2006;27 Suppl 2:82-5.	Animal
373	Harden CL, Leppik I. Optimizing therapy of seizures in women who use oral contraceptives. Neurology. 2006;67(12 Suppl 4):S56-8. doi: 10.1212/wnl.67.12_suppl_4.s56.	Review
374	Ward S, Wisner KL. Collaborative management of women with bipolar disorder during pregnancy and postpartum: pharmacologic considerations. J Midwifery Womens Health. 2007;52(1):3-13. doi: 10.1016/j.jmwh.2006.09.002.	Review
375	Seib FP, Jones AT, Duncan R. Comparison of the endocytic properties of linear and branched PEIs, and cationic PAMAM dendrimers in B16f10 melanoma cells. J Control Release. 2007;117(3):291-300. doi:10.1016/j.jconrel.2006.10.020.	Unrelated
376	Coppola D, Russo LJ, Kwarta RF Jr, Varughese R, Schmider J. Evaluating the postmarketing experience of risperidone use during pregnancy: pregnancy and neonatal outcomes. Drug Saf. 2007;30(3):247-64. doi:10.2165/00002018-200730030-00006.	Review
377	Cunnington M, Ferber S, Quartey G; International Lamotrigine Pregnancy Registry Scientific Advisory Committee. Effect of dose on the frequency of major birth defects following fetal exposure to lamotrigine monotherapy in an international observational study. Epilepsia. 2007;48(6):1207-10. doi:10.1111/j.1528-1167.2007.01021.x.	No antipsychotic
378	Nielsen RE, Stage KB, Christensen PM, Mortensen S, Andersen LL, Damkier P.Medikamentel behandling af depression under graviditet eller amning [Medical treatment of depression during pregnancy and breastfeeding]. Ugeskr Laeger. 2007;169(16):1442-4.	Review
379	Dehn C, Dalhoff KP. Antipsykotika i graviditeten--skades barnet? [Do antipsychotic drugs during pregnancy affect the unborn baby?]. Ugeskr Laeger.2007;169(18):1659-63. Danish.	Review
380	Kłys M, Rojek S, Rzepecka-Woźniak E. Neonatal death following clozapine self-poisoning in late pregnancy: an unusual case report. Forensic Sci Int. 2007;171(1):e5-e10. doi: 10.1016/j.forsciint.2007.04.216.	Case
381	Kurosawa S, Hashimoto E, Ukai W, Toki S, Saito S, Saito T. Olanzapine potentiates neuronal survival and neural stem cell differentiation: regulation of endoplasmic reticulum stress response proteins. J Neural Transm (Vienna).2007;114(9):1121-8. doi: 10.1007/s00702-007-0747-z.	Animal
382	Nordon C, Sutter AL, Verdoux H. Prise en charge des femmes souffrant d'un trouble bipolaire de la conception au post-partum [Management of women with bipolar disorders from conception through the postpartum period]. Presse Med. 2007;36(12 Pt 3):1913-8. French. doi: 10.1016/j.lpm.2007.03.042.	Review
383	Atasoy N, Erdogan A, Yalug I, Ozturk U, Konuk N, Atik L, Ustundag Y. A review of liver function tests during treatment with atypical antipsychotic drugs: a chart review study. Prog Neuropsychopharmacol Biol Psychiatry. 2007;31(6):1255-60. doi: 10.1016/j.pnpbp.2007.05.005.	Review
384	Dayan J, Yoshida K. Thérapeutique des troubles anxieux et dépressifs de la grossesse et du post-partum. Revue et synthèse [Psychological and pharmacological treatments of mood and anxiety disorders during pregnancy and postpartum. Review and synthesis]. J Gynecol Obstet Biol Reprod (Paris). 2007;36(6):530-48. French. doi: 10.1016/j.jgyn.2007.06.004. Epub 2007 Jul 5.	Review
385	Twaites BR, Wilton LV, Shakir SA. The safety of quetiapine: results of a post-marketing surveillance study on 1728 patients in England. J Psychopharmacol. 2007;21(4):392-9. doi: 10.1177/0269881107073257. Erratum in: J Psychopharmacol. 2007;21(7):783. Erratum in: J Psychopharmacol. 2008;22(6):699.	Unfocused
386	Lu Y, Yu Y, Tang X. Sucrose acetate isobutyrate as an in situ forming system for sustained risperidone release.J Pharm Sci. 2007 ;96(12):3252-62.doi: 10.1002/jps.21091.	Unrelated
387	Imamura K, Takeshima T, Nakaso K, Nakashima K. Homocysteine is toxic for dopaminergic neurons in primary mesencephalic culture. Neuroreport. 2007;18(13):1319-22. doi: 10.1097/WNR.0b013e3282aaa0b4.	Animal

388	Cohen LS. Treatment of bipolar disorder during pregnancy. J Clin Psychiatry. 2007;68 Suppl 9:4-9.	Review
389	Shor S, Koren G, Nulman I. Teratogenicity of lamotrigine. Can Fam Physician. 2007;53(6):1007-9.	Case
390	Papazisis G, Kallaras K, Kaiki-Astara A, Pourzitaki C, Tzachanis D, Dagklis T, Kouvelas D. Neuroprotection by lamotrigine in a rat model of neonatal hypoxic-ischaemic encephalopathy. Int J Neuropsychopharmacol. 2008;11(3):321-9. doi: 10.1017/S1461145707008012. Epub 2007 Sep 26.	Animal
391	Prakash, Prabhu LV, Nasar MA, Rai R, Madhyastha S, Singh G. Lamotrigine in pregnancy: safety profile and the risk of malformations. Singapore Med J. 2007;48(10):880-3.	Review
392	Zuo J, Liu Z, Ouyang X, Liu H, Hao Y, Xu L, Lu XH. Distinct neurobehavioral consequences of prenatal exposure to sulpiride (SUL) and risperidone (RIS) in rats. Prog Neuropsychopharmacol Biol Psychiatry. 2008;32(2):387-97. doi:10.1016/j.pnpbp.2007.09.005. Epub 2007 Sep 15.	Animal
393	Pennell PB, Peng L, Newport DJ, Ritchie JC, Koganti A, Holley DK, Newman M,Stowe ZN. Lamotrigine in pregnancy: clearance, therapeutic drug monitoring, and seizure frequency. Neurology. 2008;70(22 Pt 2):2130-6. doi:10.1212/01.wnl.0000289511.20864.2a.	No antipsychotic
394	Manent JB, Jorquera I, Franco V, Ben-Ari Y, Perucca E, Represa A. Antiepileptic drugs and brain maturation: fetal exposure to lamotrigine generates cortical malformations in rats. Epilepsy Res. 2008;78(2-3):131-9. doi: 10.1016/j.eplepsyres.2007.10.014. Epub 2007 Dec 31.	No antipsychotic
395	Donohoe DR, Weeks K, Aamodt EJ, Dwyer DS. Antipsychotic drugs alter neuronal development including ALM neuroblast migration and PLM axonal outgrowth in Caenorhabditis elegans. Int J Dev Neurosci. 2008;26(3-4):371-80. doi: 10.1016/j.ijdevneu.2007.08.021. Epub 2008 Jan 20.	Animal
396	Ramkisson R, Campbell M, Agius M. The clinical dilemma--prescribing in pregnancy. Psychiatr Danub. 2008;20(1):88-90.	Unfocused
397	Chen J, Han M, Manisastry SM, Trotta P, Serrano MC, Huhta JC, Linask KK. Molecular effects of lithium exposure during mouse and chick gastrulation and subsequent valve dysmorphogenesis. Birth Defects Res A Clin Mol Teratol. 2008;82(7):508-18. doi: 10.1002/bdra.20448.	Animal
398	Holmes LB, Baldwin EJ, Smith CR, Habecker E, Glassman L, Wong SL, Wyszynski DF. Increased frequency of isolated cleft palate in infants exposed to lamotrigine during pregnancy. Neurology. 2008;70(22 Pt 2):2152-8. doi: 10.1212/01.wnl.0000304343.45104.d6. Epub 2008 Apr 30. Erratum in: Neurology. 2009;72(16):1449.	No antipsychotic
399	Reis M, Källén B. Maternal use of antipsychotics in early pregnancy and delivery outcome. J Clin Psychopharmacol. 2008;28(3):279-88. doi: 10.1097/JCP.0b013e318172b8d5.	**Included**
400	Boucher N, Bairam A, Beaulac-Baillargeon L. A new look at the neonate's clinical presentation after in utero exposure to antidepressants in late pregnancy. J Clin Psychopharmacol. 2008;28(3):334-9. doi: 10.1097/JCP.0b013e318173aa2e.	No antipsychotic
401	Liy-Salmeron G, Meneses A. Effects of 5-HT drugs in prefrontal cortex during memory formation and the ketamine amnesia-model. Hippocampus.2008;18(9):965-74. doi: 10.1002/hipo.20459.	No antipsychotic
402	Dolk H, Jentink J, Loane M, Morris J, de Jong-van den Berg LT; EUROCAT Antiepileptic Drug Working Group. Does lamotrigine use in pregnancy increase orofacial cleft risk relative to other malformations? Neurology. 2008;71(10):714-22. doi: 10.1212/01.wnl.0000316194.98475.d8. Epub 2008 Jul 23.	No antipsychotic
403	Fiore M, Di Fausto V, Iannitelli A, Aloe L. Clozapine or Haloperidol in rats prenatally exposed to methylazoxymethanol, a compound inducing entorhinal- hippocampal deficits, alter brain and blood neurotrophins' concentrations. Ann Ist Super Sanita. 2008;44(2):167-77.	Animal
404	Dodd S, Berk M. The safety of medications for the treatment of bipolar disorder during pregnancy and the puerperium. Curr Drug Saf. 2006 Jan;1(1):25-33. doi: 10.2174/157488606775252692.	Review
405	Gentile S. Antipsychotic therapy during early and late pregnancy. A systematic review. Schizophr Bull. 2010 May;36(3):518-44. doi: 10.1093/schbul/sbn107. Epub 2008 Sep 11.	Review
406	Meyer U, Spoerri E, Yee BK, Schwarz MJ, Feldon J. Evaluating early preventive antipsychotic and antidepressant drug treatment in an infection-based neurodevelopmental mouse model of schizophrenia. Schizophr Bull. 2010;36(3):607-23. doi: 10.1093/schbul/sbn131. Epub 2008 Oct 8.	Animal
407	Yacobi S, Ornoy A. Is lithium a real teratogen? What can we conclude from the prospective versus retrospective studies? A review. Isr J Psychiatry Relat Sci. 2008;45(2):95-106.	Review
408	Harden CL, Sethi NK. Epileptic disorders in pregnancy: an overview. Curr Opin Obstet Gynecol. 2008;20(6):557-62. doi: 10.1097/GCO.0b013e3283184059.	No antipsychotic
409	Kumar M, Pathak K, Misra A. Formulation and characterization of nanoemulsion-based drug delivery system of risperidone. Drug Dev Ind Pharm. 2009 Apr;35(4):387-95. doi: 10.1080/03639040802363704.	Unfocused
410	Gentile S. Pregnancy exposure to serotonin reuptake inhibitors and the risk of spontaneous abortions. CNS Spectr. 2008;13(11):960-6. doi: 10.1017/s1092852900014012.	No antipsychotic

411	Cetinkaya M, Ozkan H, Köksal N. Unilateral radius aplasia due to lamotrigine and oxcarbazepine use in pregnancy. J Matern Fetal Neonatal Med. 2008;21(12):927-30. doi: 10.1080/14767050802366210.	No antipsychotic
412:	Wichman CL. Atypical antipsychotic use in pregnancy: a retrospective review. Arch Womens Ment Health. 2009;12(1):53-7. doi: 10.1007/s00737-008-0044-3. Epub 2009 Jan 10.	Review
413	Gendron MP, Martin B, Oraichi D, Bérard A. Health care providers' requests to Teratogen Information Services on medication use during pregnancy and lactation. Eur J Clin Pharmacol. 2009;65(5):523-31. doi: 10.1007/s00228-008-0611-6. Epub 2009 Jan 24.	Unfocused
414	Einarson A, Einarson TR. Maternal use of antipsychotics in early pregnancy: little evidence of increased risk of congenital malformations. Evid Based Ment Health. 2009;12(1):29. doi: 10.1136/ebmh.12.1.29.	Opinion
415	Wedzony K, Fijał K, Maćkowiak M, Chocyk A. Detrimental effect of postnatal blockade of N-methyl-D-aspartate receptors on sensorimotor gating is reversed by neuroleptic drugs. Pharmacol Rep. 2008;60(6):856-64.	Animal
416	Straiko MM, Young C, Cattano D, Creeley CE, Wang H, Smith DJ, Johnson SA, Li ES, Olney JW. Lithium protects against anesthesia-induced developmental neuroapoptosis. Anesthesiology. 2009;110(4):862-8. doi: 10.1097/ALN.0b013e31819b5eab.	No antipsychotic
417	Nguyen HT, Sharma V, McIntyre RS. Teratogenesis associated with antibipolar agents. Adv Ther. 2009 Mar;26(3):281-94. doi: 10.1007/s12325-009-0011-z. Epub 2009 Mar 28.	Review
418	López-Fraile IP, Cid AO, Juste AO, Modrego PJ. Levetiracetam plasma level monitoring during pregnancy, delivery, and postpartum: clinical and outcome implications. Epilepsy Behav. 2009;15(3):372-5. doi: 10.1016/j.yebeh.2009.04.006. Epub 2009 May 27.	No antipsychotic
419	Meador KJ, Baker GA, Browning N, Clayton-Smith J, Combs-Cantrell DT, Cohen M, Kalayjian LA, Kanner A, Liporace JD, Pennell PB, Privitera M, Loring DW; NEAD Study Group. Cognitive function at 3 years of age after fetal exposure to antiepileptic drugs. N Engl J Med. 2009 Apr 16;360(16):1597-605. doi: 10.1056/NEJMoa0803531.	No antipsychotic
420	Izumi Y, Watanabe T, Awasaki N, Hikawa K, Minagi T, Chatani F. Collaborative work on evaluation of ovarian toxicity. 16) Effects of 2 or 4 weeks repeated dose studies and fertility study of Chlorpromazine hydrochloride in rats. J Toxicol Sci. 2009;34(Suppl 1):SP167-74. doi: 10.2131/jts.34.s167.	Animal
421	Ishii S, Ube M, Okada M, Adachi T, Sugimoto J, Inoue Y, Uno Y, Mutai M. Collaborative work on evaluation of ovarian toxicity. 17) Two- or four-week repeated-dose studies and fertility study of sulpiride in female rats. J Toxicol Sci. 2009;34(Suppl 1):SP175-88. doi: 10.2131/jts.34.s175.	Animal
422	Broberg BV, Glenthøj BY, Dias R, Larsen DB, Olsen CK. Reversal of cognitive deficits by an ampakine (CX516) and sertindole in two animal models of schizophrenia--sub-chronic and early postnatal PCP treatment in attentional set-shifting. Psychopharmacology (Berl). 2009;206(4):631-40. doi: 10.1007/s00213-009-1540-5. Epub 2009 Apr 24.	Animal
423	Einarson A, Boskovic R. Use and safety of antipsychotic drugs during pregnancy. J Psychiatr Pract. 2009 May;15(3):183-92. doi: 10.1097/01.pra.0000351878.45260.94.	Review
424	Howland RH. Prescribing psychotropic medications during pregnancy and lactation: principles and guidelines. J Psychosoc Nurs Ment Health Serv. 2009;47(5):19-23. doi: 10.3928/02793695-20090331-05.	Review
425	Krüger S. Psychopharmaka in Schwangerschaft und Stillzeit--Risiken und Möglichkeiten bei Frauen mit einer bipolaren affektiven Störung [Bipolar disorder, pregnancy and the postpartum--risks and possibilities of pharmacotherapy]. Ther Umsch. 2009;66(6):475-84. German. doi: 10.1024/0040-5930.66.6.475.	Review
426	Verdoux H, Tournier M, Bégaud B. Pharmacoepidemiology of psychotropic drugs: examples of current research challenges on major public health issues. Epidemiol Psichiatr Soc. 2009;18(2):107-13.	Review
427	Raffo E, de Vasconcelos AP, Boehrer A, Desor D, Nehlig A. Neurobehavioral maturation of offspring from epileptic dams: study in the rat lithium-pilocarpine model. Exp Neurol. 2009;219(2):414-23. doi: 10.1016/j.expneurol.2009.06.014. Epub 2009 Jun 26.	Animal
428	Guillén JM, Company ES. Use of antipsychotics during pregnancy and breastfeeding. Rev Psiquiatr Salud Ment. 2009;2(3):138-45. English, Spanish. doi: 10.1016/S1888-9891(09)72405-X. Epub 2009 Oct 14.	Review
429	Motamedi M, Karvigh SA, Sahraian MA, Azimi AR, Navardi S. Lamotrigine and twin pregnancy, incidental event or possible correlation? Seizure. 2009;18(8):580-2. doi: 10.1016/j.seizure.2009.06.004. Epub 2009 Jul 8.	No antipsychotic
430	Novikova N, Chitnis M, Linder V, Hofmeyr GJ. Atypical antipsychotic (clozapine) self-poisoning in late pregnancy presenting with absent fetal heart rate variability without acidosis and delayed peristalsis in the newborn baby: a case report. Aust N Z J Obstet Gynaecol. 2009 Aug;49(4):442-4. doi: 10.1111/j.1479-828X.2009.01017.x.	Case
431	Montouris G, Abou-Khalil B. The first line of therapy in a girl with juvenile myoclonic epilepsy: should it be valproate or a new agent? Epilepsia. 2009 Sep;50 Suppl 8:16-20. doi: 10.1111/j.1528-1167.2009.02230.x.	Unrelated
432	Nakatani-Pawlak A, Yamaguchi K, Tatsumi Y, Mizoguchi H, Yoneda Y. Neonatal phencyclidine treatment in	Animal

	mice induces behavioral, histological and neurochemical abnormalities in adulthood. Biol Pharm Bull. 2009Sep;32(9):1576-83. doi: 10.1248/bpb.32.1576.	
433	Piontkewitz Y, Assaf Y, Weiner I. Clozapine administration in adolescence prevents postpubertal emergence of brain structural pathology in an animal model of schizophrenia. Biol Psychiatry. 2009;66(11):1038-46. doi: 10.1016/j.biopsych.2009.07.005. Epub 2009 Sep 2.	Animal
434	Vajda FJ, Hitchcock AA, Graham J, O'Brien TJ, Lander CM, Eadie MJ. The teratogenic risk of antiepileptic drug polytherapy. Epilepsia. 2010 May;51(5):805-10. doi: 10.1111/j.1528-1167.2009.02336.x. Epub 2009 Oct 8. PMID: 19817810.	No antipsychotic
435	Berwaerts K, Sienaert P, De Fruyt J. Teratogene effecten van lamotrigine bij vrouwen met een bipolaire stoornis [Teratogenic effects of lamotrigine in women with bipolar disorder]. Tijdschr Psychiatr. 2009;51(10):741-50. Dutch. PMID: 19821242.	No antipsychotic
436	Lu L, Mamiya T, Lu P, Toriumi K, Mouri A, Hiramatsu M, Kim HC, Zou LB, Nagai T, Nabeshima T. Prenatal exposure to phencyclidine produces abnormal behaviour and NMDA receptor expression in postpubertal mice. Int J Neuropsychopharmacol. 2010 Aug;13(7):877-89. doi: 10.1017/S1461145709990757.Epub 2009 Oct 19. PMID: 19835658.	Animal
437	Smith J, Whitehall J. Sodium valproate and the fetus: a case study and review of the literature. Neonatal Netw. 2009 Nov-Dec;28(6):363-7. doi: 10.1891/0730-0832.28.6.363. PMID: 19892633.	Review
438	Valproic acid: long-term effects on children exposed in utero. Prescrire Int. 2009 Dec;18(104):253-7. PMID: 20025093.	No antipsychotic
439	Miskov S, Gjergja-Juraski R, Cvitanović-Sojat L, Bakulić TI, Fucić A, Bosnjak-Pasić M, Mikula I, Demarin V. Prospective surveillance of Croatian pregnant women on lamotrigine monotherapy--aspects of pre-pregnancy counseling and drug monitoring. Acta Clin Croat. 2009 Sep;48(3):271-81. PMID: 20055248.	No antipsychotic
440	van Waarde A, Ramakrishnan NK, Rybczynska AA, Elsinga PH, Ishiwata K, Nijholt IM, Luiten PG, Dierckx RA. The cholinergic system, sigma-1 receptors and cognition. Behav Brain Res. 2011 Aug 10;221(2):543-54. doi: 10.1016/j.bbr.2009.12.043. Epub 2010 Jan 7. PMID: 20060423.	Unfocused
441	Pignatelli AM, Di Fabio F, Ferracuti S, Biondi M. Antipsicotici in gravidanza: alcuni aspetti e problematiche nella scelta del trattamento [Antipsychotics and pregnancy: a clinical case]. Riv Psichiatr. 2009 Sep-Oct;44(5):299-308. Italian. PMID: 20066817.	Case
442	Lohoff FW, Ferraro TN. Pharmacogenetic considerations in the treatment of psychiatric disorders. Expert Opin Pharmacother. 2010 Feb;11(3):423-39. doi: 10.1517/14656560903508762. PMID: 20102306.	Unfocused
443	Fathi-Azarbayjani A, Chan SY. Single and multi-layered nanofibers for rapid and controlled drug delivery. Chem Pharm Bull (Tokyo). 2010 Feb;58(2):143-6. doi: 10.1248/cpb.58.143. PMID: 20118570.	Unfocused
444	Kumar M, Misra A, Pathak K. Formulation and characterization of nanoemulsion of olanzapine for intranasal delivery. PDA J Pharm Sci Technol. 2009 Nov-Dec;63(6):501-11. PMID: 20169856.	Unfocused
445	Mishra AC, Mohanty B. Effects of lactational exposure of olanzapine and risperidone on hematology and lymphoid organs histopathology: a comparative study in mice neonates. Eur J Pharmacol. 2010 May 25;634(1-3):170-7. doi: 10.1016/j.ejphar.2010.02.014. Epub 2010 Feb 20. PMID: 20176014.	Animal
446	Niwa M, Kamiya A, Murai R, Kubo K, Gruber AJ, Tomita K, Lu L, Tomisato S, Jaaro-Peled H, Seshadri S, Hiyama H, Huang B, Kohda K, Noda Y, O'Donnell P, Nakajima K, Sawa A, Nabeshima T. Knockdown of DISC1 by in utero gene transfer disturbs postnatal dopaminergic maturation in the frontal cortex and leads to adult behavioral deficits. Neuron. 2010 Feb 25;65(4):480-9. doi: 10.1016/j.neuron.2010.01.019.	Unrelated
447	Bentué-Ferrer D, Tribut O, Verdier MC; le groupe Suivi Thérapeutique Pharmacologique de la Société Française de Pharmacologie et de Thérapeutique. Suivi thérapeutique pharmacologique de la lamotrigine [Therapeutic drug monitoring of lamotrigine]. Therapie. 2010 Jan-Feb;65(1):39-46. French. doi: 10.2515/therapie/2009063. Epub 2010 Mar 8.	No antipsychotic
448	Francey SM, Nelson B, Thompson A, Parker AG, Kerr M, Macneil C, Fraser R, Hughes F, Crisp K, Harrigan S, Wood SJ, Berk M, McGorry PD. Who needs antipsychotic medication in the earliest stages of psychosis? A reconsideration of benefits, risks, neurobiology and ethics in the era of early intervention. Schizophr Res. 2010 Jun;119(1-3):1-10. doi: 10.1016/j.schres.2010.02.1071. Epub 2010 Mar 26.	Unfocused
449	Keilhoff G, Grecksch G, Becker A. Haloperidol normalized prenatal vitamin D depletion-induced reduction of hippocampal cell proliferation in adult rats. Neurosci Lett. 2010 May 31;476(2):94-8. doi: 10.1016/j.neulet.2010.04.010. Epub 2010 Apr 13.	Animal
450	Piontkewitz Y, Arad M, Weiner I. Risperidone administered during asymptomatic period of adolescence prevents the emergence of brain structural pathology and behavioral abnormalities in an animal model of schizophrenia. Schizophr Bull. 2011 Nov;37(6):1257-69. doi: 10.1093/schbul/sbq040. Epub 2010 May 3.	Animal
451	Diaconu I, Cerullo V, Escutenaire S, Kanerva A, Bauerschmitz GJ, Hernandez-Alcoceba R, Pesonen S, Hemminki A. Human adenovirus replication in immunocompetent Syrian hamsters can be attenuated with chlorpromazine or cidofovir. J Gene Med. 2010;12(5):435-45. doi: 10.1002/jgm.1453.	Unrelated

452	McCauley-Elsom K, Gurvich C, Elsom SJ, Kulkarni J. Antipsychotics in pregnancy. J Psychiatr Ment Health Nurs. 2010 Mar;17(2):97-104. doi: 10.1111/j.1365-2850.2009.01481.x.	Review
453	Gentile S. Neurodevelopmental effects of prenatal exposure to psychotropic medications. Depress Anxiety. 2010 Jul;27(7):675-86. doi: 10.1002/da.20706.	Review
454	Ono T, Hashimoto E, Ukai W, Ishii T, Saito T. The role of neural stem cells for in vitro models of schizophrenia: neuroprotection via Akt/ERK signal regulation. Schizophr Res. 2010 Sep;122(1-3):239-47. doi: 10.1016/j.schres.2010.05.008.	*In vitro*
455	Uehara T, Sumiyoshi T, Seo T, Matsuoka T, Itoh H, Suzuki M, Kurachi M. Neonatal exposure to MK-801, an N-methyl-D-aspartate receptor antagonist, enhances methamphetamine-induced locomotion and disrupts sensorimotor gating in pre- and postpubertal rats. Brain Res. 2010 Sep 17;1352:223-30. doi: 10.1016/j.brainres.2010.07.013. Epub 2010 Jul 13.	Animal
456	Bersudsky Y, Applebaum J, Gaiduk Y, Sharony L, Mishory A, Podberezsky A, Agam G, Belmaker RH. Valnoctamide as a valproate substitute with low teratogenic potential in mania: a double-blind, controlled, add-on clinical trial. Bipolar Disord. 2010 Jun;12(4):376-82. doi: 10.1111/j.1399-5618.2010.00828.x.	No antipsychotic
457	Kacirova I, Grundmann M, Brozmanova H. Serum levels of lamotrigine during delivery in mothers and their infants. Epilepsy Res. 2010 Oct;91(2-3):161-5. doi: 10.1016/j.eplepsyres.2010.07.007. Epub 2010 Aug 7.	No antipsychotic
458	Vajda FJ, Graham JE, Hitchcock AA, O'Brien TJ, Lander CM, Eadie MJ. Is lamotrigine a significant human teratogen? Observations from the Australian Pregnancy Register. Seizure. 2010 Nov;19(9):558-61. doi: 10.1016/j.seizure.2010.07.019. Epub 2010 Aug 24.	No antipsychotic
459	Galbally M, Roberts M, Buist A; Perinatal Psychotropic Review Group. Mood stabilizers in pregnancy: a systematic review. Aust N Z J Psychiatry. 2010 Nov;44(11):967-77. doi: 10.3109/00048674.2010.506637.	No antipsychotic
460	Gilad O, Merlob P, Stahl B, Klinger G. Outcome of infants exposed to olanzapine during breastfeeding. Breastfeed Med. 2011;6(2):55-8. doi: 10.1089/bfm.2010.0027. Epub 2010 Oct 29.	**Included**
461	Madadi P, Ito S. Perinatal exposure to maternal lamotrigine: clinical considerations for the mother and child. Can Fam Physician. 2010 Nov;56(11):1132-4.	No antipsychotic
462	Le Pen G, Jay TM, Krebs MO. Effect of antipsychotics on spontaneous hyperactivity and hypersensitivity to MK-801-induced hyperactivity in rats prenatally exposed to methylazoxymethanol. J Psychopharmacol. 2011;25(6):822-35. doi: 10.1177/0269881110387839. Epub 2010 Nov 18.	Animal
463	Gentile S. Drug treatment for mood disorders in pregnancy. Curr Opin Psychiatry. 2011;24(1):34-40. doi: 10.1097/YCO.0b013e3283413451.	Review
464	Vlasov PN, Dranko DV, Agranovich OV. Ламотриджин в лечении женщин с эпилепсией [Lamotrigine in treatment of women with epilepsy]. Zh Nevrol Psikhiatr Im S S Korsakova. 2011;111(5 Pt 2):38-42. Russian.	No antipsychotic
465	Horvat S, McWhir J, Rozman D. Defects in cholesterol synthesis genes in mouse and in humans: lessons for drug development and safer treatments. Drug Metab Rev. 2011;43(1):69-90. doi: 0.3109/03602532.2010.540580.	Unrelated
466	Prieto MJ, Temprana CF, del Río Zabala NE, Marotta CH, Alonso Sdel V. Optimization and in vitro toxicity evaluation of G4 PAMAM dendrimer-risperidone complexes. Eur J Med Chem. 2011;46(3):845-50. doi: 10.1016/j.ejmech.2010.12.021. Epub 2010 Dec 22.	Unfocused
467	Abel K. Review: teratogenicity of first- and second-generation antipsychotics in pregnancy is unclear. Evid Based Ment Health. 2011;14(1):31. doi: 10.1136/ebmh.14.1.31.	Opinion
468	Wisner KL, Leckman-Westin E, Finnerty M, Essock SM. Valproate prescription prevalence among women of childbearing age. Psychiatr Serv. 2011;62(2):218-20. doi: 10.1176/ps.62.2.pss6202_0218.	No antipsychotic
469	Forcelli PA, Gale K, Kondratyev A. Early postnatal exposure of rats to lamotrigine, but not phenytoin, reduces seizure threshold in adulthood. Epilepsia. 2011;52(4):e20-2. doi: 10.1111/j.1528-1167.2010.02971.x. Epub 2011 Feb 14.	Animal
470	Olczak M, Duszczyk M, Mierzejewski P, Meyza K, Majewska MD. Persistent behavioral impairments and alterations of brain dopamine system after early postnatal administration of thimerosal in rats. Behav Brain Res. 2011;223(1):107-18. doi: 10.1016/j.bbr.2011.04.026. Epub 2011 Apr 28.	Animal
471	Volavka J, Citrome L. Pathways to aggression in schizophrenia affect results of treatment. Schizophr Bull. 2011;37(5):921-9. doi: 10.1093/schbul/sbr041. Epub 2011 May 11.	Unrelated
472	Cunnington MC, Weil JG, Messenheimer JA, Ferber S, Yerby M, Tennis P. Final results from 18 years of the International Lamotrigine Pregnancy Registry. Neurology. 2011;76(21):1817-23. doi: 10.1212/WNL.0b013e31821ccd18.	No antipsychotic
473	Tomson T, Battino D, Bonizzoni E, Craig J, Lindhout D, Sabers A, Perucca E, Vajda F; EURAP study group. Dose-dependent risk of malformations with antiepileptic drugs: an analysis of data from the EURAP epilepsy and pregnancy registry. Lancet Neurol. 2011;10(7):609-17. doi: 10.1016/S1474-4422(11)70107-7. Epub 2011	No antipsychotic

	Jun 5.	
474	Holmes LB, Mittendorf R, Shen A, Smith CR, Hernandez-Diaz S. Fetal effects of anticonvulsant polytherapies: different risks from different drug combinations. Arch Neurol. 2011;68(10):1275-81. doi: 10.1001/archneurol.2011.133. Epub 2011 Jun 13.	No antipsychotic
475	Pilaniya U, Khatri K, Patil UK. Depot based drug delivery system for the management of depression. Curr Drug Deliv. 2011;8(5):483-93. doi: 10.2174/156720111796642309.	No antipsychotic
476	Piontkewitz Y, Arad M, Weiner I. Tracing the development of psychosis and its prevention: what can be learned from animal models. Neuropharmacology. 2012;62(3):1273-89. doi: 10.1016/j.neuropharm.2011.04.019. Epub 2011 Jun 23.	Animal
477	Li C, Liu C, Liu J, Fang L. Correlation between rheological properties, in vitro release, and percutaneous permeation of tetrahydropalmatine. AAPS PharmSciTech. 2011;12(3):1002-10. doi: 10.1208/s12249-011-9664-4. Epub 2011 Aug 2.	Unrelated
478	Goudochnikov VI. The role of glucocorticoids in aging and age-related pharmacotherapy. Adv Gerontol. 2011;24(1):48-53.	No antipsychotic
479	Llorente-Berzal A, Mela V, Borcel E, Valero M, López-Gallardo M, Viveros MP, Marco EM. Neurobehavioral and metabolic long-term consequences of neonatal maternal deprivation stress and adolescent olanzapine treatment in male and female rats. Neuropharmacology. 2012 Mar;62(3):1332-41. doi: 10.1016/j.neuropharm.2011.07.031. Epub 2011 Jul 28.	Animal
480	Nagai T, Kitahara Y, Ibi D, Nabeshima T, Sawa A, Yamada K. Effects of antipsychotics on the behavioral deficits in human dominant-negative DISC1 transgenic mice with neonatal polyI:C treatment. Behav Brain Res. 2011;225(1):305-10. doi: 10.1016/j.bbr.2011.07.049. Epub 2011 Aug 3.	Animal
481	Seju U, Kumar A, Sawant KK. Development and evaluation of olanzapine-loaded PLGA nanoparticles for nose-to-brain delivery: in vitro and in vivo studies. Acta Biomater. 2011;7(12):4169-76. doi: 10.1016/j.actbio.2011.07.025. Epub 2011 Jul 30.	Unfocused
482	Valenti O, Cifelli P, Gill KM, Grace AA. Antipsychotic drugs rapidly induce dopamine neuron depolarization block in a developmental rat model of schizophrenia. J Neurosci. 2011;31(34):12330-8. doi: 10.1523/JNEUROSCI.2808-11.2011.	Animal
483	Galbally M, Snellen M, Lewis AJ. A review of the use of psychotropic medication in pregnancy. Curr Opin Obstet Gynecol. 2011;23(6):408-14. doi: 10.1097/GCO.0b013e32834b92f3.	Review
484	Wierońska JM, Stachowicz K, Acher F, Lech T, Pilc A. Opposing efficacy ofgroup III mGlu receptor activators, LSP1-2111 and AMN082, in animal models of positive symptoms of schizophrenia. Psychopharmacology (Berl). 2012;220(3):481-94. doi: 10.1007/s00213-011-2502-2. Epub 2011 Sep 28.	Animal
485	Nielsen RE. Treatment of psychosis during pregnancy - a case report and amini-review. Acta Neuropsychiatr. 2011;23(5):210-214. doi: 10.1111/j.1601-5215.2011.00590.x.	Case
486	Vajda FJ, Graham J, Roten A, Lander CM, O'Brien TJ, Eadie M. Teratogenicity of the newer antiepileptic drugs--the Australian experience. J Clin Neurosci. 2012;19(1):57-9. doi: 10.1016/j.jocn.2011.08.003. Epub 2011 Nov 21.	No antipsychotic
487	Sabers A. Algorithm for lamotrigine dose adjustment before, during, and after pregnancy. Acta Neurol Scand. 2012;126(1):e1-4. doi: 10.1111/j.1600-0404.2011.01627.x. Epub 2011 Dec 9.	No antipsychotic
488	Piontkewitz Y, Bernstein HG, Dobrowolny H, Bogerts B, Weiner I, Keilhoff G. Effects of risperidone treatment in adolescence on hippocampal neurogenesis, parvalbumin expression, and vascularization following prenatal immune activation in rats. Brain Behav Immun. 2012;26(2):353-63. doi: 10.1016/j.bbi.2011.11.004. Epub 2011 Nov 30.	Animal
489	Hoell I, Amanzada A, Degner D, Havemann-Reinecke U. Therapie von schwangeren Patientinnen mit Abhängigkeit von Opioiden und begleitenden Suchtstoffen. Teil II: Therapie der Komorbiditäten [Pregnant opioid addicted patients and additional drug intake. Part II: Comorbidity and their therapy]. Med Monatsschr Pharm. 2011;34(11):418-25. German.	Unfocused
490	Manakova E, Hubickova L. Antidepressant drug exposure during pregnancy. CZTIS small prospective study. Neuro Endocrinol Lett. 2011;32(Suppl 1):53-6.	No antipsychotic
491	Tauqeer S, Khan RA, Siddiqui AA. Evaluation of teratogenic effects of risperidone following simultaneous administration with antihypertensive and antiemetic drugs. Pak J Pharm Sci. 2012;25(1):261-6.	Animal
492	Nawa H, Yamada K. Experimental schizophrenia models in rodents established with inflammatory agents and cytokines. Methods Mol Biol. 2012;829:445-51. doi: 10.1007/978-1-61779-458-2_28.	Animal
493	McKnight RF, Adida M, Budge K, Stockton S, Goodwin GM, Geddes JR. Lithium toxicity profile: a systematic review and meta-analysis. Lancet. 2012;379(9817):721-8. doi: 10.1016/S0140-6736(11)61516-X. Epub 2012 Jan 20.	Review
494	Singh J, Michel D, Chitanda JM, Verrall RE, Badea I. Evaluation of cellular uptake and intracellular trafficking	Unrelated

	as determining factors of gene expression for amino acid-substituted gemini surfactant-based DNA nanoparticles. J Nanobiotechnology. 2012;10:7. doi: 10.1186/1477-3155-10-7.	
495	Lim AL, Taylor DA, Malone DT. A two-hit model: behavioural investigation of the effect of combined neonatal MK-801 administration and isolation rearing in the rat. J Psychopharmacol. 2012;26(9):1252-64. doi: 10.1177/0269881111430751. Epub 2012 Feb 23.	Animal
496	Gentile S. Lithium in pregnancy: the need to treat, the duty to ensure safety. Expert Opin Drug Saf. 2012;11(3):425-37. doi: 10.1517/14740338.2012.670419. Epub 2012 Mar 9.	No antipsychotic
497	Oyebode F, Rastogi A, Berrisford G, Coccia F. Psychotropics in pregnancy: safety and other considerations. Pharmacol Ther. 2012;135(1):71-7. doi: 10.1016/j.pharmthera.2012.03.008. Epub 2012 Mar 28.	Review
498	Jentink J, Boersma C, de Jong-van den Berg LT, Postma MJ. Economic evaluation of anti-epileptic drug therapies with specific focus on teratogenic outcomes. J Med Econ. 2012;15(5):862-8. doi: 10.3111/13696998.2012.684366. Epub 2012 May 3.	No antipsychotic
499	Matrisciano F, Tueting P, Dalal I, Kadriu B, Grayson DR, Davis JM, Nicoletti F, Guidotti A. Epigenetic modifications of GABAergic interneurons are associated with the schizophrenia-like phenotype induced by prenatal stress in mice. Neuropharmacology. 2013;68:184-94. doi:10.1016/j.neuropharm.2012.04.013. Epub 2012 Apr 28.	Animal
500	Moore JL, Aggarwal P. Lamotrigine use in pregnancy. Expert Opin Pharmacother. 2012;13(8):1213-6. doi: 10.1517/14656566.2012.665875.	Opinion
501	Zmarowski A, Beekhuijzen M, Lensen J, Emmen H. Differential performance of Wistar Han and Sprague Dawley rats in behavioral tests: differences in baseline behavior and reactivity to positive control agents. Reprod Toxicol. 2012;34(2):192-203. doi: 10.1016/j.reprotox.2012.05.091. Epub 2012 Jun 1.	Animal
502	Holmes LB, Hernandez-Diaz S. Newer anticonvulsants: lamotrigine, topiramate and gabapentin. Birth Defects Res A Clin Mol Teratol. 2012;94(8):599-606. doi: 10.1002/bdra.23028. Epub 2012 Jun 22.	No antipsychotic
503	Bahadur S, Pathak K. Buffered nanoemulsion for nose to brain delivery of ziprasidone hydrochloride: preformulation and pharmacodynamic evaluation. Curr Drug Deliv. 2012;9(6):596-607. doi: 10.2174/156720112803529792.	Unfocused
504	Goldstein N, Goldstein R, Terterov D, Kamensky AA, Kovalev GI, Zolotarev YA, Avakyan GN, Terterov S. Blood-brain barrier unlocked. Biochemistry (Mosc). 2012;77(5):419-24. doi: 10.1134/S000629791205001X.	Unrelated
505	Raha S, Taylor VH, Holloway AC. Effect of atypical antipsychotics on fetal growth: is the placenta involved? J Pregnancy. 2012;2012:315203. doi: 10.1155/2012/315203. Epub 2012 Jul 11.	Review
506	Krüger S. Psychopharmacological treatment of mood and anxiety disorders during pregnancy. Handb Exp Pharmacol. 2012;(214):279-305. doi: 10.1007/978-3-642-30726-3_14.	No antipsychotic
507	Vajda FJ, Dodd S, Horgan D. Lamotrigine in epilepsy, pregnancy and psychiatry--a drug for all seasons? J Clin Neurosci. 2013;20(1):13-6. doi: 10.1016/j.jocn.2012.05.024. Epub 2012 Oct 1.	No antipsychotic
508	Belujon P, Patton MH, Grace AA. Disruption of prefrontal cortical- hippocampal balance in a developmental model of schizophrenia: reversal by sulpiride. Int J Neuropsychopharmacol. 2013;16(3):507-12. doi: 10.1017/S146114571200106X. Epub 2012 Oct 16.	Unrelated
509	Bodén R, Lundgren M, Brandt L, Reutfors J, Andersen M, Kieler H. Risks of adverse pregnancy and birth outcomes in women treated or not treated with mood stabilisers for bipolar disorder: population based cohort study. BMJ. 2012;345:e7085. doi: 10.1136/bmj.e7085.	No antipsychotic
510	Gentile S. Bipolar disorder in pregnancy: to treat or not to treat? BMJ. 2012;345:e7367. doi: 10.1136/bmj.e7367.	No antipsychotic
511	Livio F, Renard D, Buclin T. Pharmacovigilance [Pharmacovigilance]. Rev Méd Suisse. 2012;8(324):116-9. French.	Review
512	Perez SM, Shah A, Asher A, Lodge DJ. Hippocampal deep brain stimulation reverses physiological and behavioural deficits in a rodent model of schizophrenia. Int J Neuropsychopharmacol. 2013;16(6):1331-9. doi: 10.1017/S1461145712001344. Epub 2012 Nov 28.	Animal
513	Belujon P, Patton MH, Grace AA. Role of the prefrontal cortex in altered hippocampal-accumbens synaptic plasticity in a developmental animal model of schizophrenia. Cereb Cortex. 2014;24(4):968-77. doi: 10.1093/cercor/bhs380. Epub 2012 Dec 12.	Animal
514	Koo J, Zavras A. Antiepileptic drugs (AEDs) during pregnancy and risk of congenital jaw and oral malformation. Oral Dis. 2013;19(7):712-20. doi: 10.1111/odi.12061. Epub 2013 Jan 11.	No antipsychotic
515	Barbui C, Conti V, Purgato M, Cipriani A, Fortino I, Rivolta AL, Lora A. Use of antipsychotic drugs and mood stabilizers in women of childbearing age with schizophrenia and bipolar disorder: epidemiological survey. Epidemiol Psychiatr Sci. 2013;22(4):355-61. doi: 10.1017/S2045796013000012. Epub 2013 Feb 1.	No risk assessed

516	Basta-Kaim A, Szczęsny E, Leśkiewicz M, Głombik K, Slusarczyk J, Budziszewska B, Regulska M, Kubera M, Nowak W, Wędzony K, Lasoń W. Maternal immune activation leads to age-related behavioral and immunological changes in male rat offspring – the effect of antipsychotic drugs. Pharmacol Rep. 2012;64(6):1400-10. doi: 10.1016/s1734-1140(12)70937-4.	Animal
517	Wilson KL, Alexander JM. Seizures and intracranial hemorrhage. Obstet Gynecol Clin North Am. 2013;40(1):103-20. doi: 10.1016/j.ogc.2012.11.009.	Unrelated
518	Peng L, Zhu D, Feng X, Dong H, Yue Q, Zhang J, Gao Q, Hao J, Zhang X, Liu Z, Sun J. Paliperidone protects prefrontal cortical neurons from damages caused by MK-801 via Akt1/GSK3β signaling pathway. Schizophr Res. 2013;147(1):14-23. doi: 10.1016/j.schres.2013.03.006. Epub 2013 Apr 9.	Unrelated
519	Ahmad-Sabry MH, Shareghi G. Long-term use of intrathecal droperidol as an excellent antiemetic in nonmalignant pain--a retrospective study. Middle East J Anaesthesiol. 2012;21(6):857-62.	Unfocused
520	Wang M, Zhang Y, Feng J, Gu T, Dong Q, Yang X, Sun Y, Wu Y, Chen Y, Kong W. Preparation, characterization, and in vitro and in vivo investigation of chitosan-coated poly (d,l-lactide-co-glycolide) nanoparticles for intestinal delivery of exendin-4. Int J Nanomedicine. 2013;8:1141-54. doi: 10.2147/IJN.S41457. Epub 2013 Mar 15.	Unfocused
521	Abdelbary GA, Tadros MI. Brain targeting of olanzapine via intranasal delivery of core-shell difunctional block copolymer mixed nanomicellar carriers: in vitro characterization, ex vivo estimation of nasal toxicity and in vivo biodistribution studies. Int J Pharm. 2013;452(1-2):300-10. doi: 10.1016/j.ijpharm.2013.04.084. Epub 2013 May 14.	Unrelated
522	Habermann F, Fritzsche J, Fuhlbrück F, Wacker E, Allignol A, Weber-Schoendorfer C, Meister R, Schaefer C. Atypical antipsychotic drugs and pregnancy outcome: a prospective, cohort study. J Clin Psychopharmacol. 2013;33(4):453-62. doi: 10.1097/JCP.0b013e318295fe12.	**Included**
523	Sadowski A, Todorow M, Yazdani Brojeni P, Koren G, Nulman I. Pregnancy outcomes following maternal exposure to second-generation antipsychotics given with other psychotropic drugs: a cohort study. BMJ Open. 2013;3(7):e003062. doi: 10.1136/bmjopen-2013-003062.	**Included**
524	Kennedy D, Eamus M, Hill M, Oei JL. Review of calls to an Australian teratogen information service regarding psychotropic medications over a 12-year period. Aust N Z J Obstet Gynaecol. 2013;53(6):544-52. doi: 10.1111/ajo.12129. Epub 2013 Sep 13.	Review
525	Rapanelli M, Frick LR, Bernardez-Vidal M, Zanutto BS. Different MK-801 administration schedules induce mild to severe learning impairments in an operant conditioning task: role of buspirone and risperidone in ameliorating these cognitive deficits. Behav Brain Res. 2013;257:156-65. doi: 10.1016/j.bbr.2013.09.043. Epub 2013 Oct 1.	Unrelated
526	Singh KP, Tripathi N. Prenatal exposure of a novel antipsychotic aripiprazole: impact on maternal, fetal and postnatal body weight modulation in rats. Curr Drug Saf. 2014;9(1):43-8. doi: 0.2174/1574886311308666006l.	Animal
527	Furukawa S, Hayashi S, Abe M, Hagio S, Irie K, Kuroda Y, Ogawa I, Sugiyama A. Effect of chlorpromazine on rat placenta development. Exp Toxicol Pathol. 2014;66(1):41-7. doi: 10.1016/j.etp.2013.08.002. Epub 2013 Oct 16.	Animal
528	Pearlstein T. Use of psychotropic medication during pregnancy and the postpartum period. Womens Health (Lond). 2013;9(6):605-15. doi: 10.2217/whe.13.54.	Review
529	Harms CA, McLellan WA, Moore MJ, Barco SG, Clarke EO 3rd, Thayer VG, Rowles TK. Low-residue euthanasia of stranded mysticetes. J Wildl Dis. 2014;50(1):63-73. doi: 10.7589/2013-03-074. Epub 2013 Oct 25.	Unfocused
530	Lee SH, Kang JW, Lin T, Lee JE, Jin DI. Teratogenic potential of antiepileptic drugs in the zebrafish model. Biomed Res Int. 2013;2013:726478. doi: 10.1155/2013/726478. Epub 2013 Nov 14.	Animal
531	Takcı S, Bayhan C, Celik T, Yiğit S. Hypotonia and poor feeding in an infant exposed to lamotrigine and valproic acid in utero. Turk J Pediatr. 2013;55(5):546-8.	No antipsychotic
532	Campbell E, Kennedy F, Russell A, Smithson WH, Parsons L, Morrison PJ, Liggan B, Irwin B, Delanty N, Hunt SJ, Craig J, Morrow J. Malformation risks of antiepileptic drug monotherapies in pregnancy: updated results from the UK and Ireland Epilepsy and Pregnancy Registers. J Neurol Neurosurg Psychiatry. 2014;85(9):1029-34. doi: 10.1136/jnnp-2013-306318. Epub 2014 Jan 20.	No antipsychotic
533	Christofaki M, Papaioannou A. Ondansetron: a review of pharmacokinetics and clinical experience in postoperative nausea and vomiting. Expert Opin Drug Metab Toxicol. 2014;10(3):437-44. doi: 10.1517/17425255.2014.882317. Epub 2014 Jan 28.	Unrelated
534	Webster WS, Nilsson M, Ritchie H. Therapeutic drugs that slow the heartrate of early rat embryos. Is there a risk for the human? Curr Pharm Des. 2014;20(34):5364-76. doi: 10.2174/1381612820666140205151146.	Animal
535	Pottegård A, Hallas J, Andersen JT, Løkkegaard EC, Dideriksen D, Aagaard L, Damkier P. First-trimester exposure to methylphenidate: a population-based cohort study. J Clin Psychiatry. 2014;75(1):e88-93. doi: 10.4088/JCP.13m08708.	No antipsychotic

536	Choi L, Joo SH, Jeong JH. Olanzapine use in a manic patient during second and third trimester pregnancy. Neuropsychiatr Dis Treat. 2014;10:325-8. doi: 10.2147/NDT.S59481.	Case
537	Moreno E, Moreno-Delgado D, Navarro G, Hoffmann HM, Fuentes S, Rosell-Vilar S, Gasperini P, Rodríguez-Ruiz M, Medrano M, Mallol J, Cortés A, Casadó V, Lluís C, Ferré S, Ortiz J, Canela E, McCormick PJ. Cocaine disrupts histamine H3 receptor modulation of dopamine D1 receptor signaling: σ1-D1-H3 receptor complexes as key targets for reducing cocaine's effects. J Neurosci. 2014;34(10):3545-58. doi: 10.1523/JNEUROSCI.4147-13.2014.	Unrelated
538	Akar M, Kasapkara ÇS, Özbek MN, Tüzün H, Aldudak B, Kanar B. Transient nephrogenic diabetes insipidus caused by fetal exposure to haloperidol. Ren Fail. 2014;36(6):951-2. doi: 10.3109/0886022X.2014.900403. Epub 2014 Mar 27.	Case
539	Cantilino A, Lorenzo L, Paula Jdos A, Einarson A. Use of psychotropic medications during pregnancy: perception of teratogenic risk among physicians in two Latin American countries. Braz J Psychiatry. 2014;36(2):106-10. doi: 10.1590/1516-4446-2013-1221. Epub 2014 Mar 24.	Unfocused
540	Prakash S, Chadda RK. Teratogenicity with olanzapine. Indian J Psychol Med. 2014;36(1):91-3. doi: 10.4103/0253-7176.127266.	Case
541	Zhang J, Ye L, Wang W, Du G, Yu X, Zhu X, Dong Q, Cen X, Guan X, Fu F, Tian J. A 12-week subchronic intramuscular toxicity study of risperidone-loaded microspheres in rats. Hum Exp Toxicol. 2015;34(2):205-23. doi: 10.1177/0960327114532380. Epub 2014 May 8.	Animal
542	Ajazuddin, Alexander A, Qureshi A, Kumari L, Vaishnav P, Sharma M, Saraf S, Saraf S. Role of herbal bioactives as a potential bioavailability enhancer for Active Pharmaceutical Ingredients. Fitoterapia. 2014;97:1-14. doi: 10.1016/j.fitote.2014.05.005. Epub 2014 May 23.	Unrelated
543	Im W, Kim M. Cell therapy strategies vs. paracrine effect in Huntington's disease. J Mov Disord. 2014;7(1):1-6. doi: 10.14802/jmd.14001. Epub 2014 Apr 30.	Unrelated
544	Drozdowicz LB, Bostwick JM. Psychiatric adverse effects of pediatric corticosteroid use. Mayo Clin Proc. 2014;89(6):817-34. doi: 10.1016/j.mayocp.2014.01.010.	Unrelated
545	Windhager E, Kim SW, Saria A, Zauner K, Amminger PG, Klier CM. Perinatal use of aripiprazole: plasma levels, placental transfer, and child outcome in 3 new cases. J Clin Psychopharmacol. 2014;34(5):637-41. doi: 10.1097/JCP.0000000000000171.	Unrelated
546	Sinclair S, Cunnington M, Messenheimer J, Weil J, Cragan J, Lowensohn R, Yerby M, Tennis P. Advantages and problems with pregnancy registries: observations and surprises throughout the life of the International Lamotrigine Pregnancy Registry. Pharmacoepidemiol Drug Saf. 2014;23(8):779-86. doi: 10.1002/pds.3659. Epub 2014 Jun 27.	No antipsychotic
547	Vajda FJ, O'Brien TJ, Lander CM, Graham J, Eadie MJ. The teratogenicity of the newer antiepileptic drugs - an update. Acta Neurol Scand. 2014;130(4):234-8. doi: 10.1111/ane.12280. Epub 2014 Jul 18.	No antipsychotic
548	Galbally M, Snellen M, Power J. Antipsychotic drugs in pregnancy: a review of their maternal and fetal effects. Ther Adv Drug Saf. 2014;5(2):100-9. doi: 10.1177/2042098614522682.	Review
549	Shu Q, Qin R, Chen Y, Hu G, Li M. Asenapine sensitization from adolescence to adulthood and its potential molecular basis. Behav Brain Res. 2014;273:166-76. doi: 10.1016/j.bbr.2014.07.042. Epub 2014 Aug 2. PMID: 25093543; PMCID: PMC4154364.	Animal
550	Gentile S. A safety evaluation of aripiprazole for treating schizophrenia during pregnancy and puerperium. Expert Opin Drug Saf. 2014;13(12):1733-42. doi: 10.1517/14740338.2014.951325. Epub 2014 Aug 19.	Review
551	Gentile S. Pregnancy exposure to second-generation antipsychotics and the risk of gestational diabetes. Expert Opin Drug Saf. 2014;13(12):1583-90. doi: 10.1517/14740338.2014.931368. Epub 2014 Sep 5.	Review
552	Ramey P, Osborn M, Abou-Khalil B. Conversion from immediate-release to extended-release lamotrigine improves seizure control. Epilepsy Res. 2014;108(9):1637-41. doi: 10.1016/j.eplepsyres.2014.08.004. Epub 2014 Aug 27.	Unrelated
553	Kumar U, Mohanty B. Atypical antipsychotic paliperidone prevents behavioral deficits in mice prenatally challenged with bacterial endotoxin lipopolysaccharide. Eur J Pharmacol. 2015;747:181-9. doi: 10.1016/j.ejphar.2014.09.011. Epub 2014 Sep 21.	Unrelated
554	Ifteni P, Moga MA, Burtea V, Correll CU. Schizophrenia relapse after stopping olanzapine treatment during pregnancy: a case report. Ther Clin Risk Manag. 2014;10:901-4. doi: 10.2147/TCRM.S70545.	Case
555	Chou S, Davis C, Jones S, Li M. Repeated effects of the neurotensin receptor agonist PD149163 in three animal tests of antipsychotic activity: assessing for tolerance and cross-tolerance to clozapine. Pharmacol Biochem Behav. 2015;128:78-88. doi: 10.1016/j.pbb.2014.11.015. Epub 2014 Nov 26.	Unrelated
556	Lilienthal H, Korkalainen M, Andersson PL, Viluksela M. Developmental exposure to purity-controlled polychlorinated biphenyl congeners (PCB74 and PCB95) in rats: effects on brainstem auditory evoked potentials and catalepsy. Toxicology. 2015 Jan 2;327:22-31. doi: 10.1016/j.tox.2014.11.004. Epub 2014 Nov 13. PMID:	Animal

	25449634.	
557	Morgan AP, Crowley JJ, Nonneman RJ, Quackenbush CR, Miller CN, Ryan AK, Bogue MA, Paredes SH, Yourstone S, Carroll IM, Kawula TH, Bower MA, Sartor RB, Sullivan PF. The antipsychotic olanzapine interacts with the gut microbiome to cause weight gain in mouse. PLoS One. 2014;9(12):e115225. doi: 10.1371/journal.pone.0115225.	Animal
558	Ito S. Chronic illness and the breastfeeding mother. J Popul Ther Clin Pharmacol. 2014;21(3):e565-8. Epub 2014 Dec 11.	Unrelated
559	Ennis ZN, Damkier P. Pregnancy exposure to olanzapine, quetiapine, risperidone, aripiprazole and risk of congenital malformations. A systematic review. Basic Clin Pharmacol Toxicol. 2015;116(4):315-20. doi: 10.1111/bcpt.12372. Epub 2015 Jan 28.	Review
560	Epstein RA, Moore KM, Bobo WV. Treatment of bipolar disorders during pregnancy: maternal and fetal safety and challenges. Drug Healthc Patient Saf. 2014;7:7-29. doi: 10.2147/DHPS.S50556.	Review
561	Shirasaka T, Kurosawa S. 精神疾患に対する経静脈的神経幹細胞移植療法を用いた新しい治療法の可能性：神経ネットワーク修復と行動改善促進のための戦略 [Potential therapy of intravenous neural stem cell transplantation for psychiatric disorder--a strategy for facilitation of neural network and behavioral recovery]. Nihon Arukoru Yakubutsu Igakkai Zasshi. 2014;49(5):259-69. Japanese.	Unrelated
562	Bellet F, Beyens MN, Bernard N, Beghin D, Elefant E, Vial T. Exposure to aripiprazole during embryogenesis: a prospective multicenter cohort study. Pharmacoepidemiol Drug Saf. 2015;24(4):368-80. doi: 10.1002/pds.3749. Epub 2015 Feb 12.	**Included**
563	Singh KP, Tripathi N. Prenatal exposure to a novel antipsychotic quetiapine: impact on neuro-architecture, apoptotic neurodegeneration in fetal hippocampus and cognitive impairment in young rats. Int J Dev Neurosci. 2015;42:59-67. doi: 10.1016/j.ijdevneu.2015.02.011. Epub 2015 Feb 23.	Animal
564	Bellantuono C, Di Massimo G, Mauro A, Martellini M, Nardi B. Aripiprazolo in gravidanza: una rassegna della letteratura internazionale [Aripiprazole in pregnancy: a review of literature]. Riv Psichiatr. 2015;50(1):8-11. Italian. doi: 10.1708/1794.19526.	Review
565	Goda SA, Olszewski M, Piasecka J, Rejniak K, Whittington MA, Kasicki S, Hunt MJ. Aberrant high frequency oscillations recorded in the rat nucleus accumbens in the methylazoxymethanol acetate neurodevelopmental model of schizophrenia. Prog Neuropsychopharmacol Biol Psychiatry. 2015;61:44-51. doi: 10.1016/j.pnpbp.2015.03.016. Epub 2015 Apr 7.	Unrelated
566	Coughlin CG, Blackwell KA, Bartley C, Hay M, Yonkers KA, Bloch MH. Obstetric and neonatal outcomes after antipsychotic medication exposure in pregnancy. Obstet Gynecol. 2015;125(5):1224-1235. doi: 10.1097/AOG.0000000000000759.	Review
567	Cohen LS, Viguera AC, McInerney KA, Kwiatkowski MA, Murphy SK, Lemon EL, Hernández-Díaz S. Establishment of the National Pregnancy Registry for Atypical Antipsychotics. J Clin Psychiatry. 2015;76(7):986-9. doi: 10.4088/JCP.14br09418.	Unfocused
568	Ankolekar SM, Sikdar SK. Early postnatal exposure to lithium in vitro induces changes in AMPAR mEPSCs and vesicular recycling at hippocampal glutamatergic synapses. J Biosci. 2015;40(2):339-54. doi: 10.1007/s12038-015-9527-3.	No antipsychotic
569	Kulkarni J, Storch A, Baraniuk A, Gilbert H, Gavrilidis E, Worsley R. Antipsychotic use in pregnancy. Expert Opin Pharmacother. 2015;16(9):1335-45. doi: 10.1517/14656566.2015.1041501.	Review
570	Chou S, Jones S, Li M. Adolescent olanzapine sensitization is correlated with hippocampal stem cell proliferation in a maternal immune activation rat model of schizophrenia. Brain Res. 2015;1618:122-35. doi: 10.1016/j.brainres.2015.05.036. Epub 2015 Jun 3.	Animal
571	Tomson T, Battino D, Bonizzoni E, Craig J, Lindhout D, Perucca E, Sabers A, Thomas SV, Vajda F; EURAP Study Group. Dose-dependent teratogenicity of valproate in mono- and polytherapy: an observational study. Neurology. 2015;85(10):866-72. doi: 10.1212/WNL.0000000000001772. Epub 2015 Jun 17.	No antipsychotic
572	Siafaka PI, Barmpalexis P, Lazaridou M, Papageorgiou GZ, Koutris E, Karavas E, Kostoglou M, Bikiaris DN. Controlled release formulations of risperidone antipsychotic drug in novel aliphatic polyester carriers: Data analysis and modelling. Eur J Pharm Biopharm. 2015;94:473-84. doi: 10.1016/j.ejpb.2015.06.027. Epub 2015 Jul 6.	Unrelated
573	Ma L, Yang F, Zhao R, Li L, Kang X, Xiao L, Jiang W. Quetiapine attenuates cognitive impairment and decreases seizure susceptibility possibly through promoting myelin development in a rat model of malformations of cortical development. Brain Res. 2015;1622:443-51. doi: 10.1016/j.brainres.2015.07.012. Epub 2015 Jul 16.	Animal
574	Furukawa S, Tsuji N, Hayashi S, Abe M, Hagio S, Yamagishi Y, Kuroda Y, Sugiyama A. Histomorphological comparison of rat placentas by different timing of chlorpromazine-administration. Exp Toxicol Pathol. 2015 Sep;67(9):443-52. doi: 10.1016/j.etp.2015.06.001. Epub 2015 Jul 18.	Animal
575	Terrana N, Koren G, Pivovarov J, Etwel F, Nulman I. Pregnancy outcomes following in utero exposure to	Review

	second-generation antipsychotics: A systematic review and meta-analysis. J Clin Psychopharmacol. 2015;35(5):559-65. doi: 10.1097/JCP.0000000000000391.	
576	Wlodarczyk BJ, Ogle K, Lin LY, Bialer M, Finnell RH. Comparative teratogenicity analysis of valnoctamide, risperidone, and olanzapine in mice. Bipolar Disord. 2015;17(6):615-25. doi: 10.1111/bdi.12325. Epub 2015 Aug 20.	Animal
577	Babu GN, Desai G, Chandra PS. Antipsychotics in pregnancy and lactation. Indian J Psychiatry. 2015;57(Suppl 2):S303-7. doi: 10.4103/0019-5545.161497.	Review
578	Grover S, Avasthi A. Mood stabilizers in pregnancy and lactation. Indian J Psychiatry. 2015;57(Suppl 2):S308-23. doi: 10.4103/0019-5545.161498.	No antipsychotic
579	Igartúa DE, Calienni MN, Feas DA, Chiaramoni NS, Del Valle Alonso S, Prieto MJ. Development of nutraceutical emulsions as risperidone delivery systems: Characterization and toxicological studies. J Pharm Sci. 2015;104(12):4142-4152. doi: 10.1002/jps.24636. Epub 2015 Sep 11.	Unrelated
580	Neville AJ, Zach SJ, Wang X, Larson JJ, Judge AK, Davis LA, Vennerstrom JL, Davis PH. Clinically available medicines demonstrating anti-toxoplasma activity. Antimicrob Agents Chemother. 2015;59(12):7161-9. doi: 10.1128/AAC.02009-15. Epub 2015 Sep 21.	Unrelated
581	Ritchie H, Oakes D, Hung TT, Hegedus E, Sood S, Webster W. The effect of dofetilide on the heart rate of GD11 and GD13 rat embryos, in vivo, using ultrasound. Birth Defects Res B Dev Reprod Toxicol. 2015;104(5):196-203. doi: 10.1002/bdrb.21162. Epub 2015 Sep 24.	No antipsychotic
582	Degirmencioglu H, Sari FN, Alyamac Dizdar E, Say B, Altug N, Uras N, Canpolat FE, Oguz SS. Coexistence of fetal cardiac malformation and maternal drug-induced lupus: Is lamotrigine safe? Am J Ther. 2016;23(5):e1263-5. doi: 10.1097/MJT.0000000000000324.	No antipsychotic
583	Cohen LS, Viguera AC, McInerney KA, Freeman MP, Sosinsky AZ, Moustafa D, Marfurt SP, Kwiatkowski MA, Murphy SK, Farrell AM, Chitayat D, Hernández-Díaz S. Reproductive safety of second-generation antipsychotics: Current data from the Massachusetts General Hospital National Pregnancy Registry for Atypical Antipsychotics. Am J Psychiatry. 2016;173(3):263-70. doi: 10.1176/appi.ajp.2015.15040506. Epub 2015 Oct 6.	**Included**
584	Zitko J, Dolezal M. Indole-2-carboxamide derivatives: a patent evaluation of WO2015036412A1. Expert Opin Ther Pat. 2015;25(12):1487-94. doi: 10.1517/13543776.2015.1101066. Epub 2015 Nov 4.	Unrelated
585	Haldeman-Englert CR, Jewett T. 1q21.1 Recurrent Microdeletion. 2011 Feb 24 [updated 2015 Nov 12]. In: Adam MP, Mirzaa GM, Pagon RA, Wallace SE, Bean LJH, Gripp KW, Amemiya A, editors. GeneReviews®. Seattle (WA): University of Washington, Seattle; 1993–2023.	Review
586	Qu Z, Zhang J, Yang H, Huo L, Gao J, Chen H, Gao W. Protective effect of tetrahydropalmatine against d-galactose induced memory impairment in rat. Physiol Behav. 2016;154:114-25. doi: 10.1016/j.physbeh.2015.11.016. Epub 2015 Nov 22.	Unrelated
587	Wichman CL. Managing Your Own Mood Lability: Use of Mood Stabilizers and Antipsychotics in Pregnancy. Curr Psychiatry Rep. 2016;18(1):1. doi: 10.1007/s11920-015-0646-1.	Review
588	Deslauriers J, Belleville K, Beaudet N, Sarret P, Grignon S. A two-hit model of suicide-trait-related behaviors in the context of a schizophrenia-like phenotype: Distinct effects of lithium chloride and clozapine. Physiol Behav. 2016;156:48-58. doi: 10.1016/j.physbeh.2016.01.002. Epub 2016 Jan 6.	Unrelated
589	Khan SJ, Fersh ME, Ernst C, Klipstein K, Albertini ES, Lusskin SI. Bipolar Disorder in Pregnancy and Postpartum: Principles of Management. Curr Psychiatry Rep. 2016;18(2):13. doi: 10.1007/s11920-015-0658-x.	Review
590	Deshmukh U, Adams J, Macklin EA, Dhillon R, McCarthy KD, Dworetzky B, Klein A, Holmes LB. Behavioral outcomes in children exposed prenatally to lamotrigine, valproate, or carbamazepine. Neurotoxicol Teratol. 2016;54:5-14. doi: 10.1016/j.ntt.2016.01.001. Epub 2016 Jan 12.	No antipsychotic
591	Wald MF, Muzyk AJ, Clark D. Bipolar Depression: Pregnancy, Postpartum, and Lactation. Psychiatr Clin North Am. 2016;39(1):57-74. doi: 10.1016/j.psc.2015.10.002.	Unfocused
592	Bonini SA, Mastinu A, Maccarinelli G, Mitola S, Premoli M, La Rosa LR,Ferrari-Toninelli G, Grilli M, Memo M. Cortical structure alterations and social behavior impairment in p50-deficient mice. Cereb Cortex. 2016 Jun;26(6):2832-49. doi: 10.1093/cercor/bhw037. Epub 2016 Mar 5. PMID: 26946128; PMCID: PMC4869818.	Animal
593	Montastruc F, Salvo F, Arnaud M, Bégaud B, Pariente A. Signal of gastrointestinal congenital malformations with antipsychotics after minimising competition bias: A disproportionality analysis using data from Vigibase(®). Drug Saf. 2016;39(7):689-96. doi: 10.1007/s40264-016-0413-1.	**Included**
594	Pervaiz F, Ahmad M, Hussain T, Idrees A, Yaqoob A, Abbas K. Development of olanzapine loaded PNA microgels for depot drug delivery in treatment of schizophrenia: in vitro an in vivo release profile. Acta Pol Pharm. 2016;73(1):175-81.	Unrelated
595	Ramey P, Osborn MR, Lowen KM, Reed RC, Abou-Khalil B. Unexplained spikes in lamotrigine serum concentration: nonlinear elimination? Acta Neurol Scand. 2017;135(2):240-246. doi: 10.1111/ane.12588. Epub 2016 Mar 31.	Unrelated

596	Petersen I, McCrea RL, Sammon CJ, Osborn DP, Evans SJ, Cowen PJ, Freemantle N, Nazareth I. Risks and benefits of psychotropic medication in pregnancy: cohort studies based on UK electronic primary care health records. Health Technol Assess. 2016;20(23):1-176. doi: 10.3310/hta20230.	**Included**
597	Rezgui R, Blumer K, Yeoh-Tan G, Trexler AJ, Magzoub M. Precise quantification of cellular uptake of cell-penetrating peptides using fluorescence-activated cell sorting and fluorescence correlation spectroscopy. Biochim Biophys Acta. 2016;1858(7 Pt A):1499-506. doi: 10.1016/j.bbamem.2016.03.023. Epub 2016 Mar 29.	Unrelated
598	Vanya M, Devosa I, Szok D, Bártfai G. Epilepsziával szövődött terhesség preconceptionalis és perinatalis kihívásai [Preconceptional and perinatal challenges of pregnancy in women with epilepsy]. Orv Hetil. 2016 Apr 10;157(15):563-8. Hungarian. doi: 10.1556/650.2016.30342.	Unfocused
599	Dolk H, Wang H, Loane M, Morris J, Garne E, Addor MC, Arriola L, Bakker M, Barisic I, Doray B, Gatt M, Kallen K, Khoshnood B, Klungsoyr K, Lahesmaa- Korpinen AM, Latos-Bielenska A, Mejnartowicz JP, Nelen V, Neville A, O'Mahony M, Pierini A, Rißmann A, Tucker D, Wellesley D, Wiesel A, de Jong-van den Berg LT. Lamotrigine use in pregnancy and risk of orofacial cleft and other congenital anomalies. Neurology. 2016;86(18):1716-25. doi: 10.1212/WNL.0000000000002540. Epub 2016 Apr 6.	No antipsychotic
600	Li C, Tang Y, Yang J, Zhang X, Liu Y, Tang A. Sub-chronic antipsychotic drug administration reverses the expression of neuregulin 1 and ErbB4 in a cultured MK801-induced mouse primary hippocampal neuron or a neurodevelopmental schizophrenia model. Neurochem Res. 2016;41(8):2049-64. doi: 10.1007/s11064-016-1917-x. Epub 2016 Apr 21.	Animal
601	Shah B, Khunt D, Misra M, Padh H. "Application of Box-Behnken design for optimization and development of quetiapine fumarate loaded chitosan nanoparticles for brain delivery via intranasal route*". Int J Biol Macromol. 2016;89:206-18. doi: 10.1016/j.ijbiomac.2016.04.076. Epub 2016 Apr 27.	Unrelated
602	Andrade C. Major malformation risk, pregnancy outcomes, and neurodevelopmental outcomes associated with metformin use during pregnancy. J Clin Psychiatry. 2016;77(4):e411-4. doi: 10.4088/JCP.16f10789.	No antipsychotic
603	Javidi S, Razavi BM, Hosseinzadeh H. A review of neuropharmacology effects of Nigella sativa and its main component, thymoquinone. Phytother Res. 2016;30(8):1219-29. doi: 10.1002/ptr.5634. Epub 2016 May 11.	Unrelated
604	Singh KP, Singh MK, Singh M. Effects of prenatal exposure to antipsychotic risperidone on developmental neurotoxicity, apoptotic neurodegeneration and neurobehavioral sequelae in rat offspring. Int J Dev Neurosci. 2016;52:13-23. doi: 10.1016/j.ijdevneu.2016.05.006. Epub 2016 May 13.	Animal
605	Jiang L, Wu X, Wang S, Chen SH, Zhou H, Wilson B, Jin CY, Lu RB, Xie K, Wang Q, Hong JS. Clozapine metabolites protect dopaminergic neurons through inhibition of microglial NADPH oxidase. J Neuroinflammation. 2016 May 16;13(1):110. doi: 10.1186/s12974-016-0573-z. PMID: 27184631; PMCID: PMC4869380.	Unrelated
606	Sawant K, Pandey A, Patel S. Aripiprazole loaded poly (caprolactone) nanoparticles: Optimization and in vivo pharmacokinetics. Mater Sci Eng C Mater Biol Appl. 2016;66:230-243. doi: 10.1016/j.msec.2016.04.089. Epub 2016 Apr 27.	Unrelated
607	Singh KP, Singh MK, Gautam S. Effect of in utero exposure to the atypical anti-psychotic risperidone on histopathological features of the rat placenta. Int J Exp Pathol. 2016;97(2):125-32. doi: 10.1111/iep.12176. Epub 2016 Jun 3.	Animal
608	Fasinu PS, Phillips S, ElSohly MA, Walker LA. Current status and prospects for cannabidiol preparations as new therapeutic agents. Pharmacotherapy. 2016;36(7):781-96. doi: 10.1002/phar.1780.	Unrelated
609	Convertino I, Sansone AC, Marino A, Galiulo MT, Mantarro S, Antonioli L, Fornai M, Blandizzi C, Tuccori M. Neonatal adaptation issues after maternal exposure to prescription drugs: Withdrawal syndromes and residual pharmacological effects. Drug Saf. 2016;39(10):903-24. doi: 10.1007/s40264-016-0435-8.	Unfocused
610	Zamora Rodríguez FJ, Benítez Vega C, Sánchez-Waisen Hernández MR, Guisado Macías JA, Vaz Leal FJ. Use of paliperidone palmitate throughout a schizoaffective disorder patient's gestation period. Pharmacopsychiatry. 2017;50(1):38-40. doi: 10.1055/s-0042-110492. Epub 2016 Jul 14.	Case
611	Moraes BS, Vieira SM, Salgueiro WG, Michels LR, Colomé LM, Avila DS, Haas SE. Clozapine-loaded polysorbate-coated polymeric nanocapsules: Physico-chemical characterization and toxicity evaluation in Caenorhabditis elegans model. J Nanosci Nanotechnol. 2016;16(2):1257-64. doi: 10.1166/jnn.2016.11668. PMID: 27433575.	Animal
612	Martinez Ferri M, Peña Mayor P, Perez López-Fraile I, Escartin Siquier A, Martin Moro M, Forcadas Berdusan M; en representación del registro EURAP España. Comparative study of antiepileptic drug use during pregnancy over a period of 12 years in Spain. Efficacy of the newer antiepileptic drugs lamotrigine, levetiracetam, and oxcarbazepine. Neurología (Engl Ed). 2018;33(2):78-84. English, Spanish. doi: 10.1016/j.nrl.2016.05.004. Epub 2016 Jul 21.	No antipsychotic
613	Petersen I, Sammon CJ, McCrea RL, Osborn DPJ, Evans SJ, Cowen PJ, Nazareth I. Risks associated with antipsychotic treatment in pregnancy: Comparative cohort studies based on electronic health records. Schizophr Res. 2016;176(2-3):349-356. doi: 10.1016/j.schres.2016.07.023. Epub 2016 Jul 30.	**Included**

614	Huybrechts KF, Hernández-Díaz S, Patorno E, Desai RJ, Mogun H, Dejene SZ, Cohen JM, Panchaud A, Cohen L, Bateman BT. Antipsychotic use in pregnancy and the risk for congenital malformations. JAMA Psychiatry. 2016;73(9):938-46. doi: 10.1001/jamapsychiatry.2016.1520.	**Included**
615	Bergemann N, Paulus WE. Psychopharmakotherapie in der Schwangerschaft : Welche Antipsychotika, Tranquilizer und Hypnotika sind geeignet? [Psychopharmacotherapy during pregnancy : Which antipsychotics, tranquilizers and hypnotics are suitable?]. Nervenarzt. 2016;87(9):943-54. German. doi: 10.1007/s00115-016-0192-z.	Review
616	Mehta TM, Van Lieshout RJ. A review of the safety of clozapine during pregnancy and lactation. Arch Womens Ment Health. 2017;20(1):1-9. doi: 10.1007/s00737-016-0670-0. Epub 2016 Oct 4.	Review
617	Prakash C, Hatters-Friedman S, Moller-Olsen C, North A. Maternal and fetal outcomes after lamotrigine use in pregnancy: A retrospective analysis from an urban maternal mental health centre in New Zealand. Psychopharmacol Bull. 2016;46(2):63-69.	No antipsychotic
618	Rosenberg K. No increase in congenital malformations with use of antipsychotics in pregnancy. Am J Nurs. 2016;116(11):61. doi: 10.1097/01.NAJ.0000505592.85826.98.	Opinion
619	Gentile S, Fusco ML. Neurodevelopmental outcomes in infants exposed in utero to antipsychotics: a systematic review of published data. CNS Spectr. 2017;22(3):273-281. doi: 10.1017/S1092852916000699. Epub 2016 Nov 21.	Review
620	Katare YK, Piazza JE, Bhandari J, Daya RP, Akilan K, Simpson MJ, Hoare T, Mishra RK. Intranasal delivery of antipsychotic drugs. Schizophr Res. 2017;184:2-13. doi: 10.1016/j.schres.2016.11.027. Epub 2016 Nov 29.	Unrelated
621	Bhattarai P, Vance D, Hatefi A, Khaw BA. An in vitro demonstration of overcoming drug resistance in SKOV3 TR and MCF7 ADR with targeted delivery of polymer pro-drug conjugates. J Drug Target. 2017;25(5):436-450. doi: 10.1080/1061186X.2016.1271421. Epub 2017 Jan 5.	Unrelated
622	Prades R, Munarriz-Cuezva E, Urigüen L, Gil-Pisa I, Gómez L, Mendieta L, Royo S, Giralt E, Tarragó T, Meana JJ. The prolyl oligopeptidase inhibitor IPR19 ameliorates cognitive deficits in mouse models of schizophrenia. Eur Neuropsychopharmacol. 2017;27(2):180-191. doi: 10.1016/j.euroneuro.2016.11.016. Epub 2016 Dec 14.	Animal
623	Vitale SG, Laganà AS, Muscatello MR, La Rosa VL, Currò V, Pandolfo G, Zoccali RA, Bruno A. Psychopharmacotherapy in pregnancy and breastfeeding. Obstet Gynecol Surv. 2016;71(12):721-733. doi: 10.1097/OGX.0000000000000369.	Review
624	Singh KP, Singh MK. In utero exposure to atypical antipsychotic drug, risperidone: Effects on fetal neurotoxicity in hippocampal region and cognitive impairment in rat offspring. Prog Neuropsychopharmacol Biol Psychiatry. 2017;75:35-44. doi: 10.1016/j.pnpbp.2016.12.006. Epub 2017 Jan 4.	Animal
625	Maharshi V, Banerjee I, Nagar P, Rehan HS. Tracheo-esophageal fistula (TEF) in a newborn following maternal antenatal exposure to olanzapine. Drug Saf Case Rep. 2017 Dec;4(1):2. doi: 10.1007/s40800-016-0044-6.	Case
626	Würtz AM, Rytter D, Vestergaard CH, Christensen J, Vestergaard M, Bech BH. Prenatal exposure to antiepileptic drugs and use of primary healthcare during childhood: a population-based cohort study in Denmark. BMJ Open. 2017;7(1):e012836. doi: 10.1136/bmjopen-2016-012836.	No antipsychotic
627	Rimessi A, Pavan C, Ioannidi E, Nigro F, Morganti C, Brugnoli A, Longo F, Gardin C, Ferroni L, Morari M, Vindigni V, Zavan B, Pinton P. Protein kinase C β: a new target therapy to prevent the long-term atypical antipsychotic-induced weight gain. Neuropsychopharmacology. 2017;42(7):1491-1501. doi: 10.1038/npp.2017.20. Epub 2017 Jan 27.	Unrelated
628	Korade Ž, Liu W, Warren EB, Armstrong K, Porter NA, Konradi C. Effect of psychotropic drug treatment on sterol metabolism. Schizophr Res. 2017;187:74-81. doi: 10.1016/j.schres.2017.02.001. Epub 2017 Feb 12.	Unrelated
629	Osborne AL, Solowij N, Babic I, Huang XF, Weston-Green K. Improved social interaction, recognition and working memory with cannabidiol treatment in a prenatal infection (poly I:C) rat model. Neuropsychopharmacology. 2017;42(7):1447-1457. doi: 10.1038/npp.2017.40. Epub 2017 Feb 23.	Unrelated
630	Morris WE, Goldstein J, Redondo LM, Cangelosi A, Geoghegan P, Brocco M, Loidl FC, Fernandez-Miyakawa ME. Clostridium perfringens epsilon toxin induces permanent neuronal degeneration and behavioral changes. Toxicon. 2017;130:19-28. doi: 10.1016/j.toxicon.2017.02.019. Epub 2017 Feb 22.	Unrelated
631	Grigoriadis S, Peer M. Largest study to date shows overall use of antipsychotics in pregnancy does not appear to significantly increase the risk of congenital malformations. Evid Based Ment Health. 2017;20(2):e7. doi: 10.1136/eb-2016-102578. Epub 2017 Mar 14.	Opinion
632	Tosato S, Albert U, Tomassi S, Iasevoli F, Carmassi C, Ferrari S, Nanni MG, Nivoli A, Volpe U, Atti AR, Fiorillo A. A systematized review of atypical antipsychotics in pregnant women: Balancing between risks of untreated illness and risks of drug-related adverse effects. J Clin Psychiatry. 2017;78(5):e477-e489. doi: 10.4088/JCP.15r10483.	Review
633	Whitworth AB. Psychopharmacological treatment of schizophrenia during pregnancy and lactation. Curr Opin	Review

	Psychiatry. 2017;30(3):184-190. doi: 10.1097/YCO.0000000000000329.	
634	Vazão H, Rosa S, Barata T, Costa R, Pitrez PR, Honório I, de Vries MR, Papatsenko D, Benedito R, Saris D, Khademhosseini A, Quax PH, Pereira CF, Mercader N, Fernandes H, Ferreira L. High-throughput identification of small molecules that affect human embryonic vascular development. Proc Natl Acad Sci U S A. 2017;114(15):E3022-E3031. doi: 10.1073/pnas.1617451114. Epub 2017 Mar 27.	Unrelated
635	Kus K, Ratajczak P, Czaja N, Zaprutko T, Nowakowska E. Effect of combined administration of aripiprazole and fluoxetine on cognitive functions in female rats exposed to ethyl alcohol. Acta Neurobiol Exp (Wars). 2017;77(1):86-93. doi: 10.21307/ane-2017-039.	Animal
636	Pariente G, Leibson T, Shulman T, Adams-Webber T, Barzilay E, Nulman I. Pregnancy outcomes following in utero exposure to lamotrigine: A systematic review and meta-analysis. CNS Drugs. 2017;31(6):439-450. doi: 10.1007/s40263-017-0433-0. Erratum in: CNS Drugs. 2017;31(6):451.	No antipsychotic
637	McAllister-Williams RH, Baldwin DS, Cantwell R, Easter A, Gilvarry E, Glover V, Green L, Gregoire A, Howard LM, Jones I, Khalifeh H, Lingford-Hughes A, McDonald E, Micali N, Pariante CM, Peters L, Roberts A, Smith NC, Taylor D, Wieck A, Yates LM, Young AH; endorsed by the British Association for Psychopharmacology. British Association for Psychopharmacology consensus guidance on the use of psychotropic medication preconception, in pregnancy and postpartum 2017. J Psychopharmacol. 2017;31(5):519-552. doi: 10.1177/0269881117699361. Epub 2017 Apr 25.	Review
638	Hedayati A, Yazdi SG, Dehghankelishadi P, Javan NB, Akbari H, Dorkoosh FA. Preparation, optimization and physicochemical characterization of aripiprazole loaded nano-porous in situ forming implant. Pharm Nanotechnol. 2017;5(2):138-147. doi: 10.2174/2211738505666170522153930.	Unrelated
639	Patorno E, Huybrechts KF, Bateman BT, Cohen JM, Desai RJ, Mogun H, Cohen LS, Hernandez-Diaz S. Lithium use in pregnancy and the risk of cardiac malformations. N Engl J Med. 2017;376(23):2245-2254. doi: 10.1056/NEJMoa1612222.	No antipsychotic
640	Weiser M, Levi L, Levine SZ, Bialer M, Shekh-Ahmad T, Matei V, Tiugan A, Cirjaliu D, Sava C, Sinita E, Zamora D, Davis JM. A randomized, double-blind, placebo- and risperidone-controlled study on valnoctamide for acute mania. Bipolar Disord. 2017;19(4):285-294. doi: 10.1111/bdi.12506. Epub 2017 Jun 12.	Unrelated
641	Hatters Friedman S, Moller-Olsen C, Prakash C, North A. Atypical antipsychotic use and outcomes in an urban maternal mental health service. Int J Psychiatry Med. 2016;51(6):521-533. doi: 10.1177/0091217417696739. Epub 2017 Mar 6.	**Included**
642	Shin YJ, Choi JS, Ahn HK, Ryu HM, Kim MY, Han JY. Pregnancy outcomes in women reporting ingestion of levosulpiride in early pregnancy. J Obstet Gynaecol. 2017;37(8):992-995. doi: 10.1080/01443615.2017.1312307. Epub 2017 Jun 20.	**Included**
643	Li J, Yao QY, Xue JS, Wang LJ, Yuan Y, Tian XY, Su H, Wang SY, Chen WJ, Lu W, Zhou TY. Dopamine D2 receptor antagonist sulpiride enhances dexamethasone responses in the treatment of drug-resistant and metastatic breast cancer. Acta Pharmacol Sin. 2017;38(9):1282-1296. doi: 10.1038/aps.2017.24. Epub 2017 Jun 26.	Unrelated
644	Diav-Citrin O, Shechtman S, Zvi N, Finkel-Pekarsky V, Ornoy A. Is it safe to use lamotrigine during pregnancy? A prospective comparative observational study. Birth Defects Res. 2017;109(15):1196-1203. doi: 10.1002/bdr2.1058. Epub 2017 Jun 28.	No antipsychotic
645	Kong L, Zhou T, Wang B, Gao Z, Wang C. The risks associated with the use of lamotrigine during pregnancy. Int J Psychiatry Clin Pract. 2018;22(1):2-5. doi: 10.1080/13651501.2017.1341986. Epub 2017 Jun 28.	No antipsychotic
646	Ornoy A, Weinstein-Fudim L, Ergaz Z. Antidepressants, antipsychotics, and mood stabilizers in pregnancy: what do we know and how should we treat pregnant women with depression. Birth Defects Res. 2017;109(12):933-956. doi: 10.1002/bdr2.1079.	Review
647	Kronenfeld N, Berlin M, Shaniv D, Berkovitch M. Use of psychotropic medications in breastfeeding women. Birth Defects Res. 2017;109(12):957-997. doi: 10.1002/bdr2.1077. PMID: 28714610.	Review
648	Sherje AP, Londhe V. Development and evaluation of pH-responsive cyclodextrin-based in situ gel of paliperidone for intranasal delivery. AAPS PharmSciTech. 2018;19(1):384-394. doi: 10.1208/s12249-017-0844-8. Epub 2017 Jul 26. PMID: 28748368.	Unrelated
649	Liu D, Xu P, Jiang K. The use of psychotropic drugs during pregnancy. Shanghai Arch Psychiatry. 2017;29(1):48-50. doi: 10.11919/j.issn.1002-0829.216115.	Review
650	Hara Y, Ago Y, Taruta A, Hasebe S, Kawase H, Tanabe W, Tsukada S, Nakazawa T, Hashimoto H, Matsuda T, Takuma K. Risperidone and aripiprazole alleviate prenatal valproic acid-induced abnormalities in behaviors and dendritic spine density in mice. Psychopharmacology (Berl). 2017;234(21):3217-3228. doi: 10.1007/s00213-017-4703-9. Epub 2017 Aug 10.	Animal
651	Smith B, Dubovsky SL. Pharmacotherapy of mood disorders and psychosis in pre- and post-natal women. Expert Opin Pharmacother. 2017 Nov;18(16):1703-1719. doi: 10.1080/14656566.2017.1391789. Epub 2017 Nov 7.	Review
652	Cohen-Israel M, Berger I, Martonovich EY, Klinger G, Stahl B, Linder N. Short- and long-term complications	No

	of in utero exposure to lamotrigine. Br J Clin Pharmacol. 2018;84(1):189-194. doi: 10.1111/bcp.13437. Epub 2017 Oct 22.	antipsychotic
653	Wiedemann K, Stüber T, Rehn M, Frieauff E. Fetal valproate syndrome – Still a problem today! Z Geburtshilfe Neonatol. 2017;221(5):243-246. English. doi: 10.1055/s-0043-107619. Epub 2017 Oct 26.	No antipsychotic
654	Tang H, Chen H, Jia Y, Liu X, Han Z, Wang A, Liu Q, Li X, Feng X. Effect of inhibitors of endocytosis and NF-kB signal pathway on folate-conjugated nanoparticle endocytosis by rat Kupffer cells. Int J Nanomedicine. 2017;12:6937-6947. doi: 10.2147/IJN.S141407.	Animal
655	Scrandis DA. Bipolar disorder in pregnancy: A review of pregnancy outcomes. J Midwifery Womens Health. 2017;62(6):673-683. doi: 10.1111/jmwh.12645. Epub 2017 Oct 30.	Review
656	Singh SK, Hidau MK, Gautam S, Gupta K, Singh KP, Singh SK, Singh S. Glycol chitosan functionalized asenapine nanostructured lipid carriers for targeted brain delivery: Pharmacokinetic and teratogenic assessment. Int J Biol Macromol. 2018;108:1092-1100. doi: 10.1016/j.ijbiomac.2017.11.031. Epub 2017 Nov 7.	Animal
657	Drazanova E, Ruda-Kucerova J, Kratka L, Horska K, Demlova R, Starcuk Z Jr, Kasparek T. Poly(I:C) model of schizophrenia in rats induces sex-dependent functional brain changes detected by MRI that are not reversed by aripiprazole treatment. Brain Res Bull. 2018;137:146-155. doi: 10.1016/j.brainresbull.2017.11.008. Epub 2017 Nov 16.	Unrelated
658	Patel N, Viguera AC, Baldessarini RJ. Mood-stabilizing anticonvulsants, spina bifida, and folate supplementation: Commentary. J Clin Psychopharmacol. 2018;38(1):7-10. doi: 10.1097/JCP.0000000000000813.	No antipsychotic
659	Drobnis EZ, Nangia AK. Psychotropics and male reproduction. Adv Exp Med Biol. 2017;1034:63-101. doi: 10.1007/978-3-319-69535-8_8.	Unrelated
660	Graham RK, Tavella G, Parker GB. Is there consensus across international evidence-based guidelines for the psychotropic drug management of bipolar disorder during the perinatal period? J Affect Disord. 2018 Mar 1;228:216-221. doi: 10.1016/j.jad.2017.12.022. Epub 2017 Dec 12. PMID: 29274567.	Review
661	Cuomo A, Goracci A, Fagiolini A. Aripiprazole use during pregnancy, peripartum and lactation. A systematic literature search and review to inform clinical practice. J Affect Disord. 2018;228:229-237. doi: 10.1016/j.jad.2017.12.021. Epub 2017 Dec 14.	Review
662	Ware MR, Feller DB, Hall KL. Neuroleptic Malignant Syndrome: Diagnosis and Management. Prim Care Companion CNS Disord. 2018;20(1):17r02185. doi: 10.4088/PCC.17r02185.	Unrelated
663	Oliveri AN, Ortiz E, Levin ED. Developmental exposure to an organophosphate flame retardant alters later behavioral responses to dopamine antagonism in zebrafish larvae. Neurotoxicol Teratol. 2018;67:25-30. doi: 10.1016/j.ntt.2018.03.002. Epub 2018 Mar 17.	Unrelated
664	Lapoint J, Meyer S, Yu CK, Koenig KL, Lev R, Thihalolipavan S, Staats K, Kahn CA. Cannabinoid hyperemesis syndrome: Public health implications and a novel model treatment guideline. West J Emerg Med. 2018;19(2):380-386. doi: 10.5811/westjem.2017.11.36368. Epub 2017 Nov 8.	Unrelated
665	Onken M, Mick I, Schaefer C. Paliperidone and pregnancy-an evaluation of the German Embryotox database. Arch Womens Ment Health. 2018;21(6):657-662. doi: 10.1007/s00737-018-0828-z. Epub 2018 Mar 22.	**Included**
666	Damkier P, Videbech P. The safety of second-generation antipsychotics during pregnancy: A clinically focused review. CNS Drugs. 2018;32(4):351-366. doi: 10.1007/s40263-018-0517-5.	Review
667	Diana MC, Peres FF, Justi V, Bressan RA, Lacerda ALT, Crippa JA, Hallak JEC, Abilio VC. Sodium nitroprusside is effective in preventing and/or reversing the development of schizophrenia-related behaviors in an animal model: The SHR strain. CNS Neurosci Ther. 2018;24(7):624-632. doi: 10.1111/cns.12852.	Animal
668	Tomson T, Battino D, Bonizzoni E, Craig J, Lindhout D, Perucca E, Sabers A, Thomas SV, Vajda F; EURAP Study Group. Comparative risk of major congenital malformations with eight different antiepileptic drugs: a prospective cohort study of the EURAP registry. Lancet Neurol. 2018;17(6):530-538. doi: 10.1016/S1474-4422(18)30107-8.	No antipsychotic
669	Genaro-Mattos TC, Tallman KA, Allen LB, Anderson A, Mirnics K, Korade Z, Porter NA. Dichlorophenyl piperazines, including a recently-approved atypical antipsychotic, are potent inhibitors of DHCR7, the last enzyme in cholesterol biosynthesis. Toxicol Appl Pharmacol. 2018;349:21-28. doi: 10.1016/j.taap.2018.04.029.	Unfocused
670	Kumar S, Dang S, Nigam K, Ali J, Baboota S. Selegiline nanoformulation in attenuation of oxidative stress and upregulation of dopamine in the brain for the treatment of Parkinson's disease. Rejuvenation Res. 2018;21(5):464-476. doi: 10.1089/rej.2017.2035.	Unrelated
671	Verdoux H. Safety of psychotropic medicines: contribution from observational evidence. Epidemiol Psychiatr Sci. 2018;27(6):531-536. doi:10.1017/S2045796018000276.	Review
672	Galappatthy P, Liyanage CK, Lucas MN, Jayasekara DTLM, Abhayaratna SA, Weeraratne C, De Abrew K, Gunaratne PS, Gamage R, Wijeyaratne CN. Obstetric outcomes and effects on babies born to women treated for epilepsy during pregnancy in a resource limited setting: a comparative cohort study. BMC Pregnancy Childbirth.	No antipsychotic

	2018;18(1):230. doi: 10.1186/s12884-018-1857-3.	
673	Pavlek L, Kraft M, Simmons C, Ryan M, Prusakov P, Campbell A, Brandehoff N, Ng PC, Russell J, Ciciora SL, Fathi O. Acetaminophen and acetylsalicylic acid exposure in a preterm infant after maternal overdose. Am J Perinatol. 2019;36(2):136-140. doi: 10.1055/s-0038-1661405.	No antipsychotic
674	Turek A, Borecka A, Janeczek H, Sobota M, Kasperczyk J. Formulation of delivery systems with risperidone based on biodegradable terpolymers. Int J Pharm. 2018;548(1):159-172. doi: 10.1016/j.ijpharm.2018.06.051.	Unfocused
675	Galbally M, Frayne J, Watson SJ, Snellen M. Aripiprazole and pregnancy: A retrospective, multicentre study. J Affect Disord. 2018;238:593-596. doi: 10.1016/j.jad.2018.06.004.	**Included**
676	Cohen LS, Góez-Mogollón L, Sosinsky AZ, Savella GM, Viguera AC, Chitayat D, Hernández-Díaz S, Freeman MP. Risk of major malformations in infants following first-trimester exposure to quetiapine. Am J Psychiatry. 2018;175(12):1225-1231. doi:10.1176/appi.ajp.2018.18010098.	**Included**
677	Peres FF, Diana MC, Levin R, Suiama MA, Almeida V, Vendramini AM, Santos CM, Zuardi AW, Hallak JEC, Crippa JA, Abílio VC. Cannabidiol administered during peri-adolescence prevents behavioral abnormalities in an animal model of schizophrenia. Front Pharmacol. 2018;9:901. doi: 10.3389/fphar.2018.00901.	No antipsychotic
678	Gopalakrishnan G, Ganiger S, White TEK, Yu C. Reproductive toxicology studies supporting the safety of molindone, a dopamine receptor antagonist. Birth Defects Res. 2018;110(16):1250-1262. doi: 10.1002/bdr2.1381.	Animal
679	Praveen A, Aqil M, Imam SS, Ahad A, Moolakkadath T, Ahmad FJ. Lamotrigine encapsulated intra-nasal nanoliposome formulation for epilepsy treatment: Formulation design, characterization and nasal toxicity study. Colloids Surf B Biointerfaces. 2019;174:553-562. doi: 10.1016/j.colsurfb.2018.11.025.	No antipsychotic
680	Spinelli MG. Accurate assessment of risk of major malformations in infants with first-trimester exposure to quetiapine. Am J Psychiatry. 2018;175(12):1161-1162. doi: 10.1176/appi.ajp.2018.18070877.	Opinion
681	Matrisciano F, Dong E, Nicoletti F, Guidotti A. Epigenetic alterations in prenatal stress mice as an endophenotype model for schizophrenia: Role of metabotropic glutamate 2/3 receptors. Front Mol Neurosci. 2018;11:423. doi: 10.3389/fnmol.2018.00423.	Unrelated
682	Gadhave DG, Tagalpallewar AA, Kokare CR. Agranulocytosis-protective olanzapine-loaded nanostructured lipid carriers engineered for CNS delivery: Optimization and hematological toxicity studies. AAPS PharmSciTech. 2019;20(1):22. doi: 10.1208/s12249-018-1213-y.	Unrelated
683	Genaro-Mattos TC, Allen LB, Anderson A, Tallman KA, Porter NA, Korade Z, Mirnics K. Maternal aripiprazole exposure interacts with 7-dehydrocholesterol reductase mutations and alters embryonic neurodevelopment. Mol Psychiatry. 2019;24(4):491-500. doi: 10.1038/s41380-019-0368-6.	Animal
684	Betcher HK, Montiel C, Clark CT. Use of antipsychotic drugs during pregnancy. Curr Treat Options Psychiatry. 2019;6(1):17-31. doi: 10.1007/s40501-019-0165-5.	Review
685	M'bitsi-Ibouily GC, Marimuthu T, Kumar P, Choonara YE, du Toit LC, Pradeep P, Modi G, Pillay V. Synthesis, characterisation and in vitro permeation, dissolution and cytotoxic evaluation of ruthenium(II)-liganded sulpiride and amino alcohol. Sci Rep. 2019;9(1):4146. doi: 10.1038/s41598-019-40538-1.	Unrelated
686	Gentile S, Fusco ML. Schizophrenia and motherhood. Psychiatry Clin Neurosci. 2019;73(7):376-385. doi: 10.1111/pcn.12856.	Review
687	Breadon C, Kulkarni J. An update on medication management of women with schizophrenia in pregnancy. Expert Opin Pharmacother. 2019;20(11):1365-1376. doi: 10.1080/14656566.2019.1612876. PMID: 31090482.	Review
688	Mittal M, Garcia P, Lee JP, Agustines D. A case of neuroleptic malignant syndrome in pregnancy. Cureus. 2019;11(3):e4211. doi: 10.7759/cureus.4211.	Case
689	Huber-Mollema Y, Oort FJ, Lindhout D, Rodenburg R. Behavioral problems in children of mothers with epilepsy prenatally exposed to valproate, carbamazepine, lamotrigine, or levetiracetam monotherapy. Epilepsia. 2019;60(6):1069-1082. doi: 10.1111/epi.15968.	No antipsychotic
690	Ritchie HE, Huss IB, Webster WS. The effect of anti-emetic drugs on rat embryonic heart activity. Reprod Toxicol. 2019;87:140-145. doi: 10.1016/j.reprotox.2019.06.002.	Animal
691	Anderson DG, Krause A, Margolis RL. Huntington Disease-Like 2. 2004[updated 2019]. In: Adam MP, Mirzaa GM, Pagon RA, Wallace SE, Bean LJH, Gripp KW, Amemiya A, editors. GeneReviews®. Seattle (WA): University of Washington, Seattle; 1993–2023.	Unrelated
692	Mobini GR, Karimi A, Akbari A, Rahmani F. Evaluation of teratogenic activity of antiepileptic drug lamotrigine in mouse fetuses. Folia Med (Plovdiv). 2019;61(1):84-89. doi: 10.2478/folmed-2018-0058.	No antipsychotic
693	Kitagawa K, Nagai T, Yamada K. Pharmacological and proteomic analyses of neonatal polyI:C-treated adult mice. Neurosci Res. 2019;147:39-47. doi: 10.1016/j.neures.2018.10.007.	Animal
694	Ballester-Gracia I, Pérez-Almarcha M, Galvez-Llompart A, Hernandez-Viadel M. Use of long acting injectable aripiprazole before and through pregnancy in bipolar disorder: a case report. BMC Pharmacol Toxicol.	Case

	2019;20(1):52. doi: 10.1186/s40360-019-0330-x.	
695	Babanejad N, Nabid MR, Farhadian A, Dorkoosh F, Zarrintaj P, Saeb MR, Mozafari M. Sustained delivery of olanzapine from sunflower oil-based polyol-urethane nanoparticles synthesised through a cyclic carbonate ring-opening reaction. IET Nanobiotechnol. 2019;13(7):703-711. doi: 10.1049/iet-nbt.2018.5440.	Unrelated
696	Kuczynska J, Karas-Ruszczyk K, Zakrzewska A, Dermanowski M, Sienkiewicz-Jarosz H, Kurkowska-Jastrzebska I, Bienkowski P, Konopko M, Dominiak M, Mierzejewski P. Comparison of plasma, saliva, and hair lamotrigine concentrations. Clin Biochem. 2019;74:24-30. doi: 10.1016/j.clinbiochem.2019.09.009.	No antipsychotic
697	Hara Y. [Chronic Activation of the Dopaminergic Neuronal Pathway Improves Behavioral Abnormalities in the Prenatal Valproic Acid Exposure Mouse Model of Autism Spectrum Disorder]. Yakugaku Zasshi. 2019;139(11):1391-1396. Japanese. doi: 10.1248/yakushi.19-00131.	No antipsychotic
698	Kardoost M, Hajizadeh-Saffar E, Ghorbanian MT, Ghezelayagh Z, Pooshang Bagheri K, Behdani M, Habibi-Anbouhi M. Genotoxicity assessment of antiepileptic drugs (AEDs) in human embryonic stem cells. Epilepsy Res. 2019;158:106232. doi: 10.1016/j.eplepsyres.2019.106232.	No antipsychotic
699	Hunter JE, Berry-Kravis E, Hipp H, Todd PK. FMR1 Disorders. 1998 Jun 16 [updated 2019 Nov 21]. In: Adam MP, Mirzaa GM, Pagon RA, Wallace SE, Bean LJH, Gripp KW, Amemiya A, editors. GeneReviews®. Seattle (WA): University of Washington, Seattle; 1993–2023.	Review
700	Anderson KN, Ailes EC, Lind JN, Broussard CS, Bitsko RH, Friedman JM, Bobo WV, Reefhuis J, Tinker SC; National Birth Defects Prevention Study. Atypical antipsychotic use during pregnancy and birth defect risk: National Birth Defects Prevention Study, 1997-2011. Schizophr Res. 2020;215:81-88. doi: 10.1016/j.schres.2019.11.019.	**Included**
701	Betcher HK, Wisner KL. Psychotropic treatment during pregnancy: research synthesis and clinical care principles. J Womens Health (Larchmt). 2020;29(3):310-318. doi: 10.1089/jwh.2019.7781.	Review
702	Sekhar GN, Fleckney AL, Boyanova ST, Rupawala H, Lo R, Wang H, Farag DB, Rahman KM, Broadstock M, Reeves S, Thomas SA. Region-specific blood-brain barrier transporter changes leads to increased sensitivity to amisulpride in Alzheimer's disease. Fluids Barriers CNS. 2019;16(1):38. doi: 10.1186/s12987-019-0158-1.	Unfocused
703	Keni RR, Jose M, A S R, Baishya J, Sankara Sarma P, Thomas SV. Anti-epileptic drug and folic acid usage during pregnancy, seizure and malformation outcomes: Changes over two decades in the Kerala Registry of Epilepsy and Pregnancy. Epilepsy Res. 2020;159:106250. doi: 10.1016/j.eplepsyres.2019.106250. PMID: 31855827.	No antipsychotic
704	Pokharkar V, Suryawanshi S, Dhapte-Pawar V. Exploring micellar-based polymeric systems for effective nose-to-brain drug delivery as potential neurotherapeutics. Drug Deliv Transl Res. 2020;10(4):1019-1031. doi: 10.1007/s13346-019-00702-6.	Unrelated
705	Li W, Sun F, Guo X, Hu Y, Ding S, Ding M, Song M, Shao M, Yang Y, Guo W, Zhang L, Zhang Y, Wang X, Su X, Lv L. Behavioral abnormalities and phosphorylation deficits of extracellular signal-regulated kinases 1 and 2 in rat offspring of the maternal immune activation model. Physiol Behav. 2020;217:112805. doi: 10.1016/j.physbeh.2020.112805. PMID: 31954148.	Unrelated
706	Albertini E, Ernst CL, Tamaroff RS. Psychopharmacological decision making in bipolar disorder during pregnancy and lactation: A case-by-case approach to using current evidence. Focus (Am Psychiatr Publ). 2019;17(3):249-258. doi: 10.1176/appi.focus.20190007.	Review
707	Angus-Leppan H, Moghim MM, Cock H, Kinton L, Synnott Wells M, Shankar R. Valproate risk form-Surveying 215 clinicians involving 4775 encounters. Acta Neurol Scand. 2020;141(6):483-490. doi: 10.1111/ane.13231.	No antipsychotic
708	Elmowafy M, Alruwaili NK, Shalaby K, Alharbi KS, Altowayan WM, Ahmad N, Zafar A, Elkomy M. Long-Acting Paliperidone parenteral formulations based on polycaprolactone nanoparticles; the influence of stabilizer and chitosan on in vitro release, protein adsorption, and cytotoxicity. Pharmaceutics. 2020;12(2):160. doi: 10.3390/pharmaceutics12020160.	Not in women
709	Bhattamisra SK, Shak AT, Xi LW, Safian NH, Choudhury H, Lim WM, Shahzad N, Alhakamy NA, Anwer MK, Radhakrishnan AK, Md S. Nose to brain delivery of rotigotine loaded chitosan nanoparticles in human SH-SY5Y neuroblastoma cells and animal model of Parkinson's disease. Int J Pharm. 2020;579:119148. doi: 10.1016/j.ijpharm.2020.119148.	Unrelated
710	Chew HY, De Lima PO, Gonzalez Cruz JL, Banushi B, Echejoh G, Hu L, Joseph SR, Lum B, Rae J, O'Donnell JS, Merida de Long L, Okano S, King B, Barry R, Moi D, Mazzieri R, Thomas R, Souza-Fonseca-Guimaraes F, Foote M, McCluskey A, Robinson PJ, Frazer IH, Saunders NA, Parton RG, Dolcetti R, Cuff K, Martin JH, Panizza B, Walpole E, Wells JW, Simpson F. Endocytosis inhibition in humans to improve responses to ADCC-mediating antibodies. Cell. 2020;180(5):895-914.e27. doi: 10.1016/j.cell.2020.02.019. PMID: 32142680.	Unrelated
711	Jain D, Sodani A, Ray S, Ghosh P, Nandi G. Formulation of extended-release beads of lamotrigine based on alginate and Cassia fistula seed gum by QbD approach. Curr Drug Deliv. 2020;17(5):422-437. doi: 10.2174/1567201817666200317124022.	No antipsychotic

712	Stark T, Di Bartolomeo M, Di Marco R, Drazanova E, Platania CBM, Iannotti FA, Ruda-Kucerova J, D'Addario C, Kratka L, Pekarik V, Piscitelli F, Babinska Z, Fedotova J, Giurdanella G, Salomone S, Sulcova A, Bucolo C, Wotjak CT, Starcuk Z Jr, Drago F, Mechoulam R, Di Marzo V, Micale V. Altered dopamine D3 receptor gene expression in MAM model of schizophrenia is reversed by peripubertal cannabidiol treatment. Biochem Pharmacol. 2020;177:114004. doi: 10.1016/j.bcp.2020.114004.	No antipsychotic
713	Khan KU, Akhtar N, Minhas MU. Poloxamer-407-co-poly (2-acrylamido-2-methylpropane sulfonic acid) cross-linked nanogels for solubility enhancement of olanzapine: Synthesis, characterization, and toxicity evaluation. AAPS PharmSciTech. 2020;21(5):141. doi: 10.1208/s12249-020-01694-0.	Unrelated
714	Jeon JY, Bae JG, Kim KT, Cho YW. Pregnancy and epilepsy: a Korean tertiary epilepsy center review. J Korean Med Sci. 2020;35(19):e119. doi: 10.3346/jkms.2020.35.e119.	No antipsychotic
715	Patel MR, Patel RB, Thakore SD, Solanki AB. Brain targeted delivery of lurasidone HCl via nasal administration of mucoadhesive nanoemulsion formulation for the potential management of schizophrenia. Pharm Dev Technol. 2020;25(8):1018-1030. doi: 10.1080/10837450.2020.1772292.	Unfocused
716	Zhu Y, Bateman BT, Gray KJ, Hernandez-Diaz S, Mogun H, Straub L, Huybrechts KF. Oral fluconazole use in the first trimester and risk of congenital malformations: population based cohort study. BMJ. 2020;369:m1494. doi: 10.1136/bmj.m1494.	No antipsychotic
717	Genaro-Mattos TC, Anderson A, Allen LB, Tallman KA, Porter NA, Korade Z, Mirnics K. Maternal cariprazine exposure inhibits embryonic and postnatal brain cholesterol biosynthesis. Mol Psychiatry. 2020;25(11):2685-2694. doi: 10.1038/s41380-020-0801-x.	Animal
718	Kumbhar SA, Kokare CR, Shrivastava B, Gorain B, Choudhury H. Preparation, characterization, and optimization of asenapine maleate mucoadhesive nanoemulsion using Box-Behnken design: In vitro and in vivo studies for brain targeting. Int J Pharm. 2020;586:119499. doi: 10.1016/j.ijpharm.2020.119499.	Unfocused
	719: Kamali M, Johari H, Hami J. Protective Effect of Flax Seed on Brain Teratogenicity Induced by Lamotrigine in Rat Fetuses. Folia Med (Plovdiv). 2020;62(2):372-377. doi: 10.3897/folmed.62.e46759.	No antipsychotic
720	Khatoon N, Chu MQ, Zhou CH. Nanoclay-based drug delivery systems and their therapeutic potentials. J Mater Chem B. 2020;8(33):7335-7351. doi: 10.1039/d0tb01031f.	Unrelated
721	Trifu SC, Popescu A, Marian MA. Affective disorders: A question of continuing treatment during pregnancy (Review). Exp Ther Med. 2020;20(4):3474-3482. doi: 10.3892/etm.2020.8989.	Review
722	Manikkath J, Parekh HS, Mutalik S. Surface-engineered nanoliposomes with lipidated and non-lipidated peptide-dendrimeric scaffold for efficient transdermal delivery of a therapeutic agent: Development, characterization, toxicological and preclinical performance analyses. Eur J Pharm Biopharm. 2020;156:97-113. doi: 10.1016/j.ejpb.2020.09.001.	Unrelated
723	Messinger CJ, Lipsitch M, Bateman BT, He M, Huybrechts KF, MacDonald S, Mogun H, Mott K, Hernández-Díaz S. Association Between Congenital Cytomegalovirus and the Prevalence at Birth of Microcephaly in the United States. JAMA Pediatr. 2020;174(12):1159-1167. doi: 10.1001/jamapediatrics.2020.3009. PMID: 32926077.	No antipsychotic
724	Razavi BM, Abazari AR, Rameshrad M, Hosseinzadeh H. Carnosic acid prevented olanzapine-induced metabolic disorders through AMPK activation. Mol Biol Rep. 2020;47(10):7583-7592. doi: 10.1007/s11033-020-05825-5.	Unrelated
725	Hillemacher T, Simen S, Rehme MK, Frieling H. Antipsychotika in der Schwangerschaft: eine systematische Übersichtsarbeit [Antipsychotics during pregnancy: a systematic review]. Nervenarzt. 2021;92(5):494-500. German. doi: 10.1007/s00115-020-01006-8.	Review
726	Mignani S, Shi X, Karpus A, Majoral JP. Non-invasive intranasal administration route directly to the brain using dendrimer nanoplatforms: An opportunity to develop new CNS drugs. Eur J Med Chem. 2021;209:112905. doi: 10.1016/j.ejmech.2020.112905.	Unrelated
727	Kucera J, Horska K, Hruska P, Kuruczova D, Micale V, Ruda-Kucerova J, Bienertova-Vasku J. Interacting effects of the MAM model of schizophrenia and antipsychotic treatment: Untargeted proteomics approach in adipose tissue. Prog Neuropsychopharmacol Biol Psychiatry. 2021;108:110165. doi: 10.1016/j.pnpbp.2020.110165.	Unfocused
728	Hamieh AM, Babin D, Sablé E, Hernier AM, Castagné V. Neonatal phencyclidine and social isolation in the rat: effects of clozapine on locomotor activity, social recognition, prepulse inhibition, and executive functions deficits. Psychopharmacology (Berl). 2021;238(2):517-528. doi: 10.1007/s00213-020-05700-y.	Animal
729	Yakubu MT, Fayemo HT. Anti-hyperprolactinemic activities of aqueous extract of Uvaria chamae (P. Beauv) roots and associated biochemical changes in chlorpromazine-induced hyperprolactinemic female Wistar rats. J Ethnopharmacol.2021;271:113863. doi: 10.1016/j.jep.2021.113863.	Unrelated
730	Brajcich MR, Palau MA, Messer RD, Murphy ME, Marks J. Why the maternal medication list matters: Neonatal toxicity from combined serotonergic exposures. Pediatrics. 2021;147(2):e20192250. doi: 10.1542/peds.2019-2250.	Case

731	Kumbhar SA, Kokare CR, Shrivastava B, Gorain B, Choudhury H. Antipsychotic potential and safety profile of TPGS-based mucoadhesive aripiprazole nanoemulsion: Development and optimization for nose-to-brain delivery. J Pharm Sci. 2021;110(4):1761-1778. doi: 10.1016/j.xphs.2021.01.021.	Unrelated
732	Bateman BT, Hernandez-Diaz S, Straub L, Zhu Y, Gray KJ, Desai RJ, Mogun H, Gautam N, Huybrechts KF. Association of first trimester prescription opioid use with congenital malformations in the offspring: population based cohort study. BMJ. 2021;372:n102. doi: 10.1136/bmj.n102.	No antipsychotic
733	Kaplan YC, Demir O. Use of phenytoin, phenobarbital carbamazepine, levetiracetam lamotrigine and valproate in pregnancy and breastfeeding: Risk of major malformations, dose-dependency, monotherapy vs polytherapy, pharmacokinetics and clinical implications. Curr Neuropharmacol. 2021;19(11):1805-1824. doi: 10.2174/1570159X19666210211150856. PMID: 33573557.	No antipsychotic
734	Ballout RA, El-Hattab AW, Schaaf CP, Cheung SW. Xq28 Duplication Syndrome, Int22h1/Int22h2 Mediated. 2016 [updated 2021 Feb 25]. In: Adam MP, Mirzaa GM, Pagon RA, Wallace SE, Bean LJH, Gripp KW, Amemiya A, editors. GeneReviews® [Internet]. Seattle (WA): University of Washington, Seattle; 1993–2023.	Unrelated
735	Freeman MP, Viguera AC, Góez-Mogollón L, Young AV, Caplin PS, McElheny SA, Church TR, Chitayat D, Hernández-Díaz S, Cohen LS. Reproductive safety of aripiprazole: data from the Massachusetts General Hospital National Pregnancy Registry for Atypical Antipsychotics. Arch Womens Ment Health. 2021;24(4):659-667. doi: 10.1007/s00737-021-01115-6.	**Included**
736	Mohyeldin SM, Samy WM, Ragab D, Abdelmonsif DA, Aly RG, Elgindy NA. Precisely fabricated sulpiride-loaded nanolipospheres with ameliorated oral bioavailability and antidepressant activity. Int J Nanomedicine. 2021;16:2013-2044. doi: 10.2147/IJN.S296726. Erratum in: Int J Nanomedicine. 2023;18:2069-2070.	No antipsychotic
737	Patel HP, Chaudhari PS, Gandhi PA, Desai BV, Desai DT, Dedhiya PP, Vyas BA, Maulvi FA. Nose to brain delivery of tailored clozapine nanosuspension stabilized using (+)-alpha-tocopherol polyethylene glycol 1000 succinate: Optimization and in vivo pharmacokinetic studies. Int J Pharm. 2021;600:120474. doi: 10.1016/j.ijpharm.2021.120474.	Unfocused
738	Ince S, Ozer M, Kadioglu BG, Kuzucu M, Ozkaraca M, Gezer A, Suleyman H, Cetin N. The effect of taxifolin on oxidative ovarian damage and reproductive dysfunctions induced by antipsychotic drugs in female rats. J Obstet Gynaecol Res. 2021;47(6):2140-2148. doi: 10.1111/jog.14769.	Animal
739	Wang Z, Brauer R, Man KKC, Alfageh B, Mongkhon P, Wong ICK. Prenatal exposure to antipsychotic agents and the risk of congenital malformations in children: A systematic review and meta-analysis. Br J Clin Pharmacol. 2021;87(11):4101-4123. doi: 10.1111/bcp.14839.	Review
740	Kakumoto M, Shimokawa K, Ueshima S, Hira D, Okano T. Effects of antiepileptic drugs' administration during pregnancy on the nerve cell proliferation and axonal outgrowth of human neuroblastoma SH-SY5Y nerve cells. Biochem Biophys Res Commun. 2021;554:151-157. doi: 10.1016/j.bbrc.2021.03.107.	No antipsychotic
741	Scuteri D, Cassano R, Trombino S, Russo R, Mizoguchi H, Watanabe C, Hamamura K, Katsuyama S, Komatsu T, Morrone LA, Rombolà L, Adornetto A, Laganà AS, Corasaniti MT, Tonin P, Sakurada S, Sakurada T, Nicotera P, Bagetta G. Development and Translation of NanoBEO, a Nanotechnology-Based Delivery System of Bergamot Essential Oil Deprived of Furocumarins, in the Control of Agitation in Severe Dementia. Pharmaceutics. 2021;13(3):379. doi: 10.3390/pharmaceutics13030379.	Unrelated
742	Korade Z, Heffer M, Mirnics K. Medication effects on developmental sterol biosynthesis. Mol Psychiatry. 2022;27(1):490-501. doi: 10.1038/s41380-021-01074-5. Epub 2021 Apr 5.	Unrelated
743	Liu X, Sun H, Zhang Y, Sun Y, Wang W, Xu L, Liu W. Clozapine affects the pharmacokinetics of risperidone and inhibits its metabolism and P-glycoprotein-mediated transport in vivo and in vitro: A safety attention to antipsychotic polypharmacy with clozapine and risperidone. Toxicol Appl Pharmacol. 2021;422:115560. doi: 10.1016/j.taap.2021.115560.	Unfocused
744	Orsolini L, Sceusa F, Pompili S, Mauro A, Salvi V, Volpe U. Severe and persistent mental illness (SPMI) in pregnancy and breastfeeding: focus on second-generation long acting injectable antipsychotics. Expert Opin Drug Saf. 2021;20(10):1207-1224. doi: 10.1080/14740338.2021.1928634.	Review
745	Gautam D, Singh S, Maurya P, Singh M, Kushwaha S, Saraf SA. Appraisal of nano-lipidic astaxanthin cum thermoreversible gel and its efficacy in haloperidol induced Parkinsonism. Curr Drug Deliv. 2021;18(10):1550-1562. doi: 10.2174/1567201818666210510173524.	Unrelated
746	Tetro N, Hamed R, Berman E, Eyal S. Effects of antiseizure medications on placental cells: Focus on heterodimeric placental carriers. Epilepsy Res. 2021;174:106664. doi: 10.1016/j.eplepsyres.2021.106664.	No antipsychotic
747	Ellfolk M, Leinonen MK, Gissler M, Kiuru-Kuhlefelt S, Saastamoinen L, Malm H. Second-generation antipsychotic use during pregnancy and risk of congenital malformations. Eur J Clin Pharmacol. 2021;77(11):1737-1745. doi: 10.1007/s00228-021-03169-y.	**Included**
748	Zapata RC, Chaudry BS, Valencia ML, Zhang D, Ochsner SA, McKenna NJ, Osborn O. Conserved immunomodulatory transcriptional networks underlie antipsychotic-induced weight gain. Transl Psychiatry. 2021;11(1):405. doi: 10.1038/s41398-021-01528-y.	Unfocused

749	Lefner MJ, Magnon AP, Gutierrez JM, Lopez MR, Wanat MJ. Delays to Reward Delivery Enhance the Preference for an Initially Less Desirable Option: Role for the Basolateral Amygdala and Retrosplenial Cortex. J Neurosci. 2021;41(35):7461-7478. doi: 10.1523/JNEUROSCI.0438-21.2021.	Unrelated
750	Viguera AC, Freeman MP, Góez-Mogollón L, Sosinsky AZ, McElheny SA, Church TR, Young AV, Caplin PS, Chitayat D, Hernández-Díaz S, Cohen LS. Reproductive safety of second-generation antipsychotics: Updated data from the Massachusetts General Hospital National Pregnancy Registry for Atypical Antipsychotics. J Clin Psychiatry. 2021;82(4):20m13745. doi: 10.4088/JCP.20m13745. Erratum in: J Clin Psychiatry. 2021;5(82).	**Included**
751	Li J, Toffa DH, Nguyen DK. Epilepsy and Pregnancy: An Audit of Specialized Care. Can J Neurol Sci. 2022;49(5):678-687. doi: 10.1017/cjn.2021.190. PMID: 34353406.	Unrelated
752	Mohyeldin SM, Samy WM, Ragab D, Abdelmonsif DA, Aly RG, Elgindy NA. Hybrid lipid core chitosan-TPGS shell nanocomposites as a promising integrated nanoplatform for enhanced oral delivery of sulpiride in depressive disorder therapy. Int J Biol Macromol. 2021;188:432-449. doi: 10.1016/j.ijbiomac.2021.08.035.	Unrelated
753	Mari L, Placidi F, Romigi A, Tombini M, Del Bianco C, Ulivi M, Liguori C, Manfredi N, Castelli A, Mercuri NB, Izzi F. Levetiracetam, lamotrigine and carbamazepine: which monotherapy during pregnancy? Neurol Sci. 2022;43(3):1993-2001. doi: 10.1007/s10072-021-05542-2. Epub 2021 Sep 1.	No antipsychotic
754	Müffelmann B, Hagemann A, Knaak N, Bien CG. Frauen mit Epilepsie: Wie erfolgt die Beratung bei Kinderwunsch und in der Schwangerschaft? – Eine Fallserie aus einer spezialisierten Epilepsieambulanz [Women with epilepsy before and during pregnancy: a case series of outpatient counseling in a tertiary epilepsy center]. Nervenarzt. 2022;93(6):566-574. German. doi: 10.1007/s00115-021-01198-7.	No antipsychotic
755	Andrade C. Major Congenital malformations associated with exposure to second-generation antipsychotic drugs during pregnancy. J Clin Psychiatry. 2021;82(5):21f14252. doi: 10.4088/JCP.21f14252.	Opinion
756	Alavi S, Mahjoob MA, Haeri A, Shirazi FH, Abbasian Z, Dadashzadeh S. Multivesicular liposomal depot system for sustained delivery of risperidone: development, characterization, and toxicity assessment. Drug Dev Ind Pharm. 2021;47(8):1290-1301. doi: 10.1080/03639045.2021.1989454.	Unfocused
757	Boskabadi J, Kargar-Soleiman Abad S, Mehrpisheh S, Pishavar E, Farhadi R. Suicide due to fear of COVID-19, in the last month of pregnancy, leads to neonatal seizure: A case report. Ann Med Surg (Lond). 2021;72:103119. doi: 10.1016/j.amsu.2021.103119.	Case
758	Fond G, Etchecopar-Etchart D, Blanc J, Boyer L. Antipsychotics during pregnancy and increased risk of congenital malformation in offspring: toward a systematic use of real-world data. Lancet Reg Health Eur. 2021;11:100257. doi: 10.1016/j.lanepe.2021.100257.	Opinion
759	Beex-Oosterhuis MM, Van Gool AR, Heerdink ER, van Kesteren C, van Marum RJ. Clozapine treatment during pregnancy and the postpartum period: A systematic literature review. J Clin Psychiatry. 2021;83(1):21r13952. doi: 10.4088/JCP.21r13952.	Review
760	Marson AG, Burnside G, Appleton R, Smith D, Leach JP, Sills G, Tudur-Smith C, Plumpton CO, Hughes DA, Williamson PR, Baker G, Balabanova S, Taylor C, Brown R, Hindley D, Howell S, Maguire M, Mohanraj R, Smith PE. Lamotrigine versus levetiracetam or zonisamide for focal epilepsy and valproate versus levetiracetam for generalised and unclassified epilepsy: two SANAD II non- inferiority RCTs. Health Technol Assess. 2021;25(75):1-134. doi: 10.3310/hta25750.	Unrelated
761	Zosen D, Austdal LPE, Bjørnstad S, Lumor JS, Paulsen RE. Antiepileptic drugs lamotrigine and valproate differentially affect neuronal maturation in the developing chick embryo, yet with PAX6 as a potential common mediator. Neurotoxicol Teratol. 2022;90:107057. doi: 10.1016/j.ntt.2021.107057.	No antipsychotic
762	Yland JJ, Chiu YH, Rinaudo P, Hsu J, Hernán MA, Hernández-Díaz S. Emulating a target trial of the comparative effectiveness of clomiphene citrate and letrozole for ovulation induction. Hum Reprod. 2022;37(4):793-805. doi: 0.1093/humrep/deac005.	Unrelated
763	Edinoff AN, Sathivadivel N, McNeil SE, Ly AI, Kweon J, Kelkar N, Cornett EM, Kaye AM, Kaye AD. Antipsychotic Use in Pregnancy: Patient Mental Health Challenges, Teratogenicity, Pregnancy Complications, and Postnatal Risks. Neurol Int. 2022;14(1):62-74. doi: 10.3390/neurolint14010005.	Review
764	Nigam K, Kaur A, Tyagi A, Manda K, Goswami N, Nematullah M, Khan F, Gabrani R, Gauba P, Dang S. In vitro and in vivo evaluations of PLGA nanoparticle based combinatorial drug therapy for baclofen and lamotrigine for neuropathic pain management. J Microencapsul. 2022;39(2):95-109. doi: 10.1080/02652048.2022.204175.	Unrelated
765	Dubovsky SL, Marshall D. Calcium channel antagonists for mood disorders. J Clin Psychopharmacol. 2022;42(2):188-197. Doi: 10.1097/JCP.0000000000001534.	Unrelated
766	Fernández-Abascal B, Recio-Barbero M, Sáenz-Herrero M, Segarra R. Long-acting injectable aripiprazole in pregnant women with schizophrenia: a case-series report. Ther Adv Psychopharmacol. 2021;11:2045125321991277. doi: 10.1177/2045125321991277.	Case
767	Ershadi F, Mousavi Mirzaei SM, Tabrizi N, Roshanravan B, Sahebnasagh A, Avan R. Evaluation of family planning methods in married women with epilepsy. Epilepsy Behav. 2022;129:108618. doi:	Unrelated

	10.1016/j.yebeh.2022.108618.	
768	Ma M, Yang Y, Du G, Dai Y, Zhu X, Wang W, Xu H, Zhang J, Zheng L, Zou F, Yang H, Liu B, Liu W, Ye L, Zhang R, Tian J. Improving the treatment of Parkinson's disease: Structure-based development of novel 5-HT2A/receptor antagonists/inverse agonists. Eur J Med Chem. 2022;234:114246. doi: 10.1016/j.ejmech.2022.114246.	Unrelated
769	Gyawali R, Baral A, Upreti D, Yadav CB, Gupta AK, Chandradasa M, Shoib S. Novel report on congenital talipes equinovarus (CTEV) following olanzapine exposure during pregnancy: case report and short review. Arch Womens Ment Health. 2022;25(3):671-674. doi: 10.1007/s00737-022-01221-z.	Case
770	Chiu YH, Yland JJ, Rinaudo P, Hsu J, McGrath S, Hernández-Díaz S, Hernán MA. Effectiveness and safety of intrauterine insemination vs. assisted reproductive technology: emulating a target trial using an observational database of administrative claims. Fertil Steril. 2022;117(5):981-991. doi: 10.1016/j.fertnstert.2022.02.003.	Unrelated
771	Hochbaum M, Kienitz R, Rosenow F, Schulz J, Habermehl L, Langenbruch L, Kovac S, Knake S, von Podewils F, von Brauchitsch S, Hamacher M, Strzelczyk A, Willems LM. Trends in antiseizure medication prescription patterns among all adults, women, and older adults with epilepsy: A German longitudinal analysis from 2008 to 2020. Epilepsy Behav. 2022;130:108666. doi: 10.1016/j.yebeh.2022.108666.	Unrelated
772	Straub L, Hernández-Díaz S, Bateman BT, Wisner KL, Gray KJ, Pennell PB, Lester B, McDougle CJ, Suarez EA, Zhu Y, Zakoul H, Mogun H, Huybrechts KF. Association of Antipsychotic Drug Exposure in Pregnancy With Risk of Neurodevelopmental Disorders: A National Birth Cohort Study. JAMA Intern Med. 2022;182(5):522-533. doi: 10.1001/jamainternmed.2022.0375.	Review
773	Kikuchi D, Obara T, Miura R, Suzuki N, Josaka R, Tokunaga M, Ouchi R, Usui K, Okada K. Trends in the prescription of anti-seizure medicines for pregnant women outpatients with epilepsy during 2016-2020 in Japan. Seizure. 2022;98:101-104. doi: 10.1016/j.seizure.2022.04.007.	Unrelated
774	Editorial. Antipsychotic use in pregnancy and congenital malformations. Drug Ther Bull. 2022;60(6):85. doi: 10.1136/dtb.2022.000024. PMID: 35470153.	Opinion
775	Nguyen T, Frayne J, Watson S, Lebedevs T, Teoh S, Galbally M. Long-acting injectable antipsychotic treatment during pregnancy: Outcomes for women at a tertiary maternity hospital. Psychiatry Res. 2022;313:114614. doi: 10.1016/j.psychres.2022.114614.	**Included**
776	Athar F, Ehsan M, Farooq M, Lo KB, Cheema HA, Ahmad S, Naveed A, Umer M. Adverse fetal and neonatal outcomes following in-utero exposure to oxcarbazepine: A systematic review and meta-analysis. Br J Clin Pharmacol. 2022;88(8):3600-3609. doi: 10.1111/bcp.15413.	Review
777	Andrade C. Attention-deficit/hyperactivity disorder, autism spectrum disorder, and other neurodevelopmental outcomes associated with antipsychotic drug exposure during pregnancy. J Clin Psychiatry. 2022;83(3):22f14529. doi: 10.4088/JCP.22f14529.	Opinion
778	Yakuwa N, Takahashi K, Anzai T, Ito N, Goto M, Koinuma S, Uno C, Suzuki T, Watanabe O, Yamatani A, Murashima A. Pregnancy outcomes with exposure to second-generation antipsychotics during the first trimester. J Clin Psychiatry. 2022;83(4):21m14081. doi: 10.4088/JCP.21m14081.	**Included**
779	Pa B, G SS, Thomas G, Kp A. Dosage optimization of lamotrigine in pregnancy: A pharmacometric approach using modeling and simulation. J Clin Pharmacol. 2022;62(12):1557-1565. doi: 10.1002/jcph.2111. PMID: 35739074.	No antipsychotic
780	Kowalik A, Majerek M, Mrowiec K, Solich J, Faron-Górecka A, Woźnicka O, Dziedzicka-Wasylewska M, Łukasiewicz S. Dopamine D2 and serotonin 5-HT1A dimeric receptor-binding monomeric antibody scFv as a potential ligand for carrying drugs targeting selected areas of the brain. Biomolecules. 2022;12(6):749. doi: 10.3390/biom12060749. PMID: 35740874.	Unrelated
781	Shi X, Wang Y, Zhang Y, Song C, Jiang Y, Zhao J, Xia L, Ma L, Jiang W. Effects of antiepileptic drugs polytherapy on pregnancy outcomes in women with epilepsy: An observation study in northwest China. Epilepsy Behav. 2022;135:108904. doi: 10.1016/j.yebeh.2022.108904.	No antipsychotic
782	Jiménez M, Grau-López L, Ciurans J, García-Esperón C, Fumanal A, Barambio S, Chíes E, Codina M, Becerra JL. Epilepsy and pregnancy. Factors associated with epileptic seizures during pregnancy. Neurología (Engl Ed). 2023;38(2):106-113. doi: 10.1016/j.nrleng.2020.04.029.	Unfocused
783	Mishra A, Singla R, Kumar R, Sharma A, Joshi R, Sarma P, Kaur G, Prajapat M, Bhatia A, Medhi B. Granulocyte colony-stimulating factor improved core symptoms of autism spectrum disorder via modulating glutamatergic receptors in the prefrontal cortex and hippocampus of rat brains. ACS Chem Neurosci. 2022;13(20):2942-2961. doi: 10.1021/acschemneuro.2c00270.	Animal
784	Samalin L, Arnould A, Boudieu L, Henry C, Haffen E, Drapier D, Anmella G, Pacchiarotti I, Vieta E, Belzeaux R, Llorca PM. Avis d'experts français sur la prise en charge des femmes en âge de procréer et enceintes souffrant d'un trouble bipolaire traitées par valproate [French Expert advice on the management of valproate in childbearing and pregnant women with bipolar disorder]. Encéphale. 2022;48(6):624-631. French. doi: 10.1016/j.encep.2022.07.005.	No antipsychotic

785	Cohen JM, Alvestad S, Cesta CE, Bjørk MH, Leinonen MK, Nørgaard M, Einarsdóttir K, Engeland A, Gissler M, Karlstad Ø, Klungsøyr K, Odsbu I, Reutfors J, Selmer RM, Tomson T, Ulrichsen SP, Zoega H, Furu K. Comparative safety of antiseizure medication monotherapy for major malformations. Ann Neurol. 2023;93(3):551-562. doi: 10.1002/ana.26561.	No antipsychotic
786	Huybrechts KF, Straub L, Karlsson P, Pazzagli L, Furu K, Gissler M, Hernandez-Diaz S, Nørgaard M, Zoega H, Bateman BT, Cesta CE, Cohen JM, Leinonen MK, Reutfors J, Selmer RM, Suarez EA, Ulrichsen SP, Kieler H. Association of in utero antipsychotic medication exposure with risk of congenital malformations in Nordic countries and the US. JAMA Psychiatry. 2023;80(2):156-166. doi: 10.1001/jamapsychiatry.2022.4109.	**Included**
787	Demers CJ, Walker R, Rossi NM, Bradford HM. Management of bipolar disorder during the perinatal period. Nurs Womens Health. 2023;27(1):42-52. doi: 10.1016/j.nwh.2022.11.001.	Unrelated
788	Cohen LS, Church TR, Freeman MP, Gaccione P, Caplin PS, Kobylski LA, Arakelian M, Rossa ET, Chitayat D, Hernández-Díaz S, Viguera AC. Reproductive safety of lurasidone and quetiapine: Update from the National Pregnancy Registry for Psychiatric Medications. J Womens Health (Larchmt). 2023;32(4):452-462. doi: 10.1089/jwh.2022.0310.	**Included**
789	Singh S, Deep R. Pharmacological treatment of bipolar disorder in pregnancy: An update on safety considerations. Indian J Pharmacol. 2022;54(6):443-451. doi: 10.4103/ijp.ijp_407_21.	Review
790	Gassó P, Martínez-Pinteño A, Rodríguez N, Madero S, Gómez M, Segura AG, García-Rizo C, Morén C, Mas S, Parellada E. Neurotoxic/neuroprotective effects of clozapine and the positive allosteric modulator of mGluR2 JNJ-46356479 in human neuroblastoma cell cultures. Int J Mol Sci. 2023;24(3):2054. doi: 10.3390/ijms24032054.	Unfocused
791	Viguera AC, Freeman MP, Kobylski LA, Rossa ET, Gaccione P, Chitayat D, Hernández-Díaz S, Cohen LS. Risk of major malformations following first-trimester exposure to olanzapine: Preliminary data from the Massachusetts General Hospital National Pregnancy Registry for Psychiatric Medications. J Clin Psychopharmacol. 2023;43(2):106-112. doi: 10.1097/JCP.0000000000001665.	**Included**
792	Eleftheriou G, Butera R, Sangiovanni A, Palumbo C, Bondi E. Long-acting injectable antipsychotic treatment during pregnancy: A case series. Int J Environ Res Public Health. 2023;20(4):3080. doi: 10.3390/ijerph20043080.	Case
793	Liu X, Kolding L, Momen N, Gasse C, Pedersen LH. Maternal antipsychotic use during pregnancy and congenital malformations. Am J Obstet Gynecol MFM. 2023;5(6):100950. doi: 10.1016/j.ajogmf.2023.100950.	**Included**
794	Zhu X, Yang Y, Du G, Liu B, Yu X, Ye L, Mao Y, Wang H, Tian J. Non-clinical pharmacology and toxicology studies of LPM6690061, a novel 5-hydroxytryptamine (5-HT)2A receptor inverse agonist. Food Chem Toxicol. 2023;176:113800. doi: 10.1016/j.fct.2023.113800.	Unrelated
795	Liu X, Momen N, Pedersen LH. Maternal antipsychotic use during pregnancy and congenital malformations: a reply. Am J Obstet Gynecol MFM. 2023:101016. doi: 10.1016/j.ajogmf.2023.101016. Online ahead of print 2023 May 13.	Opinion
796	Li LS, Li YY, Li DZ. Association of antipsychotic medication exposure in pregnancy with risk of congenital malformations. Am J Obstet Gynecol MFM. 2023:101015. doi: 10.1016/j.ajogmf.2023.101015. Online ahead of print 2023 May 13.	Opinion
797	Lombardo R, Ruponen M, Rautio J, Ghelardini C, Di Cesare Mannelli L, Calosi L, Bani D, Lampinen R, Kanninen KM, Koivisto AM, Penttilä E, Löppönen H, Pignatello R. Development of lyophilised Eudragit® Retard nanoparticles for the sustained release of clozapine via intranasal administration. Pharmaceutics. 2023;15(5):1554. doi: 10.3390/pharmaceutics15051554.	Unrelated
798	Howley MM, Werler MM, Fisher SC, Tracy M, Van Zutphen AR, Papadopoulos EA, Hansen C, Ailes EC, Reefhuis J, Wood ME, Browne ML; National Birth Defects Prevention Study. Maternal exposure to zolpidem and risk of specific birth defects. J Sleep Res. 2023:e13958. doi: 10.1111/jsr.13958. Online ahead of print Jun 2 2023.	No antipsychotic
799	Wang W, Battini V, Carnovale C, Noordam R, van Dijk KW, Kragholm KH, van Heemst D, Soeorg H, Sessa M. A novel approach for pharmacological substantiation of safety signals using plasma concentrations of medication and administrative/healthcare databases: A case study using Danish registries for an FDA warning on lamotrigine. Pharmacol Res. 2023;193:106811. doi: 10.1016/j.phrs.2023.106811. Epub 2023 Jun 1.	No pregnancy
800	Hrdličková K, Němcová H, Horáková A, Šebela A. Užívání antipsychotik v těhotenství a jejich dopad na vrozené malformace a ranou adaptaci novorozence [Use of antipsychotics during pregnancy and their impact on congenital malformations and early neonatal adaptation]. Ceska Gynekol. 2023;88(3):221-230. Czech. doi: 10.48095/cccg2023221.	Review
801	Matrisciano F. Epigenetic regulation of metabotropic glutamate 2/3 receptors: Potential role for ultra-resistant schizophrenia? Pharmacol Biochem Behav. 2023:173589. doi: 10.1016/j.pbb.2023.173589. Epub ahead of print 2023 Jun 20.	Review
802	Sahoo MK, Biswas H, Grover S. Safety profile of aripiprazole during pregnancy and lactation: Report of 2 cases. Türk Psikiyatri Derg. 2023;34(2):133-135. English, Turkish. doi: 10.5080/u26681.	Case

803	Litke R, Roh KH, Yoon Y, Vangeti S, Ramos-Lopez I, Kaniskan H, Jin J, Kellner C, Mobbs C. Novel compound inhibits glycolysis, proteotoxicity, inflammation, and impairments in animal models of Alzheimer's, Huntington's, and stroke: Aging as a consequence of glycolysis. bioRxiv [Preprint]. 2023 Jun 13:2023.06.12.544352. doi: 10.1101/2023.06.12.544352.	Unrelated
804	Paulzen M, Schoretsanitis G. Psychopharmakotherapie in Schwangerschaft und Stillzeit – Teil I: Schwerpunkt Schwangerschaft : Möglichkeiten der Unterstützung durch therapeutisches Drug-Monitoring [Psychopharmacotherapy during pregnancy and breastfeeding-Part I: focus on pregnancy: Support options by using therapeutic drug monitoring]. Nervenarzt. 2023. German. doi: 10.1007/s00115-023-01528-x. Epub ahead of print 2023 Jul 17.	Unfocused
805	Maxmen, Jerrold S.; Psychotropic drugs: Fast facts. New York: W W Norton & Co; 1991. xxii, 279 pp.	Review
806	Pajer, Kathleen A.; Psychotropic drugs and teratogenicity. In: Drug-induced dysfunction in psychiatry. Keshavan, Matcheri S. (Ed); Kennedy, John S. (Ed); New York: Hemisphere Publishing Corp; 1992, pp. 49-74.	Review
807	Miller, Laura J.; Psychiatric medication during pregnancy: Understanding and minimizing risks. Psychiatric Annals, 24(2), 1994 pp. 69-75. DOI: 10.3928/0048-5713-19940201-06	Opinion
808	Sams-Dodd, Frank; Lipska, Barbara K.; Weinberger, Daniel R.; Neonatal lesions of the rat ventral hippocampus result in hyperlocomotion and deficits in social behaviour in adulthood. Psychopharmacology, 132(3), 1997 pp. 303-310. DOI: 10.1007/s002130050349	Animal
809	Walker, Audrey; Rosenberg, Maris; Balaban-Gil, Karen; Neurodevelopmental and neurobehavioral sequelae of selected substances of abuse and psychiatric medications in utero. Child and Adolescent Psychiatric Clinics of North America, Vol 8(4), 1999 pp. 845-867.	Review
810	Tényi, Tamás; Trixler, Mátyás; Keresztes, Zsuzsanna; Quetiapine and pregnancy. The American Journal of Psychiatry, 159(4), 2002 pp. 674. DOI: 10.1176/appi.ajp.159.4.674	Case
811	Gupta, Nitin; Grover, Sandeep; Safety of clozapine in 2 successive pregnancies. The Canadian Journal of Psychiatry / La Revue canadienne de psychiatrie, 49(12), 2004 pp. 865. DOI: 10.1177/070674370404901213	Opinion
812	Ketter, Terence A.; Suppes, Trisha; Morrell, Martha J.; Rasgon, Natalie; Cohen, Lee S.; Viguera, Adele C.; Reproductive health and bipolar disorder. CNS Spectrums, 11(5), 2006 pp. 1-15.	Review
813	Yeshayahu, Yonatan; The use of olanzapine in pregnancy and congenital cardiac and musculoskeletal abnormalities. The American Journal of Psychiatry, 164(11), 2007 pp. 1759-1760. DOI: 10.1176/appi.ajp.2007.07010176	Case
814	Koukopoulos, Alexia; Viguera, Adele C.; Nonacs, Ruta; Petrillo, Laura F.; Cohen, Lee S.; Perinatal psychiatry and teratogenicity. In: Psychiatric genetics: Applications in clinical practice. Smoller, Jordan W. (Ed); Sheidley, Beth Rosen (Ed); Tsuang, Ming T. (Ed); Washington, D.C.: American Psychiatric Publishing, Inc.; 2008, pp. 227-254.	Review
815	Babu GN, Desai G, Tippeswamy H, Chandra PS. Birth weight and use of olanzapine in pregnancy: a prospective comparative study. J Clin Psychopharmacol. 2010;30(3):331-2. doi: 10.1097/JCP.0b013e3181db8734.	**Included**
816	Çetin, Mesut; Gebelikte psikotrop ilaç kullanımı: Bir güncelleme. Translated Title: Psychotropic drug use in pregnancy: An update. Klinik Psikofarmakoloji Bülteni/Bulletin of Clinical Psychopharmacology, 21(2), 2011 pp. 161-173. DOI: 10.5455/bcp.20110706032759	Review
817	Nawa, Hiroyuki; Yamada, Kiyofumi; Experimental schizophrenia models in rodents established with inflammatory agents and cytokines. In: Psychiatric disorders: Methods and protocols. Kobeissy, Firas H. (Ed); Totowa, New Jersey: Humana Press/Springer Nature; 2012, pp. 445-451. DOI: 10.1007/978-1-61779-458-2_28	Review
818	Widschwendter, C. G.; Hofer, A.; Aripiprazole use in early pregnancy: A case report. Pharmacopsychiatry, 45(7), 2012 pp. 299-300. DOI: 10.1055/s-0032-1312591	Case
819	Bellantuono, Cesario; Bozzi, Francesca; Orsolini, Laura; Safety of escitalopram in pregnancy: A case series. Neuropsychiatric Disease and Treatment, 9, 2013 pp. 1333-1337.	No antipsychotic
820	Degerli, Hatice Ezgi; Altinbas, Kursat; Delice, Mehtap; Kurt, Erhan; Four bipolar cases treated with quetiapine during pregnancy. Klinik Psikofarmakoloji Bülteni/Bulletin of Clinical Psychopharmacology, 24(4), 2014 pp. 391-395. DOI: 10.5455/bcp.20140714030311	Case
821	Burt, Vivien K.; Evidence-based pregnancy registries: Good for babies and their mothers. The American Journal of Psychiatry, 173(3), 2016 pp. 208-210. DOI: 10.1176/appi.ajp.2015.15111371	Opinion
822	Orsolini, Laura; Valchera, Alessandro; De Berardis, Domenico; Bellantuono, Cesario; Pregnancy and psychotropic drugs. In: Psychotropic drugs and medical conditions. Uguz, Faruk (Ed); New York: Nova Biomedical Books; 2017, pp. 181-208.	Review
823	Halpape, Katelyn; Kary, Steven; McLeod, Melanie; Mood stabilizers. In: Clinical handbook of psychotropic drugs., 23rd ed. Procyshyn, Ric M. (Ed); Bezchlibnyk-Butler, Kalyna Z. (Ed); Jeffries, J. Joel (Ed); Göttingen, D: Hogrefe; 2019, pp. 253-288.	Review

824	Brooks, Erin; Cox, Elizabeth; Kimmel, Mary; Meltzer-Brody, Samantha; Ruminjo, Anne; Risk of medication exposures in pregnancy and lactation. In: Women's mood disorders: A clinician's guide to perinatal psychiatry. Cox, Elizabeth (Ed); Cham (CH): Springer Nature Switzerland AG; 2021, pp. 55-97. DOI: 10.1007/978-3-030-71497-0_6	Review
825	Gannon, Jessica M.; Three recommendations for addressing the ongoing lithium underutilization crisis in bipolar disorder. Bipolar Disorders, 23(1), 2021 pp. 84-85. DOI: 10.1111/bdi.12914	No antipsychotic
826	Slone D, Siskind V, Heinonen OP, Monson RR, Kaufman DW, Shapiro S. Antenatal exposure to the phenothiazines in relation to congenital malformations, perinatal mortality rate, birth weight, and intelligence quotient score. Am J Obstet Gynecol. 1977;128(5):486-8. doi: 10.1016/0002-9378(77)90029-1.	**Included**
827	Einarson A, McKenna K, Levinson A. Review: women with schizophrenia have poorer pregnancy outcomes than other women, but it is unclear whether antipsychotic medications affect their infants. Evid Based Ment Health. 2003;6(3):89. doi: 10.1136/ebmh.6.3.89. Erratum in: Evid Based Ment Health. 2004;7(2):31.	Opinion
828	Royal Australian and New Zealand College of Psychiatrists Clinical Practice Guidelines Team for Depression Australian and New Zealand clinical practice guidelines for the treatment of depression. Australian & New Zealand Journal of Psychiatry, 2004; 38(6): 389-407.	Review
829	Parellada E Clinical experience and management considerations with long-acting risperidone. Current Medical Research & Opinion, 2006; 22(2): 241-255.	Review
830	Editorial. Risk of congenital malformations linked to maternal use of antipsychotics. Brown University Psychopharmacology Update, 2008; 19(8): 1-7.	Opinion
831	Andrade C Teratogenicity and hyperprolactinemia. Indian Journal of Psychiatry, 2009; 51(1): 62-64.	Opinion
832	Lim JM; Sullivan E; Kennedy D MotherSafe: review of three years of counselling by an Australian teratology Information Service. Australian & New Zealand Journal of Obstetrics & Gynaecology, 2009; 49(2): 168-172.	Unfocused
833	Devlin JW; Mallow-Corbett S; Riker RR Adverse drug events associated with the use of analgesics, sedatives, and antipsychotics in the intensive care unit. Critical Care Medicine, 2010 Supplement; 38 S231-43.	No pregnancy
834	Editorial. New drugs, drug news. P&T: A Peer-Reviewed Journal for Managed Care & Formulary Management, 2010; 35(10): 545-559.	Opinion
835	Editorial. Minimal fetal risk supports use of SGAs as first-line treatment. Brown University Psychopharmacology Update, 2013; 24(10): 3-4.	Opinion
836	McCauley, K. M.; Cross, W.; Kulkarni, J. Mental health: outcomes of 10 babies of mothers with a history of serious mental illness. Journal of Psychiatric & Mental Health Nursing, 2014; 21(7): 580-586.	No antipsychotic
837	Khalifeh, Hind; Dolman, Clare; Howard, Louise M. Safety of psychotropic drugs in pregnancy. BMJ: British Medical Journal, 2015;350(8008):h2260.	Opinion
838	Gichuhi, Stephen; Macharia, Ephantus; Kabiru, Joy; Zindamoyen, Alain M'bongo; Rono, Hilary; Ollando, Ernest; Wanyonyi, Leonard; Wachira, Joseph; Munene, Rhoda; Onyuma, Timothy; Jaoko, Walter G.; Sagoo, Mandeep S.; Weiss, Helen A.; Burton, Matthew J. Toluidine blue 0.05% vital staining for the diagnosis of ocular surface squamous neoplasia in Kenya. JAMA Ophthalmology, 2015; 133(11): 1314-1321.	Unrelated
839	Editorial. Study challenges concerns about risk of antipsychotic use during pregnancy. Brown University Psychopharmacology Update, 2016; 27(1): 1-7.	Opinion
840	Rosenberg, Karen AJN JOURNAL Watch. American Journal of Nursing, 2016; 116(11): 61-62.	Opinion
841	Editorial. Research roundup. Brown University Psychopharmacology Update, 2016; 27(12): 7-8.	Opinion
842	Mehta, Taylor M.; Van Lieshout, Ryan J. A review of the safety of clozapine during pregnancy and lactation. MIDIRS Midwifery Digest, 2017; 27(4): 504-511.	Review
843	Álvarez, A. Illana; Garvín, J. Rodríguez; Lelva, L. F. Lara Embarazo y psicofármacos. Pregnancy and psychoactive drugs. Revista ROL de Enfermería, 2018; 41(1): 22-27.	Review
844	Miller, Brian Risk of major malformations following first-trimester quetiapine exposure. Psychiatric Times, 2018; 35(11): 3.	Opinion
845	Editorial. No increased risk of malformations in infants exposed to quetiapine in utero. Brown University Psychopharmacology Update, 2018; 29(12): 3.	Opinion
846	Weiner, Ina; Piontkewitz, Yael; Arad, Michal 40.4 Effective early intervention with risperidone or clozapine in female rats prenatally exposed to poly-I:C: Must take place before the emergence of behavioral abnormalities and requires normalization of structural deficits. Schizophrenia Bulletin, 2019 Supplement 2; 45 S154-S155.	Animal
847	Editorial. Atypical antipsychotics found safe during pregnancy. Brown University Child & Adolescent Psychopharmacology Update, 2021; 23(10): 4-5.	Opinion
848	Editorial. Antipsychotic use in pregnancy and congenital malformations. Drug & Therapeutics Bulletin, 2022; 60(6): 85.	Opinion

849	Editorial. Little evidence of malformation risk in antipsychotic□exposed children. Brown University Psychopharmacology Update, 2023; 34(4): 1-6.	Opinion
850	Anderson, Kelly, Western University, Canada (Responsible Party): Use of Psychotropic Medications Among Pregnant Women With Bipolar Disorder. Sponsor Western University, Canada; Last Update Posted 2018-05-11. ClinicalTrials.gov ID NCT02970721	Unfocused
851	National Taiwan University Hospital (Fe-Lin L Wu): Study of the Causes of the Breakdown of Muscle Fibers in Hospitalized Patients. Sponsor National Taiwan University Hospital; Last Update Posted 2009-12-01. ClinicalTrials.gov ID NCT01022450	Unrelated
852	Clark, Crystal, Northwestern University (Responsible Party): Pharmacokinetics of Quetiapine Across Pregnancy and Postpartum (Quetiapine). Sponsor Northwestern University; Last Update Posted 2022-09-27. ClinicalTrials.gov ID NCT02978534	Unfocused
853	Schrijver L, Robakis TK, Kamperman AM, Bijma H, Honig A, van Kamp IL, Hoogendijk WJG, Bergink V, Poels EMP. Neurodevelopment in school-aged children after intrauterine exposure to antipsychotics. Acta Psychiatr Scand. 2023;147(1):43-53. doi: 10.1111/acps.13517. Epub 2022 Nov 10.	**Included**
854	Hálfdánarson Ó, Cohen JM, Karlstad Ø, Cesta CE, Bjørk MH, Håberg SE, Einarsdóttir K, Furu K, Gissler M, Hjellvik V, Kieler H, Leinonen MK, Nørgaard M, Öztürk Essen B, Ulrichsen SP, Reutfors J, Zoega H. Antipsychotic use in pregnancy and risk of attention/deficit-hyperactivity disorder and autism spectrum disorder: a Nordic cohort study. Evid Based Ment Health. 2022;25(2):54-62. doi: 10.1136/ebmental-2021-300311. Epub 2021 Nov 22.	**Included**
855	Straub L, Hernández-Díaz S, Bateman BT, Wisner KL, Gray KJ, Pennell PB, Lester B, McDougle CJ, Suarez EA, Zhu Y, Zakoul H, Mogun H, Huybrechts KF. Association of antipsychotic drug exposure in pregnancy with risk of neurodevelopmental disorders: A National Birth Cohort Study. JAMA Intern Med. 2022;182(5):522-533. doi: 10.1001/jamainternmed.2022.0375.	**Included**
856	Ellfolk M, Leinonen MK, Gissler M, Lahesmaa-Korpinen AM, Saastamoinen L, Nurminen ML, Malm H. Second-generation antipsychotics and pregnancy complications. Eur J Clin Pharmacol. 2020;76(1):107-115. doi: 10.1007/s00228-019-02769-z. Epub 2019 Nov 3.	**Included**

Included 38
Excluded 818
- Animal n = 238
- Review n = 162
- No antipsychotic n = 129
- Unrelated n = 121
- Case report/series n = 59
- Unfocused n = 50
- Opinion n = 50
- Not in women n = 3
- No pregnancy n = 3
- No risk assessed n = 1
- Abstract n = 1
- *In vitro* n = 1

Table S2. The ROBINS-E Risk-of-Bias of each included study.

The Risk Of Bias In Non-randomized Studies – of Exposures (ROBINS-E) assessment tool
(for follow-up studies)

Template for completion

Version 20 June 2023

This work is licensed under a Creative Commons Attribution-NonCommercial-NoDerivatives 4.0 International License.

The ROBINS-E tool

At planning stage: list confounding factors and consider appropriateness criteria

P1. List the important confounding factors relevant to all or most studies on this topic. Specify whether these are particular to specific exposures-outcome combinations.

P2. Will the review use the ROBINS-E assessment of appropriateness (important aspects of "study sensitivity")?

Yes / No

If Yes, complete sections Addressing appropriateness, Parts I and II in Appendix 1.

For each study result: preliminary considerations

A. Specify the result being assessed for risk of bias

A1. Specify the numerical result being assessed

B. Decide whether to proceed with a risk-of-bias assessment

	Response options	Comments
B1. Did the authors make any attempt to control for confounding?	Y / PY / PN / N	
B2. **If N/PN to B1**: Is there sufficient potential for confounding that an unadjusted result should not be considered further?	Y / PY / PN / N	
B3. Was the method of measuring exposure inappropriate?	Y / PY / PN / N	
B4. Was the method of measuring the outcome inappropriate?	Y / PY / PN / N	

If the answer to any of B2, B3 or B4 is 'Yes' or 'Probably yes', the result should be considered to be at very high risk of bias and no further assessment is required. Otherwise, proceed to section C.

C. Specify the analysis in the current study for which results are being assessed for risk of bias

C1. Specify the outcome to which this result relates.

C2. Specify the participant group on which this result was based.

C3 to C8: Describe the exposure measurement(s) used to produce this result.

C3. What is the exposure being measured and how was it measured or assessed?	
C4. Was exposure analysed as a quantitative (rather than a categorical) variable?	Y / PY / PN / N
C5. Did repeated measurements of exposure over time (for each participant) contribute to the analysis that produced this result?	Y / PY / PN / N
C6. **If Y/PY to C5**, was a single estimate of each participant's exposure level derived from the repeated measurements of exposure over time?	NA / Y / PY / PN / N
C7. **If N/PN to C6**, was the analysis based on splitting participants' follow up time according to exposure status and/or magnitude?	NA / Y / PY / PN / N
C8. **If Y/PY to C7**, were changes in exposure status and/or magnitude likely to be related to factors that are predictive of the outcome?	NA / Y / PY / PN / N
C9. **If N/PN to C7**, how were repeat measurements used?	

Y = Yes; PY = Probably yes; PN = Probably no; N = No; NA = Not applicable

C10. Specify the relationship analysed to produce this result. For example, this may be a quadratic relationship of cumulative exposure with the log odds of the outcome, or a risk ratio for the outcome comparing exposed with unexposed individuals.

D: Specify the causal effect of exposure being estimated by this result

D1. Specify the population of interest	Describe eligible participants (to whom the causal effect applies). These may be different from the study participants on whom the result was based (specified in C2). Such differences may give rise to selection biases.	
Specification of the exposure metric of interest		
D2. Specify the exposure	This is the factor whose causal effect on the outcome of interest is the subject of the study result being assessed. It may be thought of as the 'true' exposure of interest. It is distinct from the method with which exposure was measured.	

D3. Specify the exposure window	The exposure window of interest is the exposure period for which the result being assessed estimates the effect of exposure on the outcome. Specification of the exposure window is judged by the ROBINS-E user, who should aim to define a window that is both meaningful in answering the review question and broadly in line with when the study measured exposure. Specification should include both the time of onset and period of exposure. For example, it may be lifetime exposure (from birth or from conception), during ages 50-55, the period from first employment in a particular occupation, time from birth to age 10, or during pregnancy.<br><br>The specified exposure window is used to determine whether exposure data adequately reflect exposure during the window. Exposure before the start of the exposure window is addressed during the assessment of risk of bias due to confounding	
D4. Specify how exposure over time should be summarized	This may, for example, be ever/never exposed, cumulative exposure, average exposure, or peak exposure during the exposure period, for each participant. Alternatively, there may be only a single exposure event, or the exposure may be time invariant (such as a genetic variant or family history).	

E. Evaluation of confounding factors

Complete a row for each important confounding factor listed in advance (subsection (i)). In addition, consider any further confounding factors that are either relevant to the setting of this particular study or which the study authors identified as potentially important (subsection (ii)).

"Important" confounding factors are those for which, in the context of this study, adjustment is expected to lead to an important change in the estimated effect of the exposure.

(i) Important confounding factors listed in advance						
Confounding factor	Measured variable(s) for this factor, if any	Was this variable (or were these variables) controlled for in the analysis? (Y / N)	If this confounding factor was controlled for, was it measured validly and reliably by this variable (or these variables)?* (NA / Y / PY / PN / N / NI)	If this confounding factor was not controlled for, is there evidence that controlling for it was unnecessary?** (NA / Y / PY / PN / N)	Is failure to adjust for this confounding factor expected to bias the effect estimate towards benefit or harm of (higher) exposure?*** (Benefit of (higher) exposure / Harm of (higher) exposure / Insufficient information available)	Comments

(ii) Additional confounding factors relevant to the setting of this particular study, or identified by study authors and considered to be important, or which were identified since the protocol was written						
Confounding factor	Measured variable(s) for this factor, if any	Was this variable (or were these variables) controlled for in the analysis? (Y / N)	If this confounding factor was controlled for, was it measured validly and reliably by this variable (or these variables)?* (NA / Y / PY / PN / N / NI)	If this confounding factor was not controlled for, is there evidence that controlling for it was unnecessary?** (NA / Y / PY / PN / N)	Is failure to adjust for this confounding factor expected to bias the effect estimate towards benefit or harm of (higher) exposure?*** (Benefit of (higher) exposure / Harm of (higher) exposure / Insufficient information available)	Comments

* "Validity" refers to whether the confounding variable or variables accurately measure the confounding factor, while "reliability" refers to the precision of the measurement (more measurement error means less reliability).

** In the context of a particular study, variables need not be included in the analysis: (a) if they are measured validly and reliably and are not associated with the outcome, conditional on exposure (noting that lack of a statistically significant association is not evidence of a lack of association); (b) if they are measured validly and reliably and are not associated with exposure; (c) if they are measured validly and reliably and adjustment makes no or minimal difference to the estimated effect of the primary parameter; (d) because the confounder was addressed in the study design, for example by restricting to individuals with the same value of the confounder; (e) because a negative control demonstrates that there was unlikely to have been confounding due to this variable or that uncontrolled confounding was likely to be minimal; or (f) because external evidence suggests that controlling for the variable is not necessary in the context of the study being assessed..

For each study: risk of bias assessment

Domain 1: Risk of bias due to confounding

Domain 1, Variant (a): ***If N/PN to C5 or Y/PY to C6 or N/PN to C7*** *(only baseline confounding needs to be addressed)*

Signalling questions	Response options	Comments
1.1 Did the authors control for all the important confounding factors for	Y / PY / WN (no, but uncontrolled confounding was probably	

Signalling questions	Response options	Comments
which this was necessary?	not substantial) / SN (no, and uncontrolled confounding was probably substantial) / NI	
1.2 **If Y/PY/WN to 1.1**: Were confounding factors that were controlled for (and for which control was necessary) measured validly and reliably by the variables available in this study?	NA / Y / PY / WN (no, but the extent of measurement error in confounding factors was probably not substantial) / SN (no, and the extent of measurement error in confounding factors was probably substantial) / NI	
1.3 **If Y/PY/WN to 1.1**: Did the authors control for any variables after the start of the exposure period being studied that could have been affected by the exposure?	NA / Y / PY / PN / N / NI	
1.4 Did the use of negative controls, or other considerations, suggest serious uncontrolled confounding?	Y / PY / PN / N	
Risk of bias (due to confounding) in the estimated effect of exposure on the outcome	Low risk / Some concerns / High risk / Very high risk	
What is the predicted direction of bias due to confounding?	(Towards benefit of (higher) exposure / Towards harm of (higher) exposure / Insufficient information available)	
Is the risk of bias (due to confounding) sufficiently high, in the context of its likely direction and the magnitude of the estimated exposure effect, to threaten conclusions about whether the exposure has an important effect on the outcome?	Yes / No / Cannot tell	

Y = Yes; PY = Probably yes; PN = Probably no; N = No; SY = Strong yes; WY = Weak yes; SN = Strong no; WN = Weak no; NA = Not applicable; NI = No information

*Domain 1, variant (b): **If Y/PY to C7 and Y/PY to C8** (the analysis was based on splitting participants' follow up time according to exposure status and/or magnitude and changes in exposure status and/or magnitude likely to be related to factors that are predictive of the outcome, so both baseline and time-varying confounding need to be addressed)*

Signalling questions	Response options	Comments
1.1 Did the authors use an analysis method that was appropriate to control for time-varying as well as baseline confounding?	Y / PY / PN / N / NI	
1.2 **If Y/PY to 1.1**: Did the authors control for all the important baseline and time-varying confounding factors for which this was necessary?	NA / Y / PY / WN (no, but uncontrolled confounding was probably not substantial) / SN (no, and uncontrolled confounding was probably substantial) / NI	
1.3 **If Y/PY/WN to 1.2**: Were confounding factors that were controlled for (and for which control was necessary) measured validly and reliably by the variables available in this study?	NA / Y / WN (no, but the extent of measurement error in confounding factors was probably not substantial) / SN (no, and the extent of measurement error in confounding factors was probably substantial) / NI	
1.4 **If N/PN/NI to 1.1**: Did the authors control for time-varying factors or other variables measured after the start of the exposure window being studied?	NA / Y / PY / PN / N / NI	
1.5 Did the use of negative controls, or other considerations, suggest uncontrolled confounding?	Y / PY / PN / N	
Risk of bias (due to confounding) in the estimated effect of exposure on the outcome	Low risk / Some concerns / High risk / Very high risk	
What is the predicted direction of bias due to confounding?	Towards benefit of (higher) exposure / Towards harm of (higher) exposure / Towards null / Away from null / Insufficient information available	
Is the risk of bias (due to confounding) sufficiently high, in the context of its likely direction and the magnitude of the estimated exposure effect, to threaten conclusions about whether the exposure has an important effect on the outcome?	Yes / No / Cannot tell	

Y = Yes; PY = Probably yes; PN = Probably no; N = No; SY = Strong yes; WY = Weak yes; SN = Strong no; WN = Weak no; NA = Not applicable; NI = No information

Domain 2: Risk of bias arising from measurement of the exposure

*Domain 2, Variant (a): **If N/PN to C5** (exposure was measured at a single point in time)*

Signalling questions	Response options	Comments
**Mismeasurement or misclassification of the exposure.**		
**2.1 Does the measured exposure well-characterize the exposure metric specified to be of interest in this study? [*This was specified in the answers to D2, D3 and D4*]**	**Y / PY / WN (no, to a small extent) / SN (no, to a large extent) / NI**	
**2.2 Was the exposure likely to be measured with error, or misclassified?**	**SY (yes, probably a substantial amount) / WY (yes, but probably not a substantial amount) / PN / N / NI**	
**Bias in the estimated effect of exposure arising from mismeasurement or misclassification of the exposure**		
**2.3 If SY/WY to 2.2: Could mismeasurement or misclassification of exposure have been differential (i.e. related to the outcome or risk of the outcome)?**	**NA / SY (yes, to a large extent) / WY (yes, to a small extent) / PN / N / NI**	
**2.4 If SY/WY to 2.2 and N/PN/WY to 2.3: Is non-differential measurement error likely to bias the estimated effect of exposure on outcome?**	**NA / SY (yes, to a large extent) / WY (yes, to a small extent) / PN / N / NI**	
**Risk of bias (arising from measurement of exposure) in the estimated effect of exposure on the outcome**	**Low risk / Some concerns / High risk / Very high risk**	
**What is the predicted direction of bias arising from**	**Towards benefit of (higher) exposure / Towards**	

Signalling questions	Response options	Comments
**measurement of exposure?**	**harm of (higher) exposure / Towards null / Away from null / Insufficient information available**	
**Is the risk of bias (arising from measurement of exposure) sufficiently high, in the context of its likely direction and the magnitude of the estimated exposure effect, to threaten conclusions about whether the exposure has an important effect on the outcome?**	**Yes / No / Cannot tell**	

Y = Yes; PY = Probably yes; PN = Probably no; N = No; SY = Strong yes; WY = Weak yes; SN = Strong no; WN = Weak no; NA = Not applicable; NI = No information

*Domain 2, Variant (b): **If Y/PY to C5 and Y/PY to C6** (each individual's exposure level was estimated from measurements made at multiple time points)*

Signalling questions	Response options	Comments
**2.1 Does the measured exposure (derived from measurements at multiple time points) well-characterize the exposure metric specified to be of interest in this study? [*This was specified in the answers to D2, D3 and D4*]**	**Y / PY / WN (no, to a small extent) / SN (no, to a large extent) / NI**	
**2.2 Was there error in measurement, or misclassification, of the exposure, at each single time point?**	**SY (yes, probably a substantial amount) / WY (yes, but probably not a substantial amount) / PN / N / NI**	
**2.3 If SY/WY to 2.2: Could mismeasurement or misclassification of exposure have been differential (i.e. related to the outcome or risk of the outcome)?**	**NA / SY (yes, to a large extent) / WY (yes, to a small extent) / PN / N / NI**	
**2.4 If SY/WY to 2.2 and N/PN/WY to 2.3: Is the nature of the (non-differential) measurement error likely to bias the estimated effect of exposure on outcome?**	**NA / SY (yes, to a large extent) / WY (yes, to a small extent) / PN / N / NI**	
**Risk of bias (arising from measurement of exposure) in the estimated effect of exposure on the outcome**	**Low risk / Some concerns / High risk / Very high risk**	
**What is the predicted direction of bias arising from measurement of exposure?**	**Towards benefit of (higher) exposure / Towards harm of (higher) exposure / Towards null / Away from null / Insufficient information available**	
**Is the risk of bias (arising from measurement of exposure) sufficiently high, in the context of its likely direction and the magnitude of the estimated exposure effect, to threaten conclusions about whether the exposure has an important effect on the outcome?**	**Yes / No / Cannot tell**	

Y = Yes; PY = Probably yes; SN = Strong no; WN = Weak no; NA = Not applicable; NI = No information

*Domain 2, Variant (c): **If Y/PY to C5, N/PN to C6 and Y/PY to C7** (the analysis was based on splitting participants' follow up time according to exposure status and/or magnitude):*

Signalling questions	Response options	Comments
**2.1 Does the measured exposure (including changes over time) well-characterize the exposure metric specified to be of interest in this study? [*This was specified in the answers to D2, D3 and D4*]**	**Y / PY / WN (no, to a small extent) / SN (no, to a large extent) / NI**	
**2.2 Was there error in measurement, or misclassification, of the exposure, at each single time point?**	**SY (yes, probably a substantial amount) / WY (yes, but probably not a substantial amount) / PN / N / NI**	
**2.3 If SY/WY to 2.2: Could mismeasurement or misclassification of exposure have been differential (i.e. related to the outcome or risk of the outcome)?**	**NA / SY (yes, to a large extent) / WY (yes, to a small extent) / PN / N / NI**	
**2.4 If SY/WY to 2.2 and N/PN/WY to 2.3: Is the nature of the (non-differential) measurement error likely to bias the estimated effect of exposure on outcome?**	**NA / SY (yes, to a large extent) / WY (yes, to a small extent) / PN / N / NI**	
**Risk of bias (arising from measurement of exposure) in the estimated effect of exposure on the outcome**	**Low risk / Some concerns / High risk / Very high risk**	
**What is the predicted direction of bias arising from measurement of exposure?**	**Towards benefit of (higher) exposure / Towards harm of (higher) exposure / Towards null / Away from null / Insufficient information available**	
**Is the risk of bias (arising from measurement of exposure) sufficiently high, in the context of its likely direction and the magnitude of the estimated exposure effect, to threaten conclusions about whether the exposure has an important effect on the outcome?**	**Yes / No / Cannot tell**	

Y = Yes; PY = Probably yes; SN = Strong no; WN = Weak no; NA = Not applicable; NI = No information

Domain 3: Risk of bias in selection of participants into the study (or into the analysis)

Signalling questions	Response options	Comments

Signalling questions	Response options	Comments
3.1 Did follow-up begin at (or close to) the start of the exposure window for most participants? [*The exposure window is specified in D3*]	Y / PY / PN / N / NI	
3.2 **If N/PN to 3.1**: Is the effect of exposure likely to be constant over the period of follow up analysed?	NA / Y / PY / PN / N / NI	
3.3 Was selection of participants into the study (or into the analysis) based on participant characteristics observed after the start of the exposure window being studied? [*The exposure window is specified in D3*]	Y / PY / PN / N / NI	
3.4 **If Y/PY to 3.3**: Were these characteristics likely to be influenced by exposure or a cause of exposure?	NA / Y / PY / PN / N / NI	
3.5 **If Y/PY to 3.4**: Were these characteristics likely to be influenced by the outcome or a cause of the outcome?	NA / Y / PY / PN / N / NI	
3.6 **If N/PN to 3.2 or Y/PY to 3.5**: Is it likely that the analysis corrected for all of the potential selection biases identified in A and B above?	NA / Y / PY / PN / N / NI	
3.7 **If N/PN to 3.2 or Y/PY to 3.5**: Did sensitivity analyses demonstrate that the likely impact of the potential selection biases identified in A or B above was minimal?	NA / Y / PY / WN (no, there were no sensitivity analyses or there is evidence of some impact) / SN (no, there is evidence of substantial impact)	
Risk of bias (due to selection of participants into the study) in the estimated effect of exposure on the outcome	Low risk / Some concerns / High risk / Very high risk	
What is the predicted direction of bias due to selection of participants into the study?	Towards benefit of (higher) exposure / Towards harm of (higher) exposure / Towards null / Away from null / Insufficient information available	
Is the risk of bias (due to selection of participants into the study) sufficiently high, in the context of its likely direction and the magnitude of the estimated exposure effect, to threaten conclusions about whether the exposure has an important effect on the outcome?	Yes / No / Cannot tell	

Y = Yes; PY = Probably yes; PN = Probably no; N = No; SN = Strong no; WN = Weak no; NA = Not applicable; NI = No information

Domain 4: Risk of bias due to post-exposure interventions

Signalling questions	Response options	Comments
4.1 Were there post-exposure interventions that were influenced by prior exposure during the follow-up period?	Y / PY / PN / N / NI	
4.2 **If Y/PY to 4.1**: Is it likely that the analysis corrected for the effect of post-exposure interventions that were influenced by prior exposure?	NA / Y / PY / PN / N / NI	
Risk of bias (due post-exposure interventions) in the estimated effect of exposure on the outcome	Low risk / Some concerns / High risk / Very high risk	
What is the predicted direction of bias due to confounding?	Towards benefit of (higher) exposure / Towards harm of (higher) exposure / Towards null / Away from null / Insufficient information available	
Is the risk of bias (due post-exposure interventions) sufficiently high, in the context of its likely direction and the magnitude of the estimated exposure effect, to threaten conclusions about whether the exposure has an important effect on the outcome?	Yes / No / Cannot tell	

Y = Yes; PY = Probably yes; PN = Probably no; N = No; NA = Not applicable; NI = No information

Domain 5: Risk of bias due to missing data

Signalling questions	Response options	Comments
5.1 Were complete data on exposure status available for all, or nearly all, participants?	Y / PY / PN / N / NI	
5.2 Were complete data on the outcome available for all, or nearly all, participants?	Y / PY / PN / N / NI	
5.3 Were complete data on confounding variables available for all, or nearly all, participants?	Y / PY / PN / N / NI	
5.4 **If N/PN/NI to 5.1, 5.2 or 5.3**: Is the result based on a complete case analysis?	NA / Y / PY / PN / N / NI	
5.5 **If Y/PY/NI**: Was exclusion from the analysis because of missing data (in exposure, confounders or the outcome) likely to be related to the true value of the outcome?	NA / SY (Yes, strongly related) / WY (Yes, but not strongly related) / PN / N / NI	
5.6 **If N/PN to 5.5**: Were all or most predictors of missingness (in exposure, confounders or the outcome) included in the analysis model?	NA / SY (Yes, for sure) / WY (Yes, mostly or probably) / PN / N / NI	
5.7 **If N/PN to 5.4**: Was the analysis based on imputing missing values?	NA / Y / PY / PN / N	
5.8 **If Y/PY to 5.7**: Was imputation performed appropriately?	NA / Y / PY / WN (no, but not leading to substantial bias) / SN (no, such that bias would not be substantially reduced) / NI	
5.9 **If N/PN to 5.7**: Was an appropriate alternative method used to correct for bias due to missing data?	NA / Y / PY / WN (no, but not leading to substantial bias) / SN (no, such that bias would not be substantially reduced) / NI	
5.10 **If PN/N/NI to 5.1, 5.2 or 5.3**: Is there evidence that the result was not biased by missing data?	NA / Y / PY / PN / N	
Risk of bias (due to missing data) in the estimated effect of exposure on the outcome	Low risk / Some concerns / High risk / Very high risk	
What is the predicted direction of bias due to missing data?	Towards benefit of (higher) exposure / Towards harm of (higher) exposure / Towards null / Away from null / Insufficient information available	
Is the risk of bias (due to missing data) sufficiently high, in the context of its likely	Yes / No / Cannot tell	

Signalling questions	Response options	Comments
direction and the magnitude of the estimated exposure effect, to threaten conclusions about whether the exposure has an important effect on the outcome?		

Y = Yes; PY = Probably yes; PN = Probably no; N = No; SY = Strong yes; WY = Weak yes; NA = Not applicable; NI = No information

Domain 6: Risk of bias arising from measurement of the outcome

Signalling questions	Response options	Comments
6.1 Could measurement or ascertainment of the outcome have differed between exposure groups or levels of exposure?	Y / PY / PN / N / NI	
6.2 Were outcome assessors aware of study participants' exposure history?	Y / PY / PN / N / NI	
6.3 **If Y/PY/NI to 6.2**: Could assessment of the outcome have been influenced by knowledge of participants' exposure history?	NA / SY (yes, to a large extent) / WY (yes, to a small extent) / PN / N / NI	
Risk of bias (arising from measurement of outcomes) in the estimated effect of exposure on the outcome	Low risk / Some concerns / High risk / Very high risk	
What is the predicted direction of bias arising from measurement of outcomes?	Towards benefit of (higher) exposure / Towards harm of (higher) exposure / Towards null / Away from null / Insufficient information available	
Is the risk of bias (arising from measurement of outcomes) sufficiently high, in the context of its likely direction and the magnitude of the estimated exposure effect, to threaten conclusions about whether the exposure has an important effect on the outcome?	Yes / No / Cannot tell	

Y = Yes; PY = Probably yes; PN = Probably no; N = No; SY = Strong yes; WY = Weak yes; NA = Not applicable; NI = No information

Domain 7: Risk of bias in selection of the reported result

Signalling questions	Response options	Comments
7.1 Was the result reported in accordance with an available, pre-determined analysis plan?	Y / PY / PN / N / NI	
7.2 **If N/PN/NI to 7.1**: Is the reported effect estimate likely to be selected, based on desirability of the magnitude (or statistical significance) of the estimated effect of exposure on outcome, from multiple *exposure measurements* within the exposure domain?	NA / Y / PY / PN / N / NI	
7.3 Is the reported effect estimate likely to be selected, based on desirability of the magnitude (or statistical significance) of the estimated effect of exposure on outcome, from multiple *outcome measurements* within the outcome domain?	Y / PY / PN / N / NI	
7.4 Is the reported effect estimate likely to be selected, based on desirability of the magnitude (or statistical significance) of the estimated effect of exposure on outcome, from multiple *analyses* of the exposure-outcome relationship?	Y / PY / PN / N / NI	
7.5 Is the reported effect estimate likely to be selected, based on the basis of desirability of the results (e.g. statistical significance), from different *subgroups*?	Y / PY / PN / N / NI	
Risk of bias (due to selection of the reported result) in the estimated effect of exposure on the outcome	Low risk / Some concerns / High risk / Very high risk	
What is the predicted direction of bias due to selection of the reported result?	Towards benefit of (higher) exposure / Towards harm of (higher) exposure / Towards null / Away from null / Insufficient information available	
Is the risk of bias (due to selection of the reported result) sufficiently high, in the context of its likely direction and the magnitude of the estimated exposure effect, to threaten conclusions about whether the exposure has an important effect on the outcome?	Yes / No / Cannot tell	

Y = Yes; PY = Probably yes; PN = Probably no; N = No; NA = Not applicable; NI = No information

Overall risk of bias

	Response options	Comments
Overall risk of bias	Low risk of bias except for concerns about uncontrolled confounding / Some concerns / High risk / Very high risk	
What is the predicted direction of bias?	Towards benefit of (higher) exposure / Towards harm of (higher) exposure / Towards null / Away from null / Insufficient information available	
Is the overall risk of bias sufficiently high, in the context of its likely direction and the magnitude of the estimated exposure effect, to threaten conclusions about whether the exposure has an important effect on the outcome?	Yes / No / Cannot tell	

This work is licensed under a Creative Commons Attribution-NonCommercial-NoDerivatives 4.0 International License.

[16] Rieder, R.O.; Rosenthal, D.; Wender, P.; Blumenthal, H. The offspring of schizophrenics. Fetal and neonatal deaths. *Arch. Gen. Psychiatry* **1975**, *32*(2), 200-211. doi: 10.1001/archpsyc.1975.01760200064006.

Domain 1: Risk of bias due to confounding *variant (b): **If Y/PY to C7 and Y/PY to C8** (the analysis was based on splitting participants' follow up time according to exposure status and/or magnitude and changes in exposure status and/or magnitude likely to be related to factors that are predictive of the outcome, so both baseline and time-varying confounding need to be addressed)*

Signalling questions	Response options	Comments
1.1 Did the authors use an analysis method that was appropriate to control for time-varying as well as baseline confounding?	Y	Awkward classification of schizophrenia; unclear description of antipsychotic intake
1.2 **If Y/PY to 1.1**: Did the authors control for all the important baseline and time-varying confounding factors for which this was necessary?	Y	
1.3 **If Y/PY/WN to 1.2**: Were confounding factors that were controlled for (and for which control was necessary) measured validly and reliably by the variables available in this study?	WN	
1.4 **If N/PN/NI to 1.1**: Did the authors control for time-varying factors or other variables measured after the start of the exposure window being studied?	NA	
1.5 Did the use of negative controls, or other considerations, suggest uncontrolled confounding?	N	
Risk of bias (due to confounding) in the estimated effect of exposure on the outcome	Some concerns	
What is the predicted direction of bias due to confounding?	Towards null	
Is the risk of bias (due to confounding) sufficiently high, in the context of its likely direction and the magnitude of the estimated exposure effect, to threaten conclusions about whether the exposure has an important effect on the outcome?	Cannot tell	

Y = Yes; PY = Probably yes; PN = Probably no; N = No; SY = Strong yes; WY = Weak yes; SN = Strong no; WN = Weak no; NA = Not applicable; NI = No information

*Domain 2, Variant (b): **If Y/PY to C5 and Y/PY to C6** (each individual's exposure level was estimated from measurements made at multiple time points)*

Signalling questions	Response options	Comments
**2.1 Does the measured exposure (derived from measurements at multiple time points) well-characterize the exposure metric specified to be of interest in this study? [*This was specified in the answers to D2, D3 and D4*]**	**PY**	
**2.2 Was there error in measurement, or misclassification, of the exposure, at each single time point?**	**PN**	
**2.3 If SY/WY to 2.2: Could mismeasurement or misclassification of exposure have been differential (i.e. related to the outcome or risk of the outcome)?**	**PN**	
**2.4 If SY/WY to 2.2 and N/PN/WY to 2.3: Is the nature of the (non-differential) measurement error likely to bias the estimated effect of exposure on outcome?**	**PN**	
**Risk of bias (arising from measurement of exposure) in the estimated effect of exposure on the outcome**	**Some concerns**	
**What is the predicted direction of bias arising from measurement of exposure?**	**Towards null**	
**Is the risk of bias (arising from measurement of exposure) sufficiently high, in the context of its likely direction and the magnitude of the estimated exposure effect, to threaten conclusions about whether the exposure has an important effect on the outcome?**	**Cannot tell**	

Y = Yes; PY = Probably yes; SN = Strong no; WN = Weak no; NA = Not applicable; NI = No information

Domain 3: Risk of bias in selection of participants into the study (or into the analysis)

Signalling questions	Response options	Comments
3.1 Did follow-up begin at (or close to) the start of the exposure window for most participants? [*The exposure window is specified in D3*]	Y	
3.2 **If N/PN to 3.1**: Is the effect of exposure likely to be constant over the period of follow up analysed?	Y	
3.3 Was selection of participants into the study (or into the analysis) based on participant characteristics observed after the start of the exposure window being studied? [*The exposure window is specified in D3*]	PN	
3.4 **If Y/PY to 3.3**: Were these characteristics likely to be influenced by exposure or a cause of exposure?	N	
3.5 **If Y/PY to 3.4**: Were these characteristics likely to be influenced by the outcome or a cause of the outcome?	N	
3.6 **If N/PN to 3.2 or Y/PY to 3.5**: Is it likely that the analysis corrected for all of the potential selection biases identified in A and B above?	PY	
3.7 **If N/PN to 3.2 or Y/PY to 3.5**: Did sensitivity analyses demonstrate that the likely impact of the potential selection biases identified in A or B above was minimal?	NA	
Risk of bias (due to selection of participants into the study) in the estimated effect of exposure on the outcome	Some concerns	
What is the predicted direction of bias due to selection of participants into the study?	Towards null	
Is the risk of bias (due to selection of participants into the study) sufficiently high, in the context of its likely direction and the magnitude of the estimated exposure effect, to threaten conclusions about whether the exposure has an important effect on the outcome?	Cannot tell	

Y = Yes; PY = Probably yes; PN = Probably no; N = No; SN = Strong no; WN = Weak no; NA = Not applicable; NI = No information

Domain 4: Risk of bias due to post-exposure interventions

Signalling questions	Response options	Comments

4.1 Were there post-exposure interventions that were influenced by prior exposure during the follow-up period?	N	
4.2 **If Y/PY to 4.1**: Is it likely that the analysis corrected for the effect of post-exposure interventions that were influenced by prior exposure?	PY	
Risk of bias (due post-exposure interventions) in the estimated effect of exposure on the outcome	Low risk	
What is the predicted direction of bias due to confounding?	Towards null	
Is the risk of bias (due post-exposure interventions) sufficiently high, in the context of its likely direction and the magnitude of the estimated exposure effect, to threaten conclusions about whether the exposure has an important effect on the outcome?	Cannot tell	

Y = Yes; PY = Probably yes; PN = Probably no; N = No; NA = Not applicable; NI = No information

Domain 5: Risk of bias due to missing data

Signalling questions	Response options	Comments
5.1 Were complete data on exposure status available for all, or nearly all, participants?	NI	
5.2 Were complete data on the outcome available for all, or nearly all, participants?	PY	
5.3 Were complete data on confounding variables available for all, or nearly all, participants?	PY	
5.4 **If N/PN/NI to 5.1, 5.2 or 5.3**: Is the result based on a complete case analysis?	PY	
5.5 **If Y/PY/NI**: Was exclusion from the analysis because of missing data (in exposure, confounders or the outcome) likely to be related to the true value of the outcome?	NI	
5.6 **If N/PN to 5.5**: Were all or most predictors of missingness (in exposure, confounders or the outcome) included in the analysis model?	NI	
5.7 **If N/PN to 5.4**: Was the analysis based on imputing missing values?	N	
5.8 **If Y/PY to 5.7**: Was imputation performed appropriately?	SN	
5.9 **If N/PN to 5.7**: Was an appropriate alternative method used to correct for bias due to missing data?	NI	
5.10 **If PN/N/NI to 5.1, 5.2 or 5.3**: Is there evidence that the result was not biased by missing data?	NA	
Risk of bias (due to missing data) in the estimated effect of exposure on the outcome	High risk	
What is the predicted direction of bias due to missing data?	Away from null	
Is the risk of bias (due to missing data) sufficiently high, in the context of its likely direction and the magnitude of the estimated exposure effect, to threaten conclusions about whether the exposure has an important effect on the outcome?	Yes	

Y = Yes; PY = Probably yes; PN = Probably no; N = No; SY = Strong yes; WY = Weak yes; NA = Not applicable; NI = No information

Domain 6: Risk of bias arising from measurement of the outcome

Signalling questions	Response options	Comments
6.1 Could measurement or ascertainment of the outcome have differed between exposure groups or levels of exposure?	N	
6.2 Were outcome assessors aware of study participants' exposure history?	PN	
6.3 **If Y/PY/NI to 6.2**: Could assessment of the outcome have been influenced by knowledge of participants' exposure history?	PN	
Risk of bias (arising from measurement of outcomes) in the estimated effect of exposure on the outcome	Low risk	
What is the predicted direction of bias arising from measurement of outcomes?	Towards null	
Is the risk of bias (arising from measurement of outcomes) sufficiently high, in the context of its likely direction and the magnitude of the estimated exposure effect, to threaten conclusions about whether the exposure has an important effect on the outcome?	No	

Y = Yes; PY = Probably yes; PN = Probably no; N = No; SY = Strong yes; WY = Weak yes; NA = Not applicable; NI = No information

Domain 7: Risk of bias in selection of the reported result

Signalling questions	Response options	Comments
7.1 Was the result reported in accordance with an available, pre-determined analysis plan?	Y	
7.2 **If N/PN/NI to 7.1**: Is the reported effect estimate likely to be selected, based on desirability of the magnitude (or statistical significance) of the estimated effect of exposure on outcome, from multiple *exposure measurements* within the exposure domain?	N	
7.3 Is the reported effect estimate likely to be selected, based on desirability of the magnitude (or statistical significance) of the estimated effect of exposure on outcome, from multiple *outcome measurements* within the outcome domain?	N	
7.4 Is the reported effect estimate likely to be selected, based on desirability of the magnitude (or statistical significance) of the estimated effect of exposure on outcome, from multiple *analyses* of the exposure-outcome relationship?	N	
7.5 Is the reported effect estimate likely to be selected, based on the basis of desirability of the results (e.g. statistical significance), from different *subgroups*?	PN	
Risk of bias (due to selection of the reported result) in the estimated effect of exposure on the outcome	Some concerns	
What is the predicted direction of bias due to selection of the reported result?	Towards null	
Is the risk of bias (due to selection of the reported result) sufficiently high, in the context of its likely direction and the magnitude of the estimated exposure effect, to threaten conclusions about whether the exposure has an important effect on the outcome?	No	

Y = Yes; PY = Probably yes; PN = Probably no; N = No; NA = Not applicable; NI = No information

Overall risk of bias

	Response options
Overall risk of bias	Some concerr
What is the predicted direction of bias?	Away from nu

Is the overall risk of bias sufficiently high, in the context of its likely direction and the magnitude of the estimated exposure effect, to threaten conclusions about whether the exposure has an important effect on the outcome?	No

[17] Milkovich, L., van den Berg, B.J. An evaluation of the teratogenicity of certain antinauseant drugs. *Am. J. Obstet. Gynecol.* **1976**, *125*(2), 244-248. doi: 10.1016/0002-9378(76)90601-3.

Domain 1: Risk of bias due to confounding *variant (b): **If Y/PY to C7 and Y/PY to C8** (the analysis was based on splitting participants' follow up time according to exposure status and/or magnitude and changes in exposure status and/or magnitude likely to be related to factors that are predictive of the outcome, so both baseline and time-varying confounding need to be addressed)*

Signalling questions	Response options	Comments
1.1 Did the authors use an analysis method that was appropriate to control for time-varying as well as baseline confounding?	NI	Incongruent data presentation (figures did not match between one table and another
1.2 **If Y/PY to 1.1**: Did the authors control for all the important baseline and time-varying confounding factors for which this was necessary?	NI	
1.3 **If Y/PY/WN to 1.2**: Were confounding factors that were controlled for (and for which control was necessary) measured validly and reliably by the variables available in this study?	NA	
1.4 **If N/PN/NI to 1.1**: Did the authors control for time-varying factors or other variables measured after the start of the exposure window being studied?	NI	
1.5 Did the use of negative controls, or other considerations, suggest uncontrolled confounding?	PY	
Risk of bias (due to confounding) in the estimated effect of exposure on the outcome	High risk	
What is the predicted direction of bias due to confounding?	Away from null	
Is the risk of bias (due to confounding) sufficiently high, in the context of its likely direction and the magnitude of the estimated exposure effect, to threaten conclusions about whether the exposure has an important effect on the outcome?	Yes	

Y = Yes; PY = Probably yes; PN = Probably no; N = No; SY = Strong yes; WY = Weak yes; SN = Strong no; WN = Weak no; NA = Not applicable; NI = No information

*Domain 2, Domain 2: Risk of bias arising from measurement of the exposure Variant (b): **If Y/PY to C5 and Y/PY to C6** (each individual's exposure level was estimated from measurements made at multiple time points)*

Signalling questions	Response options	Comments
**2.1 Does the measured exposure (derived from measurements at multiple time points) well-characterize the exposure metric specified to be of interest in this study? [*This was specified in the answers to D2, D3 and D4*]**	**WN**	
**2.2 Was there error in measurement, or misclassification, of the exposure, at each single time point?**	**WY**	
**2.3 If SY/WY to 2.2: Could mismeasurement or misclassification of exposure have been differential (i.e. related to the outcome or risk of the outcome)?**	**SY**	
**2.4 If SY/WY to 2.2 and N/PN/WY to 2.3: Is the nature of the (non-differential) measurement error likely to bias the estimated effect of exposure on outcome?**	**NA**	
**Risk of bias (arising from measurement of exposure) in the estimated effect of exposure on the outcome**	**High risk**	
**What is the predicted direction of bias arising from measurement of exposure?**	**Away from null**	
**Is the risk of bias (arising from measurement of exposure) sufficiently high, in the context of its likely direction and the magnitude of the estimated exposure effect, to threaten conclusions about whether the exposure has an important effect on the outcome?**	**Yes**	

Y = Yes; PY = Probably yes; SN = Strong no; WN = Weak no; NA = Not applicable; NI = No information

Domain 3: Risk of bias in selection of participants into the study (or into the analysis)

Signalling questions	Response options	Comments
3.1 Did follow-up begin at (or close to) the start of the exposure window for most participants? [*The exposure window is specified in D3*]	Y	
3.2 **If N/PN to 3.1**: Is the effect of exposure likely to be constant over the period of follow up analysed?	PY	
3.3 Was selection of participants into the study (or into the analysis) based on participant characteristics observed after the start of the exposure window being studied? [*The exposure window is specified in D3*]	PN	
3.4 **If Y/PY to 3.3**: Were these characteristics likely to be influenced by exposure or a cause of exposure?	PN	
3.5 **If Y/PY to 3.4**: Were these characteristics likely to be influenced by the outcome or a cause of the outcome?	NA	
3.6 **If N/PN to 3.2 or Y/PY to 3.5**: Is it likely that the analysis corrected for all of the potential selection biases identified in A and B above?	NA	
3.7 **If N/PN to 3.2 or Y/PY to 3.5**: Did sensitivity analyses demonstrate that the likely impact of the potential selection biases identified in A or B above was minimal?	NA	
Risk of bias (due to selection of participants into the study) in the estimated effect of exposure on the outcome	High risk	
What is the predicted direction of bias due to selection of participants into the study?	Away from null	
Is the risk of bias (due to selection of participants into the study) sufficiently high, in the context of its likely direction and the magnitude of the estimated exposure effect, to threaten conclusions about whether the exposure has an important effect on the outcome?	Cannot tell	

Y = Yes; PY = Probably yes; PN = Probably no; N = No; SN = Strong no; WN = Weak no; NA = Not applicable; NI = No information

Domain 4: Risk of bias due to post-exposure interventions

Signalling questions	Response options	Comments
4.1 Were there post-exposure interventions that were influenced by prior exposure during the follow-up period?	N	
4.2 **If Y/PY to 4.1**: Is it likely that the analysis corrected for the effect of post-exposure interventions that were influenced by prior exposure?	NA	
Risk of bias (due post-exposure interventions) in the estimated effect of exposure on the outcome	Some concerns	
What is the predicted direction of bias due to confounding?	Away from null	
Is the risk of bias (due post-exposure interventions) sufficiently high, in the context of its likely direction and the magnitude of the estimated exposure effect, to threaten conclusions about whether the exposure has an important effect on the outcome?	Cannot tell	

Y = Yes; PY = Probably yes; PN = Probably no; N = No; NA = Not applicable; NI = No information

Domain 5: Risk of bias due to missing data

Signalling questions	Response options	Comments
5.1 Were complete data on exposure status available for all, or nearly all, participants?	PY	
5.2 Were complete data on the outcome available for all, or nearly all, participants?	PY	
5.3 Were complete data on confounding variables available for all, or nearly all, participants?	PY	
5.4 **If N/PN/NI to 5.1, 5.2 or 5.3**: Is the result based on a complete case analysis?	NA	
5.5 **If Y/PY/NI**: Was exclusion from the analysis because of missing data (in exposure, confounders or the outcome) likely to be related to the true value of the outcome?	NA	
5.6 **If N/PN to 5.5**: Were all or most predictors of missingness (in exposure, confounders or the outcome) included in the analysis model?	NA	
5.7 **If N/PN to 5.4**: Was the analysis based on imputing missing values?	N	
5.8 **If Y/PY to 5.7**: Was imputation performed appropriately?	NI	
5.9 **If N/PN to 5.7**: Was an appropriate alternative method used to correct for bias due to missing data?	NI	
5.10 **If PN/N/NI to 5.1, 5.2 or 5.3**: Is there evidence that the result was not biased by missing data?	NA	
Risk of bias (due to missing data) in the estimated effect of exposure on the outcome	Some concerns	
What is the predicted direction of bias due to missing data?	Insufficient information available	
Is the risk of bias (due to missing data) sufficiently high, in the context of its likely direction and the magnitude of the estimated exposure effect, to threaten conclusions about whether the exposure has an important effect on the outcome?	Cannot tell	

Y = Yes; PY = Probably yes; PN = Probably no; N = No; SY = Strong yes; WY = Weak yes; NA = Not applicable; NI = No information

Domain 6: Risk of bias arising from measurement of the outcome

Signalling questions	Response options	Comments
6.1 Could measurement or ascertainment of the outcome have differed between exposure groups or levels of exposure?	N	
6.2 Were outcome assessors aware of study participants' exposure history?	N	
6.3 **If Y/PY/NI to 6.2**: Could assessment of the outcome have been influenced by knowledge of participants' exposure history?	NA	
Risk of bias (arising from measurement of outcomes) in the estimated effect of exposure on the outcome	Low risk	
What is the predicted direction of bias arising from measurement of outcomes?	Insufficient information available	
Is the risk of bias (arising from measurement of outcomes) sufficiently high, in the context of its likely direction and the magnitude of the estimated exposure effect, to threaten conclusions about whether the exposure has an important effect on the outcome?	Cannot tell	

Y = Yes; PY = Probably yes; PN = Probably no; N = No; SY = Strong yes; WY = Weak yes; NA = Not applicable; NI = No information

Domain 7: Risk of bias in selection of the reported result

Signalling questions	Response options	Comments
7.1 Was the result reported in accordance with an available, pre-determined analysis plan?	Y	
7.2 **If N/PN/NI to 7.1**: Is the reported effect estimate likely to be selected, based on desirability of the magnitude (or statistical significance) of the estimated effect of exposure on outcome, from multiple *exposure measurements* within the exposure domain?	NA	
7.3 Is the reported effect estimate likely to be selected, based on desirability of the magnitude (or statistical significance) of the estimated effect of exposure on outcome, from multiple *outcome measurements* within the outcome domain?	PN	
7.4 Is the reported effect estimate likely to be selected, based on desirability of the magnitude (or statistical significance)	PN	

Signalling questions	Response options	Comments
of the estimated effect of exposure on outcome, from multiple *analyses* of the exposure-outcome relationship?		
7.5 Is the reported effect estimate likely to be selected, based on the basis of desirability of the results (e.g. statistical significance), from different *subgroups*?	PN	
Risk of bias (due to selection of the reported result) in the estimated effect of exposure on the outcome	Some concerns	
What is the predicted direction of bias due to selection of the reported result?	Towards null	
Is the risk of bias (due to selection of the reported result) sufficiently high, in the context of its likely direction and the magnitude of the estimated exposure effect, to threaten conclusions about whether the exposure has an important effect on the outcome?	Cannot tell	

Y = Yes; PY = Probably yes; PN = Probably no; N = No; NA = Not applicable; NI = No information

Overall risk of bias

	Response options	Comments
Overall risk of bias	High risk	
What is the predicted direction of bias?	Away from null	
Is the overall risk of bias sufficiently high, in the context of its likely direction and the magnitude of the estimated exposure effect, to threaten conclusions about whether the exposure has an important effect on the outcome?	Cannot tell	

[19] Slone, D.; Siskind, V.; Heinonen, O.P.; Monson, R.R.; Kaufman, D.W.; Shapiro, S. Antenatal exposure to the phenothiazines in relation to congenital malformations, perinatal mortality rate, birth weight, and intelligence quotient score. Am. *J. Obstet. Gynecol.* **1977**, *128*(5), 486-8. doi: 10.1016/0002-9378(77)90029-1.

Domain 1: Risk of bias due to confoundin *variant (b): **If Y/PY to C7 and Y/PY to C8** (the analysis was based on splitting participants' follow up time according to exposure status and/or magnitude and changes in exposure status and/or magnitude likely to be related to factors that are predictive of the outcome, so both baseline and time-varying confounding need to be addressed)*

Signalling questions	Response options	Comments
1.1 Did the authors use an analysis method that was appropriate to control for time-varying as well as baseline confounding?	Y	
1.2 **If Y/PY to 1.1**: Did the authors control for all the important baseline and time-varying confounding factors for which this was necessary?	PY	
1.3 **If Y/PY/WN to 1.2**: Were confounding factors that were controlled for (and for which control was necessary) measured validly and reliably by the variables available in this study?	NI	
1.4 **If N/PN/NI to 1.1**: Did the authors control for time-varying factors or other variables measured after the start of the exposure window being studied?	NA	
1.5 Did the use of negative controls, or other considerations, suggest uncontrolled confounding?	PN	
Risk of bias (due to confounding) in the estimated effect of exposure on the outcome	Some concerns	
What is the predicted direction of bias due to confounding?	Towards null	
Is the risk of bias (due to confounding) sufficiently high, in the context of its likely direction and the magnitude of the estimated exposure effect, to threaten conclusions about whether the exposure has an important effect on the outcome?	Cannot tell	

Y = Yes; PY = Probably yes; PN = Probably no; N = No; SY = Strong yes; WY = Weak yes; SN = Strong no; WN = Weak no; NA = Not applicable; NI = No information

Domain 2: Risk of bias arising from measurement of the exposure *Variant (b): **If Y/PY to C5 and Y/PY to C6** (each individual's exposure level was estimated from measurements made at multiple time points)*

Signalling questions	Response options	Comments
**2.1 Does the measured exposure (derived from measurements at multiple time points) well-characterize the exposure metric specified to be of interest in this study? [*This was specified in the answers to D2, D3 and D4*]**	**PY**	
**2.2 Was there error in measurement, or misclassification, of the exposure, at each single time point?**	**PN**	
**2.3 If SY/WY to 2.2: Could mismeasurement or misclassification of exposure have been differential (i.e. related to the outcome or risk of the outcome)?**	**NA**	
**2.4 If SY/WY to 2.2 and N/PN/WY to 2.3: Is the nature of the (non-differential) measurement error likely to bias the estimated effect of exposure on outcome?**	**NA**	
**Risk of bias (arising from measurement of exposure) in the estimated effect of exposure on the outcome**	**Some concerns**	
**What is the predicted direction of bias arising from measurement of exposure?**	**Insufficient information available**	
**Is the risk of bias (arising from measurement of exposure) sufficiently high, in the context of its likely direction and the magnitude of the estimated exposure effect, to threaten conclusions about whether the exposure has an important effect on the outcome?**	**No**	

Y = Yes; PY = Probably yes; SN = Strong no; WN = Weak no; NA = Not applicable; NI = No information

Domain 3: Risk of bias in selection of participants into the study (or into the analysis)

Signalling questions	Response options	Comments
3.1 Did follow-up begin at (or close to) the start of the exposure window for most participants? [*The exposure window is specified in D3*]	Y	
3.2 **If N/PN to 3.1**: Is the effect of exposure likely to be constant over the period of follow up analysed?	NA	
3.3 Was selection of participants into the study (or into the analysis) based on participant characteristics observed after the start of the exposure window being studied? [*The exposure window is specified in D3*]	PN	
3.4 **If Y/PY to 3.3**: Were these characteristics likely to be influenced by exposure or a cause of exposure?	NA	
3.5 **If Y/PY to 3.4**: Were these characteristics likely to be influenced by the outcome or a cause of the outcome?	NA	
3.6 **If N/PN to 3.2 or Y/PY to 3.5**: Is it likely that the analysis corrected for all of the potential selection biases identified in A and B above?	NA	
3.7 **If N/PN to 3.2 or Y/PY to 3.5**: Did sensitivity analyses demonstrate that the likely impact of the potential selection biases identified in A or B above was minimal?	NA	
Risk of bias (due to selection of participants into the study) in the estimated effect of exposure on the outcome	Some concerns	
What is the predicted direction of bias due to selection of participants into the study?	Away from null	
Is the risk of bias (due to selection of participants into the study) sufficiently high, in the context of its likely direction and the magnitude of the estimated exposure effect, to threaten conclusions about whether the exposure has an important effect on the outcome?	Cannot tell	

Y = Yes; PY = Probably yes; PN = Probably no; N = No; SN = Strong no; WN = Weak no; NA = Not applicable; NI = No information

Domain 4: Risk of bias due to post-exposure interventions

Signalling questions	Response options	Comments
4.1 Were there post-exposure interventions that were influenced by prior exposure during the follow-up period?	N	
4.2 **If Y/PY to 4.1**: Is it likely that the analysis corrected for the effect of post-exposure interventions that were influenced by prior exposure?	NA	
Risk of bias (due post-exposure interventions) in the estimated effect of exposure on the outcome	Low risk	
What is the predicted direction of bias due to confounding?	Insufficient information available	
Is the risk of bias (due post-exposure interventions) sufficiently high, in the context of its likely direction and the magnitude of the estimated exposure effect, to threaten conclusions about whether the exposure has an important effect on the outcome?	Cannot tell	

Y = Yes; PY = Probably yes; PN = Probably no; N = No; NA = Not applicable; NI = No information

Domain 5: Risk of bias due to missing data

Signalling questions	Response options	Comments
5.1 Were complete data on exposure status available for all, or nearly all, participants?	Y	
5.2 Were complete data on the outcome available for all, or nearly all, participants?	Y	
5.3 Were complete data on confounding variables available for all, or nearly all, participants?	Y	
5.4 **If N/PN/NI to 5.1, 5.2 or 5.3**: Is the result based on a complete case analysis?	NA	
5.5 **If Y/PY/NI**: Was exclusion from the analysis because of missing data (in exposure, confounders or the outcome) likely to be related to the true value of the outcome?	NA	
5.6 **If N/PN to 5.5**: Were all or most predictors of missingness (in exposure, confounders or the outcome) included in the analysis model?	NA	
5.7 **If N/PN to 5.4**: Was the analysis based on imputing missing values?	N	
5.8 **If Y/PY to 5.7**: Was imputation performed appropriately?	NA	
5.9 **If N/PN to 5.7**: Was an appropriate alternative method used to correct for bias due to missing data?	NA	
5.10 **If PN/N/NI to 5.1, 5.2 or 5.3**: Is there evidence that the result was not biased by missing data?	NA	
Risk of bias (due to missing data) in the estimated effect of exposure on the outcome	Low risk	
What is the predicted direction of bias due to missing data?	Towards null	
Is the risk of bias (due to missing data) sufficiently high, in the context of its likely direction and the magnitude of the estimated exposure effect, to threaten conclusions about whether the exposure has an important effect on the outcome?	No	

Y = Yes; PY = Probably yes; PN = Probably no; N = No; SY = Strong yes; WY = Weak yes; NA = Not applicable; NI = No information

Domain 6: Risk of bias arising from measurement of the outcome

Signalling questions	Response options	Comments
6.1 Could measurement or ascertainment of the outcome have differed between exposure groups or levels of exposure?	N	
6.2 Were outcome assessors aware of study participants' exposure history?	N	
6.3 **If Y/PY/NI to 6.2**: Could assessment of the outcome have been influenced by knowledge of participants' exposure history?	N	
Risk of bias (arising from measurement of outcomes) in the estimated effect of exposure on the outcome	Low risk	
What is the predicted direction of bias arising from measurement of outcomes?	Towards null	
Is the risk of bias (arising from measurement of outcomes) sufficiently high, in the context of its likely direction and the magnitude of the estimated exposure effect, to threaten conclusions about whether the exposure has an important effect on	No	

Signalling questions	Response options	Comments
the outcome?		

Y = Yes; PY = Probably yes; PN = Probably no; N = No; SY = Strong yes; WY = Weak yes; NA = Not applicable; NI = No information

Domain 7: Risk of bias in selection of the reported result

Signalling questions	Response options	Comments
7.1 Was the result reported in accordance with an available, pre-determined analysis plan?	Y	
7.2 **If N/PN/NI to 7.1**: Is the reported effect estimate likely to be selected, based on desirability of the magnitude (or statistical significance) of the estimated effect of exposure on outcome, from multiple *exposure measurements* within the exposure domain?	N	
7.3 Is the reported effect estimate likely to be selected, based on desirability of the magnitude (or statistical significance) of the estimated effect of exposure on outcome, from multiple *outcome measurements* within the outcome domain?	PN	
7.4 Is the reported effect estimate likely to be selected, based on desirability of the magnitude (or statistical significance) of the estimated effect of exposure on outcome, from multiple *analyses* of the exposure-outcome relationship?	PN	
7.5 Is the reported effect estimate likely to be selected, based on the basis of desirability of the results (e.g. statistical significance), from different *subgroups*?	PN	
Risk of bias (due to selection of the reported result) in the estimated effect of exposure on the outcome	Low risk	
What is the predicted direction of bias due to selection of the reported result?	Towards null	
Is the risk of bias (due to selection of the reported result) sufficiently high, in the context of its likely direction and the magnitude of the estimated exposure effect, to threaten conclusions about whether the exposure has an important effect on the outcome?	No	

Y = Yes; PY = Probably yes; PN = Probably no; N = No; NA = Not applicable; NI = No information

Overall risk of bias

	Response options	Comments
Overall risk of bias	Low risk of bias	
What is the predicted direction of bias?	Towards null	
Is the overall risk of bias sufficiently high, in the context of its likely direction and the magnitude of the estimated exposure effect, to threaten conclusions about whether the exposure has an important effect on the outcome?	No	

[20] Godet, P.F.; Marie-Cardine, M. Neuroleptiques, schizophrénie et grossesse. Étude épidémiologique et tératologique [Neuroleptics, schizophrenia and pregnancy. Epidemiological and teratologic study]. *Encéphale* **1991**, *17*(6), 543-547. French.

Domain 1: Risk of bias due to confounding*, variant (b): **If Y/PY to C7 and Y/PY to C8** (the analysis was based on splitting participants' follow up time according to exposure status and/or magnitude and changes in exposure status and/or magnitude likely to be related to factors that are predictive of the outcome, so both baseline and time-varying confounding need to be addressed)*

Signalling questions	Response options	Comments
1.1 Did the authors use an analysis method that was appropriate to control for time-varying as well as baseline confounding?	Y	
1.2 **If Y/PY to 1.1**: Did the authors control for all the important baseline and time-varying confounding factors for which this was necessary?	Y	
1.3 **If Y/PY/WN to 1.2**: Were confounding factors that were controlled for (and for which control was necessary) measured validly and reliably by the variables available in this study?	Y	
1.4 **If N/PN/NI to 1.1**: Did the authors control for time-varying factors or other variables measured after the start of the exposure window being studied?	NA	
1.5 Did the use of negative controls, or other considerations, suggest uncontrolled confounding?	N	
Risk of bias (due to confounding) in the estimated effect of exposure on the outcome	Low risk	
What is the predicted direction of bias due to confounding?	Towards null	
Is the risk of bias (due to confounding) sufficiently high, in the context of its likely direction and the magnitude of the estimated exposure effect, to threaten conclusions about whether the exposure has an important effect on the outcome?	No	

Y = Yes; PY = Probably yes; PN = Probably no; N = No; SY = Strong yes; WY = Weak yes; SN = Strong no; WN = Weak no; NA = Not applicable; NI = No information.

Domain 2: Risk of bias arising from measurement of the exposure *Variant (b): **If Y/PY to C5 and Y/PY to C6** (each individual's exposure level was estimated from measurements made at multiple time points)*

Signalling questions	Response options	Comments
**2.1 Does the measured exposure (derived from measurements at multiple time points) well-characterize the exposure metric specified to be of interest in this study? [*This was specified in the answers to D2, D3 and D4*]**	**Y**	
**2.2 Was there error in measurement, or misclassification, of the exposure, at each single time point?**	**N**	
**2.3 If SY/WY to 2.2: Could mismeasurement or misclassification of exposure have been differential (i.e. related to the outcome or risk of the outcome)?**	**NA**	

Signalling questions	Response options	Comments
**2.4 If SY/WY to 2.2 and N/PN/WY to 2.3: Is the nature of the (non-differential) measurement error likely to bias the estimated effect of exposure on outcome?**	**NA**	
**Risk of bias (arising from measurement of exposure) in the estimated effect of exposure on the outcome**	**Low risk**	
**What is the predicted direction of bias arising from measurement of exposure?**	**Towards null**	
**Is the risk of bias (arising from measurement of exposure) sufficiently high, in the context of its likely direction and the magnitude of the estimated exposure effect, to threaten conclusions about whether the exposure has an important effect on the outcome?**	**No**	

Y = Yes; PY = Probably yes; SN = Strong no; WN = Weak no; NA = Not applicable; NI = No information

Domain 3: Risk of bias in selection of participants into the study (or into the analysis)

Signalling questions	Response options	Comments
3.1 Did follow-up begin at (or close to) the start of the exposure window for most participants? [*The exposure window is specified in D3*]	Y	
3.2 **If N/PN to 3.1**: Is the effect of exposure likely to be constant over the period of follow up analysed?	NA	
3.3 Was selection of participants into the study (or into the analysis) based on participant characteristics observed after the start of the exposure window being studied? [*The exposure window is specified in D3*]	N	
3.4 **If Y/PY to 3.3**: Were these characteristics likely to be influenced by exposure or a cause of exposure?	NA	
3.5 **If Y/PY to 3.4**: Were these characteristics likely to be influenced by the outcome or a cause of the outcome?	NA	
3.6 **If N/PN to 3.2 or Y/PY to 3.5**: Is it likely that the analysis corrected for all of the potential selection biases identified in A and B above?	NA	
3.7 **If N/PN to 3.2 or Y/PY to 3.5**: Did sensitivity analyses demonstrate that the likely impact of the potential selection biases identified in A or B above was minimal?	NA	
Risk of bias (due to selection of participants into the study) in the estimated effect of exposure on the outcome	Low risk	
What is the predicted direction of bias due to selection of participants into the study?	Towards null	
Is the risk of bias (due to selection of participants into the study) sufficiently high, in the context of its likely direction and the magnitude of the estimated exposure effect, to threaten conclusions about whether the exposure has an important effect on the outcome?	No	

Y = Yes; PY = Probably yes; PN = Probably no; N = No; SN = Strong no; WN = Weak no; NA = Not applicable; NI = No information

Domain 4: Risk of bias due to post-exposure interventions

Signalling questions	Response options	Comments
4.1 Were there post-exposure interventions that were influenced by prior exposure during the follow-up period?	N	
4.2 **If Y/PY to 4.1**: Is it likely that the analysis corrected for the effect of post-exposure interventions that were influenced by prior exposure?	NA	
Risk of bias (due post-exposure interventions) in the estimated effect of exposure on the outcome	Low risk	
What is the predicted direction of bias due to confounding?	Towards null	
Is the risk of bias (due post-exposure interventions) sufficiently high, in the context of its likely direction and the magnitude of the estimated exposure effect, to threaten conclusions about whether the exposure has an important effect on the outcome?	No	

Y = Yes; PY = Probably yes; PN = Probably no; N = No; NA = Not applicable; NI = No information

Domain 5: Risk of bias due to missing data

Signalling questions	Response options	Comments
5.1 Were complete data on exposure status available for all, or nearly all, participants?	Y	
5.2 Were complete data on the outcome available for all, or nearly all, participants?	Y	
5.3 Were complete data on confounding variables available for all, or nearly all, participants?	Y	
5.4 **If N/PN/NI to 5.1, 5.2 or 5.3**: Is the result based on a complete case analysis?	NA	
5.5 **If Y/PY/NI**: Was exclusion from the analysis because of missing data (in exposure, confounders or the outcome) likely to be related to the true value of the outcome?	N	
5.6 **If N/PN to 5.5**: Were all or most predictors of missingness (in exposure, confounders or the outcome) included in the analysis model?	SY	
5.7 **If N/PN to 5.4**: Was the analysis based on imputing missing values?	NA	
5.8 **If Y/PY to 5.7**: Was imputation performed appropriately?	NA	
5.9 **If N/PN to 5.7**: Was an appropriate alternative method used to correct for bias due to missing data?	Y	
5.10 **If PN/N/NI to 5.1, 5.2 or 5.3**: Is there evidence that the result was not biased by missing data?	NA	
Risk of bias (due to missing data) in the estimated effect of exposure on the outcome	Low risk	
What is the predicted direction of bias due to missing data?	Towards null	
Is the risk of bias (due to missing data) sufficiently high, in the context of its likely direction and the magnitude of the estimated exposure effect, to threaten conclusions about whether the exposure has an important effect on the outcome?	No	

Y = Yes; PY = Probably yes; PN = Probably no; N = No; SY = Strong yes; WY = Weak yes; NA = Not applicable; NI = No information

Domain 6: Risk of bias arising from measurement of the outcome

Signalling questions	Response options	Comments
6.1 Could measurement or ascertainment of the outcome have differed between exposure groups or levels of exposure?	N	
6.2 Were outcome assessors aware of study participants' exposure history?	N	
6.3 **If Y/PY/NI to 6.2**: Could assessment of the outcome have been influenced by knowledge of participants' exposure history?	NA	
Risk of bias (arising from measurement of outcomes) in the estimated effect of exposure on the outcome	Low risk	
What is the predicted direction of bias arising from measurement of outcomes?	Towards null	
Is the risk of bias (arising from measurement of outcomes) sufficiently high, in the context of its likely direction and the magnitude of the estimated exposure effect, to threaten conclusions about whether the exposure has an important effect on the outcome?	No	

Y = Yes; PY = Probably yes; PN = Probably no; N = No; SY = Strong yes; WY = Weak yes; NA = Not applicable; NI = No information

Domain 7: Risk of bias in selection of the reported result

Signalling questions	Response options	Comments
7.1 Was the result reported in accordance with an available, pre-determined analysis plan?	Y	
7.2 **If N/PN/NI to 7.1**: Is the reported effect estimate likely to be selected, based on desirability of the magnitude (or statistical significance) of the estimated effect of exposure on outcome, from multiple *exposure measurements* within the exposure domain?	N	
7.3 Is the reported effect estimate likely to be selected, based on desirability of the magnitude (or statistical significance) of the estimated effect of exposure on outcome, from multiple *outcome measurements* within the outcome domain?	N	
7.4 Is the reported effect estimate likely to be selected, based on desirability of the magnitude (or statistical significance) of the estimated effect of exposure on outcome, from multiple *analyses* of the exposure-outcome relationship?	N	
7.5 Is the reported effect estimate likely to be selected, based on the basis of desirability of the results (e.g. statistical significance), from different *subgroups*?	N	
Risk of bias (due to selection of the reported result) in the estimated effect of exposure on the outcome	Low risk	
What is the predicted direction of bias due to selection of the reported result?	Towards null	
Is the risk of bias (due to selection of the reported result) sufficiently high, in the context of its likely direction and the magnitude of the estimated exposure effect, to threaten conclusions about whether the exposure has an important effect on the outcome?	No	

Y = Yes; PY = Probably yes; PN = Probably no; N = No; NA = Not applicable; NI = No information

Overall risk of bias

	Response options	Comments
Overall risk of bias	Low risk of bias	
What is the predicted direction of bias?	Towards null	
Is the overall risk of bias sufficiently high, in the context of its likely direction and the magnitude of the estimated exposure effect, to threaten conclusions about whether the exposure has an important effect on the outcome?	No	

[21] Sharma, JB.; Sharma, S. Role of thioridazine in unexplained infertility. *Int. J. Gynaecol. Obstet.* **1992**, *37*(1), 37-41. doi: 10.1016/0020-7292(92)90975-o.

Domain 1: Risk of bias due to confounding*, variant (b): **If Y/PY to C7 and Y/PY to C8** (the analysis was based on splitting participants' follow up time according to exposure status and/or magnitude and changes in exposure status and/or magnitude likely to be related to factors that are predictive of the outcome, so both baseline and time-varying confounding need to be addressed)*

Signalling questions	Response options	Comments
1.1 Did the authors use an analysis method that was appropriate to control for time-varying as well as baseline confounding?	Y	
1.2 **If Y/PY to 1.1**: Did the authors control for all the important baseline and time-varying confounding factors for which this was necessary?	Y	
1.3 **If Y/PY/WN to 1.2**: Were confounding factors that were controlled for (and for which control was necessary) measured validly and reliably by the variables available in this study?	Y	
1.4 **If N/PN/NI to 1.1**: Did the authors control for time-varying factors or other variables measured after the start of the exposure window being studied?	NA	
1.5 Did the use of negative controls, or other considerations, suggest uncontrolled confounding?	PN	
Risk of bias (due to confounding) in the estimated effect of exposure on the outcome	Low risk	
What is the predicted direction of bias due to confounding?	Towards null	
Is the risk of bias (due to confounding) sufficiently high, in the context of its likely direction and the magnitude of the estimated exposure effect, to threaten conclusions about whether the exposure has an important effect on the outcome?	No	

Y = Yes; PY = Probably yes; PN = Probably no; N = No; SY = Strong yes; WY = Weak yes; SN = Strong no; WN = Weak no; NA = Not applicable; NI = No information

Domain 2: Risk of bias arising from measurement of the exposure *Variant (b): **If Y/PY to C5 and Y/PY to C6** (each individual's exposure level was estimated from measurements made at multiple time points)*

Signalling questions	Response options	Comments
**2.1 Does the measured exposure (derived from measurements at multiple time points) well-characterize the exposure metric specified to be of interest in this study? [*This was specified in the answers to D2, D3 and D4*]**	**Y**	
**2.2 Was there error in measurement, or misclassification, of the exposure, at each single time point?**	**PN**	
**2.3 If SY/WY to 2.2: Could mismeasurement or misclassification of exposure have been differential (i.e. related to the outcome or risk of the outcome)?**	**NA**	
**2.4 If SY/WY to 2.2 and N/PN/WY to 2.3: Is the nature of the (non-differential) measurement error likely to bias the estimated effect of exposure on outcome?**	**NA**	
**Risk of bias (arising from measurement of exposure) in the estimated effect of exposure on the outcome**	**Low risk**	
**What is the predicted direction of bias arising from measurement of exposure?**	**Towards null**	
**Is the risk of bias (arising from measurement of exposure) sufficiently high, in the context of its likely direction and the magnitude of the estimated exposure effect, to threaten conclusions about whether the exposure has an important effect on the outcome?**	**No**	

Y = Yes; PY = Probably yes; SN = Strong no; WN = Weak no; NA = Not applicable; NI = No information

Domain 3: Risk of bias in selection of participants into the study (or into the analysis)

Signalling questions	Response options	Comments
3.1 Did follow-up begin at (or close to) the start of the exposure window for most participants? [*The exposure window is specified in D3*]	Y	
3.2 **If N/PN to 3.1**: Is the effect of exposure likely to be constant over the period of follow up analysed?	NA	
3.3 Was selection of participants into the study (or into the analysis) based on participant characteristics observed after the start of the exposure window being studied? [*The exposure window is specified in D3*]	N	
3.4 **If Y/PY to 3.3**: Were these characteristics likely to be influenced by exposure or a cause of exposure?	NA	
3.5 **If Y/PY to 3.4**: Were these characteristics likely to be influenced by the outcome or a cause of the outcome?	NA	
3.6 **If N/PN to 3.2 or Y/PY to 3.5**: Is it likely that the analysis corrected for all of the potential selection biases identified in A and B above?	NA	
3.7 **If N/PN to 3.2 or Y/PY to 3.5**: Did sensitivity analyses demonstrate that the likely impact of the potential selection biases identified in A or B above was minimal?	NA	
Risk of bias (due to selection of participants into the study) in the estimated effect of exposure on the outcome	Low risk	
What is the predicted direction of bias due to selection of participants into the study?	Towards null	
Is the risk of bias (due to selection of participants into the study) sufficiently high, in the context of its likely direction and the magnitude of the estimated exposure effect, to threaten conclusions about whether the exposure has an important effect on the outcome?	No	

Y = Yes; PY = Probably yes; PN = Probably no; N = No; SN = Strong no; WN = Weak no; NA = Not applicable; NI = No information

Domain 4: Risk of bias due to post-exposure interventions

Signalling questions	Response options	Comments
4.1 Were there post-exposure interventions that were influenced by prior exposure during the follow-up period?	N	
4.2 **If Y/PY to 4.1**: Is it likely that the analysis corrected for the effect of post-exposure interventions that were influenced by prior exposure?	NA	
Risk of bias (due post-exposure interventions) in the estimated effect of exposure on the outcome	Low risk	
What is the predicted direction of bias due to confounding?	Towards null	
Is the risk of bias (due post-exposure interventions) sufficiently high, in the context of its likely direction and the magnitude of the estimated exposure effect, to threaten conclusions about whether the exposure has an important effect on the outcome?	No	

Y = Yes; PY = Probably yes; PN = Probably no; N = No; NA = Not applicable; NI = No information

Domain 5: Risk of bias due to missing data

Signalling questions	Response options	Comments
5.1 Were complete data on exposure status available for all, or nearly all, participants?	Y	
5.2 Were complete data on the outcome available for all, or nearly all, participants?	Y	
5.3 Were complete data on confounding variables available for all, or nearly all, participants?	Y	
5.4 **If N/PN/NI to 5.1, 5.2 or 5.3**: Is the result based on a complete case analysis?	NA	
5.5 **If Y/PY/NI**: Was exclusion from the analysis because of missing data (in exposure, confounders or the outcome) likely to be related to the true value of the outcome?	N	
5.6 **If N/PN to 5.5**: Were all or most predictors of missingness (in exposure, confounders or the outcome) included in the analysis model?	SY	
5.7 **If N/PN to 5.4**: Was the analysis based on imputing missing values?	NA	
5.8 **If Y/PY to 5.7**: Was imputation performed appropriately?	NA	
5.9 **If N/PN to 5.7**: Was an appropriate alternative method used to correct for bias due to missing data?	Y	
5.10 **If PN/N/NI to 5.1, 5.2 or 5.3**: Is there evidence that the result was not biased by missing data?	NA	
Risk of bias (due to missing data) in the estimated effect of exposure on the outcome	Low risk	

Signalling questions	Response options	Comments
What is the predicted direction of bias due to missing data?	Towards null	
Is the risk of bias (due to missing data) sufficiently high, in the context of its likely direction and the magnitude of the estimated exposure effect, to threaten conclusions about whether the exposure has an important effect on the outcome?	No	

Y = Yes; PY = Probably yes; PN = Probably no; N = No; SY = Strong yes; WY = Weak yes; NA = Not applicable; NI = No information

Domain 6: Risk of bias arising from measurement of the outcome

Signalling questions	Response options	Comments
6.1 Could measurement or ascertainment of the outcome have differed between exposure groups or levels of exposure?	N	
6.2 Were outcome assessors aware of study participants' exposure history?	N	
6.3 **If Y/PY/NI to 6.2**: Could assessment of the outcome have been influenced by knowledge of participants' exposure history?	NA	
Risk of bias (arising from measurement of outcomes) in the estimated effect of exposure on the outcome	Low risk	
What is the predicted direction of bias arising from measurement of outcomes?	Towards null	
Is the risk of bias (arising from measurement of outcomes) sufficiently high, in the context of its likely direction and the magnitude of the estimated exposure effect, to threaten conclusions about whether the exposure has an important effect on the outcome?	No	

Y = Yes; PY = Probably yes; PN = Probably no; N = No; SY = Strong yes; WY = Weak yes; NA = Not applicable; NI = No information

Domain 7: Risk of bias in selection of the reported result

Signalling questions	Response options	Comments
7.1 Was the result reported in accordance with an available, pre-determined analysis plan?	Y	
7.2 **If N/PN/NI to 7.1**: Is the reported effect estimate likely to be selected, based on desirability of the magnitude (or statistical significance) of the estimated effect of exposure on outcome, from multiple *exposure measurements* within the exposure domain?	N	
7.3 Is the reported effect estimate likely to be selected, based on desirability of the magnitude (or statistical significance) of the estimated effect of exposure on outcome, from multiple *outcome measurements* within the outcome domain?	N	
7.4 Is the reported effect estimate likely to be selected, based on desirability of the magnitude (or statistical significance) of the estimated effect of exposure on outcome, from multiple *analyses* of the exposure-outcome relationship?	N	
7.5 Is the reported effect estimate likely to be selected, based on the basis of desirability of the results (e.g. statistical significance), from different *subgroups*?	N	
Risk of bias (due to selection of the reported result) in the estimated effect of exposure on the outcome	Low risk	
What is the predicted direction of bias due to selection of the reported result?	Towards null	
Is the risk of bias (due to selection of the reported result) sufficiently high, in the context of its likely direction and the magnitude of the estimated exposure effect, to threaten conclusions about whether the exposure has an important effect on the outcome?	No	

Y = Yes; PY = Probably yes; PN = Probably no; N = No; NA = Not applicable; NI = No information

Overall risk of bias

	Response options	Comments
Overall risk of bias	Low risk of bias	
What is the predicted direction of bias?	Towards null	
Is the overall risk of bias sufficiently high, in the context of its likely direction and the magnitude of the estimated exposure effect, to threaten conclusions about whether the exposure has an important effect on the outcome?	No	

[22] Goldstein, D.J.; Corbin, L.A.; Fung, M.C. Olanzapine-exposed pregnancies and lactation: early experience. *J. Clin. Psychopharmacol.* **2000**, *20*(4), 399-403.doi: 10.1097/00004714-200008000-00002.

Domain 1: Risk of bias due to confounding, *variant (b): **If Y/PY to C7 and Y/PY to C8** (the analysis was based on splitting participants' follow up time according to exposure status and/or magnitude and changes in exposure status and/or magnitude likely to be related to factors that are predictive of the outcome, so both baseline and time-varying confounding need to be addressed)*

Signalling questions	Response options	Comments
1.1 Did the authors use an analysis method that was appropriate to control for time-varying as well as baseline confounding?	Y	
1.2 **If Y/PY to 1.1**: Did the authors control for all the important baseline and time-varying confounding factors for which this was necessary?	Y	
1.3 **If Y/PY/WN to 1.2**: Were confounding factors that were controlled for (and for which control was necessary) measured validly and reliably by the variables available in this study?	Y	
1.4 **If N/PN/NI to 1.1**: Did the authors control for time-varying factors or other variables measured after the start of the exposure window being studied?	NA	
1.5 Did the use of negative controls, or other considerations, suggest uncontrolled confounding?	N	
Risk of bias (due to confounding) in the estimated effect of exposure on the outcome	Low risk	
What is the predicted direction of bias due to confounding?	Towards null	
Is the risk of bias (due to confounding) sufficiently high, in the context of its likely direction and the magnitude of the estimated exposure effect, to threaten conclusions about whether the exposure has an important effect on the outcome?	No	

Y = Yes; PY = Probably yes; PN = Probably no; N = No; SY = Strong yes; WY = Weak yes; SN = Strong no; WN = Weak no; NA = Not applicable; NI = No information

Domain 2: Risk of bias arising from measurement of the exposure *Variant (b): If Y/PY to C5 and Y/PY to C6 (each individual's exposure level was estimated from measurements made at multiple time points)*

Signalling questions	Response options	Comments
**2.1 Does the measured exposure (derived from measurements at multiple time points) well-characterize the exposure metric specified to be of interest in this study? [*This was specified in the answers to D2, D3 and D4*]**	**Y**	
**2.2 Was there error in measurement, or misclassification, of the exposure, at each single time point?**	**N**	
**2.3 If SY/WY to 2.2: Could mismeasurement or misclassification of exposure have been differential (i.e. related to the outcome or risk of the outcome)?**	**NA**	
**2.4 If SY/WY to 2.2 and N/PN/WY to 2.3: Is the nature of the (non-differential) measurement error likely to bias the estimated effect of exposure on outcome?**	**NA**	
**Risk of bias (arising from measurement of exposure) in the estimated effect of exposure on the outcome**	**Low risk**	
**What is the predicted direction of bias arising from measurement of exposure?**	**Towards null**	
**Is the risk of bias (arising from measurement of exposure) sufficiently high, in the context of its likely direction and the magnitude of the estimated exposure effect, to threaten conclusions about whether the exposure has an important effect on the outcome?**	**No**	

Y = Yes; PY = Probably yes; SN = Strong no; WN = Weak no; NA = Not applicable; NI = No information

Domain 3: Risk of bias in selection of participants into the study (or into the analysis)

Signalling questions	Response options	Comments
3.1 Did follow-up begin at (or close to) the start of the exposure window for most participants? [*The exposure window is specified in D3*]	Y	
3.2 **If N/PN to 3.1**: Is the effect of exposure likely to be constant over the period of follow up analysed?	NA	
3.3 Was selection of participants into the study (or into the analysis) based on participant characteristics observed after the start of the exposure window being studied? [*The exposure window is specified in D3*]	N	
3.4 **If Y/PY to 3.3**: Were these characteristics likely to be influenced by exposure or a cause of exposure?	NA	
3.5 **If Y/PY to 3.4**: Were these characteristics likely to be influenced by the outcome or a cause of the outcome?	NA	
3.6 **If N/PN to 3.2 or Y/PY to 3.5**: Is it likely that the analysis corrected for all of the potential selection biases identified in A and B above?	NA	
3.7 **If N/PN to 3.2 or Y/PY to 3.5**: Did sensitivity analyses demonstrate that the likely impact of the potential selection biases identified in A or B above was minimal?	NA	
Risk of bias (due to selection of participants into the study) in the estimated effect of exposure on the outcome	Low risk	
What is the predicted direction of bias due to selection of participants into the study?	Towards null	
Is the risk of bias (due to selection of participants into the study) sufficiently high, in the context of its likely direction and the magnitude of the estimated exposure effect, to threaten conclusions about whether the exposure has an important effect on the outcome?	No	

Y = Yes; PY = Probably yes; PN = Probably no; N = No; SN = Strong no; WN = Weak no; NA = Not applicable; NI = No information

Domain 4: Risk of bias due to post-exposure interventions

Signalling questions	Response options	Comments
4.1 Were there post-exposure interventions that were influenced by prior exposure during the follow-up period?	N	
4.2 **If Y/PY to 4.1**: Is it likely that the analysis corrected for the effect of post-exposure interventions that were influenced by prior exposure?	NA	
Risk of bias (due post-exposure interventions) in the estimated effect of exposure on the outcome	Low risk	
What is the predicted direction of bias due to confounding?	Towards null	
Is the risk of bias (due post-exposure interventions) sufficiently high, in the context of its likely direction and the magnitude of the estimated exposure effect, to threaten conclusions about whether the exposure has an important effect on the outcome?	No	

Y = Yes; PY = Probably yes; PN = Probably no; N = No; NA = Not applicable; NI = No information

Domain 5: Risk of bias due to missing data

Signalling questions	Response options	Comments

Signalling questions	Response options	Comments
5.1 Were complete data on exposure status available for all, or nearly all, participants?	Y	
5.2 Were complete data on the outcome available for all, or nearly all, participants?	Y	
5.3 Were complete data on confounding variables available for all, or nearly all, participants?	Y	
5.4 **If N/PN/NI to 5.1, 5.2 or 5.3**: Is the result based on a complete case analysis?	NA	
5.5 **If Y/PY/NI**: Was exclusion from the analysis because of missing data (in exposure, confounders or the outcome) likely to be related to the true value of the outcome?	N	
5.6 **If N/PN to 5.5**: Were all or most predictors of missingness (in exposure, confounders or the outcome) included in the analysis model?	SY	
5.7 **If N/PN to 5.4**: Was the analysis based on imputing missing values?	NA	
5.8 **If Y/PY to 5.7**: Was imputation performed appropriately?	NA	
5.9 **If N/PN to 5.7**: Was an appropriate alternative method used to correct for bias due to missing data?	Y	
5.10 **If PN/N/NI to 5.1, 5.2 or 5.3**: Is there evidence that the result was not biased by missing data?	NA	
Risk of bias (due to missing data) in the estimated effect of exposure on the outcome	Low risk	
What is the predicted direction of bias due to missing data?	Towards null	
Is the risk of bias (due to missing data) sufficiently high, in the context of its likely direction and the magnitude of the estimated exposure effect, to threaten conclusions about whether the exposure has an important effect on the outcome?	No	

Y = Yes; PY = Probably yes; PN = Probably no; N = No; SY = Strong yes; WY = Weak yes; NA = Not applicable; NI = No information

Domain 6: Risk of bias arising from measurement of the outcome

Signalling questions	Response options	Comments
6.1 Could measurement or ascertainment of the outcome have differed between exposure groups or levels of exposure?	N	
6.2 Were outcome assessors aware of study participants' exposure history?	N	
6.3 **If Y/PY/NI to 6.2**: Could assessment of the outcome have been influenced by knowledge of participants' exposure history?	NA	
Risk of bias (arising from measurement of outcomes) in the estimated effect of exposure on the outcome	Low risk	
What is the predicted direction of bias arising from measurement of outcomes?	Towards null	
Is the risk of bias (arising from measurement of outcomes) sufficiently high, in the context of its likely direction and the magnitude of the estimated exposure effect, to threaten conclusions about whether the exposure has an important effect on the outcome?	No	

Y = Yes; PY = Probably yes; PN = Probably no; N = No; SY = Strong yes; WY = Weak yes; NA = Not applicable; NI = No information

Domain 7: Risk of bias in selection of the reported result

Signalling questions	Response options	Comments
7.1 Was the result reported in accordance with an available, pre-determined analysis plan?	Y	
7.2 **If N/PN/NI to 7.1**: Is the reported effect estimate likely to be selected, based on desirability of the magnitude (or statistical significance) of the estimated effect of exposure on outcome, from multiple *exposure measurements* within the exposure domain?	N	
7.3 Is the reported effect estimate likely to be selected, based on desirability of the magnitude (or statistical significance) of the estimated effect of exposure on outcome, from multiple *outcome measurements* within the outcome domain?	N	
7.4 Is the reported effect estimate likely to be selected, based on desirability of the magnitude (or statistical significance) of the estimated effect of exposure on outcome, from multiple *analyses* of the exposure-outcome relationship?	N	
7.5 Is the reported effect estimate likely to be selected, based on the basis of desirability of the results (e.g. statistical significance), from different *subgroups*?	N	
Risk of bias (due to selection of the reported result) in the estimated effect of exposure on the outcome	Low risk	
What is the predicted direction of bias due to selection of the reported result?	Towards null	
Is the risk of bias (due to selection of the reported result) sufficiently high, in the context of its likely direction and the magnitude of the estimated exposure effect, to threaten conclusions about whether the exposure has an important effect on the outcome?	No	

Y = Yes; PY = Probably yes; PN = Probably no; N = No; NA = Not applicable; NI = No information

Overall risk of bias

	Response options	Comments
Overall risk of bias	Low risk of bias	
What is the predicted direction of bias?	Towards null	
Is the overall risk of bias sufficiently high, in the context of its likely direction and the magnitude of the estimated exposure effect, to threaten conclusions about whether the exposure has an important effect on the outcome?	No	

[23] Diav-Citrin, O.; Shechtman, S.; Ornoy, S.; Arnon, J.; Schaefer, C.; Garbis, H,Clementi, M.; Ornoy, A. Safety of haloperidol and penfluridol in pregnancy: a multicenter, prospective, controlled study. *J. Clin. Psychiatry* **2005**, *66*(3), 317-322. doi: 10.4088/jcp.v66n0307.

Domain 1: Risk of bias due to confounding*, variant (b): **If Y/PY to C7 and Y/PY to C8** (the analysis was based on splitting participants' follow up time according to exposure status and/or magnitude and changes in exposure status and/or magnitude likely to be related to factors that are predictive of the outcome, so both baseline and time-varying confounding need to be addressed)*

Signalling questions	Response options	Comments
1.1 Did the authors use an analysis method that was appropriate to control for time-varying as well as baseline confounding?	Y	
1.2 **If Y/PY to 1.1**: Did the authors control for all the important baseline and time-varying confounding factors for which this was necessary?	Y	
1.3 **If Y/PY/WN to 1.2**: Were confounding factors that were controlled for (and for which control was necessary) measured validly and reliably by the variables available in this study?	Y	
1.4 **If N/PN/NI to 1.1**: Did the authors control for time-varying factors or other variables measured after the start of the exposure window being studied?	NA	
1.5 Did the use of negative controls, or other considerations, suggest uncontrolled confounding?	N	
Risk of bias (due to confounding) in the estimated effect of exposure on the outcome	Low risk	
What is the predicted direction of bias due to confounding?	Towards null	
Is the risk of bias (due to confounding) sufficiently high, in the context of its likely direction and the magnitude of the estimated exposure effect, to threaten conclusions about whether the exposure has an important effect on the outcome?	No	

Y = Yes; PY = Probably yes; PN = Probably no; N = No; SY = Strong yes; WY = Weak yes; SN = Strong no; WN = Weak no; NA = Not applicable; NI = No information

Domain 2: Risk of bias arising from measurement of the exposure *Variant (b): **If Y/PY to C5 and Y/PY to C6** (each individual's exposure level was estimated from measurements made at multiple time points)*

Signalling questions	Response options	Comments
**2.1 Does the measured exposure (derived from measurements at multiple time points) well-characterize the exposure metric specified to be of interest in this study? [*This was specified in the answers to D2, D3 and D4*]**	**Y**	
**2.2 Was there error in measurement, or misclassification, of the exposure, at each single time point?**	**N**	
**2.3 If SY/WY to 2.2: Could mismeasurement or misclassification of exposure have been differential (i.e. related to the outcome or risk of the outcome)?**	**NA**	
**2.4 If SY/WY to 2.2 and N/PN/WY to 2.3: Is the nature of the (non-differential) measurement error likely to bias the estimated effect of exposure on outcome?**	**NA**	
**Risk of bias (arising from measurement of exposure) in the estimated effect of exposure on the outcome**	**Low risk**	
**What is the predicted direction of bias arising from measurement of exposure?**	**Towards null**	
**Is the risk of bias (arising from measurement of exposure) sufficiently high, in the context of its likely direction and the magnitude of the estimated exposure effect, to threaten conclusions about whether the exposure has an important effect on the outcome?**	**No**	

Y = Yes; PY = Probably yes; SN = Strong no; WN = Weak no; NA = Not applicable; NI = No information

Domain 3: Risk of bias in selection of participants into the study (or into the analysis)

Signalling questions	Response options	Comments
3.1 Did follow-up begin at (or close to) the start of the exposure window for most participants? [*The exposure window is specified in D3*]	Y	
3.2 **If N/PN to 3.1**: Is the effect of exposure likely to be constant over the period of follow up analysed?	NA	
3.3 Was selection of participants into the study (or into the analysis) based on participant characteristics observed after the start of the exposure window being studied? [*The exposure window is specified in D3*]	N	
3.4 **If Y/PY to 3.3**: Were these characteristics likely to be influenced by exposure or a cause of exposure?	NA	
3.5 **If Y/PY to 3.4**: Were these characteristics likely to be influenced by the outcome or a cause of the outcome?	NA	
3.6 **If N/PN to 3.2 or Y/PY to 3.5**: Is it likely that the analysis corrected for all of the potential selection biases identified in A and B above?	NA	
3.7 **If N/PN to 3.2 or Y/PY to 3.5**: Did sensitivity analyses demonstrate that the likely impact of the potential selection biases identified in A or B above was minimal?	NA	
Risk of bias (due to selection of participants into the study) in the estimated effect of exposure on the outcome	Low risk	
What is the predicted direction of bias due to selection of participants into the study?	Towards null	
Is the risk of bias (due to selection of participants into the study) sufficiently high, in the context of its likely direction and the magnitude of the estimated exposure effect, to threaten conclusions about whether the exposure has an important effect on the outcome?	No	

Y = Yes; PY = Probably yes; PN = Probably no; N = No; SN = Strong no; WN = Weak no; NA = Not applicable; NI = No information

Domain 4: Risk of bias due to post-exposure interventions

Signalling questions	Response options	Comments
4.1 Were there post-exposure interventions that were influenced by prior exposure during the follow-up period?	N	
4.2 **If Y/PY to 4.1**: Is it likely that the analysis corrected for the effect of post-exposure interventions that were influenced by prior exposure?	NA	
Risk of bias (due post-exposure interventions) in the estimated effect of exposure on the outcome	Low risk	
What is the predicted direction of bias due to confounding?	Towards null	
Is the risk of bias (due post-exposure interventions) sufficiently high, in the context of its likely direction and the	No	

magnitude of the estimated exposure effect, to threaten conclusions about whether the exposure has an important effect on the outcome?		

Y = Yes; PY = Probably yes; PN = Probably no; N = No; NA = Not applicable; NI = No information

Domain 5: Risk of bias due to missing data

Signalling questions	Response options	Comments
5.1 Were complete data on exposure status available for all, or nearly all, participants?	Y	
5.2 Were complete data on the outcome available for all, or nearly all, participants?	Y	
5.3 Were complete data on confounding variables available for all, or nearly all, participants?	Y	
5.4 **If N/PN/NI to 5.1, 5.2 or 5.3**: Is the result based on a complete case analysis?	NA	
5.5 **If Y/PY/NI**: Was exclusion from the analysis because of missing data (in exposure, confounders or the outcome) likely to be related to the true value of the outcome?	N	
5.6 **If N/PN to 5.5**: Were all or most predictors of missingness (in exposure, confounders or the outcome) included in the analysis model?	SY	
5.7 **If N/PN to 5.4**: Was the analysis based on imputing missing values?	NA	
5.8 **If Y/PY to 5.7**: Was imputation performed appropriately?	NA	
5.9 **If N/PN to 5.7**: Was an appropriate alternative method used to correct for bias due to missing data?	Y	
5.10 **If PN/N/NI to 5.1, 5.2 or 5.3**: Is there evidence that the result was not biased by missing data?	NA	
Risk of bias (due to missing data) in the estimated effect of exposure on the outcome	Low risk	
What is the predicted direction of bias due to missing data?	Towards null	
Is the risk of bias (due to missing data) sufficiently high, in the context of its likely direction and the magnitude of the estimated exposure effect, to threaten conclusions about whether the exposure has an important effect on the outcome?	No	

Y = Yes; PY = Probably yes; PN = Probably no; N = No; SY = Strong yes; WY = Weak yes; NA = Not applicable; NI = No information

Domain 6: Risk of bias arising from measurement of the outcome

Signalling questions	Response options	Comments
6.1 Could measurement or ascertainment of the outcome have differed between exposure groups or levels of exposure?	N	
6.2 Were outcome assessors aware of study participants' exposure history?	N	
6.3 **If Y/PY/NI to 6.2**: Could assessment of the outcome have been influenced by knowledge of participants' exposure history?	NA	
Risk of bias (arising from measurement of outcomes) in the estimated effect of exposure on the outcome	Low risk	
What is the predicted direction of bias arising from measurement of outcomes?	Towards null	
Is the risk of bias (arising from measurement of outcomes) sufficiently high, in the context of its likely direction and the magnitude of the estimated exposure effect, to threaten conclusions about whether the exposure has an important effect on the outcome?	No	

Y = Yes; PY = Probably yes; PN = Probably no; N = No; SY = Strong yes; WY = Weak yes; NA = Not applicable; NI = No information

Domain 7: Risk of bias in selection of the reported result

Signalling questions	Response options	Comments
7.1 Was the result reported in accordance with an available, pre-determined analysis plan?	Y	
7.2 **If N/PN/NI to 7.1**: Is the reported effect estimate likely to be selected, based on desirability of the magnitude (or statistical significance) of the estimated effect of exposure on outcome, from multiple *exposure measurements* within the exposure domain?	N	
7.3 Is the reported effect estimate likely to be selected, based on desirability of the magnitude (or statistical significance) of the estimated effect of exposure on outcome, from multiple *outcome measurements* within the outcome domain?	N	
7.4 Is the reported effect estimate likely to be selected, based on desirability of the magnitude (or statistical significance) of the estimated effect of exposure on outcome, from multiple *analyses* of the exposure-outcome relationship?	N	
7.5 Is the reported effect estimate likely to be selected, based on the basis of desirability of the results (e.g. statistical significance), from different *subgroups*?	N	
Risk of bias (due to selection of the reported result) in the estimated effect of exposure on the outcome	Low risk	
What is the predicted direction of bias due to selection of the reported result?	Towards null	
Is the risk of bias (due to selection of the reported result) sufficiently high, in the context of its likely direction and the magnitude of the estimated exposure effect, to threaten conclusions about whether the exposure has an important effect on the outcome?	No	

Y = Yes; PY = Probably yes; PN = Probably no; N = No; NA = Not applicable; NI = No information

Overall risk of bias

	Response options	Comments
Overall risk of bias	Low risk of bias	
What is the predicted direction of bias?	Towards null	
Is the overall risk of bias sufficiently high, in the context of its likely direction and the magnitude of the estimated exposure effect, to threaten conclusions about whether the exposure has an important effect on the outcome?	No	

[24] McKenna, K.; Koren, G.; Tetelbaum, M.; Wilton, L.; Shakir, S.; Diav-Citrin, O.; Levinson, A.; Zipursky, R.B.; Einarson, A. Pregnancy outcome of women using atypical antipsychotic drugs: a prospective comparative study. *J. Clin. Psychiatry* **2005**, *66*(4), 444-449; quiz 546. doi: 10.4088/jcp.v66n0406.

Domain 1: Risk of bias due to confounding, *variant (b): **If Y/PY to C7 and Y/PY to C8** (the analysis was based on splitting participants' follow up time according to exposure status and/or magnitude and changes in exposure status and/or magnitude likely to be related to factors that are predictive of the outcome, so both baseline and time-varying confounding need to be addressed)*

Signalling questions	Response options	Comments
1.1 Did the authors use an analysis method that was appropriate to control for time-varying as well as baseline confounding?	Y	
1.2 **If Y/PY to 1.1**: Did the authors control for all the important baseline and time-varying confounding factors for which this was necessary?	Y	
1.3 **If Y/PY/WN to 1.2**: Were confounding factors that were controlled for (and for which control was necessary) measured validly and reliably by the variables available in this study?	PY	
1.4 **If N/PN/NI to 1.1**: Did the authors control for time-varying factors or other variables measured after the start of the exposure window being studied?	NA	
1.5 Did the use of negative controls, or other considerations, suggest uncontrolled confounding?	N	
Risk of bias (due to confounding) in the estimated effect of exposure on the outcome	Low risk	
What is the predicted direction of bias due to confounding?	Towards null	
Is the risk of bias (due to confounding) sufficiently high, in the context of its likely direction and the magnitude of the estimated exposure effect, to threaten conclusions about whether the exposure has an important effect on the outcome?	No	

Y = Yes; PY = Probably yes; PN = Probably no; N = No; SY = Strong yes; WY = Weak yes; SN = Strong no; WN = Weak no; NA = Not applicable; NI = No information

Domain 2: Risk of bias arising from measurement of the exposure *Variant (b): **If Y/PY to C5 and Y/PY to C6** (each individual's exposure level was estimated from measurements made at multiple time points)*

Signalling questions	Response options	Comments
**2.1 Does the measured exposure (derived from measurements at multiple time points) well-characterize the exposure metric specified to be of interest in this study? [*This was specified in the answers to D2, D3 and D4*]**	**PY**	
**2.2 Was there error in measurement, or misclassification, of the exposure, at each single time point?**	**N**	
**2.3 If SY/WY to 2.2: Could mismeasurement or misclassification of exposure have been differential (i.e. related to the outcome or risk of the outcome)?**	**NA**	
**2.4 If SY/WY to 2.2 and N/PN/WY to 2.3: Is the nature of the (non-differential) measurement error likely to bias the estimated effect of exposure on outcome?**	**NA**	
**Risk of bias (arising from measurement of exposure) in the estimated effect of exposure on the outcome**	**Low risk**	
**What is the predicted direction of bias arising from measurement of exposure?**	**Towards null**	
**Is the risk of bias (arising from measurement of exposure) sufficiently high, in the context of its likely direction and the magnitude of the estimated exposure effect, to threaten conclusions about whether the exposure has an important effect on the outcome?**	**No**	

Y = Yes; PY = Probably yes; SN = Strong no; WN = Weak no; NA = Not applicable; NI = No information

Domain 3: Risk of bias in selection of participants into the study (or into the analysis)

Signalling questions	Response options	Comments
3.1 Did follow-up begin at (or close to) the start of the exposure window for most participants? [*The exposure window is specified in D3*]	Y	
3.2 **If N/PN to 3.1**: Is the effect of exposure likely to be constant over the period of follow up analysed?	NA	
3.3 Was selection of participants into the study (or into the analysis) based on participant characteristics observed after the start of the exposure window being studied? [*The exposure window is specified in D3*]	N	
3.4 **If Y/PY to 3.3**: Were these characteristics likely to be influenced by exposure or a cause of exposure?	NA	
3.5 **If Y/PY to 3.4**: Were these characteristics likely to be influenced by the outcome or a cause of the outcome?	NA	
3.6 **If N/PN to 3.2 or Y/PY to 3.5**: Is it likely that the analysis corrected for all of the potential selection biases identified in A and B above?	NA	
3.7 **If N/PN to 3.2 or Y/PY to 3.5**: Did sensitivity analyses demonstrate that the likely impact of the potential selection biases identified in A or B above was minimal?	NA	
Risk of bias (due to selection of participants into the study) in the estimated effect of exposure on the outcome	Low risk	
What is the predicted direction of bias due to selection of participants into the study?	Towards null	
Is the risk of bias (due to selection of participants into the study) sufficiently high, in the context of its likely direction and the magnitude of the estimated exposure effect, to threaten conclusions about whether the exposure has an important effect on the outcome?	No	

Y = Yes; PY = Probably yes; PN = Probably no; N = No; SN = Strong no; WN = Weak no; NA = Not applicable; NI = No information

Domain 4: Risk of bias due to post-exposure interventions

Signalling questions	Response options	Comments
4.1 Were there post-exposure interventions that were influenced by prior exposure during the follow-up period?	N	
4.2 **If Y/PY to 4.1**: Is it likely that the analysis corrected for the effect of post-exposure interventions that were influenced by prior exposure?	NA	
Risk of bias (due post-exposure interventions) in the estimated effect of exposure on the outcome	Low risk	
What is the predicted direction of bias due to confounding?	Towards null	
Is the risk of bias (due post-exposure interventions) sufficiently high, in the context of its likely direction and the magnitude of the estimated exposure effect, to threaten conclusions about whether the exposure has an important effect on the outcome?	No	

Y = Yes; PY = Probably yes; PN = Probably no; N = No; NA = Not applicable; NI = No information

Domain 5: Risk of bias due to missing data

Signalling questions	Response options	Comments
5.1 Were complete data on exposure status available for all, or nearly all, participants?	Y	
5.2 Were complete data on the outcome available for all, or nearly all, participants?	Y	
5.3 Were complete data on confounding variables available for all, or nearly all, participants?	Y	
5.4 **If N/PN/NI to 5.1, 5.2 or 5.3**: Is the result based on a complete case analysis?	NA	
5.5 **If Y/PY/NI**: Was exclusion from the analysis because of missing data (in exposure, confounders or the outcome) likely to be related to the true value of the outcome?	N	
5.6 **If N/PN to 5.5**: Were all or most predictors of missingness (in exposure, confounders or the outcome) included in the analysis model?	SY	
5.7 **If N/PN to 5.4**: Was the analysis based on imputing missing values?	NA	
5.8 **If Y/PY to 5.7**: Was imputation performed appropriately?	NA	
5.9 **If N/PN to 5.7**: Was an appropriate alternative method used to correct for bias due to missing data?	Y	
5.10 **If PN/N/NI to 5.1, 5.2 or 5.3**: Is there evidence that the result was not biased by missing data?	NA	
Risk of bias (due to missing data) in the estimated effect of exposure on the outcome	Low risk	
What is the predicted direction of bias due to missing data?	Towards null	
Is the risk of bias (due to missing data) sufficiently high, in the context of its likely direction and the magnitude of the estimated exposure effect, to threaten conclusions about whether the exposure has an important effect on the outcome?	No	

Y = Yes; PY = Probably yes; PN = Probably no; N = No; SY = Strong yes; WY = Weak yes; NA = Not applicable; NI = No information

Domain 6: Risk of bias arising from measurement of the outcome

Signalling questions	Response options	Comments
6.1 Could measurement or ascertainment of the outcome have differed between exposure groups or levels of exposure?	N	
6.2 Were outcome assessors aware of study participants' exposure history?	PN	
6.3 **If Y/PY/NI to 6.2**: Could assessment of the outcome have been influenced by knowledge of participants' exposure history?	NA	
Risk of bias (arising from measurement of outcomes) in the estimated effect of exposure on the outcome	Low risk	
What is the predicted direction of bias arising from measurement of outcomes?	Towards null	
Is the risk of bias (arising from measurement of outcomes) sufficiently high, in the context of its likely direction and the magnitude of the estimated exposure effect, to threaten conclusions about whether the exposure has an important effect on the outcome?	No	

Y = Yes; PY = Probably yes; PN = Probably no; N = No; SY = Strong yes; WY = Weak yes; NA = Not applicable; NI = No information

Domain 7: Risk of bias in selection of the reported result

Signalling questions	Response options	Comments
7.1 Was the result reported in accordance with an available, pre-determined analysis plan?	Y	
7.2 **If N/PN/NI to 7.1**: Is the reported effect estimate likely to be selected, based on desirability of the magnitude (or statistical significance) of the estimated effect of exposure on outcome, from multiple *exposure measurements* within the exposure domain?	N	
7.3 Is the reported effect estimate likely to be selected, based on desirability of the magnitude (or statistical significance) of the estimated effect of exposure on outcome, from multiple *outcome measurements* within the outcome domain?	N	
7.4 Is the reported effect estimate likely to be selected, based on desirability of the magnitude (or statistical significance) of the estimated effect of exposure on outcome, from multiple *analyses* of the exposure-outcome relationship?	N	
7.5 Is the reported effect estimate likely to be selected, based on the basis of desirability of the results (e.g. statistical significance), from different *subgroups*?	N	
Risk of bias (due to selection of the reported result) in the estimated effect of exposure on the outcome	Low risk	
What is the predicted direction of bias due to selection of the reported result?	Towards null	
Is the risk of bias (due to selection of the reported result) sufficiently high, in the context of its likely direction and the magnitude of the estimated exposure effect, to threaten conclusions about whether the exposure has an important effect on the outcome?	No	

Y = Yes; PY = Probably yes; PN = Probably no; N = No; NA = Not applicable; NI = No information

Overall risk of bias

	Response	Comments

	options	
Overall risk of bias	Low risk of bias	
What is the predicted direction of bias?	Towards null	
Is the overall risk of bias sufficiently high, in the context of its likely direction and the magnitude of the estimated exposure effect, to threaten conclusions about whether the exposure has an important effect on the outcome?	No	

[25] Reis, M.; Källén, B. Maternal use of antipsychotics in early pregnancy and delivery outcome. *J. Clin. Psychopharmacol.* **2008**, *28*(3), 279-288. doi: 10.1097/JCP.0b013e318172b8d5.

Domain 1: Risk of bias due to confounding, *variant (b): **If Y/PY to C7 and Y/PY to C8** (the analysis was based on splitting participants' follow up time according to exposure status and/or magnitude and changes in exposure status and/or magnitude likely to be related to factors that are predictive of the outcome, so both baseline and time-varying confounding need to be addressed)*

Signalling questions	Response options	Comments
1.1 Did the authors use an analysis method that was appropriate to control for time-varying as well as baseline confounding?	Y	
1.2 **If Y/PY to 1.1**: Did the authors control for all the important baseline and time-varying confounding factors for which this was necessary?	PY	
1.3 **If Y/PY/WN to 1.2**: Were confounding factors that were controlled for (and for which control was necessary) measured validly and reliably by the variables available in this study?	Y	
1.4 **If N/PN/NI to 1.1**: Did the authors control for time-varying factors or other variables measured after the start of the exposure window being studied?	NA	
1.5 Did the use of negative controls, or other considerations, suggest uncontrolled confounding?	N	
Risk of bias (due to confounding) in the estimated effect of exposure on the outcome	Low risk	
What is the predicted direction of bias due to confounding?	Towards null	
Is the risk of bias (due to confounding) sufficiently high, in the context of its likely direction and the magnitude of the estimated exposure effect, to threaten conclusions about whether the exposure has an important effect on the outcome?	No	

Y = Yes; PY = Probably yes; PN = Probably no; N = No; SY = Strong yes; WY = Weak yes; SN = Strong no; WN = Weak no; NA = Not applicable; NI = No information

Domain 2: Risk of bias arising from measurement of the exposure *Variant (b): **If Y/PY to C5 and Y/PY to C6** (each individual's exposure level was estimated from measurements made at multiple time points)*

Signalling questions	Response options	Comments
**2.1 Does the measured exposure (derived from measurements at multiple time points) well-characterize the exposure metric specified to be of interest in this study? [*This was specified in the answers to D2, D3 and D4*]**	**Y**	
**2.2 Was there error in measurement, or misclassification, of the exposure, at each single time point?**	**N**	
**2.3 If SY/WY to 2.2: Could mismeasurement or misclassification of exposure have been differential (i.e. related to the outcome or risk of the outcome)?**	**NA**	
**2.4 If SY/WY to 2.2 and N/PN/WY to 2.3: Is the nature of the (non-differential) measurement error likely to bias the estimated effect of exposure on outcome?**	**NA**	
**Risk of bias (arising from measurement of exposure) in the estimated effect of exposure on the outcome**	**Low risk**	
**What is the predicted direction of bias arising from measurement of exposure?**	**Towards null**	
**Is the risk of bias (arising from measurement of exposure) sufficiently high, in the context of its likely direction and the magnitude of the estimated exposure effect, to threaten conclusions about whether the exposure has an important effect on the outcome?**	**No**	

Y = Yes; PY = Probably yes; SN = Strong no; WN = Weak no; NA = Not applicable; NI = No information

Domain 3: Risk of bias in selection of participants into the study (or into the analysis)

Signalling questions	Response options	Comments
3.1 Did follow-up begin at (or close to) the start of the exposure window for most participants? [*The exposure window is specified in D3*]	Y	
3.2 **If N/PN to 3.1**: Is the effect of exposure likely to be constant over the period of follow up analysed?	NA	
3.3 Was selection of participants into the study (or into the analysis) based on participant characteristics observed after the start of the exposure window being studied? [*The exposure window is specified in D3*]	PN	
3.4 **If Y/PY to 3.3**: Were these characteristics likely to be influenced by exposure or a cause of exposure?	NA	
3.5 **If Y/PY to 3.4**: Were these characteristics likely to be influenced by the outcome or a cause of the outcome?	NA	
3.6 **If N/PN to 3.2 or Y/PY to 3.5**: Is it likely that the analysis corrected for all of the potential selection biases identified	NA	

Signalling questions	Response options	Comments
in A and B above?		
3.7 **If N/PN to 3.2 or Y/PY to 3.5**: Did sensitivity analyses demonstrate that the likely impact of the potential selection biases identified in A or B above was minimal?	NA	
Risk of bias (due to selection of participants into the study) in the estimated effect of exposure on the outcome	Low risk	
What is the predicted direction of bias due to selection of participants into the study?	Towards null	
Is the risk of bias (due to selection of participants into the study) sufficiently high, in the context of its likely direction and the magnitude of the estimated exposure effect, to threaten conclusions about whether the exposure has an important effect on the outcome?	No	

Y = Yes; PY = Probably yes; PN = Probably no; N = No; SN = Strong no; WN = Weak no; NA = Not applicable; NI = No information

Domain 4: Risk of bias due to post-exposure interventions

Signalling questions	Response options	Comments
4.1 Were there post-exposure interventions that were influenced by prior exposure during the follow-up period?	N	
4.2 **If Y/PY to 4.1**: Is it likely that the analysis corrected for the effect of post-exposure interventions that were influenced by prior exposure?	NA	
Risk of bias (due post-exposure interventions) in the estimated effect of exposure on the outcome	Low risk	
What is the predicted direction of bias due to confounding?	Towards null	
Is the risk of bias (due post-exposure interventions) sufficiently high, in the context of its likely direction and the magnitude of the estimated exposure effect, to threaten conclusions about whether the exposure has an important effect on the outcome?	No	

Y = Yes; PY = Probably yes; PN = Probably no; N = No; NA = Not applicable; NI = No information

Domain 5: Risk of bias due to missing data

Signalling questions	Response options	Comments
5.1 Were complete data on exposure status available for all, or nearly all, participants?	Y	
5.2 Were complete data on the outcome available for all, or nearly all, participants?	Y	
5.3 Were complete data on confounding variables available for all, or nearly all, participants?	Y	
5.4 **If N/PN/NI to 5.1, 5.2 or 5.3**: Is the result based on a complete case analysis?	NA	
5.5 **If Y/PY/NI**: Was exclusion from the analysis because of missing data (in exposure, confounders or the outcome) likely to be related to the true value of the outcome?	N	
5.6 **If N/PN to 5.5**: Were all or most predictors of missingness (in exposure, confounders or the outcome) included in the analysis model?	SY	
5.7 **If N/PN to 5.4**: Was the analysis based on imputing missing values?	NA	
5.8 **If Y/PY to 5.7**: Was imputation performed appropriately?	NA	
5.9 **If N/PN to 5.7**: Was an appropriate alternative method used to correct for bias due to missing data?	Y	
5.10 **If PN/N/NI to 5.1, 5.2 or 5.3**: Is there evidence that the result was not biased by missing data?	NA	
Risk of bias (due to missing data) in the estimated effect of exposure on the outcome	Low risk	
What is the predicted direction of bias due to missing data?	Towards null	
Is the risk of bias (due to missing data) sufficiently high, in the context of its likely direction and the magnitude of the estimated exposure effect, to threaten conclusions about whether the exposure has an important effect on the outcome?	No	

Y = Yes; PY = Probably yes; PN = Probably no; N = No; SY = Strong yes; WY = Weak yes; NA = Not applicable; NI = No information

Domain 6: Risk of bias arising from measurement of the outcome

Signalling questions	Response options	Comments
6.1 Could measurement or ascertainment of the outcome have differed between exposure groups or levels of exposure?	N	
6.2 Were outcome assessors aware of study participants' exposure history?	N	
6.3 **If Y/PY/NI to 6.2**: Could assessment of the outcome have been influenced by knowledge of participants' exposure history?	NA	
Risk of bias (arising from measurement of outcomes) in the estimated effect of exposure on the outcome	Low risk	
What is the predicted direction of bias arising from measurement of outcomes?	Towards null	
Is the risk of bias (arising from measurement of outcomes) sufficiently high, in the context of its likely direction and the magnitude of the estimated exposure effect, to threaten conclusions about whether the exposure has an important effect on the outcome?	No	

Y = Yes; PY = Probably yes; PN = Probably no; N = No; SY = Strong yes; WY = Weak yes; NA = Not applicable; NI = No information

Domain 7: Risk of bias in selection of the reported result

Signalling questions	Response options	Comments
7.1 Was the result reported in accordance with an available, pre-determined analysis plan?	Y	
7.2 **If N/PN/NI to 7.1**: Is the reported effect estimate likely to be selected, based on desirability of the magnitude (or statistical significance) of the estimated effect of exposure on outcome, from multiple *exposure measurements* within the exposure domain?	N	
7.3 Is the reported effect estimate likely to be selected, based on desirability of the magnitude (or statistical significance) of the estimated effect of exposure on outcome, from multiple *outcome measurements* within the outcome domain?	N	

Signalling questions	Response options	Comments
7.4 Is the reported effect estimate likely to be selected, based on desirability of the magnitude (or statistical significance) of the estimated effect of exposure on outcome, from multiple *analyses* of the exposure-outcome relationship?	PN	
7.5 Is the reported effect estimate likely to be selected, based on the basis of desirability of the results (e.g. statistical significance), from different *subgroups*?	N	
Risk of bias (due to selection of the reported result) in the estimated effect of exposure on the outcome	Low risk	
What is the predicted direction of bias due to selection of the reported result?	Towards null	
Is the risk of bias (due to selection of the reported result) sufficiently high, in the context of its likely direction and the magnitude of the estimated exposure effect, to threaten conclusions about whether the exposure has an important effect on the outcome?	No	

Y = Yes; PY = Probably yes; PN = Probably no; N = No; NA = Not applicable; NI = No information

Overall risk of bias

	Response options	Comments
Overall risk of bias	Low risk of bias	
What is the predicted direction of bias?	Towards null	
Is the overall risk of bias sufficiently high, in the context of its likely direction and the magnitude of the estimated exposure effect, to threaten conclusions about whether the exposure has an important effect on the outcome?	No	

[26] Babu, G.N.; Desai, G.; Tippeswamy, H.; Chandra, P.S. Birth weight and use of olanzapine in pregnancy: a prospective comparative study. *J. Clin. Psychopharmacol.* **2010**, *30*(3), 331-2. doi: 10.1097/JCP.0b013e3181db8734.

Domain 1: Risk of bias due to confounding, *variant (b): **If Y/PY to C7 and Y/PY to C8** (the analysis was based on splitting participants' follow up time according to exposure status and/or magnitude and changes in exposure status and/or magnitude likely to be related to factors that are predictive of the outcome, so both baseline and time-varying confounding need to be addressed)*

Signalling questions	Response options	Comments
1.1 Did the authors use an analysis method that was appropriate to control for time-varying as well as baseline confounding?	Y	
1.2 **If Y/PY to 1.1**: Did the authors control for all the important baseline and time-varying confounding factors for which this was necessary?	Y	
1.3 **If Y/PY/WN to 1.2**: Were confounding factors that were controlled for (and for which control was necessary) measured validly and reliably by the variables available in this study?	Y	
1.4 **If N/PN/NI to 1.1**: Did the authors control for time-varying factors or other variables measured after the start of the exposure window being studied?	NA	
1.5 Did the use of negative controls, or other considerations, suggest uncontrolled confounding?	N	
Risk of bias (due to confounding) in the estimated effect of exposure on the outcome	Low risk	
What is the predicted direction of bias due to confounding?	Towards null	
Is the risk of bias (due to confounding) sufficiently high, in the context of its likely direction and the magnitude of the estimated exposure effect, to threaten conclusions about whether the exposure has an important effect on the outcome?	No	

Y = Yes; PY = Probably yes; PN = Probably no; N = No; SY = Strong yes; WY = Weak yes; SN = Strong no; WN = Weak no; NA = Not applicable; NI = No information

Domain 2: Risk of bias arising from measurement of the exposure *Variant (b): **If Y/PY to C5 and Y/PY to C6** (each individual's exposure level was estimated from measurements made at multiple time points)*

Signalling questions	Response options	Comments
**2.1 Does the measured exposure (derived from measurements at multiple time points) well-characterize the exposure metric specified to be of interest in this study? [*This was specified in the answers to D2, D3 and D4*]**	**Y**	
**2.2 Was there error in measurement, or misclassification, of the exposure, at each single time point?**	**PN**	
**2.3 If SY/WY to 2.2: Could mismeasurement or misclassification of exposure have been differential (i.e. related to the outcome or risk of the outcome)?**	**NA**	
**2.4 If SY/WY to 2.2 and N/PN/WY to 2.3: Is the nature of the (non-differential) measurement error likely to bias the estimated effect of exposure on outcome?**	**NA**	
**Risk of bias (arising from measurement of exposure) in the estimated effect of exposure on the outcome**	**Low risk**	
**What is the predicted direction of bias arising from measurement of exposure?**	**Towards null**	
**Is the risk of bias (arising from measurement of exposure) sufficiently high, in the context of its likely direction and the magnitude of the estimated exposure effect, to threaten conclusions about whether the exposure has an important effect on the outcome?**	**No**	

Y = Yes; PY = Probably yes; SN = Strong no; WN = Weak no; NA = Not applicable; NI = No information

Domain 3: Risk of bias in selection of participants into the study (or into the analysis)

Signalling questions	Response options	Comments
3.1 Did follow-up begin at (or close to) the start of the exposure window for most participants? [*The exposure window is specified in D3*]	Y	
3.2 **If N/PN to 3.1**: Is the effect of exposure likely to be constant over the period of follow up analysed?	NA	
3.3 Was selection of participants into the study (or into the analysis) based on participant characteristics observed after the start of the exposure window being studied? [*The exposure window is specified in D3*]	N	
3.4 **If Y/PY to 3.3**: Were these characteristics likely to be influenced by exposure or a cause of exposure?	NA	
3.5 **If Y/PY to 3.4**: Were these characteristics likely to be influenced by the outcome or a cause of the outcome?	NA	
3.6 **If N/PN to 3.2 or Y/PY to 3.5**: Is it likely that the analysis corrected for all of the potential selection biases identified in A and B above?	NA	
3.7 **If N/PN to 3.2 or Y/PY to 3.5**: Did sensitivity analyses demonstrate that the likely impact of the potential selection biases identified in A or B above was minimal?	NA	
Risk of bias (due to selection of participants into the study) in the estimated effect of exposure on the outcome	Low risk	
What is the predicted direction of bias due to selection of participants into the study?	Towards null	
Is the risk of bias (due to selection of participants into the study) sufficiently high, in the context of its likely direction and the magnitude of the estimated exposure effect, to threaten conclusions about whether the exposure has an important effect on the outcome?	No	

Y = Yes; PY = Probably yes; PN = Probably no; N = No; SN = Strong no; WN = Weak no; NA = Not applicable; NI = No information

Domain 4: Risk of bias due to post-exposure interventions

Signalling questions	Response options	Comments
4.1 Were there post-exposure interventions that were influenced by prior exposure during the follow-up period?	N	
4.2 **If Y/PY to 4.1**: Is it likely that the analysis corrected for the effect of post-exposure interventions that were influenced by prior exposure?	NA	
Risk of bias (due post-exposure interventions) in the estimated effect of exposure on the outcome	Low risk	
What is the predicted direction of bias due to confounding?	Towards null	
Is the risk of bias (due post-exposure interventions) sufficiently high, in the context of its likely direction and the magnitude of the estimated exposure effect, to threaten conclusions about whether the exposure has an important effect on the outcome?	No	

Y = Yes; PY = Probably yes; PN = Probably no; N = No; NA = Not applicable; NI = No information

Domain 5: Risk of bias due to missing data

Signalling questions	Response options	Comments
5.1 Were complete data on exposure status available for all, or nearly all, participants?	Y	
5.2 Were complete data on the outcome available for all, or nearly all, participants?	PY	
5.3 Were complete data on confounding variables available for all, or nearly all, participants?	Y	
5.4 **If N/PN/NI to 5.1, 5.2 or 5.3**: Is the result based on a complete case analysis?	NA	
5.5 **If Y/PY/NI**: Was exclusion from the analysis because of missing data (in exposure, confounders or the outcome) likely to be related to the true value of the outcome?	N	
5.6 **If N/PN to 5.5**: Were all or most predictors of missingness (in exposure, confounders or the outcome) included in the analysis model?	SY	
5.7 **If N/PN to 5.4**: Was the analysis based on imputing missing values?	NA	
5.8 **If Y/PY to 5.7**: Was imputation performed appropriately?	NA	
5.9 **If N/PN to 5.7**: Was an appropriate alternative method used to correct for bias due to missing data?	Y	
5.10 **If PN/N/NI to 5.1, 5.2 or 5.3**: Is there evidence that the result was not biased by missing data?	NA	
Risk of bias (due to missing data) in the estimated effect of exposure on the outcome	Low risk	
What is the predicted direction of bias due to missing data?	Towards null	
Is the risk of bias (due to missing data) sufficiently high, in the context of its likely direction and the magnitude of the estimated exposure effect, to threaten conclusions about whether the exposure has an important effect on the outcome?	No	

Y = Yes; PY = Probably yes; PN = Probably no; N = No; SY = Strong yes; WY = Weak yes; NA = Not applicable; NI = No information

Domain 6: Risk of bias arising from measurement of the outcome

Signalling questions	Response options	Comments
6.1 Could measurement or ascertainment of the outcome have differed between exposure groups or levels of exposure?	N	
6.2 Were outcome assessors aware of study participants' exposure history?	N	
6.3 **If Y/PY/NI to 6.2**: Could assessment of the outcome have been influenced by knowledge of participants' exposure history?	NA	
Risk of bias (arising from measurement of outcomes) in the estimated effect of exposure on the outcome	Low risk	
What is the predicted direction of bias arising from measurement of outcomes?	Towards null	
Is the risk of bias (arising from measurement of outcomes) sufficiently high, in the context of its likely direction and the magnitude of the estimated exposure effect, to threaten conclusions about whether the exposure has an important effect on the outcome?	No	

Y = Yes; PY = Probably yes; PN = Probably no; N = No; SY = Strong yes; WY = Weak yes; NA = Not applicable; NI = No information

Domain 7: Risk of bias in selection of the reported result

Signalling questions	Response options	Comments
7.1 Was the result reported in accordance with an available, pre-determined analysis plan?	Y	
7.2 **If N/PN/NI to 7.1**: Is the reported effect estimate likely to be selected, based on desirability of the magnitude (or statistical significance) of the estimated effect of exposure on outcome, from multiple *exposure measurements* within the exposure domain?	N	
7.3 Is the reported effect estimate likely to be selected, based on desirability of the magnitude (or statistical significance) of the estimated effect of exposure on outcome, from multiple *outcome measurements* within the outcome domain?	PN	
7.4 Is the reported effect estimate likely to be selected, based on desirability of the magnitude (or statistical significance) of the estimated effect of exposure on outcome, from multiple *analyses* of the exposure-outcome relationship?	N	
7.5 Is the reported effect estimate likely to be selected, based on the basis of desirability of the results (e.g. statistical significance), from different *subgroups*?	N	
Risk of bias (due to selection of the reported result) in the estimated effect of exposure on the outcome	Low risk	
What is the predicted direction of bias due to selection of the reported result?	Towards null	
Is the risk of bias (due to selection of the reported result) sufficiently high, in the context of its likely direction and the magnitude of the estimated exposure effect, to threaten conclusions about whether the exposure has an important effect on the outcome?	No	

Y = Yes; PY = Probably yes; PN = Probably no; N = No; NA = Not applicable; NI = No information

Overall risk of bias

	Response options	Comments
Overall risk of bias	Low risk of bias	
What is the predicted direction of bias?	Towards null	
Is the overall risk of bias sufficiently high, in the context of its likely direction and the magnitude of the estimated exposure effect, to threaten conclusions about whether the exposure has an important effect on the outcome?	No	

[27] Gilad, O.; Merlob, P.; Stahl, B.; Klinger, G. Outcome of infants exposed to olanzapine during breastfeeding. *Breastfeed. Med.* **2011**, *6*(2), 55-58. doi: 10.1089/bfm.2010.0027. Epub 2010 Oct 29.

Domain 1: Risk of bias due to confounding*, variant (b): **If Y/PY to C7 and Y/PY to C8** (the analysis was based on splitting participants' follow up time according to exposure status and/or magnitude and changes in exposure status and/or magnitude likely to be related to factors that are predictive of the outcome, so both baseline and time-varying confounding need to be addressed)*

Signalling questions	Response options	Comments
1.1 Did the authors use an analysis method that was appropriate to control for time-varying as well as baseline confounding?	Y	
1.2 **If Y/PY to 1.1**: Did the authors control for all the important baseline and time-varying confounding factors for which this was necessary?	Y	
1.3 **If Y/PY/WN to 1.2**: Were confounding factors that were controlled for (and for which control was necessary) measured validly and reliably by the variables available in this study?	Y	
1.4 **If N/PN/NI to 1.1**: Did the authors control for time-varying factors or other variables measured after the start of the exposure window being studied?	NA	
1.5 Did the use of negative controls, or other considerations, suggest uncontrolled confounding?	N	
Risk of bias (due to confounding) in the estimated effect of exposure on the outcome	Low risk	
What is the predicted direction of bias due to confounding?	Towards null	
Is the risk of bias (due to confounding) sufficiently high, in the context of its likely direction and the magnitude of the estimated exposure effect, to threaten conclusions about whether the exposure has an important effect on the outcome?	No	

Y = Yes; PY = Probably yes; PN = Probably no; N = No; SY = Strong yes; WY = Weak yes; SN = Strong no; WN = Weak no; NA = Not applicable; NI = No information

Domain 2: Risk of bias arising from measurement of the exposure *Variant (b): **If Y/PY to C5 and Y/PY to C6** (each individual's exposure level was estimated from measurements made at multiple time points)*

Signalling questions	Response options	Comments
**2.1 Does the measured exposure (derived from measurements at multiple time points) well-characterize the exposure metric specified to be of interest in this study? [*This was specified in the answers to D2, D3 and D4*]**	**Y**	
**2.2 Was there error in measurement, or misclassification, of the exposure, at each single time point?**	**N**	
**2.3 If SY/WY to 2.2: Could mismeasurement or misclassification of exposure have been differential (i.e. related to the outcome or risk of the outcome)?**	**NA**	
**2.4 If SY/WY to 2.2 and N/PN/WY to 2.3: Is the nature of the (non-differential) measurement error likely to bias the estimated effect of exposure on outcome?**	**NA**	
**Risk of bias (arising from measurement of exposure) in the estimated effect of exposure on the**	**Low risk**	

Signalling questions	Response options	Comments
**outcome**		
**What is the predicted direction of bias arising from measurement of exposure?**	**Towards null**	
**Is the risk of bias (arising from measurement of exposure) sufficiently high, in the context of its likely direction and the magnitude of the estimated exposure effect, to threaten conclusions about whether the exposure has an important effect on the outcome?**	**No**	

Y = Yes; PY = Probably yes; SN = Strong no; WN = Weak no; NA = Not applicable; NI = No information

Domain 3: Risk of bias in selection of participants into the study (or into the analysis)

Signalling questions	Response options	Comments
3.1 Did follow-up begin at (or close to) the start of the exposure window for most participants? [*The exposure window is specified in D3*]	Y	
3.2 **If N/PN to 3.1**: Is the effect of exposure likely to be constant over the period of follow up analysed?	NA	
3.3 Was selection of participants into the study (or into the analysis) based on participant characteristics observed after the start of the exposure window being studied? [*The exposure window is specified in D3*]	N	
3.4 **If Y/PY to 3.3**: Were these characteristics likely to be influenced by exposure or a cause of exposure?	NA	
3.5 **If Y/PY to 3.4**: Were these characteristics likely to be influenced by the outcome or a cause of the outcome?	NA	
3.6 **If N/PN to 3.2 or Y/PY to 3.5**: Is it likely that the analysis corrected for all of the potential selection biases identified in A and B above?	NA	
3.7 **If N/PN to 3.2 or Y/PY to 3.5**: Did sensitivity analyses demonstrate that the likely impact of the potential selection biases identified in A or B above was minimal?	NA	
Risk of bias (due to selection of participants into the study) in the estimated effect of exposure on the outcome	Low risk	
What is the predicted direction of bias due to selection of participants into the study?	Towards null	
Is the risk of bias (due to selection of participants into the study) sufficiently high, in the context of its likely direction and the magnitude of the estimated exposure effect, to threaten conclusions about whether the exposure has an important effect on the outcome?	No	

Y = Yes; PY = Probably yes; PN = Probably no; N = No; SN = Strong no; WN = Weak no; NA = Not applicable; NI = No information

Domain 4: Risk of bias due to post-exposure interventions

Signalling questions	Response options	Comments
4.1 Were there post-exposure interventions that were influenced by prior exposure during the follow-up period?	N	
4.2 **If Y/PY to 4.1**: Is it likely that the analysis corrected for the effect of post-exposure interventions that were influenced by prior exposure?	NA	
Risk of bias (due post-exposure interventions) in the estimated effect of exposure on the outcome	Low risk	
What is the predicted direction of bias due to confounding?	Towards null	
Is the risk of bias (due post-exposure interventions) sufficiently high, in the context of its likely direction and the magnitude of the estimated exposure effect, to threaten conclusions about whether the exposure has an important effect on the outcome?	No	

Y = Yes; PY = Probably yes; PN = Probably no; N = No; NA = Not applicable; NI = No information

Domain 5: Risk of bias due to missing data

Signalling questions	Response options	Comments
5.1 Were complete data on exposure status available for all, or nearly all, participants?	Y	
5.2 Were complete data on the outcome available for all, or nearly all, participants?	Y	
5.3 Were complete data on confounding variables available for all, or nearly all, participants?	Y	
5.4 **If N/PN/NI to 5.1, 5.2 or 5.3**: Is the result based on a complete case analysis?	NA	
5.5 **If Y/PY/NI**: Was exclusion from the analysis because of missing data (in exposure, confounders or the outcome) likely to be related to the true value of the outcome?	N	
5.6 **If N/PN to 5.5**: Were all or most predictors of missingness (in exposure, confounders or the outcome) included in the analysis model?	SY	
5.7 **If N/PN to 5.4**: Was the analysis based on imputing missing values?	NA	
5.8 **If Y/PY to 5.7**: Was imputation performed appropriately?	NA	
5.9 **If N/PN to 5.7**: Was an appropriate alternative method used to correct for bias due to missing data?	Y	
5.10 **If PN/N/NI to 5.1, 5.2 or 5.3**: Is there evidence that the result was not biased by missing data?	NA	
Risk of bias (due to missing data) in the estimated effect of exposure on the outcome	Low risk	
What is the predicted direction of bias due to missing data?	Towards null	
Is the risk of bias (due to missing data) sufficiently high, in the context of its likely direction and the magnitude of the estimated exposure effect, to threaten conclusions about whether the exposure has an important effect on the outcome?	No	

Y = Yes; PY = Probably yes; PN = Probably no; N = No; SY = Strong yes; WY = Weak yes; NA = Not applicable; NI = No information

Domain 6: Risk of bias arising from measurement of the outcome

Signalling questions	Response options	Comments
6.1 Could measurement or ascertainment of the outcome have differed between exposure groups or levels of exposure?	N	
6.2 Were outcome assessors aware of study participants' exposure history?	PN	
6.3 **If Y/PY/NI to 6.2**: Could assessment of the outcome have been influenced by knowledge of participants' exposure history?	NA	
Risk of bias (arising from measurement of outcomes) in the estimated effect of exposure on the outcome	Low risk	
What is the predicted direction of bias arising from measurement of outcomes?	Towards null	
Is the risk of bias (arising from measurement of outcomes) sufficiently high, in the context of its likely direction and the magnitude of the estimated exposure effect, to threaten conclusions about whether the exposure has an important effect on the outcome?	No	

Y = Yes; PY = Probably yes; PN = Probably no; N = No; SY = Strong yes; WY = Weak yes; NA = Not applicable; NI = No information

Domain 7: Risk of bias in selection of the reported result

Signalling questions	Response options	Comments
7.1 Was the result reported in accordance with an available, pre-determined analysis plan?	Y	
7.2 **If N/PN/NI to 7.1**: Is the reported effect estimate likely to be selected, based on desirability of the magnitude (or statistical significance) of the estimated effect of exposure on outcome, from multiple *exposure measurements* within the exposure domain?	N	
7.3 Is the reported effect estimate likely to be selected, based on desirability of the magnitude (or statistical significance) of the estimated effect of exposure on outcome, from multiple *outcome measurements* within the outcome domain?	N	
7.4 Is the reported effect estimate likely to be selected, based on desirability of the magnitude (or statistical significance) of the estimated effect of exposure on outcome, from multiple *analyses* of the exposure-outcome relationship?	N	
7.5 Is the reported effect estimate likely to be selected, based on the basis of desirability of the results (e.g. statistical significance), from different *subgroups*?	N	
Risk of bias (due to selection of the reported result) in the estimated effect of exposure on the outcome	Low risk	
What is the predicted direction of bias due to selection of the reported result?	Towards null	
Is the risk of bias (due to selection of the reported result) sufficiently high, in the context of its likely direction and the magnitude of the estimated exposure effect, to threaten conclusions about whether the exposure has an important effect on the outcome?	No	

Y = Yes; PY = Probably yes; PN = Probably no; N = No; NA = Not applicable; NI = No information

Overall risk of bias

	Response options	Comments
Overall risk of bias	Low risk of bias	
What is the predicted direction of bias?	Towards null	
Is the overall risk of bias sufficiently high, in the context of its likely direction and the magnitude of the estimated exposure effect, to threaten conclusions about whether the exposure has an important effect on the outcome?	No	

[28] Habermann, F.; Fritzsche, J.; Fuhlbrück, F.; Wacker, E.; Allignol, A.; Weber-Schoendorfer, C.; Meister, R.; Schaefer, C. Atypical antipsychotic drugs and pregnancy outcome: a prospective, cohort study. *J. Clin. Psychopharmacol.* **2013**, *33*(4), 453-462. doi: 10.1097/JCP.0b013e318295fe12.

Domain 1: Risk of bias due to confounding*, variant (b): **If Y/PY to C7 and Y/PY to C8** (the analysis was based on splitting participants' follow up time according to exposure status and/or magnitude and changes in exposure status and/or magnitude likely to be related to factors that are predictive of the outcome, so both baseline and time-varying confounding need to be addressed)*

Signalling questions	Response options	Comments
1.1 Did the authors use an analysis method that was appropriate to control for time-varying as well as baseline confounding?	Y	
1.2 **If Y/PY to 1.1**: Did the authors control for all the important baseline and time-varying confounding factors for which this was necessary?	Y	
1.3 **If Y/PY/WN to 1.2**: Were confounding factors that were controlled for (and for which control was necessary) measured validly and reliably by the variables available in this study?	Y	
1.4 **If N/PN/NI to 1.1**: Did the authors control for time-varying factors or other variables measured after the start of the exposure window being studied?	NA	
1.5 Did the use of negative controls, or other considerations, suggest uncontrolled confounding?	N	
Risk of bias (due to confounding) in the estimated effect of exposure on the outcome	Low risk	
What is the predicted direction of bias due to confounding?	Towards null	
Is the risk of bias (due to confounding) sufficiently high, in the context of its likely direction and the magnitude of the estimated exposure effect, to threaten conclusions about whether the exposure has an important effect on the outcome?	No	

Y = Yes; PY = Probably yes; PN = Probably no; N = No; SY = Strong yes; WY = Weak yes; SN = Strong no; WN = Weak no; NA = Not applicable; NI = No information

Domain 2: Risk of bias arising from measurement of the exposure *Variant (b): **If Y/PY to C5 and Y/PY to C6** (each individual's exposure level was estimated from measurements made at multiple time points)*

Signalling questions	Response options	Comments
**2.1 Does the measured exposure (derived from measurements at multiple time points) well-characterize the exposure metric specified to be of interest in this study? [*This was specified in the answers to D2, D3 and D4*]**	**Y**	
**2.2 Was there error in measurement, or misclassification, of the exposure, at each single time point?**	**PN**	
**2.3 If SY/WY to 2.2: Could mismeasurement or misclassification of exposure have been differential (i.e. related to the outcome or risk of the outcome)?**	**NA**	
**2.4 If SY/WY to 2.2 and N/PN/WY to 2.3: Is the nature of the (non-differential) measurement error likely to bias the estimated effect of exposure on outcome?**	**NA**	
**Risk of bias (arising from measurement of exposure) in the estimated effect of exposure on the outcome**	**Low risk**	
**What is the predicted direction of bias arising from measurement of exposure?**	**Towards null**	
**Is the risk of bias (arising from measurement of exposure) sufficiently high, in the context of its likely direction and the magnitude of the estimated exposure effect, to threaten conclusions about whether the exposure has an important effect on the outcome?**	**No**	

Y = Yes; PY = Probably yes; SN = Strong no; WN = Weak no; NA = Not applicable; NI = No information

Domain 3: Risk of bias in selection of participants into the study (or into the analysis)

Signalling questions	Response options	Comments
3.1 Did follow-up begin at (or close to) the start of the exposure window for most participants? [*The exposure window is specified in D3*]	Y	
3.2 **If N/PN to 3.1**: Is the effect of exposure likely to be constant over the period of follow up analysed?	NA	
3.3 Was selection of participants into the study (or into the analysis) based on participant characteristics observed after the start of the exposure window being studied? [*The exposure window is specified in D3*]	N	
3.4 **If Y/PY to 3.3**: Were these characteristics likely to be influenced by exposure or a cause of exposure?	NA	
3.5 **If Y/PY to 3.4**: Were these characteristics likely to be influenced by the outcome or a cause of the outcome?	NA	
3.6 **If N/PN to 3.2 or Y/PY to 3.5**: Is it likely that the analysis corrected for all of the potential selection biases identified in A and B above?	NA	
3.7 **If N/PN to 3.2 or Y/PY to 3.5**: Did sensitivity analyses demonstrate that the likely impact of the potential selection biases identified in A or B above was minimal?	NA	
Risk of bias (due to selection of participants into the study) in the estimated effect of exposure on the outcome	Low risk	
What is the predicted direction of bias due to selection of participants into the study?	Towards null	
Is the risk of bias (due to selection of participants into the study) sufficiently high, in the context of its likely direction and the magnitude of the estimated exposure effect, to threaten conclusions about whether the exposure has an important effect on the outcome?	No	

Y = Yes; PY = Probably yes; PN = Probably no; N = No; SN = Strong no; WN = Weak no; NA = Not applicable; NI = No information

Domain 4: Risk of bias due to post-exposure interventions

Signalling questions	Response options	Comments
4.1 Were there post-exposure interventions that were influenced by prior exposure during the follow-up period?	N	
4.2 **If Y/PY to 4.1**: Is it likely that the analysis corrected for the effect of post-exposure interventions that were influenced by prior exposure?	NA	
Risk of bias (due post-exposure interventions) in the estimated effect of exposure on the outcome	Low risk	
What is the predicted direction of bias due to confounding?	Towards null	
Is the risk of bias (due post-exposure interventions) sufficiently high, in the context of its likely direction and the magnitude of the estimated exposure effect, to threaten conclusions about whether the exposure has an important effect on the outcome?	No	

Y = Yes; PY = Probably yes; PN = Probably no; N = No; NA = Not applicable; NI = No information

Domain 5: Risk of bias due to missing data

Signalling questions	Response options	Comments
5.1 Were complete data on exposure status available for all, or nearly all, participants?	Y	
5.2 Were complete data on the outcome available for all, or nearly all, participants?	Y	
5.3 Were complete data on confounding variables available for all, or nearly all, participants?	Y	
5.4 **If N/PN/NI to 5.1, 5.2 or 5.3**: Is the result based on a complete case analysis?	NA	
5.5 **If Y/PY/NI**: Was exclusion from the analysis because of missing data (in exposure, confounders or the outcome) likely to be related to the true value of the outcome?	N	
5.6 **If N/PN to 5.5**: Were all or most predictors of missingness (in exposure, confounders or the outcome) included in the analysis model?	SY	
5.7 **If N/PN to 5.4**: Was the analysis based on imputing missing values?	NA	
5.8 **If Y/PY to 5.7**: Was imputation performed appropriately?	NA	
5.9 **If N/PN to 5.7**: Was an appropriate alternative method used to correct for bias due to missing data?	Y	
5.10 **If PN/N/NI to 5.1, 5.2 or 5.3**: Is there evidence that the result was not biased by missing data?	NA	
Risk of bias (due to missing data) in the estimated effect of exposure on the outcome	Low risk	

Signalling questions	Response options	Comments
What is the predicted direction of bias due to missing data?	Towards null	
Is the risk of bias (due to missing data) sufficiently high, in the context of its likely direction and the magnitude of the estimated exposure effect, to threaten conclusions about whether the exposure has an important effect on the outcome?	No	

Y = Yes; PY = Probably yes; PN = Probably no; N = No; SY = Strong yes; WY = Weak yes; NA = Not applicable; NI = No information

Domain 6: Risk of bias arising from measurement of the outcome

Signalling questions	Response options	Comments
6.1 Could measurement or ascertainment of the outcome have differed between exposure groups or levels of exposure?	N	
6.2 Were outcome assessors aware of study participants' exposure history?	N	
6.3 **If Y/PY/NI to 6.2**: Could assessment of the outcome have been influenced by knowledge of participants' exposure history?	NA	
Risk of bias (arising from measurement of outcomes) in the estimated effect of exposure on the outcome	Low risk	
What is the predicted direction of bias arising from measurement of outcomes?	Towards null	
Is the risk of bias (arising from measurement of outcomes) sufficiently high, in the context of its likely direction and the magnitude of the estimated exposure effect, to threaten conclusions about whether the exposure has an important effect on the outcome?	No	

Y = Yes; PY = Probably yes; PN = Probably no; N = No; SY = Strong yes; WY = Weak yes; NA = Not applicable; NI = No information

Domain 7: Risk of bias in selection of the reported result

Signalling questions	Response options	Comments
7.1 Was the result reported in accordance with an available, pre-determined analysis plan?	Y	
7.2 **If N/PN/NI to 7.1**: Is the reported effect estimate likely to be selected, based on desirability of the magnitude (or statistical significance) of the estimated effect of exposure on outcome, from multiple *exposure measurements* within the exposure domain?	N	
7.3 Is the reported effect estimate likely to be selected, based on desirability of the magnitude (or statistical significance) of the estimated effect of exposure on outcome, from multiple *outcome measurements* within the outcome domain?	N	
7.4 Is the reported effect estimate likely to be selected, based on desirability of the magnitude (or statistical significance) of the estimated effect of exposure on outcome, from multiple *analyses* of the exposure-outcome relationship?	N	
7.5 Is the reported effect estimate likely to be selected, based on the basis of desirability of the results (e.g. statistical significance), from different *subgroups*?	N	
Risk of bias (due to selection of the reported result) in the estimated effect of exposure on the outcome	Low risk	
What is the predicted direction of bias due to selection of the reported result?	Towards null	
Is the risk of bias (due to selection of the reported result) sufficiently high, in the context of its likely direction and the magnitude of the estimated exposure effect, to threaten conclusions about whether the exposure has an important effect on the outcome?	No	

Y = Yes; PY = Probably yes; PN = Probably no; N = No; NA = Not applicable; NI = No information

Overall risk of bias

	Response options	Comments
Overall risk of bias	Low risk of bias	
What is the predicted direction of bias?	Towards null	
Is the overall risk of bias sufficiently high, in the context of its likely direction and the magnitude of the estimated exposure effect, to threaten conclusions about whether the exposure has an important effect on the outcome?	No	

[29] Sadowski, A.; Todorow, M.; Yazdani, Brojeni, P.; Koren, G.; Nulman, I. Pregnancy outcomes following maternal exposure to second-generation antipsychotics given with other psychotropic drugs: a cohort study. *BMJ Open* **2013**, *3*(7), e003062. doi: 10.1136/bmjopen-2013-003062.

Domain 1: Risk of bias due to confounding*, variant (b):* ***If Y/PY to C7 and Y/PY to C8*** *(the analysis was based on splitting participants' follow up time according to exposure status and/or magnitude and changes in exposure status and/or magnitude likely to be related to factors that are predictive of the outcome, so both baseline and time-varying confounding need to be addressed)*

Signalling questions	Response options	Comments
1.1 Did the authors use an analysis method that was appropriate to control for time-varying as well as baseline confounding?	Y	
1.2 **If Y/PY to 1.1**: Did the authors control for all the important baseline and time-varying confounding factors for which this was necessary?	PY	
1.3 **If Y/PY/WN to 1.2**: Were confounding factors that were controlled for (and for which control was necessary) measured validly and reliably by the variables available in this study?	Y	
1.4 **If N/PN/NI to 1.1**: Did the authors control for time-varying factors or other variables measured after the start of the exposure window being studied?	NA	
1.5 Did the use of negative controls, or other considerations, suggest uncontrolled confounding?	N	

Signalling questions	Response options	Comments
Risk of bias (due to confounding) in the estimated effect of exposure on the outcome	Low risk	
What is the predicted direction of bias due to confounding?	Towards null	
Is the risk of bias (due to confounding) sufficiently high, in the context of its likely direction and the magnitude of the estimated exposure effect, to threaten conclusions about whether the exposure has an important effect on the outcome?	No	

Y = Yes; PY = Probably yes; PN = Probably no; N = No; SY = Strong yes; WY = Weak yes; SN = Strong no; WN = Weak no; NA = Not applicable; NI = No information

Domain 2: Risk of bias arising from measurement of the exposure *Variant (b): If Y/PY to C5 and Y/PY to C6 (each individual's exposure level was estimated from measurements made at multiple time points)*

Signalling questions	Response options	Comments
**2.1 Does the measured exposure (derived from measurements at multiple time points) well-characterize the exposure metric specified to be of interest in this study? [*This was specified in the answers to D2, D3 and D4*]**	**PY**	
**2.2 Was there error in measurement, or misclassification, of the exposure, at each single time point?**	**N**	
**2.3 If SY/WY to 2.2: Could mismeasurement or misclassification of exposure have been differential (i.e. related to the outcome or risk of the outcome)?**	**NA**	
**2.4 If SY/WY to 2.2 and N/PN/WY to 2.3: Is the nature of the (non-differential) measurement error likely to bias the estimated effect of exposure on outcome?**	**NA**	
**Risk of bias (arising from measurement of exposure) in the estimated effect of exposure on the outcome**	**Low risk**	
**What is the predicted direction of bias arising from measurement of exposure?**	**Towards null**	
**Is the risk of bias (arising from measurement of exposure) sufficiently high, in the context of its likely direction and the magnitude of the estimated exposure effect, to threaten conclusions about whether the exposure has an important effect on the outcome?**	**No**	

Y = Yes; PY = Probably yes; SN = Strong no; WN = Weak no; NA = Not applicable; NI = No information

Domain 3: Risk of bias in selection of participants into the study (or into the analysis)

Signalling questions	Response options	Comments
3.1 Did follow-up begin at (or close to) the start of the exposure window for most participants? [*The exposure window is specified in D3*]	Y	
3.2 **If N/PN to 3.1**: Is the effect of exposure likely to be constant over the period of follow up analysed?	NA	
3.3 Was selection of participants into the study (or into the analysis) based on participant characteristics observed after the start of the exposure window being studied? [*The exposure window is specified in D3*]	N	
3.4 **If Y/PY to 3.3**: Were these characteristics likely to be influenced by exposure or a cause of exposure?	NA	
3.5 **If Y/PY to 3.4**: Were these characteristics likely to be influenced by the outcome or a cause of the outcome?	NA	
3.6 **If N/PN to 3.2 or Y/PY to 3.5**: Is it likely that the analysis corrected for all of the potential selection biases identified in A and B above?	NA	
3.7 **If N/PN to 3.2 or Y/PY to 3.5**: Did sensitivity analyses demonstrate that the likely impact of the potential selection biases identified in A or B above was minimal?	NA	
Risk of bias (due to selection of participants into the study) in the estimated effect of exposure on the outcome	Low risk	
What is the predicted direction of bias due to selection of participants into the study?	Towards null	
Is the risk of bias (due to selection of participants into the study) sufficiently high, in the context of its likely direction and the magnitude of the estimated exposure effect, to threaten conclusions about whether the exposure has an important effect on the outcome?	No	

Y = Yes; PY = Probably yes; PN = Probably no; N = No; SN = Strong no; WN = Weak no; NA = Not applicable; NI = No information

Domain 4: Risk of bias due to post-exposure interventions

Signalling questions	Response options	Comments
4.1 Were there post-exposure interventions that were influenced by prior exposure during the follow-up period?	N	
4.2 **If Y/PY to 4.1**: Is it likely that the analysis corrected for the effect of post-exposure interventions that were influenced by prior exposure?	NA	
Risk of bias (due post-exposure interventions) in the estimated effect of exposure on the outcome	Low risk	
What is the predicted direction of bias due to confounding?	Towards null	
Is the risk of bias (due post-exposure interventions) sufficiently high, in the context of its likely direction and the magnitude of the estimated exposure effect, to threaten conclusions about whether the exposure has an important effect on the outcome?	No	

Y = Yes; PY = Probably yes; PN = Probably no; N = No; NA = Not applicable; NI = No information

Domain 5: Risk of bias due to missing data

Signalling questions	Response options	Comments
5.1 Were complete data on exposure status available for all, or nearly all, participants?	Y	

Signalling questions	Response options	Comments
5.2 Were complete data on the outcome available for all, or nearly all, participants?	Y	
5.3 Were complete data on confounding variables available for all, or nearly all, participants?	Y	
5.4 **If N/PN/NI to 5.1, 5.2 or 5.3**: Is the result based on a complete case analysis?	NA	
5.5 **If Y/PY/NI**: Was exclusion from the analysis because of missing data (in exposure, confounders or the outcome) likely to be related to the true value of the outcome?	N	
5.6 **If N/PN to 5.5**: Were all or most predictors of missingness (in exposure, confounders or the outcome) included in the analysis model?	SY	
5.7 **If N/PN to 5.4**: Was the analysis based on imputing missing values?	NA	
5.8 **If Y/PY to 5.7**: Was imputation performed appropriately?	NA	
5.9 **If N/PN to 5.7**: Was an appropriate alternative method used to correct for bias due to missing data?	Y	
5.10 **If PN/N/NI to 5.1, 5.2 or 5.3**: Is there evidence that the result was not biased by missing data?	NA	
Risk of bias (due to missing data) in the estimated effect of exposure on the outcome	Low risk	
What is the predicted direction of bias due to missing data?	Towards null	
Is the risk of bias (due to missing data) sufficiently high, in the context of its likely direction and the magnitude of the estimated exposure effect, to threaten conclusions about whether the exposure has an important effect on the outcome?	No	

Y = Yes; PY = Probably yes; PN = Probably no; N = No; SY = Strong yes; WY = Weak yes; NA = Not applicable; NI = No information

Domain 6: Risk of bias arising from measurement of the outcome

Signalling questions	Response options	Comments
6.1 Could measurement or ascertainment of the outcome have differed between exposure groups or levels of exposure?	N	
6.2 Were outcome assessors aware of study participants' exposure history?	N	
6.3 **If Y/PY/NI to 6.2**: Could assessment of the outcome have been influenced by knowledge of participants' exposure history?	NA	
Risk of bias (arising from measurement of outcomes) in the estimated effect of exposure on the outcome	Low risk	
What is the predicted direction of bias arising from measurement of outcomes?	Towards null	
Is the risk of bias (arising from measurement of outcomes) sufficiently high, in the context of its likely direction and the magnitude of the estimated exposure effect, to threaten conclusions about whether the exposure has an important effect on the outcome?	No	

Y = Yes; PY = Probably yes; PN = Probably no; N = No; SY = Strong yes; WY = Weak yes; NA = Not applicable; NI = No information

Domain 7: Risk of bias in selection of the reported result

Signalling questions	Response options	Comments
7.1 Was the result reported in accordance with an available, pre-determined analysis plan?	Y	
7.2 **If N/PN/NI to 7.1**: Is the reported effect estimate likely to be selected, based on desirability of the magnitude (or statistical significance) of the estimated effect of exposure on outcome, from multiple *exposure measurements* within the exposure domain?	N	
7.3 Is the reported effect estimate likely to be selected, based on desirability of the magnitude (or statistical significance) of the estimated effect of exposure on outcome, from multiple *outcome measurements* within the outcome domain?	PN	
7.4 Is the reported effect estimate likely to be selected, based on desirability of the magnitude (or statistical significance) of the estimated effect of exposure on outcome, from multiple *analyses* of the exposure-outcome relationship?	N	
7.5 Is the reported effect estimate likely to be selected, based on the basis of desirability of the results (e.g. statistical significance), from different *subgroups*?	N	
Risk of bias (due to selection of the reported result) in the estimated effect of exposure on the outcome	Low risk	
What is the predicted direction of bias due to selection of the reported result?	Towards null	
Is the risk of bias (due to selection of the reported result) sufficiently high, in the context of its likely direction and the magnitude of the estimated exposure effect, to threaten conclusions about whether the exposure has an important effect on the outcome?	No	

Y = Yes; PY = Probably yes; PN = Probably no; N = No; NA = Not applicable; NI = No information

Overall risk of bias

	Response options	Comments

Overall risk of bias	Low risk of bias	
What is the predicted direction of bias?	Towards null	
Is the overall risk of bias sufficiently high, in the context of its likely direction and the magnitude of the estimated exposure effect, to threaten conclusions about whether the exposure has an important effect on the outcome?	No	

[30] Bellet, F.; Beyens, M.N.; Bernard, N.; Beghin, D.; Elefant, E.; Vial, T. Exposure to aripiprazole during embryogenesis: a prospective multicenter cohort study. *Pharmacoepidemiol. Drug Saf.* **2015**, *24*(4), 368-380. doi: 10.1002/pds.3749. Epub 2015 Feb 12.

Domain 1: Risk of bias due to confounding, *variant (b): **If Y/PY to C7 and Y/PY to C8** (the analysis was based on splitting participants' follow up time according to exposure status and/or magnitude and changes in exposure status and/or magnitude likely to be related to factors that are predictive of the outcome, so both baseline and time-varying confounding need to be addressed)*

Signalling questions	Response options	Comments
1.1 Did the authors use an analysis method that was appropriate to control for time-varying as well as baseline confounding?	Y	
1.2 **If Y/PY to 1.1**: Did the authors control for all the important baseline and time-varying confounding factors for which this was necessary?	Y	
1.3 **If Y/PY/WN to 1.2**: Were confounding factors that were controlled for (and for which control was necessary) measured validly and reliably by the variables available in this study?	Y	
1.4 **If N/PN/NI to 1.1**: Did the authors control for time-varying factors or other variables measured after the start of the exposure window being studied?	NA	
1.5 Did the use of negative controls, or other considerations, suggest uncontrolled confounding?	N	
Risk of bias (due to confounding) in the estimated effect of exposure on the outcome	Low risk	
What is the predicted direction of bias due to confounding?	Towards null	
Is the risk of bias (due to confounding) sufficiently high, in the context of its likely direction and the magnitude of the estimated exposure effect, to threaten conclusions about whether the exposure has an important effect on the outcome?	No	

Y = Yes; PY = Probably yes; PN = Probably no; N = No; SY = Strong yes; WY = Weak yes; SN = Strong no; WN = Weak no; NA = Not applicable; NI = No information

Domain 2: Risk of bias arising from measurement of the exposure *Variant (b): **If Y/PY to C5 and Y/PY to C6** (each individual's exposure level was estimated from measurements made at multiple time points)*

Signalling questions	Response options	Comments
**2.1 Does the measured exposure (derived from measurements at multiple time points) well-characterize the exposure metric specified to be of interest in this study? [*This was specified in the answers to D2, D3 and D4*]**	**Y**	
**2.2 Was there error in measurement, or misclassification, of the exposure, at each single time point?**	**N**	
**2.3 If SY/WY to 2.2: Could mismeasurement or misclassification of exposure have been differential (i.e. related to the outcome or risk of the outcome)?**	**NA**	
**2.4 If SY/WY to 2.2 and N/PN/WY to 2.3: Is the nature of the (non-differential) measurement error likely to bias the estimated effect of exposure on outcome?**	**NA**	
**Risk of bias (arising from measurement of exposure) in the estimated effect of exposure on the outcome**	**Low risk**	
**What is the predicted direction of bias arising from measurement of exposure?**	**Towards null**	
**Is the risk of bias (arising from measurement of exposure) sufficiently high, in the context of its likely direction and the magnitude of the estimated exposure effect, to threaten conclusions about whether the exposure has an important effect on the outcome?**	**No**	

Y = Yes; PY = Probably yes; SN = Strong no; WN = Weak no; NA = Not applicable; NI = No information

Domain 3: Risk of bias in selection of participants into the study (or into the analysis)

Signalling questions	Response options	Comments
3.1 Did follow-up begin at (or close to) the start of the exposure window for most participants? [*The exposure window is specified in D3*]	Y	
3.2 **If N/PN to 3.1**: Is the effect of exposure likely to be constant over the period of follow up analysed?	NA	
3.3 Was selection of participants into the study (or into the analysis) based on participant characteristics observed after the start of the exposure window being studied? [*The exposure window is specified in D3*]	N	
3.4 **If Y/PY to 3.3**: Were these characteristics likely to be influenced by exposure or a cause of exposure?	NA	
3.5 **If Y/PY to 3.4**: Were these characteristics likely to be influenced by the outcome or a cause of the outcome?	NA	
3.6 **If N/PN to 3.2 or Y/PY to 3.5**: Is it likely that the analysis corrected for all of the potential selection biases identified in A and B above?	NA	
3.7 **If N/PN to 3.2 or Y/PY to 3.5**: Did sensitivity analyses demonstrate that the likely impact of the potential selection biases identified in A or B above was minimal?	NA	
Risk of bias (due to selection of participants into the study) in the estimated effect of exposure on the outcome	Low risk	
What is the predicted direction of bias due to selection of participants into the study?	Towards null	
Is the risk of bias (due to selection of participants into the study) sufficiently high, in the context of its likely direction and the magnitude of the estimated exposure effect, to threaten conclusions about whether the exposure has an important	No	

Signalling questions	Response options	Comments
effect on the outcome?		

Y = Yes; PY = Probably yes; PN = Probably no; N = No; SN = Strong no; WN = Weak no; NA = Not applicable; NI = No information

Domain 4: Risk of bias due to post-exposure interventions

Signalling questions	Response options	Comments
4.1 Were there post-exposure interventions that were influenced by prior exposure during the follow-up period?	N	
4.2 **If Y/PY to 4.1**: Is it likely that the analysis corrected for the effect of post-exposure interventions that were influenced by prior exposure?	NA	
Risk of bias (due post-exposure interventions) in the estimated effect of exposure on the outcome	Low risk	
What is the predicted direction of bias due to confounding?	Towards null	
Is the risk of bias (due post-exposure interventions) sufficiently high, in the context of its likely direction and the magnitude of the estimated exposure effect, to threaten conclusions about whether the exposure has an important effect on the outcome?	No	

Y = Yes; PY = Probably yes; PN = Probably no; N = No; NA = Not applicable; NI = No information

Domain 5: Risk of bias due to missing data

Signalling questions	Response options	Comments
5.1 Were complete data on exposure status available for all, or nearly all, participants?	Y	
5.2 Were complete data on the outcome available for all, or nearly all, participants?	Y	
5.3 Were complete data on confounding variables available for all, or nearly all, participants?	Y	
5.4 **If N/PN/NI to 5.1, 5.2 or 5.3**: Is the result based on a complete case analysis?	NA	
5.5 **If Y/PY/NI**: Was exclusion from the analysis because of missing data (in exposure, confounders or the outcome) likely to be related to the true value of the outcome?	N	
5.6 **If N/PN to 5.5**: Were all or most predictors of missingness (in exposure, confounders or the outcome) included in the analysis model?	SY	
5.7 **If N/PN to 5.4**: Was the analysis based on imputing missing values?	NA	
5.8 **If Y/PY to 5.7**: Was imputation performed appropriately?	NA	
5.9 **If N/PN to 5.7**: Was an appropriate alternative method used to correct for bias due to missing data?	Y	
5.10 **If PN/N/NI to 5.1, 5.2 or 5.3**: Is there evidence that the result was not biased by missing data?	NA	
Risk of bias (due to missing data) in the estimated effect of exposure on the outcome	Low risk	
What is the predicted direction of bias due to missing data?	Towards null	
Is the risk of bias (due to missing data) sufficiently high, in the context of its likely direction and the magnitude of the estimated exposure effect, to threaten conclusions about whether the exposure has an important effect on the outcome?	No	

Y = Yes; PY = Probably yes; PN = Probably no; N = No; SY = Strong yes; WY = Weak yes; NA = Not applicable; NI = No information

Domain 6: Risk of bias arising from measurement of the outcome

Signalling questions	Response options	Comments
6.1 Could measurement or ascertainment of the outcome have differed between exposure groups or levels of exposure?	N	
6.2 Were outcome assessors aware of study participants' exposure history?	PN	
6.3 **If Y/PY/NI to 6.2**: Could assessment of the outcome have been influenced by knowledge of participants' exposure history?	NA	
Risk of bias (arising from measurement of outcomes) in the estimated effect of exposure on the outcome	Low risk	
What is the predicted direction of bias arising from measurement of outcomes?	Towards null	
Is the risk of bias (arising from measurement of outcomes) sufficiently high, in the context of its likely direction and the magnitude of the estimated exposure effect, to threaten conclusions about whether the exposure has an important effect on the outcome?	No	

Y = Yes; PY = Probably yes; PN = Probably no; N = No; SY = Strong yes; WY = Weak yes; NA = Not applicable; NI = No information

Domain 7: Risk of bias in selection of the reported result

Signalling questions	Response options	Comments
7.1 Was the result reported in accordance with an available, pre-determined analysis plan?	Y	
7.2 **If N/PN/NI to 7.1**: Is the reported effect estimate likely to be selected, based on desirability of the magnitude (or statistical significance) of the estimated effect of exposure on outcome, from multiple *exposure measurements* within the exposure domain?	N	
7.3 Is the reported effect estimate likely to be selected, based on desirability of the magnitude (or statistical significance) of the estimated effect of exposure on outcome, from multiple *outcome measurements* within the outcome domain?	N	
7.4 Is the reported effect estimate likely to be selected, based on desirability of the magnitude (or statistical significance) of the estimated effect of exposure on outcome, from multiple *analyses* of the exposure-outcome relationship?	N	
7.5 Is the reported effect estimate likely to be selected, based on the basis of desirability of the results (e.g. statistical significance), from different *subgroups*?	N	
Risk of bias (due to selection of the reported result) in the estimated effect of exposure on the outcome	Low risk	
What is the predicted direction of bias due to selection of the reported result?	Towards null	
Is the risk of bias (due to selection of the reported result) sufficiently high, in the context of its likely direction and the	No	

Signalling questions	Response options	Comments
magnitude of the estimated exposure effect, to threaten conclusions about whether the exposure has an important effect on the outcome?		

Y = Yes; PY = Probably yes; PN = Probably no; N = No; NA = Not applicable; NI = No information

Overall risk of bias

	Response options	Comments
Overall risk of bias	Low risk of bias	
What is the predicted direction of bias?	Towards null	
Is the overall risk of bias sufficiently high, in the context of its likely direction and the magnitude of the estimated exposure effect, to threaten conclusions about whether the exposure has an important effect on the outcome?	No	

[31] Cohen, L.S.; Viguera, A.C.; McInerney, K.A.; Freeman, M.P.; Sosinsky, A.Z.; Moustafa, D.; Marfurt, S.P.; Kwiatkowski, M.A.; Murphy, S.K.; Farrell, A.M.; Chitayat, D.; Hernández-Díaz, S. Reproductive safety of second-generation antipsychotics: Current data from the Massachusetts General Hospital National Pregnancy Registry for Atypical Antipsychotics. *Am. J. Psychiatry* **2016**, *173*(3), 263-270. doi: 10.1176/appi.ajp.2015.15040506. Epub 2015 Oct 6.

Domain 1: Risk of bias due to confounding, *variant (b):* ***If Y/PY to C7 and Y/PY to C8*** *(the analysis was based on splitting participants' follow up time according to exposure status and/or magnitude and changes in exposure status and/or magnitude likely to be related to factors that are predictive of the outcome, so both baseline and time-varying confounding need to be addressed)*

Signalling questions	Response options	Comments
1.1 Did the authors use an analysis method that was appropriate to control for time-varying as well as baseline confounding?	Y	
1.2 **If Y/PY to 1.1**: Did the authors control for all the important baseline and time-varying confounding factors for which this was necessary?	PY	
1.3 **If Y/PY/WN to 1.2**: Were confounding factors that were controlled for (and for which control was necessary) measured validly and reliably by the variables available in this study?	Y	
1.4 **If N/PN/NI to 1.1**: Did the authors control for time-varying factors or other variables measured after the start of the exposure window being studied?	NA	
1.5 Did the use of negative controls, or other considerations, suggest uncontrolled confounding?	N	
Risk of bias (due to confounding) in the estimated effect of exposure on the outcome	Low risk	
What is the predicted direction of bias due to confounding?	Towards null	
Is the risk of bias (due to confounding) sufficiently high, in the context of its likely direction and the magnitude of the estimated exposure effect, to threaten conclusions about whether the exposure has an important effect on the outcome?	No	

Y = Yes; PY = Probably yes; PN = Probably no; N = No; SY = Strong yes; WY = Weak yes; SN = Strong no; WN = Weak no; NA = Not applicable; NI = No information

Domain 2: Risk of bias arising from measurement of the exposure *Variant (b):* ***If Y/PY to C5 and Y/PY to C6*** *(each individual's exposure level was estimated from measurements made at multiple time points)*

Signalling questions	Response options	Comments
**2.1 Does the measured exposure (derived from measurements at multiple time points) well-characterize the exposure metric specified to be of interest in this study? [*This was specified in the answers to D2, D3 and D4*]**	**Y**	
**2.2 Was there error in measurement, or misclassification, of the exposure, at each single time point?**	**N**	
**2.3 If SY/WY to 2.2: Could mismeasurement or misclassification of exposure have been differential (i.e. related to the outcome or risk of the outcome)?**	**NA**	
**2.4 If SY/WY to 2.2 and N/PN/WY to 2.3: Is the nature of the (non-differential) measurement error likely to bias the estimated effect of exposure on outcome?**	**NA**	
**Risk of bias (arising from measurement of exposure) in the estimated effect of exposure on the outcome**	**Low risk**	
**What is the predicted direction of bias arising from measurement of exposure?**	**Towards null**	
**Is the risk of bias (arising from measurement of exposure) sufficiently high, in the context of its likely direction and the magnitude of the estimated exposure effect, to threaten conclusions about whether the exposure has an important effect on the outcome?**	**No**	

Y = Yes; PY = Probably yes; SN = Strong no; WN = Weak no; NA = Not applicable; NI = No information

Domain 3: Risk of bias in selection of participants into the study (or into the analysis)

Signalling questions	Response options	Comments
3.1 Did follow-up begin at (or close to) the start of the exposure window for most participants? [*The exposure window is specified in D3*]	Y	
3.2 **If N/PN to 3.1**: Is the effect of exposure likely to be constant over the period of follow up analysed?	NA	
3.3 Was selection of participants into the study (or into the analysis) based on participant characteristics observed after the start of the exposure window being studied? [*The exposure window is specified in D3*]	N	
3.4 **If Y/PY to 3.3**: Were these characteristics likely to be influenced by exposure or a cause of exposure?	NA	
3.5 **If Y/PY to 3.4**: Were these characteristics likely to be influenced by the outcome or a cause of the outcome?	NA	
3.6 **If N/PN to 3.2 or Y/PY to 3.5**: Is it likely that the analysis corrected for all of the potential selection biases identified in A and B above?	NA	
3.7 **If N/PN to 3.2 or Y/PY to 3.5**: Did sensitivity analyses demonstrate that the likely impact of the potential selection biases identified in A or B above was minimal?	NA	
Risk of bias (due to selection of participants into the study) in the estimated effect of exposure on the outcome	Low risk	
What is the predicted direction of bias due to selection of participants into the study?	Towards null	
Is the risk of bias (due to selection of participants into the study) sufficiently high, in the context of its likely direction and the magnitude of the estimated exposure effect, to threaten conclusions about whether the exposure has an important effect on the outcome?	No	

Y = Yes; PY = Probably yes; PN = Probably no; N = No; SN = Strong no; WN = Weak no; NA = Not applicable; NI = No information

Domain 4: Risk of bias due to post-exposure interventions

Signalling questions	Response options	Comments
4.1 Were there post-exposure interventions that were influenced by prior exposure during the follow-up period?	N	
4.2 **If Y/PY to 4.1**: Is it likely that the analysis corrected for the effect of post-exposure interventions that were influenced by prior exposure?	NA	
Risk of bias (due post-exposure interventions) in the estimated effect of exposure on the outcome	Low risk	
What is the predicted direction of bias due to confounding?	Towards null	
Is the risk of bias (due post-exposure interventions) sufficiently high, in the context of its likely direction and the magnitude of the estimated exposure effect, to threaten conclusions about whether the exposure has an important effect on the outcome?	No	

Y = Yes; PY = Probably yes; PN = Probably no; N = No; NA = Not applicable; NI = No information

Domain 5: Risk of bias due to missing data

Signalling questions	Response options	Comments
5.1 Were complete data on exposure status available for all, or nearly all, participants?	Y	
5.2 Were complete data on the outcome available for all, or nearly all, participants?	Y	
5.3 Were complete data on confounding variables available for all, or nearly all, participants?	Y	
5.4 **If N/PN/NI to 5.1, 5.2 or 5.3**: Is the result based on a complete case analysis?	NA	
5.5 **If Y/PY/NI**: Was exclusion from the analysis because of missing data (in exposure, confounders or the outcome) likely to be related to the true value of the outcome?	N	
5.6 **If N/PN to 5.5**: Were all or most predictors of missingness (in exposure, confounders or the outcome) included in the analysis model?	SY	
5.7 **If N/PN to 5.4**: Was the analysis based on imputing missing values?	NA	
5.8 **If Y/PY to 5.7**: Was imputation performed appropriately?	NA	
5.9 **If N/PN to 5.7**: Was an appropriate alternative method used to correct for bias due to missing data?	Y	
5.10 **If PN/N/NI to 5.1, 5.2 or 5.3**: Is there evidence that the result was not biased by missing data?	NA	
Risk of bias (due to missing data) in the estimated effect of exposure on the outcome	Low risk	
What is the predicted direction of bias due to missing data?	Towards null	
Is the risk of bias (due to missing data) sufficiently high, in the context of its likely direction and the magnitude of the estimated exposure effect, to threaten conclusions about whether the exposure has an important effect on the outcome?	No	

Y = Yes; PY = Probably yes; PN = Probably no; N = No; SY = Strong yes; WY = Weak yes; NA = Not applicable; NI = No information

Domain 6: Risk of bias arising from measurement of the outcome

Signalling questions	Response options	Comments
6.1 Could measurement or ascertainment of the outcome have differed between exposure groups or levels of exposure?	N	
6.2 Were outcome assessors aware of study participants' exposure history?	N	
6.3 **If Y/PY/NI to 6.2**: Could assessment of the outcome have been influenced by knowledge of participants' exposure history?	NA	

Signalling questions	Response options	Comments
Risk of bias (arising from measurement of outcomes) in the estimated effect of exposure on the outcome	Low risk	
What is the predicted direction of bias arising from measurement of outcomes?	Towards null	
Is the risk of bias (arising from measurement of outcomes) sufficiently high, in the context of its likely direction and the magnitude of the estimated exposure effect, to threaten conclusions about whether the exposure has an important effect on the outcome?	No	

Y = Yes; PY = Probably yes; PN = Probably no; N = No; SY = Strong yes; WY = Weak yes; NA = Not applicable; NI = No information

Domain 7: Risk of bias in selection of the reported result

Signalling questions	Response options	Comments
7.1 Was the result reported in accordance with an available, pre-determined analysis plan?	Y	
7.2 **If N/PN/NI to 7.1**: Is the reported effect estimate likely to be selected, based on desirability of the magnitude (or statistical significance) of the estimated effect of exposure on outcome, from multiple *exposure measurements* within the exposure domain?	N	
7.3 Is the reported effect estimate likely to be selected, based on desirability of the magnitude (or statistical significance) of the estimated effect of exposure on outcome, from multiple *outcome measurements* within the outcome domain?	N	
7.4 Is the reported effect estimate likely to be selected, based on desirability of the magnitude (or statistical significance) of the estimated effect of exposure on outcome, from multiple *analyses* of the exposure-outcome relationship?	N	
7.5 Is the reported effect estimate likely to be selected, based on the basis of desirability of the results (e.g. statistical significance), from different *subgroups*?	N	
Risk of bias (due to selection of the reported result) in the estimated effect of exposure on the outcome	Low risk	
What is the predicted direction of bias due to selection of the reported result?	Towards null	
Is the risk of bias (due to selection of the reported result) sufficiently high, in the context of its likely direction and the magnitude of the estimated exposure effect, to threaten conclusions about whether the exposure has an important effect on the outcome?	No	

Y = Yes; PY = Probably yes; PN = Probably no; N = No; NA = Not applicable; NI = No information

Overall risk of bias

	Response options	Comments
Overall risk of bias	Low risk of bias	
What is the predicted direction of bias?	Towards null	
Is the overall risk of bias sufficiently high, in the context of its likely direction and the magnitude of the estimated exposure effect, to threaten conclusions about whether the exposure has an important effect on the outcome?	No	

[32] Montastruc, F.; Salvo, F.; Arnaud, M.; Bégaud, B.; Pariente, A. Signal of gastrointestinal congenital malformations with antipsychotics after minimising competition bias: A disproportionality analysis using data from Vigibase(®). *Drug Saf.* **2016**, *39*(7), 689-696. doi: 10.1007/s40264-016-0413-1.

Domain 1: Risk of bias due to confounding*, variant (b): **If Y/PY to C7 and Y/PY to C8** (the analysis was based on splitting participants' follow up time according to exposure status and/or magnitude and changes in exposure status and/or magnitude likely to be related to factors that are predictive of the outcome, so both baseline and time-varying confounding need to be addressed)*

Signalling questions	Response options	Comments
1.1 Did the authors use an analysis method that was appropriate to control for time-varying as well as baseline confounding?	Y	
1.2 **If Y/PY to 1.1**: Did the authors control for all the important baseline and time-varying confounding factors for which this was necessary?	Y	
1.3 **If Y/PY/WN to 1.2**: Were confounding factors that were controlled for (and for which control was necessary) measured validly and reliably by the variables available in this study?	Y	
1.4 **If N/PN/NI to 1.1**: Did the authors control for time-varying factors or other variables measured after the start of the exposure window being studied?	NA	
1.5 Did the use of negative controls, or other considerations, suggest uncontrolled confounding?	PN	
Risk of bias (due to confounding) in the estimated effect of exposure on the outcome	Low risk	
What is the predicted direction of bias due to confounding?	Towards null	
Is the risk of bias (due to confounding) sufficiently high, in the context of its likely direction and the magnitude of the estimated exposure effect, to threaten conclusions about whether the exposure has an important effect on the outcome?	No	

Y = Yes; PY = Probably yes; PN = Probably no; N = No; SY = Strong yes; WY = Weak yes; SN = Strong no; WN = Weak no; NA = Not applicable; NI = No information

Domain 2: Risk of bias arising from measurement of the exposure *Variant (b): **If Y/PY to C5 and Y/PY to C6** (each individual's exposure level was estimated from measurements made at multiple time points)*

Signalling questions	Response options	Comments
**2.1 Does the measured exposure (derived from measurements at multiple time points) well-characterize the exposure metric specified to be of interest in this study? [*This was specified in the answers to D2, D3 and D4*]**	Y	

Signalling questions	Response options	Comments
**2.2 Was there error in measurement, or misclassification, of the exposure, at each single time point?**	**PN**	
**2.3 If SY/WY to 2.2: Could mismeasurement or misclassification of exposure have been differential (i.e. related to the outcome or risk of the outcome)?**	**NA**	
**2.4 If SY/WY to 2.2 and N/PN/WY to 2.3: Is the nature of the (non-differential) measurement error likely to bias the estimated effect of exposure on outcome?**	**NA**	
**Risk of bias (arising from measurement of exposure) in the estimated effect of exposure on the outcome**	**Low risk**	
**What is the predicted direction of bias arising from measurement of exposure?**	**Towards null**	
**Is the risk of bias (arising from measurement of exposure) sufficiently high, in the context of its likely direction and the magnitude of the estimated exposure effect, to threaten conclusions about whether the exposure has an important effect on the outcome?**	**No**	

Y = Yes; PY = Probably yes; SN = Strong no; WN = Weak no; NA = Not applicable; NI = No information

Domain 3: Risk of bias in selection of participants into the study (or into the analysis)

Signalling questions	Response options	Comments
3.1 Did follow-up begin at (or close to) the start of the exposure window for most participants? [*The exposure window is specified in D3*]	Y	
3.2 **If N/PN to 3.1**: Is the effect of exposure likely to be constant over the period of follow up analysed?	NA	
3.3 Was selection of participants into the study (or into the analysis) based on participant characteristics observed after the start of the exposure window being studied? [*The exposure window is specified in D3*]	N	
3.4 **If Y/PY to 3.3**: Were these characteristics likely to be influenced by exposure or a cause of exposure?	NA	
3.5 **If Y/PY to 3.4**: Were these characteristics likely to be influenced by the outcome or a cause of the outcome?	NA	
3.6 **If N/PN to 3.2 or Y/PY to 3.5**: Is it likely that the analysis corrected for all of the potential selection biases identified in A and B above?	NA	
3.7 **If N/PN to 3.2 or Y/PY to 3.5**: Did sensitivity analyses demonstrate that the likely impact of the potential selection biases identified in A or B above was minimal?	NA	
Risk of bias (due to selection of participants into the study) in the estimated effect of exposure on the outcome	Low risk	
What is the predicted direction of bias due to selection of participants into the study?	Towards null	
Is the risk of bias (due to selection of participants into the study) sufficiently high, in the context of its likely direction and the magnitude of the estimated exposure effect, to threaten conclusions about whether the exposure has an important effect on the outcome?	No	

Y = Yes; PY = Probably yes; PN = Probably no; N = No; SN = Strong no; WN = Weak no; NA = Not applicable; NI = No information

Domain 4: Risk of bias due to post-exposure interventions

Signalling questions	Response options	Comments
4.1 Were there post-exposure interventions that were influenced by prior exposure during the follow-up period?	N	
4.2 **If Y/PY to 4.1**: Is it likely that the analysis corrected for the effect of post-exposure interventions that were influenced by prior exposure?	NA	
Risk of bias (due post-exposure interventions) in the estimated effect of exposure on the outcome	Low risk	
What is the predicted direction of bias due to confounding?	Towards null	
Is the risk of bias (due post-exposure interventions) sufficiently high, in the context of its likely direction and the magnitude of the estimated exposure effect, to threaten conclusions about whether the exposure has an important effect on the outcome?	No	

Y = Yes; PY = Probably yes; PN = Probably no; N = No; NA = Not applicable; NI = No information

Domain 5: Risk of bias due to missing data

Signalling questions	Response options	Comments
5.1 Were complete data on exposure status available for all, or nearly all, participants?	Y	
5.2 Were complete data on the outcome available for all, or nearly all, participants?	Y	
5.3 Were complete data on confounding variables available for all, or nearly all, participants?	Y	
5.4 **If N/PN/NI to 5.1, 5.2 or 5.3**: Is the result based on a complete case analysis?	NA	
5.5 **If Y/PY/NI**: Was exclusion from the analysis because of missing data (in exposure, confounders or the outcome) likely to be related to the true value of the outcome?	N	
5.6 **If N/PN to 5.5**: Were all or most predictors of missingness (in exposure, confounders or the outcome) included in the analysis model?	SY	
5.7 **If N/PN to 5.4**: Was the analysis based on imputing missing values?	NA	
5.8 **If Y/PY to 5.7**: Was imputation performed appropriately?	NA	
5.9 **If N/PN to 5.7**: Was an appropriate alternative method used to correct for bias due to missing data?	Y	
5.10 **If PN/N/NI to 5.1, 5.2 or 5.3**: Is there evidence that the result was not biased by missing data?	NA	
Risk of bias (due to missing data) in the estimated effect of exposure on the outcome	Low risk	
What is the predicted direction of bias due to missing data?	Towards null	
Is the risk of bias (due to missing data) sufficiently high, in the context of its likely direction and the magnitude of the estimated exposure effect, to threaten conclusions about whether the exposure has an important effect on the outcome?	No	

Y = Yes; PY = Probably yes; PN = Probably no; N = No; SY = Strong yes; WY = Weak yes; NA = Not applicable; NI = No information

Domain 6: Risk of bias arising from measurement of the outcome

Signalling questions	Response options	Comments
6.1 Could measurement or ascertainment of the outcome have differed between exposure groups or levels of exposure?	N	
6.2 Were outcome assessors aware of study participants' exposure history?	N	
6.3 **If Y/PY/NI to 6.2**: Could assessment of the outcome have been influenced by knowledge of participants' exposure history?	NA	
Risk of bias (arising from measurement of outcomes) in the estimated effect of exposure on the outcome	Low risk	
What is the predicted direction of bias arising from measurement of outcomes?	Towards null	
Is the risk of bias (arising from measurement of outcomes) sufficiently high, in the context of its likely direction and the magnitude of the estimated exposure effect, to threaten conclusions about whether the exposure has an important effect on the outcome?	No	

Y = Yes; PY = Probably yes; PN = Probably no; N = No; SY = Strong yes; WY = Weak yes; NA = Not applicable; NI = No information

Domain 7: Risk of bias in selection of the reported result

Signalling questions	Response options	Comments
7.1 Was the result reported in accordance with an available, pre-determined analysis plan?	Y	
7.2 **If N/PN/NI to 7.1**: Is the reported effect estimate likely to be selected, based on desirability of the magnitude (or statistical significance) of the estimated effect of exposure on outcome, from multiple *exposure measurements* within the exposure domain?	N	
7.3 Is the reported effect estimate likely to be selected, based on desirability of the magnitude (or statistical significance) of the estimated effect of exposure on outcome, from multiple *outcome measurements* within the outcome domain?	N	
7.4 Is the reported effect estimate likely to be selected, based on desirability of the magnitude (or statistical significance) of the estimated effect of exposure on outcome, from multiple *analyses* of the exposure-outcome relationship?	N	
7.5 Is the reported effect estimate likely to be selected, based on the basis of desirability of the results (e.g. statistical significance), from different *subgroups*?	N	
Risk of bias (due to selection of the reported result) in the estimated effect of exposure on the outcome	Low risk	
What is the predicted direction of bias due to selection of the reported result?	Towards null	
Is the risk of bias (due to selection of the reported result) sufficiently high, in the context of its likely direction and the magnitude of the estimated exposure effect, to threaten conclusions about whether the exposure has an important effect on the outcome?	No	

Y = Yes; PY = Probably yes; PN = Probably no; N = No; NA = Not applicable; NI = No information

Overall risk of bias

	Response options	Comments
Overall risk of bias	Low risk of bias	
What is the predicted direction of bias?	Towards null	
Is the overall risk of bias sufficiently high, in the context of its likely direction and the magnitude of the estimated exposure effect, to threaten conclusions about whether the exposure has an important effect on the outcome?	No	

[33] Petersen, I.; McCrea, R.L.; Sammon, C.J.; Osborn, D.P.; Evans, S.J.; Cowen, P.J.; Freemantle, N.; Nazareth, I. Risks and benefits of psychotropic medication in pregnancy: cohort studies based on UK electronic primary care health records. *Health Technol. Assess.* **2016**, *20*(23), 1-176. doi: 10.3310/hta20230.

Domain 1: Risk of bias due to confounding, *variant (b): **If Y/PY to C7 and Y/PY to C8** (the analysis was based on splitting participants' follow up time according to exposure status and/or magnitude and changes in exposure status and/or magnitude likely to be related to factors that are predictive of the outcome, so both baseline and time-varying confounding need to be addressed)*

Signalling questions	Response options	Comments
1.1 Did the authors use an analysis method that was appropriate to control for time-varying as well as baseline confounding?	Y	
1.2 **If Y/PY to 1.1**: Did the authors control for all the important baseline and time-varying confounding factors for which this was necessary?	Y	
1.3 **If Y/PY/WN to 1.2**: Were confounding factors that were controlled for (and for which control was necessary) measured validly and reliably by the variables available in this study?	Y	
1.4 **If N/PN/NI to 1.1**: Did the authors control for time-varying factors or other variables measured after the start of the exposure window being studied?	NA	
1.5 Did the use of negative controls, or other considerations, suggest uncontrolled confounding?	N	
Risk of bias (due to confounding) in the estimated effect of exposure on the outcome	Low risk	
What is the predicted direction of bias due to confounding?	Towards null	
Is the risk of bias (due to confounding) sufficiently high, in the context of its likely direction and the magnitude of the estimated exposure effect, to threaten conclusions about whether the exposure has an important effect on the outcome?	No	

Y = Yes; PY = Probably yes; PN = Probably no; N = No; SY = Strong yes; WY = Weak yes; SN = Strong no; WN = Weak no; NA = Not applicable; NI = No information

Domain 2: Risk of bias arising from measurement of the exposure *Variant (b): **If Y/PY to C5 and Y/PY to C6** (each individual's exposure level was estimated from measurements made at multiple time points)*

Signalling questions	Response options	Comments
**2.1 Does the measured exposure (derived from measurements at multiple time points) well-characterize the exposure metric specified to be of interest in this study? [*This was specified in the answers to D2, D3 and D4*]**	**Y**	
**2.2 Was there error in measurement, or misclassification, of the exposure, at each single time point?**	**N**	
**2.3 If SY/WY to 2.2: Could mismeasurement or misclassification of exposure have been differential (i.e. related to the outcome or risk of the outcome)?**	**NA**	
**2.4 If SY/WY to 2.2 and N/PN/WY to 2.3: Is the nature of the (non-differential) measurement error likely to bias the estimated effect of exposure on outcome?**	**NA**	
**Risk of bias (arising from measurement of exposure) in the estimated effect of exposure on the outcome**	**Low risk**	
**What is the predicted direction of bias arising from measurement of exposure?**	**Towards null**	
**Is the risk of bias (arising from measurement of exposure) sufficiently high, in the context of its likely direction and the magnitude of the estimated exposure effect, to threaten conclusions about whether the exposure has an important effect on the outcome?**	**No**	

Y = Yes; PY = Probably yes; SN = Strong no; WN = Weak no; NA = Not applicable; NI = No information

Domain 3: Risk of bias in selection of participants into the study (or into the analysis)

Signalling questions	Response options	Comments
3.1 Did follow-up begin at (or close to) the start of the exposure window for most participants? [*The exposure window is specified in D3*]	Y	
3.2 **If N/PN to 3.1**: Is the effect of exposure likely to be constant over the period of follow up analysed?	NA	
3.3 Was selection of participants into the study (or into the analysis) based on participant characteristics observed after the start of the exposure window being studied? [*The exposure window is specified in D3*]	N	
3.4 **If Y/PY to 3.3**: Were these characteristics likely to be influenced by exposure or a cause of exposure?	NA	
3.5 **If Y/PY to 3.4**: Were these characteristics likely to be influenced by the outcome or a cause of the outcome?	NA	
3.6 **If N/PN to 3.2 or Y/PY to 3.5**: Is it likely that the analysis corrected for all of the potential selection biases identified in A and B above?	NA	
3.7 **If N/PN to 3.2 or Y/PY to 3.5**: Did sensitivity analyses demonstrate that the likely impact of the potential selection biases identified in A or B above was minimal?	NA	
Risk of bias (due to selection of participants into the study) in the estimated effect of exposure on the outcome	Low risk	
What is the predicted direction of bias due to selection of participants into the study?	Towards null	
Is the risk of bias (due to selection of participants into the study) sufficiently high, in the context of its likely direction and the magnitude of the estimated exposure effect, to threaten conclusions about whether the exposure has an important effect on the outcome?	No	

Y = Yes; PY = Probably yes; PN = Probably no; N = No; SN = Strong no; WN = Weak no; NA = Not applicable; NI = No information

Domain 4: Risk of bias due to post-exposure interventions

Signalling questions	Response options	Comments
4.1 Were there post-exposure interventions that were influenced by prior exposure during the follow-up period?	N	
4.2 **If Y/PY to 4.1**: Is it likely that the analysis corrected for the effect of post-exposure interventions that were influenced by prior exposure?	NA	
Risk of bias (due post-exposure interventions) in the estimated effect of exposure on the outcome	Low risk	
What is the predicted direction of bias due to confounding?	Towards null	
Is the risk of bias (due post-exposure interventions) sufficiently high, in the context of its likely direction and the magnitude of the estimated exposure effect, to threaten conclusions about whether the exposure has an important effect on the outcome?	No	

Y = Yes; PY = Probably yes; PN = Probably no; N = No; NA = Not applicable; NI = No information

Domain 5: Risk of bias due to missing data

Signalling questions	Response options	Comments
5.1 Were complete data on exposure status available for all, or nearly all, participants?	Y	
5.2 Were complete data on the outcome available for all, or nearly all, participants?	Y	
5.3 Were complete data on confounding variables available for all, or nearly all, participants?	Y	
5.4 **If N/PN/NI to 5.1, 5.2 or 5.3**: Is the result based on a complete case analysis?	NA	
5.5 **If Y/PY/NI**: Was exclusion from the analysis because of missing data (in exposure, confounders or the outcome) likely to be related to the true value of the outcome?	N	
5.6 **If N/PN to 5.5**: Were all or most predictors of missingness (in exposure, confounders or the outcome) included in the analysis model?	SY	
5.7 **If N/PN to 5.4**: Was the analysis based on imputing missing values?	NA	
5.8 **If Y/PY to 5.7**: Was imputation performed appropriately?	NA	

Signalling questions	Response options	Comments
5.9 **If N/PN to 5.7**: Was an appropriate alternative method used to correct for bias due to missing data?	Y	
5.10 **If PN/N/NI to 5.1, 5.2 or 5.3**: Is there evidence that the result was not biased by missing data?	NA	
Risk of bias (due to missing data) in the estimated effect of exposure on the outcome	Low risk	
What is the predicted direction of bias due to missing data?	Towards null	
Is the risk of bias (due to missing data) sufficiently high, in the context of its likely direction and the magnitude of the estimated exposure effect, to threaten conclusions about whether the exposure has an important effect on the outcome?	No	

Y = Yes; PY = Probably yes; PN = Probably no; N = No; SY = Strong yes; WY = Weak yes; NA = Not applicable; NI = No information

Domain 6: Risk of bias arising from measurement of the outcome

Signalling questions	Response options	Comments
6.1 Could measurement or ascertainment of the outcome have differed between exposure groups or levels of exposure?	N	
6.2 Were outcome assessors aware of study participants' exposure history?	PN	
6.3 **If Y/PY/NI to 6.2**: Could assessment of the outcome have been influenced by knowledge of participants' exposure history?	NA	
Risk of bias (arising from measurement of outcomes) in the estimated effect of exposure on the outcome	Low risk	
What is the predicted direction of bias arising from measurement of outcomes?	Towards null	
Is the risk of bias (arising from measurement of outcomes) sufficiently high, in the context of its likely direction and the magnitude of the estimated exposure effect, to threaten conclusions about whether the exposure has an important effect on the outcome?	No	

Y = Yes; PY = Probably yes; PN = Probably no; N = No; SY = Strong yes; WY = Weak yes; NA = Not applicable; NI = No information

Domain 7: Risk of bias in selection of the reported result

Signalling questions	Response options	Comments
7.1 Was the result reported in accordance with an available, pre-determined analysis plan?	Y	
7.2 **If N/PN/NI to 7.1**: Is the reported effect estimate likely to be selected, based on desirability of the magnitude (or statistical significance) of the estimated effect of exposure on outcome, from multiple *exposure measurements* within the exposure domain?	N	
7.3 Is the reported effect estimate likely to be selected, based on desirability of the magnitude (or statistical significance) of the estimated effect of exposure on outcome, from multiple *outcome measurements* within the outcome domain?	N	
7.4 Is the reported effect estimate likely to be selected, based on desirability of the magnitude (or statistical significance) of the estimated effect of exposure on outcome, from multiple *analyses* of the exposure-outcome relationship?	N	
7.5 Is the reported effect estimate likely to be selected, based on the basis of desirability of the results (e.g. statistical significance), from different *subgroups*?	PN	
Risk of bias (due to selection of the reported result) in the estimated effect of exposure on the outcome	Low risk	
What is the predicted direction of bias due to selection of the reported result?	Towards null	
Is the risk of bias (due to selection of the reported result) sufficiently high, in the context of its likely direction and the magnitude of the estimated exposure effect, to threaten conclusions about whether the exposure has an important effect on the outcome?	No	

Y = Yes; PY = Probably yes; PN = Probably no; N = No; NA = Not applicable; NI = No information

Overall risk of bias

	Response options	Comments
Overall risk of bias	Low risk of bias	
What is the predicted direction of bias?	Towards null	
Is the overall risk of bias sufficiently high, in the context of its likely direction and the magnitude of the estimated exposure effect, to threaten conclusions about whether the exposure has an important effect on the outcome?	No	

[34] Petersen, I.; Sammon, C.J.; McCrea, R.L.; Osborn, D.P.J.; Evans, S.J.; Cowen, P.J.; Nazareth, I., Risks associated with antipsychotic treatment in pregnancy: Comparative cohort studies based on electronic health records. *Schizophr. Res.* **2016**, *176*(2-3), 349-356. doi: 10.1016/j.schres.2016.07.023. Epub 2016 Jul 30.

Domain 1: Risk of bias due to confounding*, variant (b): **If Y/PY to C7 and Y/PY to C8** (the analysis was based on splitting participants' follow up time according to exposure status and/or magnitude and changes in exposure status and/or magnitude likely to be related to factors that are predictive of the outcome, so both baseline and time-varying confounding need to be addressed)*

Signalling questions	Response options	Comments
1.1 Did the authors use an analysis method that was appropriate to control for time-varying as well as baseline confounding?	Y	
1.2 **If Y/PY to 1.1**: Did the authors control for all the important baseline and time-varying confounding factors for which this was necessary?	PY	
1.3 **If Y/PY/WN to 1.2**: Were confounding factors that were controlled for (and for which control was necessary) measured validly and reliably by the variables available in this study?	Y	
1.4 **If N/PN/NI to 1.1**: Did the authors control for time-varying factors or other variables measured after the start of the	NA	

Signalling questions	Response options	Comments
exposure window being studied?		
1.5 Did the use of negative controls, or other considerations, suggest uncontrolled confounding?	N	
Risk of bias (due to confounding) in the estimated effect of exposure on the outcome	Low risk	
What is the predicted direction of bias due to confounding?	Towards null	
Is the risk of bias (due to confounding) sufficiently high, in the context of its likely direction and the magnitude of the estimated exposure effect, to threaten conclusions about whether the exposure has an important effect on the outcome?	No	

Y = Yes; PY = Probably yes; PN = Probably no; N = No; SY = Strong yes; WY = Weak yes; SN = Strong no; WN = Weak no; NA = Not applicable; NI = No information

Domain 2: Risk of bias arising from measurement of the exposure *Variant (b): If Y/PY to C5 and Y/PY to C6 (each individual's exposure level was estimated from measurements made at multiple time points)*

Signalling questions	Response options	Comments
**2.1 Does the measured exposure (derived from measurements at multiple time points) well-characterize the exposure metric specified to be of interest in this study? [*This was specified in the answers to D2, D3 and D4*]**	**Y**	
**2.2 Was there error in measurement, or misclassification, of the exposure, at each single time point?**	**N**	
**2.3 If SY/WY to 2.2: Could mismeasurement or misclassification of exposure have been differential (i.e. related to the outcome or risk of the outcome)?**	**NA**	
**2.4 If SY/WY to 2.2 and N/PN/WY to 2.3: Is the nature of the (non-differential) measurement error likely to bias the estimated effect of exposure on outcome?**	**NA**	
**Risk of bias (arising from measurement of exposure) in the estimated effect of exposure on the outcome**	**Low risk**	
**What is the predicted direction of bias arising from measurement of exposure?**	**Towards null**	
**Is the risk of bias (arising from measurement of exposure) sufficiently high, in the context of its likely direction and the magnitude of the estimated exposure effect, to threaten conclusions about whether the exposure has an important effect on the outcome?**	**No**	

Y = Yes; PY = Probably yes; SN = Strong no; WN = Weak no; NA = Not applicable; NI = No information

Domain 3: Risk of bias in selection of participants into the study (or into the analysis)

Signalling questions	Response options	Comments
3.1 Did follow-up begin at (or close to) the start of the exposure window for most participants? [*The exposure window is specified in D3*]	Y	
3.2 **If N/PN to 3.1**: Is the effect of exposure likely to be constant over the period of follow up analysed?	NA	
3.3 Was selection of participants into the study (or into the analysis) based on participant characteristics observed after the start of the exposure window being studied? [*The exposure window is specified in D3*]	N	
3.4 **If Y/PY to 3.3**: Were these characteristics likely to be influenced by exposure or a cause of exposure?	NA	
3.5 **If Y/PY to 3.4**: Were these characteristics likely to be influenced by the outcome or a cause of the outcome?	NA	
3.6 **If N/PN to 3.2 or Y/PY to 3.5**: Is it likely that the analysis corrected for all of the potential selection biases identified in A and B above?	NA	
3.7 **If N/PN to 3.2 or Y/PY to 3.5**: Did sensitivity analyses demonstrate that the likely impact of the potential selection biases identified in A or B above was minimal?	NA	
Risk of bias (due to selection of participants into the study) in the estimated effect of exposure on the outcome	Low risk	
What is the predicted direction of bias due to selection of participants into the study?	Towards null	
Is the risk of bias (due to selection of participants into the study) sufficiently high, in the context of its likely direction and the magnitude of the estimated exposure effect, to threaten conclusions about whether the exposure has an important effect on the outcome?	No	

Y = Yes; PY = Probably yes; PN = Probably no; N = No; SN = Strong no; WN = Weak no; NA = Not applicable; NI = No information

Domain 4: Risk of bias due to post-exposure interventions

Signalling questions	Response options	Comments
4.1 Were there post-exposure interventions that were influenced by prior exposure during the follow-up period?	N	
4.2 **If Y/PY to 4.1**: Is it likely that the analysis corrected for the effect of post-exposure interventions that were influenced by prior exposure?	NA	
Risk of bias (due post-exposure interventions) in the estimated effect of exposure on the outcome	Low risk	
What is the predicted direction of bias due to confounding?	Towards null	
Is the risk of bias (due post-exposure interventions) sufficiently high, in the context of its likely direction and the magnitude of the estimated exposure effect, to threaten conclusions about whether the exposure has an important effect on the outcome?	No	

Y = Yes; PY = Probably yes; PN = Probably no; N = No; NA = Not applicable; NI = No information

Domain 5: Risk of bias due to missing data

Signalling questions	Response options	Comments
5.1 Were complete data on exposure status available for all, or nearly all, participants?	Y	
5.2 Were complete data on the outcome available for all, or nearly all, participants?	Y	
5.3 Were complete data on confounding variables available for all, or nearly all, participants?	Y	
5.4 **If N/PN/NI to 5.1, 5.2 or 5.3**: Is the result based on a complete case analysis?	NA	
5.5 **If Y/PY/NI**: Was exclusion from the analysis because of missing data (in exposure, confounders or the outcome) likely to be related to the true value of the outcome?	N	
5.6 **If N/PN to 5.5**: Were all or most predictors of missingness (in exposure, confounders or the outcome) included in the analysis model?	SY	
5.7 **If N/PN to 5.4**: Was the analysis based on imputing missing values?	NA	
5.8 **If Y/PY to 5.7**: Was imputation performed appropriately?	NA	
5.9 **If N/PN to 5.7**: Was an appropriate alternative method used to correct for bias due to missing data?	Y	
5.10 **If PN/N/NI to 5.1, 5.2 or 5.3**: Is there evidence that the result was not biased by missing data?	NA	
Risk of bias (due to missing data) in the estimated effect of exposure on the outcome	Low risk	
What is the predicted direction of bias due to missing data?	Towards null	
Is the risk of bias (due to missing data) sufficiently high, in the context of its likely direction and the magnitude of the estimated exposure effect, to threaten conclusions about whether the exposure has an important effect on the outcome?	No	

Y = Yes; PY = Probably yes; PN = Probably no; N = No; SY = Strong yes; WY = Weak yes; NA = Not applicable; NI = No information

Domain 6: Risk of bias arising from measurement of the outcome

Signalling questions	Response options	Comments
6.1 Could measurement or ascertainment of the outcome have differed between exposure groups or levels of exposure?	N	
6.2 Were outcome assessors aware of study participants' exposure history?	N	
6.3 **If Y/PY/NI to 6.2**: Could assessment of the outcome have been influenced by knowledge of participants' exposure history?	NA	
Risk of bias (arising from measurement of outcomes) in the estimated effect of exposure on the outcome	Low risk	
What is the predicted direction of bias arising from measurement of outcomes?	Towards null	
Is the risk of bias (arising from measurement of outcomes) sufficiently high, in the context of its likely direction and the magnitude of the estimated exposure effect, to threaten conclusions about whether the exposure has an important effect on the outcome?	No	

Y = Yes; PY = Probably yes; PN = Probably no; N = No; SY = Strong yes; WY = Weak yes; NA = Not applicable; NI = No information

Domain 7: Risk of bias in selection of the reported result

Signalling questions	Response options	Comments
7.1 Was the result reported in accordance with an available, pre-determined analysis plan?	Y	
7.2 **If N/PN/NI to 7.1**: Is the reported effect estimate likely to be selected, based on desirability of the magnitude (or statistical significance) of the estimated effect of exposure on outcome, from multiple *exposure measurements* within the exposure domain?	N	
7.3 Is the reported effect estimate likely to be selected, based on desirability of the magnitude (or statistical significance) of the estimated effect of exposure on outcome, from multiple *outcome measurements* within the outcome domain?	N	
7.4 Is the reported effect estimate likely to be selected, based on desirability of the magnitude (or statistical significance) of the estimated effect of exposure on outcome, from multiple *analyses* of the exposure-outcome relationship?	N	
7.5 Is the reported effect estimate likely to be selected, based on the basis of desirability of the results (e.g. statistical significance), from different *subgroups*?	N	
Risk of bias (due to selection of the reported result) in the estimated effect of exposure on the outcome	Low risk	
What is the predicted direction of bias due to selection of the reported result?	Towards null	
Is the risk of bias (due to selection of the reported result) sufficiently high, in the context of its likely direction and the magnitude of the estimated exposure effect, to threaten conclusions about whether the exposure has an important effect on the outcome?	No	

Y = Yes; PY = Probably yes; PN = Probably no; N = No; NA = Not applicable; NI = No information

Overall risk of bias

	Response options	Comments
Overall risk of bias	Low risk of bias	
What is the predicted direction of bias?	Towards null	
Is the overall risk of bias sufficiently high, in the context of its likely direction and the magnitude of the estimated exposure effect, to threaten conclusions about whether the exposure has an important effect on the outcome?	No	

[35] Huybrechts, K.F.; Hernández-Díaz, S.; Patorno, E.; Desai, R.J.; Mogun, H.; Dejene, S.Z.; Cohen, J.M.; Panchaud, A.; Cohen, L.; Bateman, B.T. Antipsychotic use in pregnancy and the risk for congenital malformations. *JAMA Psychiatry* **2016**, *73*(9), 938-946. doi: 10.1001/jamapsychiatry.2016.1520.

Domain 1: Risk of bias due to confounding, *variant (b): **If Y/PY to C7 and Y/PY to C8** (the analysis was based on splitting participants' follow up time according to exposure status and/or magnitude and changes in exposure status and/or magnitude likely to be related to factors that are predictive of the outcome, so both baseline and time-varying confounding need to be addressed)*

Signalling questions	Response options	Comments
1.1 Did the authors use an analysis method that was appropriate to control for time-varying as well as baseline confounding?	Y	
1.2 **If Y/PY to 1.1**: Did the authors control for all the important baseline and time-varying confounding factors for which this was necessary?	Y	
1.3 **If Y/PY/WN to 1.2**: Were confounding factors that were controlled for (and for which control was necessary) measured validly and reliably by the variables available in this study?	Y	
1.4 **If N/PN/NI to 1.1**: Did the authors control for time-varying factors or other variables measured after the start of the exposure window being studied?	NA	
1.5 Did the use of negative controls, or other considerations, suggest uncontrolled confounding?	N	
Risk of bias (due to confounding) in the estimated effect of exposure on the outcome	Low risk	
What is the predicted direction of bias due to confounding?	Towards null	
Is the risk of bias (due to confounding) sufficiently high, in the context of its likely direction and the magnitude of the estimated exposure effect, to threaten conclusions about whether the exposure has an important effect on the outcome?	No	

Y = Yes; PY = Probably yes; PN = Probably no; N = No; SY = Strong yes; WY = Weak yes; SN = Strong no; WN = Weak no; NA = Not applicable; NI = No information

Domain 2: Risk of bias arising from measurement of the exposure *Variant (b): **If Y/PY to C5 and Y/PY to C6** (each individual's exposure level was estimated from measurements made at multiple time points)*

Signalling questions	Response options	Comments
**2.1 Does the measured exposure (derived from measurements at multiple time points) well-characterize the exposure metric specified to be of interest in this study? [*This was specified in the answers to D2, D3 and D4*]**	**Y**	
**2.2 Was there error in measurement, or misclassification, of the exposure, at each single time point?**	**PN**	
**2.3 If SY/WY to 2.2: Could mismeasurement or misclassification of exposure have been differential (i.e. related to the outcome or risk of the outcome)?**	**NA**	
**2.4 If SY/WY to 2.2 and N/PN/WY to 2.3: Is the nature of the (non-differential) measurement error likely to bias the estimated effect of exposure on outcome?**	**NA**	
**Risk of bias (arising from measurement of exposure) in the estimated effect of exposure on the outcome**	**Low risk**	
**What is the predicted direction of bias arising from measurement of exposure?**	**Towards null**	
**Is the risk of bias (arising from measurement of exposure) sufficiently high, in the context of its likely direction and the magnitude of the estimated exposure effect, to threaten conclusions about whether the exposure has an important effect on the outcome?**	**No**	

Y = Yes; PY = Probably yes; SN = Strong no; WN = Weak no; NA = Not applicable; NI = No information

Domain 3: Risk of bias in selection of participants into the study (or into the analysis)

Signalling questions	Response options	Comments
3.1 Did follow-up begin at (or close to) the start of the exposure window for most participants? [*The exposure window is specified in D3*]	Y	
3.2 **If N/PN to 3.1**: Is the effect of exposure likely to be constant over the period of follow up analysed?	NA	
3.3 Was selection of participants into the study (or into the analysis) based on participant characteristics observed after the start of the exposure window being studied? [*The exposure window is specified in D3*]	PN	
3.4 **If Y/PY to 3.3**: Were these characteristics likely to be influenced by exposure or a cause of exposure?	NA	
3.5 **If Y/PY to 3.4**: Were these characteristics likely to be influenced by the outcome or a cause of the outcome?	NA	
3.6 **If N/PN to 3.2 or Y/PY to 3.5**: Is it likely that the analysis corrected for all of the potential selection biases identified in A and B above?	NA	
3.7 **If N/PN to 3.2 or Y/PY to 3.5**: Did sensitivity analyses demonstrate that the likely impact of the potential selection biases identified in A or B above was minimal?	NA	
Risk of bias (due to selection of participants into the study) in the estimated effect of exposure on the outcome	Low risk	
What is the predicted direction of bias due to selection of participants into the study?	Towards null	
Is the risk of bias (due to selection of participants into the study) sufficiently high, in the context of its likely direction and the magnitude of the estimated exposure effect, to threaten conclusions about whether the exposure has an important effect on the outcome?	No	

Y = Yes; PY = Probably yes; PN = Probably no; N = No; SN = Strong no; WN = Weak no; NA = Not applicable; NI = No information

Domain 4: Risk of bias due to post-exposure interventions

Signalling questions	Response options	Comments
4.1 Were there post-exposure interventions that were influenced by prior exposure during the follow-up period?	N	

4.2 **If Y/PY to 4.1**: Is it likely that the analysis corrected for the effect of post-exposure interventions that were influenced by prior exposure?	NA	
Risk of bias (due post-exposure interventions) in the estimated effect of exposure on the outcome	Low risk	
What is the predicted direction of bias due to confounding?	Towards null	
Is the risk of bias (due post-exposure interventions) sufficiently high, in the context of its likely direction and the magnitude of the estimated exposure effect, to threaten conclusions about whether the exposure has an important effect on the outcome?	No	

Y = Yes; PY = Probably yes; PN = Probably no; N = No; NA = Not applicable; NI = No information

Domain 5: Risk of bias due to missing data

**Signalling questions**	**Response options**	**Comments**
5.1 Were complete data on exposure status available for all, or nearly all, participants?	Y	
5.2 Were complete data on the outcome available for all, or nearly all, participants?	Y	
5.3 Were complete data on confounding variables available for all, or nearly all, participants?	Y	
5.4 **If N/PN/NI to 5.1, 5.2 or 5.3**: Is the result based on a complete case analysis?	NA	
5.5 **If Y/PY/NI**: Was exclusion from the analysis because of missing data (in exposure, confounders or the outcome) likely to be related to the true value of the outcome?	N	
5.6 **If N/PN to 5.5**: Were all or most predictors of missingness (in exposure, confounders or the outcome) included in the analysis model?	SY	
5.7 **If N/PN to 5.4**: Was the analysis based on imputing missing values?	NA	
5.8 **If Y/PY to 5.7**: Was imputation performed appropriately?	NA	
5.9 **If N/PN to 5.7**: Was an appropriate alternative method used to correct for bias due to missing data?	Y	
5.10 **If PN/N/NI to 5.1, 5.2 or 5.3**: Is there evidence that the result was not biased by missing data?	NA	
Risk of bias (due to missing data) in the estimated effect of exposure on the outcome	Low risk	
What is the predicted direction of bias due to missing data?	Towards null	
Is the risk of bias (due to missing data) sufficiently high, in the context of its likely direction and the magnitude of the estimated exposure effect, to threaten conclusions about whether the exposure has an important effect on the outcome?	No	

Y = Yes; PY = Probably yes; PN = Probably no; N = No; SY = Strong yes; WY = Weak yes; NA = Not applicable; NI = No information

Domain 6: Risk of bias arising from measurement of the outcome

**Signalling questions**	**Response options**	**Comments**
6.1 Could measurement or ascertainment of the outcome have differed between exposure groups or levels of exposure?	N	
6.2 Were outcome assessors aware of study participants' exposure history?	N	
6.3 **If Y/PY/NI to 6.2**: Could assessment of the outcome have been influenced by knowledge of participants' exposure history?	NA	
Risk of bias (arising from measurement of outcomes) in the estimated effect of exposure on the outcome	Low risk	
What is the predicted direction of bias arising from measurement of outcomes?	Towards null	
Is the risk of bias (arising from measurement of outcomes) sufficiently high, in the context of its likely direction and the magnitude of the estimated exposure effect, to threaten conclusions about whether the exposure has an important effect on the outcome?	No	

Y = Yes; PY = Probably yes; PN = Probably no; N = No; SY = Strong yes; WY = Weak yes; NA = Not applicable; NI = No information

Domain 7: Risk of bias in selection of the reported result

**Signalling questions**	**Response options**	**Comments**
7.1 Was the result reported in accordance with an available, pre-determined analysis plan?	Y	
7.2 **If N/PN/NI to 7.1**: Is the reported effect estimate likely to be selected, based on desirability of the magnitude (or statistical significance) of the estimated effect of exposure on outcome, from multiple *exposure measurements* within the exposure domain?	N	
7.3 Is the reported effect estimate likely to be selected, based on desirability of the magnitude (or statistical significance) of the estimated effect of exposure on outcome, from multiple *outcome measurements* within the outcome domain?	N	
7.4 Is the reported effect estimate likely to be selected, based on desirability of the magnitude (or statistical significance) of the estimated effect of exposure on outcome, from multiple *analyses* of the exposure-outcome relationship?	N	
7.5 Is the reported effect estimate likely to be selected, based on the basis of desirability of the results (e.g. statistical significance), from different *subgroups*?	N	
Risk of bias (due to selection of the reported result) in the estimated effect of exposure on the outcome	Low risk	
What is the predicted direction of bias due to selection of the reported result?	Towards null	
Is the risk of bias (due to selection of the reported result) sufficiently high, in the context of its likely direction and the magnitude of the estimated exposure effect, to threaten conclusions about whether the exposure has an important effect on the outcome?	No	

Y = Yes; PY = Probably yes; PN = Probably no; N = No; NA = Not applicable; NI = No information

Overall risk of bias

	**Response options**	**Comments**
Overall risk of bias	Low risk of bias	
What is the predicted direction of bias?	Towards null	

Is the overall risk of bias sufficiently high, in the context of its likely direction and the magnitude of the estimated exposure effect, to threaten conclusions about whether the exposure has an important effect on the outcome?	No	

[36] Hatters Friedman, S.; Moller-Olsen, C.; Prakash, C.; North, A. Atypical antipsychotic use and outcomes in an urban maternal mental health service. *Int. J. Psychiatry Med.* **2016**, *51*(6), 521-533. doi: 10.1177/0091217417696739. Epub 2017 Mar 6.

Domain 1: Risk of bias due to confounding, *variant (b): **If Y/PY to C7 and Y/PY to C8** (the analysis was based on splitting participants' follow up time according to exposure status and/or magnitude and changes in exposure status and/or magnitude likely to be related to factors that are predictive of the outcome, so both baseline and time-varying confounding need to be addressed)*

Signalling questions	Response options	Comments
1.1 Did the authors use an analysis method that was appropriate to control for time-varying as well as baseline confounding?	Y	
1.2 **If Y/PY to 1.1**: Did the authors control for all the important baseline and time-varying confounding factors for which this was necessary?	Y	
1.3 **If Y/PY/WN to 1.2**: Were confounding factors that were controlled for (and for which control was necessary) measured validly and reliably by the variables available in this study?	Y	
1.4 **If N/PN/NI to 1.1**: Did the authors control for time-varying factors or other variables measured after the start of the exposure window being studied?	NA	
1.5 Did the use of negative controls, or other considerations, suggest uncontrolled confounding?	N	
Risk of bias (due to confounding) in the estimated effect of exposure on the outcome	Low risk	
What is the predicted direction of bias due to confounding?	Towards null	
Is the risk of bias (due to confounding) sufficiently high, in the context of its likely direction and the magnitude of the estimated exposure effect, to threaten conclusions about whether the exposure has an important effect on the outcome?	No	

Y = Yes; PY = Probably yes; PN = Probably no; N = No; SY = Strong yes; WY = Weak yes; SN = Strong no; WN = Weak no; NA = Not applicable; NI = No information

Domain 2: Risk of bias arising from measurement of the exposure *Variant (b): **If Y/PY to C5 and Y/PY to C6** (each individual's exposure level was estimated from measurements made at multiple time points)*

Signalling questions	Response options	Comments
**2.1 Does the measured exposure (derived from measurements at multiple time points) well-characterize the exposure metric specified to be of interest in this study? [*This was specified in the answers to D2, D3 and D4*]**	**Y**	
**2.2 Was there error in measurement, or misclassification, of the exposure, at each single time point?**	**N**	
**2.3 If SY/WY to 2.2: Could mismeasurement or misclassification of exposure have been differential (i.e. related to the outcome or risk of the outcome)?**	**NA**	
**2.4 If SY/WY to 2.2 and N/PN/WY to 2.3: Is the nature of the (non-differential) measurement error likely to bias the estimated effect of exposure on outcome?**	**NA**	
**Risk of bias (arising from measurement of exposure) in the estimated effect of exposure on the outcome**	**Low risk**	
**What is the predicted direction of bias arising from measurement of exposure?**	**Towards null**	
**Is the risk of bias (arising from measurement of exposure) sufficiently high, in the context of its likely direction and the magnitude of the estimated exposure effect, to threaten conclusions about whether the exposure has an important effect on the outcome?**	**No**	

Y = Yes; PY = Probably yes; SN = Strong no; WN = Weak no; NA = Not applicable; NI = No information

Domain 3: Risk of bias in selection of participants into the study (or into the analysis)

Signalling questions	Response options	Comments
3.1 Did follow-up begin at (or close to) the start of the exposure window for most participants? [*The exposure window is specified in D3*]	Y	
3.2 **If N/PN to 3.1**: Is the effect of exposure likely to be constant over the period of follow up analysed?	NA	
3.3 Was selection of participants into the study (or into the analysis) based on participant characteristics observed after the start of the exposure window being studied? [*The exposure window is specified in D3*]	N	
3.4 **If Y/PY to 3.3**: Were these characteristics likely to be influenced by exposure or a cause of exposure?	NA	
3.5 **If Y/PY to 3.4**: Were these characteristics likely to be influenced by the outcome or a cause of the outcome?	NA	
3.6 **If N/PN to 3.2 or Y/PY to 3.5**: Is it likely that the analysis corrected for all of the potential selection biases identified in A and B above?	NA	
3.7 **If N/PN to 3.2 or Y/PY to 3.5**: Did sensitivity analyses demonstrate that the likely impact of the potential selection biases identified in A or B above was minimal?	NA	
Risk of bias (due to selection of participants into the study) in the estimated effect of exposure on the outcome	Low risk	
What is the predicted direction of bias due to selection of participants into the study?	Towards null	
Is the risk of bias (due to selection of participants into the study) sufficiently high, in the context of its likely direction and the magnitude of the estimated exposure effect, to threaten conclusions about whether the exposure has an important effect on the outcome?	No	

Y = Yes; PY = Probably yes; PN = Probably no; N = No; SN = Strong no; WN = Weak no; NA = Not applicable; NI = No information

Domain 4: Risk of bias due to post-exposure interventions

Signalling questions	Response options	Comments
4.1 Were there post-exposure interventions that were influenced by prior exposure during the follow-up period?	N	
4.2 **If Y/PY to 4.1**: Is it likely that the analysis corrected for the effect of post-exposure interventions that were influenced by prior exposure?	NA	
Risk of bias (due post-exposure interventions) in the estimated effect of exposure on the outcome	Low risk	
What is the predicted direction of bias due to confounding?	Towards null	
Is the risk of bias (due post-exposure interventions) sufficiently high, in the context of its likely direction and the magnitude of the estimated exposure effect, to threaten conclusions about whether the exposure has an important effect on the outcome?	No	

Y = Yes; PY = Probably yes; PN = Probably no; N = No; NA = Not applicable; NI = No information

Domain 5: Risk of bias due to missing data

Signalling questions	Response options	Comments
5.1 Were complete data on exposure status available for all, or nearly all, participants?	Y	
5.2 Were complete data on the outcome available for all, or nearly all, participants?	Y	
5.3 Were complete data on confounding variables available for all, or nearly all, participants?	Y	
5.4 **If N/PN/NI to 5.1, 5.2 or 5.3**: Is the result based on a complete case analysis?	NA	
5.5 **If Y/PY/NI**: Was exclusion from the analysis because of missing data (in exposure, confounders or the outcome) likely to be related to the true value of the outcome?	N	
5.6 **If N/PN to 5.5**: Were all or most predictors of missingness (in exposure, confounders or the outcome) included in the analysis model?	SY	
5.7 **If N/PN to 5.4**: Was the analysis based on imputing missing values?	NA	
5.8 **If Y/PY to 5.7**: Was imputation performed appropriately?	NA	
5.9 **If N/PN to 5.7**: Was an appropriate alternative method used to correct for bias due to missing data?	Y	
5.10 **If PN/N/NI to 5.1, 5.2 or 5.3**: Is there evidence that the result was not biased by missing data?	NA	
Risk of bias (due to missing data) in the estimated effect of exposure on the outcome	Low risk	
What is the predicted direction of bias due to missing data?	Towards null	
Is the risk of bias (due to missing data) sufficiently high, in the context of its likely direction and the magnitude of the estimated exposure effect, to threaten conclusions about whether the exposure has an important effect on the outcome?	No	

Y = Yes; PY = Probably yes; PN = Probably no; N = No; SY = Strong yes; WY = Weak yes; NA = Not applicable; NI = No information

Domain 6: Risk of bias arising from measurement of the outcome

Signalling questions	Response options	Comments
6.1 Could measurement or ascertainment of the outcome have differed between exposure groups or levels of exposure?	N	
6.2 Were outcome assessors aware of study participants' exposure history?	N	
6.3 **If Y/PY/NI to 6.2**: Could assessment of the outcome have been influenced by knowledge of participants' exposure history?	NA	
Risk of bias (arising from measurement of outcomes) in the estimated effect of exposure on the outcome	Low risk	
What is the predicted direction of bias arising from measurement of outcomes?	Towards null	
Is the risk of bias (arising from measurement of outcomes) sufficiently high, in the context of its likely direction and the magnitude of the estimated exposure effect, to threaten conclusions about whether the exposure has an important effect on the outcome?	No	

Y = Yes; PY = Probably yes; PN = Probably no; N = No; SY = Strong yes; WY = Weak yes; NA = Not applicable; NI = No information

Domain 7: Risk of bias in selection of the reported result

Signalling questions	Response options	Comments
7.1 Was the result reported in accordance with an available, pre-determined analysis plan?	Y	
7.2 **If N/PN/NI to 7.1**: Is the reported effect estimate likely to be selected, based on desirability of the magnitude (or statistical significance) of the estimated effect of exposure on outcome, from multiple *exposure measurements* within the exposure domain?	N	
7.3 Is the reported effect estimate likely to be selected, based on desirability of the magnitude (or statistical significance) of the estimated effect of exposure on outcome, from multiple *outcome measurements* within the outcome domain?	N	
7.4 Is the reported effect estimate likely to be selected, based on desirability of the magnitude (or statistical significance) of the estimated effect of exposure on outcome, from multiple *analyses* of the exposure-outcome relationship?	N	
7.5 Is the reported effect estimate likely to be selected, based on the basis of desirability of the results (e.g. statistical significance), from different *subgroups*?	N	
Risk of bias (due to selection of the reported result) in the estimated effect of exposure on the outcome	Low risk	
What is the predicted direction of bias due to selection of the reported result?	Towards null	
Is the risk of bias (due to selection of the reported result) sufficiently high, in the context of its likely direction and the magnitude of the estimated exposure effect, to threaten conclusions about whether the exposure has an important effect on the outcome?	No	

Y = Yes; PY = Probably yes; PN = Probably no; N = No; NA = Not applicable; NI = No information

Overall risk of bias

	Response options	Comments
Overall risk of bias	Low risk of bias	
What is the predicted direction of bias?	Towards null	
Is the overall risk of bias sufficiently high, in the context of its likely direction and the magnitude of the estimated exposure effect, to threaten conclusions about whether the exposure has an important effect on the outcome?	No	

[37] Shin, Y.J.; Choi, J.S.; Ahn, H.K.; Ryu, H.M.; Kim, M.Y.; Han, J.Y. Pregnancy outcomes in women reporting ingestion of levosulpiride in early pregnancy. *J. Obstet. Gynaecol.* **2017**, *37*(8), 992-995. doi: 10.1080/01443615.2017.1312307. Epub 2017 Jun 20.

Domain 1: Risk of bias due to confounding, *variant (b): **If Y/PY to C7 and Y/PY to C8** (the analysis was based on splitting participants' follow up time according to exposure status and/or magnitude and changes in exposure status and/or magnitude likely to be related to factors that are predictive of the outcome, so both baseline and time-varying confounding need to be addressed)*

Signalling questions	Response options	Comments
1.1 Did the authors use an analysis method that was appropriate to control for time-varying as well as baseline confounding?	Y	
1.2 **If Y/PY to 1.1**: Did the authors control for all the important baseline and time-varying confounding factors for which this was necessary?	Y	
1.3 **If Y/PY/WN to 1.2**: Were confounding factors that were controlled for (and for which control was necessary) measured validly and reliably by the variables available in this study?	Y	
1.4 **If N/PN/NI to 1.1**: Did the authors control for time-varying factors or other variables measured after the start of the exposure window being studied?	NA	
1.5 Did the use of negative controls, or other considerations, suggest uncontrolled confounding?	N	
Risk of bias (due to confounding) in the estimated effect of exposure on the outcome	Low risk	
What is the predicted direction of bias due to confounding?	Towards null	
Is the risk of bias (due to confounding) sufficiently high, in the context of its likely direction and the magnitude of the estimated exposure effect, to threaten conclusions about whether the exposure has an important effect on the outcome?	No	

Y = Yes; PY = Probably yes; PN = Probably no; N = No; SY = Strong yes; WY = Weak yes; SN = Strong no; WN = Weak no; NA = Not applicable; NI = No information

Domain 2: Risk of bias arising from measurement of the exposure *Variant (b): **If Y/PY to C5 and Y/PY to C6** (each individual's exposure level was estimated from measurements made at multiple time points)*

Signalling questions	Response options	Comments
**2.1 Does the measured exposure (derived from measurements at multiple time points) well-characterize the exposure metric specified to be of interest in this study? [*This was specified in the answers to D2, D3 and D4*]**	**Y**	
**2.2 Was there error in measurement, or misclassification, of the exposure, at each single time point?**	**PN**	
**2.3 If SY/WY to 2.2: Could mismeasurement or misclassification of exposure have been differential (i.e. related to the outcome or risk of the outcome)?**	**NA**	
**2.4 If SY/WY to 2.2 and N/PN/WY to 2.3: Is the nature of the (non-differential) measurement error likely to bias the estimated effect of exposure on outcome?**	**NA**	
**Risk of bias (arising from measurement of exposure) in the estimated effect of exposure on the outcome**	**Low risk**	
**What is the predicted direction of bias arising from measurement of exposure?**	**Towards null**	
**Is the risk of bias (arising from measurement of exposure) sufficiently high, in the context of its likely direction and the magnitude of the estimated exposure effect, to threaten conclusions about whether the exposure has an important effect on the outcome?**	**No**	

Y = Yes; PY = Probably yes; SN = Strong no; WN = Weak no; NA = Not applicable; NI = No information

Domain 3: Risk of bias in selection of participants into the study (or into the analysis)

Signalling questions	Response options	Comments
3.1 Did follow-up begin at (or close to) the start of the exposure window for most participants? [*The exposure window is specified in D3*]	Y	
3.2 **If N/PN to 3.1**: Is the effect of exposure likely to be constant over the period of follow up analysed?	NA	
3.3 Was selection of participants into the study (or into the analysis) based on participant characteristics observed after the start of the exposure window being studied? [*The exposure window is specified in D3*]	N	
3.4 **If Y/PY to 3.3**: Were these characteristics likely to be influenced by exposure or a cause of exposure?	NA	
3.5 **If Y/PY to 3.4**: Were these characteristics likely to be influenced by the outcome or a cause of the outcome?	NA	
3.6 **If N/PN to 3.2 or Y/PY to 3.5**: Is it likely that the analysis corrected for all of the potential selection biases identified in A and B above?	NA	

**Signalling questions**	**Response options**	**Comments**
3.7 **If N/PN to 3.2 or Y/PY to 3.5**: Did sensitivity analyses demonstrate that the likely impact of the potential selection biases identified in A or B above was minimal?	NA	
Risk of bias (due to selection of participants into the study) in the estimated effect of exposure on the outcome	Low risk	
What is the predicted direction of bias due to selection of participants into the study?	Towards null	
Is the risk of bias (due to selection of participants into the study) sufficiently high, in the context of its likely direction and the magnitude of the estimated exposure effect, to threaten conclusions about whether the exposure has an important effect on the outcome?	No	

Y = Yes; PY = Probably yes; PN = Probably no; N = No; SN = Strong no; WN = Weak no; NA = Not applicable; NI = No information

Domain 4: Risk of bias due to post-exposure interventions

**Signalling questions**	**Response options**	**Comments**
4.1 Were there post-exposure interventions that were influenced by prior exposure during the follow-up period?	N	
4.2 **If Y/PY to 4.1**: Is it likely that the analysis corrected for the effect of post-exposure interventions that were influenced by prior exposure?	NA	
Risk of bias (due post-exposure interventions) in the estimated effect of exposure on the outcome	Low risk	
What is the predicted direction of bias due to confounding?	Towards null	
Is the risk of bias (due post-exposure interventions) sufficiently high, in the context of its likely direction and the magnitude of the estimated exposure effect, to threaten conclusions about whether the exposure has an important effect on the outcome?	No	

Y = Yes; PY = Probably yes; PN = Probably no; N = No; NA = Not applicable; NI = No information

Domain 5: Risk of bias due to missing data

**Signalling questions**	**Response options**	**Comments**
5.1 Were complete data on exposure status available for all, or nearly all, participants?	Y	
5.2 Were complete data on the outcome available for all, or nearly all, participants?	Y	
5.3 Were complete data on confounding variables available for all, or nearly all, participants?	Y	
5.4 **If N/PN/NI to 5.1, 5.2 or 5.3**: Is the result based on a complete case analysis?	NA	
5.5 **If Y/PY/NI**: Was exclusion from the analysis because of missing data (in exposure, confounders or the outcome) likely to be related to the true value of the outcome?	N	
5.6 **If N/PN to 5.5**: Were all or most predictors of missingness (in exposure, confounders or the outcome) included in the analysis model?	SY	
5.7 **If N/PN to 5.4**: Was the analysis based on imputing missing values?	NA	
5.8 **If Y/PY to 5.7**: Was imputation performed appropriately?	NA	
5.9 **If N/PN to 5.7**: Was an appropriate alternative method used to correct for bias due to missing data?	Y	
5.10 **If PN/N/NI to 5.1, 5.2 or 5.3**: Is there evidence that the result was not biased by missing data?	NA	
Risk of bias (due to missing data) in the estimated effect of exposure on the outcome	Low risk	
What is the predicted direction of bias due to missing data?	Towards null	
Is the risk of bias (due to missing data) sufficiently high, in the context of its likely direction and the magnitude of the estimated exposure effect, to threaten conclusions about whether the exposure has an important effect on the outcome?	No	

Y = Yes; PY = Probably yes; PN = Probably no; N = No; SY = Strong yes; WY = Weak yes; NA = Not applicable; NI = No information

Domain 6: Risk of bias arising from measurement of the outcome

**Signalling questions**	**Response options**	**Comments**
6.1 Could measurement or ascertainment of the outcome have differed between exposure groups or levels of exposure?	N	
6.2 Were outcome assessors aware of study participants' exposure history?	N	
6.3 **If Y/PY/NI to 6.2**: Could assessment of the outcome have been influenced by knowledge of participants' exposure history?	NA	
Risk of bias (arising from measurement of outcomes) in the estimated effect of exposure on the outcome	Low risk	
What is the predicted direction of bias arising from measurement of outcomes?	Towards null	
Is the risk of bias (arising from measurement of outcomes) sufficiently high, in the context of its likely direction and the magnitude of the estimated exposure effect, to threaten conclusions about whether the exposure has an important effect on the outcome?	No	

Y = Yes; PY = Probably yes; PN = Probably no; N = No; SY = Strong yes; WY = Weak yes; NA = Not applicable; NI = No information

Domain 7: Risk of bias in selection of the reported result

**Signalling questions**	**Response options**	**Comments**
7.1 Was the result reported in accordance with an available, pre-determined analysis plan?	Y	
7.2 **If N/PN/NI to 7.1**: Is the reported effect estimate likely to be selected, based on desirability of the magnitude (or statistical significance) of the estimated effect of exposure on outcome, from multiple *exposure measurements* within the exposure domain?	N	
7.3 Is the reported effect estimate likely to be selected, based on desirability of the magnitude (or statistical significance) of the estimated effect of exposure on outcome, from multiple *outcome measurements* within the outcome domain?	N	

Signalling questions	Response options	Comments
7.4 Is the reported effect estimate likely to be selected, based on desirability of the magnitude (or statistical significance) of the estimated effect of exposure on outcome, from multiple *analyses* of the exposure-outcome relationship?	N	
7.5 Is the reported effect estimate likely to be selected, based on the basis of desirability of the results (e.g. statistical significance), from different *subgroups*?	N	
Risk of bias (due to selection of the reported result) in the estimated effect of exposure on the outcome	Low risk	
What is the predicted direction of bias due to selection of the reported result?	Towards null	
Is the risk of bias (due to selection of the reported result) sufficiently high, in the context of its likely direction and the magnitude of the estimated exposure effect, to threaten conclusions about whether the exposure has an important effect on the outcome?	No	

Y = Yes; PY = Probably yes; PN = Probably no; N = No; NA = Not applicable; NI = No information

Overall risk of bias

	Response options	Comments
Overall risk of bias	Low risk of bias	
What is the predicted direction of bias?	Towards null	
Is the overall risk of bias sufficiently high, in the context of its likely direction and the magnitude of the estimated exposure effect, to threaten conclusions about whether the exposure has an important effect on the outcome?	No	

[38] Onken, M.; Mick, I.; Schaefer, C. Paliperidone and pregnancy-an evaluation of the German Embryotox database. *Arch. Womens Ment. Health* **2018**, *21*(6), 657-662. doi: 10.1007/s00737-018-0828-z. Epub 2018 Mar 22.

Domain 1: Risk of bias due to confounding*, variant (b): **If Y/PY to C7 and Y/PY to C8** (the analysis was based on splitting participants' follow up time according to exposure status and/or magnitude and changes in exposure status and/or magnitude likely to be related to factors that are predictive of the outcome, so both baseline and time-varying confounding need to be addressed)*

Signalling questions	Response options	Comments
1.1 Did the authors use an analysis method that was appropriate to control for time-varying as well as baseline confounding?	Y	
1.2 **If Y/PY to 1.1**: Did the authors control for all the important baseline and time-varying confounding factors for which this was necessary?	Y	
1.3 **If** Y/PY/WN **to 1.2**: Were confounding factors that were controlled for (and for which control was necessary) measured validly and reliably by the variables available in this study?	Y	
1.4 **If N/PN/NI to 1.1**: Did the authors control for time-varying factors or other variables measured after the start of the exposure window being studied?	NA	
1.5 Did the use of negative controls, or other considerations, suggest uncontrolled confounding?	N	
Risk of bias (due to confounding) in the estimated effect of exposure on the outcome	Low risk	
What is the predicted direction of bias due to confounding?	Towards null	
Is the risk of bias (due to confounding) sufficiently high, in the context of its likely direction and the magnitude of the estimated exposure effect, to threaten conclusions about whether the exposure has an important effect on the outcome?	No	

Y = Yes; PY = Probably yes; PN = Probably no; N = No; SY = Strong yes; WY = Weak yes; SN = Strong no; WN = Weak no; NA = Not applicable; NI = No information

Domain 2: Risk of bias arising from measurement of the exposure *Variant (b): **If Y/PY to C5 and Y/PY to C6** (each individual's exposure level was estimated from measurements made at multiple time points)*

Signalling questions	Response options	Comments
**2.1 Does the measured exposure (derived from measurements at multiple time points) well-characterize the exposure metric specified to be of interest in this study? [*This was specified in the answers to D2, D3 and D4*]**	**Y**	
**2.2 Was there error in measurement, or misclassification, of the exposure, at each single time point?**	**N**	
**2.3 If SY/WY to 2.2: Could mismeasurement or misclassification of exposure have been differential (i.e. related to the outcome or risk of the outcome)?**	**NA**	
**2.4 If SY/WY to 2.2 and N/PN/WY to 2.3: Is the nature of the (non-differential) measurement error likely to bias the estimated effect of exposure on outcome?**	**NA**	
**Risk of bias (arising from measurement of exposure) in the estimated effect of exposure on the outcome**	**Low risk**	
**What is the predicted direction of bias arising from measurement of exposure?**	**Towards null**	
**Is the risk of bias (arising from measurement of exposure) sufficiently high, in the context of its likely direction and the magnitude of the estimated exposure effect, to threaten conclusions about whether the exposure has an important effect on the outcome?**	**No**	

Y = Yes; PY = Probably yes; SN = Strong no; WN = Weak no; NA = Not applicable; NI = No information

Domain 3: Risk of bias in selection of participants into the study (or into the analysis)

Signalling questions	Response options	Comments
3.1 Did follow-up begin at (or close to) the start of the exposure window for most participants? [*The exposure window is specified in D3*]	Y	
3.2 **If N/PN to 3.1**: Is the effect of exposure likely to be constant over the period of follow up analysed?	NA	
3.3 Was selection of participants into the study (or into the analysis) based on participant characteristics observed after the start of the exposure window being studied? [*The exposure window is specified in D3*]	N	
3.4 **If Y/PY to 3.3**: Were these characteristics likely to be influenced by exposure or a cause of exposure?	NA	
3.5 **If Y/PY to 3.4**: Were these characteristics likely to be influenced by the outcome or a cause of the outcome?	NA	
3.6 **If N/PN to 3.2 or Y/PY to 3.5**: Is it likely that the analysis corrected for all of the potential selection biases identified in A and B above?	NA	
3.7 **If N/PN to 3.2 or Y/PY to 3.5**: Did sensitivity analyses demonstrate that the likely impact of the potential selection biases identified in A or B above was minimal?	NA	
Risk of bias (due to selection of participants into the study) in the estimated effect of exposure on the outcome	Low risk	
What is the predicted direction of bias due to selection of participants into the study?	Towards null	
Is the risk of bias (due to selection of participants into the study) sufficiently high, in the context of its likely direction and the magnitude of the estimated exposure effect, to threaten conclusions about whether the exposure has an important effect on the outcome?	No	

Y = Yes; PY = Probably yes; PN = Probably no; N = No; SN = Strong no; WN = Weak no; NA = Not applicable; NI = No information

Domain 4: Risk of bias due to post-exposure interventions

Signalling questions	Response options	Comments
4.1 Were there post-exposure interventions that were influenced by prior exposure during the follow-up period?	N	
4.2 **If Y/PY to 4.1**: Is it likely that the analysis corrected for the effect of post-exposure interventions that were influenced by prior exposure?	NA	
Risk of bias (due post-exposure interventions) in the estimated effect of exposure on the outcome	Low risk	
What is the predicted direction of bias due to confounding?	Towards null	
Is the risk of bias (due post-exposure interventions) sufficiently high, in the context of its likely direction and the magnitude of the estimated exposure effect, to threaten conclusions about whether the exposure has an important effect on the outcome?	No	

Y = Yes; PY = Probably yes; PN = Probably no; N = No; NA = Not applicable; NI = No information

Domain 5: Risk of bias due to missing data

Signalling questions	Response options	Comments
5.1 Were complete data on exposure status available for all, or nearly all, participants?	Y	
5.2 Were complete data on the outcome available for all, or nearly all, participants?	Y	
5.3 Were complete data on confounding variables available for all, or nearly all, participants?	Y	
5.4 **If N/PN/NI to 5.1, 5.2 or 5.3**: Is the result based on a complete case analysis?	NA	
5.5 **If Y/PY/NI**: Was exclusion from the analysis because of missing data (in exposure, confounders or the outcome) likely to be related to the true value of the outcome?	N	
5.6 **If N/PN to 5.5**: Were all or most predictors of missingness (in exposure, confounders or the outcome) included in the analysis model?	SY	
5.7 **If N/PN to 5.4**: Was the analysis based on imputing missing values?	NA	
5.8 **If Y/PY to 5.7**: Was imputation performed appropriately?	NA	
5.9 **If N/PN to 5.7**: Was an appropriate alternative method used to correct for bias due to missing data?	Y	
5.10 **If PN/N/NI to 5.1, 5.2 or 5.3**: Is there evidence that the result was not biased by missing data?	NA	
Risk of bias (due to missing data) in the estimated effect of exposure on the outcome	Low risk	
What is the predicted direction of bias due to missing data?	Towards null	
Is the risk of bias (due to missing data) sufficiently high, in the context of its likely direction and the magnitude of the estimated exposure effect, to threaten conclusions about whether the exposure has an important effect on the outcome?	No	

Y = Yes; PY = Probably yes; PN = Probably no; N = No; SY = Strong yes; WY = Weak yes; NA = Not applicable; NI = No information

Domain 6: Risk of bias arising from measurement of the outcome

Signalling questions	Response options	Comments
6.1 Could measurement or ascertainment of the outcome have differed between exposure groups or levels of exposure?	N	
6.2 Were outcome assessors aware of study participants' exposure history?	N	
6.3 **If Y/PY/NI to 6.2**: Could assessment of the outcome have been influenced by knowledge of participants' exposure history?	NA	
Risk of bias (arising from measurement of outcomes) in the estimated effect of exposure on the outcome	Low risk	
What is the predicted direction of bias arising from measurement of outcomes?	Towards null	
Is the risk of bias (arising from measurement of outcomes) sufficiently high, in the context of its likely direction and the magnitude of the estimated exposure effect, to threaten conclusions about whether the exposure has an important effect on the outcome?	No	

Y = Yes; PY = Probably yes; PN = Probably no; N = No; SY = Strong yes; WY = Weak yes; NA = Not applicable; NI = No information

Domain 7: Risk of bias in selection of the reported result

Signalling questions	Response options	Comments
7.1 Was the result reported in accordance with an available, pre-determined analysis plan?	Y	
7.2 **If N/PN/NI to 7.1**: Is the reported effect estimate likely to be selected, based on desirability of the magnitude (or statistical significance) of the estimated effect of exposure on outcome, from multiple *exposure measurements* within the exposure domain?	N	
7.3 Is the reported effect estimate likely to be selected, based on desirability of the magnitude (or statistical significance) of the estimated effect of exposure on outcome, from multiple *outcome measurements* within the outcome domain?	N	
7.4 Is the reported effect estimate likely to be selected, based on desirability of the magnitude (or statistical significance) of the estimated effect of exposure on outcome, from multiple *analyses* of the exposure-outcome relationship?	N	
7.5 Is the reported effect estimate likely to be selected, based on the basis of desirability of the results (e.g. statistical significance), from different *subgroups*?	N	
Risk of bias (due to selection of the reported result) in the estimated effect of exposure on the outcome	Low risk	
What is the predicted direction of bias due to selection of the reported result?	Towards null	
Is the risk of bias (due to selection of the reported result) sufficiently high, in the context of its likely direction and the magnitude of the estimated exposure effect, to threaten conclusions about whether the exposure has an important effect on the outcome?	No	

Y = Yes; PY = Probably yes; PN = Probably no; N = No; NA = Not applicable; NI = No information

Overall risk of bias

	Response options	Comments
Overall risk of bias	Low risk of bias	
What is the predicted direction of bias?	Towards null	
Is the overall risk of bias sufficiently high, in the context of its likely direction and the magnitude of the estimated exposure effect, to threaten conclusions about whether the exposure has an important effect on the outcome?	No	

[39] Galbally, M.; Frayne, J.; Watson, S.J.; Snellen, M. Aripiprazole and pregnancy: A retrospective, multicentre study. *J. Affect. Disord.* **2018**, *238*, 593-596. doi: 10.1016/j.jad.2018.06.004.

Domain 1: Risk of bias due to confounding, *variant (b): **If Y/PY to C7 and Y/PY to C8** (the analysis was based on splitting participants' follow up time according to exposure status and/or magnitude and changes in exposure status and/or magnitude likely to be related to factors that are predictive of the outcome, so both baseline and time-varying confounding need to be addressed)*

Signalling questions	Response options	Comments
1.1 Did the authors use an analysis method that was appropriate to control for time-varying as well as baseline confounding?	Y	
1.2 **If Y/PY to 1.1**: Did the authors control for all the important baseline and time-varying confounding factors for which this was necessary?	Y	
1.3 **If Y/PY/WN to 1.2**: Were confounding factors that were controlled for (and for which control was necessary) measured validly and reliably by the variables available in this study?	Y	
1.4 **If N/PN/NI to 1.1**: Did the authors control for time-varying factors or other variables measured after the start of the exposure window being studied?	NA	
1.5 Did the use of negative controls, or other considerations, suggest uncontrolled confounding?	N	
Risk of bias (due to confounding) in the estimated effect of exposure on the outcome	Low risk	
What is the predicted direction of bias due to confounding?	Towards null	
Is the risk of bias (due to confounding) sufficiently high, in the context of its likely direction and the magnitude of the estimated exposure effect, to threaten conclusions about whether the exposure has an important effect on the outcome?	No	

Y = Yes; PY = Probably yes; PN = Probably no; N = No; SY = Strong yes; WY = Weak yes; SN = Strong no; WN = Weak no; NA = Not applicable; NI = No information

Domain 2: Risk of bias arising from measurement of the exposure *Variant (b): **If Y/PY to C5 and Y/PY to C6** (each individual's exposure level was estimated from measurements made at multiple time points)*

Signalling questions	Response options	Comments
**2.1 Does the measured exposure (derived from measurements at multiple time points) well-characterize the exposure metric specified to be of interest in this study? [*This was specified in the answers to D2, D3 and D4*]**	**Y**	
**2.2 Was there error in measurement, or misclassification, of the exposure, at each single time point?**	**N**	
**2.3 If SY/WY to 2.2: Could mismeasurement or misclassification of exposure have been differential (i.e. related to the outcome or risk of the outcome)?**	**NA**	
**2.4 If SY/WY to 2.2 and N/PN/WY to 2.3: Is the nature of the (non-differential) measurement error likely to bias the estimated effect of exposure on outcome?**	**NA**	
**Risk of bias (arising from measurement of exposure) in the estimated effect of exposure on the outcome**	**Low risk**	
**What is the predicted direction of bias arising from measurement of exposure?**	**Towards null**	
**Is the risk of bias (arising from measurement of exposure) sufficiently high, in the context of its**	**No**	

Signalling questions	Response options	Comments
**likely direction and the magnitude of the estimated exposure effect, to threaten conclusions about whether the exposure has an important effect on the outcome?**		

Y = Yes; PY = Probably yes; SN = Strong no; WN = Weak no; NA = Not applicable; NI = No information

Domain 3: Risk of bias in selection of participants into the study (or into the analysis)

Signalling questions	Response options	Comments
3.1 Did follow-up begin at (or close to) the start of the exposure window for most participants? [*The exposure window is specified in D3*]	Y	
3.2 **If N/PN to 3.1**: Is the effect of exposure likely to be constant over the period of follow up analysed?	NA	
3.3 Was selection of participants into the study (or into the analysis) based on participant characteristics observed after the start of the exposure window being studied? [*The exposure window is specified in D3*]	N	
3.4 **If Y/PY to 3.3**: Were these characteristics likely to be influenced by exposure or a cause of exposure?	NA	
3.5 **If Y/PY to 3.4**: Were these characteristics likely to be influenced by the outcome or a cause of the outcome?	NA	
3.6 **If N/PN to 3.2 or Y/PY to 3.5**: Is it likely that the analysis corrected for all of the potential selection biases identified in A and B above?	NA	
3.7 **If N/PN to 3.2 or Y/PY to 3.5**: Did sensitivity analyses demonstrate that the likely impact of the potential selection biases identified in A or B above was minimal?	NA	
Risk of bias (due to selection of participants into the study) in the estimated effect of exposure on the outcome	Low risk	
What is the predicted direction of bias due to selection of participants into the study?	Towards null	
Is the risk of bias (due to selection of participants into the study) sufficiently high, in the context of its likely direction and the magnitude of the estimated exposure effect, to threaten conclusions about whether the exposure has an important effect on the outcome?	No	

Y = Yes; PY = Probably yes; PN = Probably no; N = No; SN = Strong no; WN = Weak no; NA = Not applicable; NI = No information

Domain 4: Risk of bias due to post-exposure interventions

Signalling questions	Response options	Comments
4.1 Were there post-exposure interventions that were influenced by prior exposure during the follow-up period?	N	
4.2 **If Y/PY to 4.1**: Is it likely that the analysis corrected for the effect of post-exposure interventions that were influenced by prior exposure?	NA	
Risk of bias (due post-exposure interventions) in the estimated effect of exposure on the outcome	Low risk	
What is the predicted direction of bias due to confounding?	Towards null	
Is the risk of bias (due post-exposure interventions) sufficiently high, in the context of its likely direction and the magnitude of the estimated exposure effect, to threaten conclusions about whether the exposure has an important effect on the outcome?	No	

Y = Yes; PY = Probably yes; PN = Probably no; N = No; NA = Not applicable; NI = No information

Domain 5: Risk of bias due to missing data

Signalling questions	Response options	Comments
5.1 Were complete data on exposure status available for all, or nearly all, participants?	Y	
5.2 Were complete data on the outcome available for all, or nearly all, participants?	PY	
5.3 Were complete data on confounding variables available for all, or nearly all, participants?	Y	
5.4 **If N/PN/NI to 5.1, 5.2 or 5.3**: Is the result based on a complete case analysis?	NA	
5.5 **If Y/PY/NI**: Was exclusion from the analysis because of missing data (in exposure, confounders or the outcome) likely to be related to the true value of the outcome?	N	
5.6 **If N/PN to 5.5**: Were all or most predictors of missingness (in exposure, confounders or the outcome) included in the analysis model?	SY	
5.7 **If N/PN to 5.4**: Was the analysis based on imputing missing values?	NA	
5.8 **If Y/PY to 5.7**: Was imputation performed appropriately?	NA	
5.9 **If N/PN to 5.7**: Was an appropriate alternative method used to correct for bias due to missing data?	Y	
5.10 **If PN/N/NI to 5.1, 5.2 or 5.3**: Is there evidence that the result was not biased by missing data?	NA	
Risk of bias (due to missing data) in the estimated effect of exposure on the outcome	Low risk	
What is the predicted direction of bias due to missing data?	Towards null	
Is the risk of bias (due to missing data) sufficiently high, in the context of its likely direction and the magnitude of the estimated exposure effect, to threaten conclusions about whether the exposure has an important effect on the outcome?	No	

Y = Yes; PY = Probably yes; PN = Probably no; N = No; SY = Strong yes; WY = Weak yes; NA = Not applicable; NI = No information

Domain 6: Risk of bias arising from measurement of the outcome

Signalling questions	Response options	Comments
6.1 Could measurement or ascertainment of the outcome have differed between exposure groups or levels of exposure?	N	
6.2 Were outcome assessors aware of study participants' exposure history?	N	

Signalling questions	Response options	Comments
6.3 **If Y/PY/NI to 6.2**: Could assessment of the outcome have been influenced by knowledge of participants' exposure history?	NA	
Risk of bias (arising from measurement of outcomes) in the estimated effect of exposure on the outcome	Low risk	
What is the predicted direction of bias arising from measurement of outcomes?	Towards null	
Is the risk of bias (arising from measurement of outcomes) sufficiently high, in the context of its likely direction and the magnitude of the estimated exposure effect, to threaten conclusions about whether the exposure has an important effect on the outcome?	No	

Y = Yes; PY = Probably yes; PN = Probably no; N = No; SY = Strong yes; WY = Weak yes; NA = Not applicable; NI = No information

Domain 7: Risk of bias in selection of the reported result

Signalling questions	Response options	Comments
7.1 Was the result reported in accordance with an available, pre-determined analysis plan?	Y	
7.2 **If N/PN/NI to 7.1**: Is the reported effect estimate likely to be selected, based on desirability of the magnitude (or statistical significance) of the estimated effect of exposure on outcome, from multiple *exposure measurements* within the exposure domain?	N	
7.3 Is the reported effect estimate likely to be selected, based on desirability of the magnitude (or statistical significance) of the estimated effect of exposure on outcome, from multiple *outcome measurements* within the outcome domain?	PN	
7.4 Is the reported effect estimate likely to be selected, based on desirability of the magnitude (or statistical significance) of the estimated effect of exposure on outcome, from multiple *analyses* of the exposure-outcome relationship?	N	
7.5 Is the reported effect estimate likely to be selected, based on the basis of desirability of the results (e.g. statistical significance), from different *subgroups*?	N	
Risk of bias (due to selection of the reported result) in the estimated effect of exposure on the outcome	Low risk	
What is the predicted direction of bias due to selection of the reported result?	Towards null	
Is the risk of bias (due to selection of the reported result) sufficiently high, in the context of its likely direction and the magnitude of the estimated exposure effect, to threaten conclusions about whether the exposure has an important effect on the outcome?	No	

Y = Yes; PY = Probably yes; PN = Probably no; N = No; NA = Not applicable; NI = No information

Overall risk of bias

	Response options	Comments
Overall risk of bias	Low risk of bias	
What is the predicted direction of bias?	Towards null	
Is the overall risk of bias sufficiently high, in the context of its likely direction and the magnitude of the estimated exposure effect, to threaten conclusions about whether the exposure has an important effect on the outcome?	No	

[40] Cohen, L.S.; Góez-Mogollón, L.; Sosinsky, A.Z.; Savella, G.M.; Viguera, A.C.; Chitayat, D.; Hernández-Díaz, S.; Freeman, M.P. Risk of major malformations in infants following first-trimester exposure to quetiapine. *Am. J. Psychiatry* **2018**, *175*(12), 1225-1231. doi:10.1176/appi.ajp.2018.18010098.

Domain 1: Risk of bias due to confounding*, variant (b): **If Y/PY to C7 and Y/PY to C8** (the analysis was based on splitting participants' follow up time according to exposure status and/or magnitude and changes in exposure status and/or magnitude likely to be related to factors that are predictive of the outcome, so both baseline and time-varying confounding need to be addressed)*

Signalling questions	Response options	Comments
1.1 Did the authors use an analysis method that was appropriate to control for time-varying as well as baseline confounding?	Y	
1.2 **If Y/PY to 1.1**: Did the authors control for all the important baseline and time-varying confounding factors for which this was necessary?	Y	
1.3 **If Y/PY/WN to 1.2**: Were confounding factors that were controlled for (and for which control was necessary) measured validly and reliably by the variables available in this study?	Y	
1.4 **If N/PN/NI to 1.1**: Did the authors control for time-varying factors or other variables measured after the start of the exposure window being studied?	NA	
1.5 Did the use of negative controls, or other considerations, suggest uncontrolled confounding?	N	
Risk of bias (due to confounding) in the estimated effect of exposure on the outcome	Low risk	
What is the predicted direction of bias due to confounding?	Towards null	
Is the risk of bias (due to confounding) sufficiently high, in the context of its likely direction and the magnitude of the estimated exposure effect, to threaten conclusions about whether the exposure has an important effect on the outcome?	No	

Y = Yes; PY = Probably yes; PN = Probably no; N = No; SY = Strong yes; WY = Weak yes; SN = Strong no; WN = Weak no; NA = Not applicable; NI = No information

Domain 2: Risk of bias arising from measurement of the exposure *Variant (b): **If Y/PY to C5 and Y/PY to C6** (each individual's exposure level was estimated from measurements made at multiple time points)*

Signalling questions	Response options	Comments

**Signalling questions**	**Response options**	**Comments**
**2.1 Does the measured exposure (derived from measurements at multiple time points) well-characterize the exposure metric specified to be of interest in this study? [*This was specified in the answers to D2, D3 and D4*]**	**Y**	
**2.2 Was there error in measurement, or misclassification, of the exposure, at each single time point?**	**N**	
**2.3 If SY/WY to 2.2: Could mismeasurement or misclassification of exposure have been differential (i.e. related to the outcome or risk of the outcome)?**	**NA**	
**2.4 If SY/WY to 2.2 and N/PN/WY to 2.3: Is the nature of the (non-differential) measurement error likely to bias the estimated effect of exposure on outcome?**	**NA**	
**Risk of bias (arising from measurement of exposure) in the estimated effect of exposure on the outcome**	**Low risk**	
**What is the predicted direction of bias arising from measurement of exposure?**	**Towards null**	
**Is the risk of bias (arising from measurement of exposure) sufficiently high, in the context of its likely direction and the magnitude of the estimated exposure effect, to threaten conclusions about whether the exposure has an important effect on the outcome?**	**No**	

Y = Yes; PY = Probably yes; SN = Strong no; WN = Weak no; NA = Not applicable; NI = No information

Domain 3: Risk of bias in selection of participants into the study (or into the analysis)

**Signalling questions**	**Response options**	**Comments**
3.1 Did follow-up begin at (or close to) the start of the exposure window for most participants? [*The exposure window is specified in D3*]	Y	
3.2 **If N/PN to 3.1**: Is the effect of exposure likely to be constant over the period of follow up analysed?	NA	
3.3 Was selection of participants into the study (or into the analysis) based on participant characteristics observed after the start of the exposure window being studied? [*The exposure window is specified in D3*]	N	
3.4 **If Y/PY to 3.3**: Were these characteristics likely to be influenced by exposure or a cause of exposure?	NA	
3.5 **If Y/PY to 3.4**: Were these characteristics likely to be influenced by the outcome or a cause of the outcome?	NA	
3.6 **If N/PN to 3.2 or Y/PY to 3.5**: Is it likely that the analysis corrected for all of the potential selection biases identified in A and B above?	NA	
3.7 **If N/PN to 3.2 or Y/PY to 3.5**: Did sensitivity analyses demonstrate that the likely impact of the potential selection biases identified in A or B above was minimal?	NA	
Risk of bias (due to selection of participants into the study) in the estimated effect of exposure on the outcome	Low risk	
What is the predicted direction of bias due to selection of participants into the study?	Towards null	
Is the risk of bias (due to selection of participants into the study) sufficiently high, in the context of its likely direction and the magnitude of the estimated exposure effect, to threaten conclusions about whether the exposure has an important effect on the outcome?	No	

Y = Yes; PY = Probably yes; PN = Probably no; N = No; SN = Strong no; WN = Weak no; NA = Not applicable; NI = No information

Domain 4: Risk of bias due to post-exposure interventions

**Signalling questions**	**Response options**	**Comments**
4.1 Were there post-exposure interventions that were influenced by prior exposure during the follow-up period?	N	
4.2 **If Y/PY to 4.1**: Is it likely that the analysis corrected for the effect of post-exposure interventions that were influenced by prior exposure?	NA	
Risk of bias (due post-exposure interventions) in the estimated effect of exposure on the outcome	Low risk	
What is the predicted direction of bias due to confounding?	Towards null	
Is the risk of bias (due post-exposure interventions) sufficiently high, in the context of its likely direction and the magnitude of the estimated exposure effect, to threaten conclusions about whether the exposure has an important effect on the outcome?	No	

Y = Yes; PY = Probably yes; PN = Probably no; N = No; NA = Not applicable; NI = No information

Domain 5: Risk of bias due to missing data

**Signalling questions**	**Response options**	**Comments**
5.1 Were complete data on exposure status available for all, or nearly all, participants?	Y	
5.2 Were complete data on the outcome available for all, or nearly all, participants?	Y	
5.3 Were complete data on confounding variables available for all, or nearly all, participants?	Y	
5.4 **If N/PN/NI to 5.1, 5.2 or 5.3**: Is the result based on a complete case analysis?	NA	
5.5 **If Y/PY/NI**: Was exclusion from the analysis because of missing data (in exposure, confounders or the outcome) likely to be related to the true value of the outcome?	N	
5.6 **If N/PN to 5.5**: Were all or most predictors of missingness (in exposure, confounders or the outcome) included in the analysis model?	SY	
5.7 **If N/PN to 5.4**: Was the analysis based on imputing missing values?	NA	
5.8 **If Y/PY to 5.7**: Was imputation performed appropriately?	NA	
5.9 **If N/PN to 5.7**: Was an appropriate alternative method used to correct for bias due to missing data?	Y	
5.10 **If PN/N/NI to 5.1, 5.2 or 5.3**: Is there evidence that the result was not biased by missing data?	NA	
Risk of bias (due to missing data) in the estimated effect of exposure on the outcome	Low risk	

Signalling questions	Response options	Comments
What is the predicted direction of bias due to missing data?	Towards null	
Is the risk of bias (due to missing data) sufficiently high, in the context of its likely direction and the magnitude of the estimated exposure effect, to threaten conclusions about whether the exposure has an important effect on the outcome?	No	

Y = Yes; PY = Probably yes; PN = Probably no; N = No; SY = Strong yes; WY = Weak yes; NA = Not applicable; NI = No information

Domain 6: Risk of bias arising from measurement of the outcome

Signalling questions	Response options	Comments
6.1 Could measurement or ascertainment of the outcome have differed between exposure groups or levels of exposure?	N	
6.2 Were outcome assessors aware of study participants' exposure history?	N	
6.3 **If Y/PY/NI to 6.2**: Could assessment of the outcome have been influenced by knowledge of participants' exposure history?	NA	
Risk of bias (arising from measurement of outcomes) in the estimated effect of exposure on the outcome	Low risk	
What is the predicted direction of bias arising from measurement of outcomes?	Towards null	
Is the risk of bias (arising from measurement of outcomes) sufficiently high, in the context of its likely direction and the magnitude of the estimated exposure effect, to threaten conclusions about whether the exposure has an important effect on the outcome?	No	

Y = Yes; PY = Probably yes; PN = Probably no; N = No; SY = Strong yes; WY = Weak yes; NA = Not applicable; NI = No information

Domain 7: Risk of bias in selection of the reported result

Signalling questions	Response options	Comments
7.1 Was the result reported in accordance with an available, pre-determined analysis plan?	Y	
7.2 **If N/PN/NI to 7.1**: Is the reported effect estimate likely to be selected, based on desirability of the magnitude (or statistical significance) of the estimated effect of exposure on outcome, from multiple *exposure measurements* within the exposure domain?	N	
7.3 Is the reported effect estimate likely to be selected, based on desirability of the magnitude (or statistical significance) of the estimated effect of exposure on outcome, from multiple *outcome measurements* within the outcome domain?	N	
7.4 Is the reported effect estimate likely to be selected, based on desirability of the magnitude (or statistical significance) of the estimated effect of exposure on outcome, from multiple *analyses* of the exposure-outcome relationship?	N	
7.5 Is the reported effect estimate likely to be selected, based on the basis of desirability of the results (e.g. statistical significance), from different *subgroups*?	N	
Risk of bias (due to selection of the reported result) in the estimated effect of exposure on the outcome	Low risk	
What is the predicted direction of bias due to selection of the reported result?	Towards null	
Is the risk of bias (due to selection of the reported result) sufficiently high, in the context of its likely direction and the magnitude of the estimated exposure effect, to threaten conclusions about whether the exposure has an important effect on the outcome?	No	

Y = Yes; PY = Probably yes; PN = Probably no; N = No; NA = Not applicable; NI = No information

Overall risk of bias

	Response options	Comments
Overall risk of bias	Low risk of bias	
What is the predicted direction of bias?	Towards null	
Is the overall risk of bias sufficiently high, in the context of its likely direction and the magnitude of the estimated exposure effect, to threaten conclusions about whether the exposure has an important effect on the outcome?	No	

[41] Anderson, K.N.; Ailes, E.C.; Lind, J.N.; Broussard, C.S.; Bitsko, R.H.; Friedman, J.M.; Bobo, W.V.; Reefhuis, J.; Tinker, S.C; National Birth Defects Prevention Study. Atypical antipsychotic use during pregnancy and birth defect risk: National Birth Defects Prevention Study, 1997-2011. *Schizophr. Res.* **2020**, *215*, 81-88. doi: 10.1016/j.schres.2019.11.019.

Domain 1: Risk of bias due to confounding*, variant (b):* ***If Y/PY to C7 and Y/PY to C8*** *(the analysis was based on splitting participants' follow up time according to exposure status and/or magnitude and changes in exposure status and/or magnitude likely to be related to factors that are predictive of the outcome, so both baseline and time-varying confounding need to be addressed)*

Signalling questions	Response options	Comments
1.1 Did the authors use an analysis method that was appropriate to control for time-varying as well as baseline confounding?	Y	
1.2 **If Y/PY to 1.1**: Did the authors control for all the important baseline and time-varying confounding factors for which this was necessary?	Y	
1.3 **If Y/PY/WN to 1.2**: Were confounding factors that were controlled for (and for which control was necessary) measured validly and reliably by the variables available in this study?	PY	
1.4 **If N/PN/NI to 1.1**: Did the authors control for time-varying factors or other variables measured after the start of the exposure window being studied?	NA	
1.5 Did the use of negative controls, or other considerations, suggest uncontrolled confounding?	N	

Signalling questions	Response options	Comments
Risk of bias (due to confounding) in the estimated effect of exposure on the outcome	Low risk	
What is the predicted direction of bias due to confounding?	Towards null	
Is the risk of bias (due to confounding) sufficiently high, in the context of its likely direction and the magnitude of the estimated exposure effect, to threaten conclusions about whether the exposure has an important effect on the outcome?	No	

Y = Yes; PY = Probably yes; PN = Probably no; N = No; SY = Strong yes; WY = Weak yes; SN = Strong no; WN = Weak no; NA = Not applicable; NI = No information

Domain 2: Risk of bias arising from measurement of the exposure *Variant (b): If Y/PY to C5 and Y/PY to C6 (each individual's exposure level was estimated from measurements made at multiple time points)*

Signalling questions	Response options	Comments
**2.1 Does the measured exposure (derived from measurements at multiple time points) well-characterize the exposure metric specified to be of interest in this study? [*This was specified in the answers to D2, D3 and D4*]**	Y	
**2.2 Was there error in measurement, or misclassification, of the exposure, at each single time point?**	N	
**2.3 If SY/WY to 2.2: Could mismeasurement or misclassification of exposure have been differential (i.e. related to the outcome or risk of the outcome)?**	**NA**	
**2.4 If SY/WY to 2.2 and N/PN/WY to 2.3: Is the nature of the (non-differential) measurement error likely to bias the estimated effect of exposure on outcome?**	**NA**	
**Risk of bias (arising from measurement of exposure) in the estimated effect of exposure on the outcome**	**Low risk**	
**What is the predicted direction of bias arising from measurement of exposure?**	**Towards null**	
**Is the risk of bias (arising from measurement of exposure) sufficiently high, in the context of its likely direction and the magnitude of the estimated exposure effect, to threaten conclusions about whether the exposure has an important effect on the outcome?**	**No**	

Y = Yes; PY = Probably yes; SN = Strong no; WN = Weak no; NA = Not applicable; NI = No information

Domain 3: Risk of bias in selection of participants into the study (or into the analysis)

Signalling questions	Response options	Comments
3.1 Did follow-up begin at (or close to) the start of the exposure window for most participants? [*The exposure window is specified in D3*]	Y	
3.2 **If N/PN to 3.1**: Is the effect of exposure likely to be constant over the period of follow up analysed?	NA	
3.3 Was selection of participants into the study (or into the analysis) based on participant characteristics observed after the start of the exposure window being studied? [*The exposure window is specified in D3*]	N	
3.4 **If Y/PY to 3.3**: Were these characteristics likely to be influenced by exposure or a cause of exposure?	NA	
3.5 **If Y/PY to 3.4**: Were these characteristics likely to be influenced by the outcome or a cause of the outcome?	NA	
3.6 **If N/PN to 3.2 or Y/PY to 3.5**: Is it likely that the analysis corrected for all of the potential selection biases identified in A and B above?	NA	
3.7 **If N/PN to 3.2 or Y/PY to 3.5**: Did sensitivity analyses demonstrate that the likely impact of the potential selection biases identified in A or B above was minimal?	NA	
Risk of bias (due to selection of participants into the study) in the estimated effect of exposure on the outcome	Low risk	
What is the predicted direction of bias due to selection of participants into the study?	Towards null	
Is the risk of bias (due to selection of participants into the study) sufficiently high, in the context of its likely direction and the magnitude of the estimated exposure effect, to threaten conclusions about whether the exposure has an important effect on the outcome?	No	

Y = Yes; PY = Probably yes; PN = Probably no; N = No; SN = Strong no; WN = Weak no; NA = Not applicable; NI = No information

Domain 4: Risk of bias due to post-exposure interventions

Signalling questions	Response options	Comments
4.1 Were there post-exposure interventions that were influenced by prior exposure during the follow-up period?	N	
4.2 **If Y/PY to 4.1**: Is it likely that the analysis corrected for the effect of post-exposure interventions that were influenced by prior exposure?	NA	
Risk of bias (due post-exposure interventions) in the estimated effect of exposure on the outcome	Low risk	
What is the predicted direction of bias due to confounding?	Towards null	
Is the risk of bias (due post-exposure interventions) sufficiently high, in the context of its likely direction and the magnitude of the estimated exposure effect, to threaten conclusions about whether the exposure has an important effect on the outcome?	No	

Y = Yes; PY = Probably yes; PN = Probably no; N = No; NA = Not applicable; NI = No information

Domain 5: Risk of bias due to missing data

Signalling questions	Response options	Comments
5.1 Were complete data on exposure status available for all, or nearly all, participants?	Y	
5.2 Were complete data on the outcome available for all, or nearly all, participants?	Y	
5.3 Were complete data on confounding variables available for all, or nearly all, participants?	PY	
5.4 **If N/PN/NI to 5.1, 5.2 or 5.3**: Is the result based on a complete case analysis?	NA	
5.5 **If Y/PY/NI**: Was exclusion from the analysis because of missing data (in exposure, confounders or the outcome) likely to be related to the true value of the outcome?	N	
5.6 **If N/PN to 5.5**: Were all or most predictors of missingness (in exposure, confounders or the outcome) included in the analysis model?	SY	
5.7 **If N/PN to 5.4**: Was the analysis based on imputing missing values?	NA	
5.8 **If Y/PY to 5.7**: Was imputation performed appropriately?	NA	
5.9 **If N/PN to 5.7**: Was an appropriate alternative method used to correct for bias due to missing data?	Y	
5.10 **If PN/N/NI to 5.1, 5.2 or 5.3**: Is there evidence that the result was not biased by missing data?	NA	
Risk of bias (due to missing data) in the estimated effect of exposure on the outcome	Low risk	
What is the predicted direction of bias due to missing data?	Towards null	
Is the risk of bias (due to missing data) sufficiently high, in the context of its likely direction and the magnitude of the estimated exposure effect, to threaten conclusions about whether the exposure has an important effect on the outcome?	No	

Y = Yes; PY = Probably yes; PN = Probably no; N = No; SY = Strong yes; WY = Weak yes; NA = Not applicable; NI = No information

Domain 6: Risk of bias arising from measurement of the outcome

Signalling questions	Response options	Comments
6.1 Could measurement or ascertainment of the outcome have differed between exposure groups or levels of exposure?	N	
6.2 Were outcome assessors aware of study participants' exposure history?	N	
6.3 **If Y/PY/NI to 6.2**: Could assessment of the outcome have been influenced by knowledge of participants' exposure history?	NA	
Risk of bias (arising from measurement of outcomes) in the estimated effect of exposure on the outcome	Low risk	
What is the predicted direction of bias arising from measurement of outcomes?	Towards null	
Is the risk of bias (arising from measurement of outcomes) sufficiently high, in the context of its likely direction and the magnitude of the estimated exposure effect, to threaten conclusions about whether the exposure has an important effect on the outcome?	No	

Y = Yes; PY = Probably yes; PN = Probably no; N = No; SY = Strong yes; WY = Weak yes; NA = Not applicable; NI = No information

Domain 7: Risk of bias in selection of the reported result

Signalling questions	Response options	Comments
7.1 Was the result reported in accordance with an available, pre-determined analysis plan?	Y	
7.2 **If N/PN/NI to 7.1**: Is the reported effect estimate likely to be selected, based on desirability of the magnitude (or statistical significance) of the estimated effect of exposure on outcome, from multiple *exposure measurements* within the exposure domain?	N	
7.3 Is the reported effect estimate likely to be selected, based on desirability of the magnitude (or statistical significance) of the estimated effect of exposure on outcome, from multiple *outcome measurements* within the outcome domain?	N	
7.4 Is the reported effect estimate likely to be selected, based on desirability of the magnitude (or statistical significance) of the estimated effect of exposure on outcome, from multiple *analyses* of the exposure-outcome relationship?	N	
7.5 Is the reported effect estimate likely to be selected, based on the basis of desirability of the results (e.g. statistical significance), from different *subgroups*?	N	
Risk of bias (due to selection of the reported result) in the estimated effect of exposure on the outcome	Low risk	
What is the predicted direction of bias due to selection of the reported result?	Towards null	
Is the risk of bias (due to selection of the reported result) sufficiently high, in the context of its likely direction and the magnitude of the estimated exposure effect, to threaten conclusions about whether the exposure has an important effect on the outcome?	No	

Y = Yes; PY = Probably yes; PN = Probably no; N = No; NA = Not applicable; NI = No information

Overall risk of bias

	Response options	Comments
Overall risk of bias	Low risk of bias	
What is the predicted direction of bias?	Towards null	
Is the overall risk of bias sufficiently high, in the context of its likely direction and the magnitude of the estimated	No	

exposure effect, to threaten conclusions about whether the exposure has an important effect on the outcome?		

[42] Ellfolk, M.; Leinonen, M.K.; Gissler, M.; Lahesmaa-Korpinen, A.M.; Saastamoinen, L.; Nurminen, M.L.; Malm, H. Second-generation antipsychotics and pregnancy complications. *Eur. J. Clin. Pharmacol.* **2020**, *76*(1), 107-115. doi: 10.1007/s00228-019-02769-z. Epub 2019 Nov 3.

Domain 1: Risk of bias due to confounding*, variant (b): **If Y/PY to C7 and Y/PY to C8** (the analysis was based on splitting participants' follow up time according to exposure status and/or magnitude and changes in exposure status and/or magnitude likely to be related to factors that are predictive of the outcome, so both baseline and time-varying confounding need to be addressed)*

Signalling questions	Response options	Comments
1.1 Did the authors use an analysis method that was appropriate to control for time-varying as well as baseline confounding?	Y	
1.2 **If Y/PY to 1.1**: Did the authors control for all the important baseline and time-varying confounding factors for which this was necessary?	Y	
1.3 **If Y/PY/WN to 1.2**: Were confounding factors that were controlled for (and for which control was necessary) measured validly and reliably by the variables available in this study?	Y	
1.4 **If N/PN/NI to 1.1**: Did the authors control for time-varying factors or other variables measured after the start of the exposure window being studied?	NA	
1.5 Did the use of negative controls, or other considerations, suggest uncontrolled confounding?	N	
Risk of bias (due to confounding) in the estimated effect of exposure on the outcome	Low risk	
What is the predicted direction of bias due to confounding?	Towards null	
Is the risk of bias (due to confounding) sufficiently high, in the context of its likely direction and the magnitude of the estimated exposure effect, to threaten conclusions about whether the exposure has an important effect on the outcome?	No	

Y = Yes; PY = Probably yes; PN = Probably no; N = No; SY = Strong yes; WY = Weak yes; SN = Strong no; WN = Weak no; NA = Not applicable; NI = No information

Domain 2: Risk of bias arising from measurement of the exposure *Variant (b): **If Y/PY to C5 and Y/PY to C6** (each individual's exposure level was estimated from measurements made at multiple time points)*

Signalling questions	Response options	Comments
**2.1 Does the measured exposure (derived from measurements at multiple time points) well-characterize the exposure metric specified to be of interest in this study? [*This was specified in the answers to D2, D3 and D4*]**	**Y**	
**2.2 Was there error in measurement, or misclassification, of the exposure, at each single time point?**	**N**	
**2.3 If SY/WY to 2.2: Could mismeasurement or misclassification of exposure have been differential (i.e. related to the outcome or risk of the outcome)?**	**NA**	
**2.4 If SY/WY to 2.2 and N/PN/WY to 2.3: Is the nature of the (non-differential) measurement error likely to bias the estimated effect of exposure on outcome?**	**NA**	
**Risk of bias (arising from measurement of exposure) in the estimated effect of exposure on the outcome**	**Low risk**	
**What is the predicted direction of bias arising from measurement of exposure?**	**Towards null**	
**Is the risk of bias (arising from measurement of exposure) sufficiently high, in the context of its likely direction and the magnitude of the estimated exposure effect, to threaten conclusions about whether the exposure has an important effect on the outcome?**	**No**	

Y = Yes; PY = Probably yes; SN = Strong no; WN = Weak no; NA = Not applicable; NI = No information

Domain 3: Risk of bias in selection of participants into the study (or into the analysis)

Signalling questions	Response options	Comments
3.1 Did follow-up begin at (or close to) the start of the exposure window for most participants? [*The exposure window is specified in D3*]	Y	
3.2 **If N/PN to 3.1**: Is the effect of exposure likely to be constant over the period of follow up analysed?	NA	
3.3 Was selection of participants into the study (or into the analysis) based on participant characteristics observed after the start of the exposure window being studied? [*The exposure window is specified in D3*]	N	
3.4 **If Y/PY to 3.3**: Were these characteristics likely to be influenced by exposure or a cause of exposure?	NA	
3.5 **If Y/PY to 3.4**: Were these characteristics likely to be influenced by the outcome or a cause of the outcome?	NA	
3.6 **If N/PN to 3.2 or Y/PY to 3.5**: Is it likely that the analysis corrected for all of the potential selection biases identified in A and B above?	NA	
3.7 **If N/PN to 3.2 or Y/PY to 3.5**: Did sensitivity analyses demonstrate that the likely impact of the potential selection biases identified in A or B above was minimal?	NA	
Risk of bias (due to selection of participants into the study) in the estimated effect of exposure on the outcome	Low risk	
What is the predicted direction of bias due to selection of participants into the study?	Towards null	
Is the risk of bias (due to selection of participants into the study) sufficiently high, in the context of its likely direction and the magnitude of the estimated exposure effect, to threaten conclusions about whether the exposure has an important effect on the outcome?	No	

Y = Yes; PY = Probably yes; PN = Probably no; N = No; SN = Strong no; WN = Weak no; NA = Not applicable; NI = No information

Domain 4: Risk of bias due to post-exposure interventions

Signalling questions	Response options	Comments
4.1 Were there post-exposure interventions that were influenced by prior exposure during the follow-up period?	N	
4.2 **If Y/PY to 4.1**: Is it likely that the analysis corrected for the effect of post-exposure interventions that were influenced by prior exposure?	NA	
Risk of bias (due post-exposure interventions) in the estimated effect of exposure on the outcome	Low risk	
What is the predicted direction of bias due to confounding?	Towards null	
Is the risk of bias (due post-exposure interventions) sufficiently high, in the context of its likely direction and the magnitude of the estimated exposure effect, to threaten conclusions about whether the exposure has an important effect on the outcome?	No	

Y = Yes; PY = Probably yes; PN = Probably no; N = No; NA = Not applicable; NI = No information

Domain 5: Risk of bias due to missing data

Signalling questions	Response options	Comments
5.1 Were complete data on exposure status available for all, or nearly all, participants?	Y	
5.2 Were complete data on the outcome available for all, or nearly all, participants?	PY	
5.3 Were complete data on confounding variables available for all, or nearly all, participants?	Y	
5.4 **If N/PN/NI to 5.1, 5.2 or 5.3**: Is the result based on a complete case analysis?	NA	
5.5 **If Y/PY/NI**: Was exclusion from the analysis because of missing data (in exposure, confounders or the outcome) likely to be related to the true value of the outcome?	N	
5.6 **If N/PN to 5.5**: Were all or most predictors of missingness (in exposure, confounders or the outcome) included in the analysis model?	SY	
5.7 **If N/PN to 5.4**: Was the analysis based on imputing missing values?	NA	
5.8 **If Y/PY to 5.7**: Was imputation performed appropriately?	NA	
5.9 **If N/PN to 5.7**: Was an appropriate alternative method used to correct for bias due to missing data?	Y	
5.10 **If PN/N/NI to 5.1, 5.2 or 5.3**: Is there evidence that the result was not biased by missing data?	NA	
Risk of bias (due to missing data) in the estimated effect of exposure on the outcome	Low risk	
What is the predicted direction of bias due to missing data?	Towards null	
Is the risk of bias (due to missing data) sufficiently high, in the context of its likely direction and the magnitude of the estimated exposure effect, to threaten conclusions about whether the exposure has an important effect on the outcome?	No	

Y = Yes; PY = Probably yes; PN = Probably no; N = No; SY = Strong yes; WY = Weak yes; NA = Not applicable; NI = No information

Domain 6: Risk of bias arising from measurement of the outcome

Signalling questions	Response options	Comments
6.1 Could measurement or ascertainment of the outcome have differed between exposure groups or levels of exposure?	N	
6.2 Were outcome assessors aware of study participants' exposure history?	N	
6.3 **If Y/PY/NI to 6.2**: Could assessment of the outcome have been influenced by knowledge of participants' exposure history?	NA	
Risk of bias (arising from measurement of outcomes) in the estimated effect of exposure on the outcome	Low risk	
What is the predicted direction of bias arising from measurement of outcomes?	Towards null	
Is the risk of bias (arising from measurement of outcomes) sufficiently high, in the context of its likely direction and the magnitude of the estimated exposure effect, to threaten conclusions about whether the exposure has an important effect on the outcome?	No	

Y = Yes; PY = Probably yes; PN = Probably no; N = No; SY = Strong yes; WY = Weak yes; NA = Not applicable; NI = No information

Domain 7: Risk of bias in selection of the reported result

Signalling questions	Response options	Comments
7.1 Was the result reported in accordance with an available, pre-determined analysis plan?	Y	
7.2 **If N/PN/NI to 7.1**: Is the reported effect estimate likely to be selected, based on desirability of the magnitude (or statistical significance) of the estimated effect of exposure on outcome, from multiple *exposure measurements* within the exposure domain?	N	
7.3 Is the reported effect estimate likely to be selected, based on desirability of the magnitude (or statistical significance) of the estimated effect of exposure on outcome, from multiple *outcome measurements* within the outcome domain?	N	
7.4 Is the reported effect estimate likely to be selected, based on desirability of the magnitude (or statistical significance) of the estimated effect of exposure on outcome, from multiple *analyses* of the exposure-outcome relationship?	N	
7.5 Is the reported effect estimate likely to be selected, based on the basis of desirability of the results (e.g. statistical significance), from different *subgroups*?	N	
Risk of bias (due to selection of the reported result) in the estimated effect of exposure on the outcome	Low risk	
What is the predicted direction of bias due to selection of the reported result?	Towards null	
Is the risk of bias (due to selection of the reported result) sufficiently high, in the context of its likely direction and the magnitude of the estimated exposure effect, to threaten conclusions about whether the exposure has an important effect on the outcome?	No	

Y = Yes; PY = Probably yes; PN = Probably no; N = No; NA = Not applicable; NI = No information

Overall risk of bias

	Response options	Comments
Overall risk of bias	Low risk of bias	
What is the predicted direction of bias?	Towards null	
Is the overall risk of bias sufficiently high, in the context of its likely direction and the magnitude of the estimated exposure effect, to threaten conclusions about whether the exposure has an important effect on the outcome?	No	

[43] Ellfolk, M.; Leinonen, M.K.; Gissler, M.; Kiuru-Kuhlefelt, S.; Saastamoinen, L.; Malm, H. Second-generation antipsychotic use during pregnancy and risk of congenital malformations. *Eur. J. Clin. Pharmacol.* **2021**, *77*(11), 1737-1745. doi: 10.1007/s00228-021-03169-y.

Domain 1: Risk of bias due to confounding*, variant (b): **If Y/PY to C7 and Y/PY to C8** (the analysis was based on splitting participants' follow up time according to exposure status and/or magnitude and changes in exposure status and/or magnitude likely to be related to factors that are predictive of the outcome, so both baseline and time-varying confounding need to be addressed)*

Signalling questions	Response options	Comments
1.1 Did the authors use an analysis method that was appropriate to control for time-varying as well as baseline confounding?	Y	
1.2 **If Y/PY to 1.1**: Did the authors control for all the important baseline and time-varying confounding factors for which this was necessary?	PY	
1.3 **If Y/PY/WN to 1.2**: Were confounding factors that were controlled for (and for which control was necessary) measured validly and reliably by the variables available in this study?	Y	
1.4 **If N/PN/NI to 1.1**: Did the authors control for time-varying factors or other variables measured after the start of the exposure window being studied?	NA	
1.5 Did the use of negative controls, or other considerations, suggest uncontrolled confounding?	N	
Risk of bias (due to confounding) in the estimated effect of exposure on the outcome	Low risk	
What is the predicted direction of bias due to confounding?	Towards null	
Is the risk of bias (due to confounding) sufficiently high, in the context of its likely direction and the magnitude of the estimated exposure effect, to threaten conclusions about whether the exposure has an important effect on the outcome?	No	

Y = Yes; PY = Probably yes; PN = Probably no; N = No; SY = Strong yes; WY = Weak yes; SN = Strong no; WN = Weak no; NA = Not applicable; NI = No information

Domain 2: Risk of bias arising from measurement of the exposure *Variant (b): **If Y/PY to C5 and Y/PY to C6** (each individual's exposure level was estimated from measurements made at multiple time points)*

Signalling questions	Response options	Comments
**2.1 Does the measured exposure (derived from measurements at multiple time points) well-characterize the exposure metric specified to be of interest in this study? [*This was specified in the answers to D2, D3 and D4*]**	**Y**	
**2.2 Was there error in measurement, or misclassification, of the exposure, at each single time point?**	**N**	
**2.3 If SY/WY to 2.2: Could mismeasurement or misclassification of exposure have been differential (i.e. related to the outcome or risk of the outcome)?**	**NA**	
**2.4 If SY/WY to 2.2 and N/PN/WY to 2.3: Is the nature of the (non-differential) measurement error likely to bias the estimated effect of exposure on outcome?**	**NA**	
**Risk of bias (arising from measurement of exposure) in the estimated effect of exposure on the outcome**	**Low risk**	
**What is the predicted direction of bias arising from measurement of exposure?**	**Towards null**	
**Is the risk of bias (arising from measurement of exposure) sufficiently high, in the context of its likely direction and the magnitude of the estimated exposure effect, to threaten conclusions about whether the exposure has an important effect on the outcome?**	**No**	

Y = Yes; PY = Probably yes; SN = Strong no; WN = Weak no; NA = Not applicable; NI = No information

Domain 3: Risk of bias in selection of participants into the study (or into the analysis)

Signalling questions	Response options	Comments
3.1 Did follow-up begin at (or close to) the start of the exposure window for most participants? [*The exposure window is specified in D3*]	Y	
3.2 **If N/PN to 3.1**: Is the effect of exposure likely to be constant over the period of follow up analysed?	NA	
3.3 Was selection of participants into the study (or into the analysis) based on participant characteristics observed after the start of the exposure window being studied? [*The exposure window is specified in D3*]	N	
3.4 **If Y/PY to 3.3**: Were these characteristics likely to be influenced by exposure or a cause of exposure?	NA	
3.5 **If Y/PY to 3.4**: Were these characteristics likely to be influenced by the outcome or a cause of the outcome?	NA	
3.6 **If N/PN to 3.2 or Y/PY to 3.5**: Is it likely that the analysis corrected for all of the potential selection biases identified in A and B above?	NA	
3.7 **If N/PN to 3.2 or Y/PY to 3.5**: Did sensitivity analyses demonstrate that the likely impact of the potential selection biases identified in A or B above was minimal?	NA	
Risk of bias (due to selection of participants into the study) in the estimated effect of exposure on the outcome	Low risk	
What is the predicted direction of bias	Towards null	

Signalling questions	Response options	Comments
due to selection of participants into the study?		
Is the risk of bias (due to selection of participants into the study) sufficiently high, in the context of its likely direction and the magnitude of the estimated exposure effect, to threaten conclusions about whether the exposure has an important effect on the outcome?	No	

Y = Yes; PY = Probably yes; PN = Probably no; N = No; SN = Strong no; WN = Weak no; NA = Not applicable; NI = No information

Domain 4: Risk of bias due to post-exposure interventions

Signalling questions	Response options	Comments
4.1 Were there post-exposure interventions that were influenced by prior exposure during the follow-up period?	N	
4.2 **If Y/PY to 4.1**: Is it likely that the analysis corrected for the effect of post-exposure interventions that were influenced by prior exposure?	NA	
Risk of bias (due post-exposure interventions) in the estimated effect of exposure on the outcome	Low risk	
What is the predicted direction of bias due to confounding?	Towards null	
Is the risk of bias (due post-exposure interventions) sufficiently high, in the context of its likely direction and the magnitude of the estimated exposure effect, to threaten conclusions about whether the exposure has an important effect on the outcome?	No	

Y = Yes; PY = Probably yes; PN = Probably no; N = No; NA = Not applicable; NI = No information

Domain 5: Risk of bias due to missing data

Signalling questions	Response options	Comments
5.1 Were complete data on exposure status available for all, or nearly all, participants?	Y	
5.2 Were complete data on the outcome available for all, or nearly all, participants?	Y	
5.3 Were complete data on confounding variables available for all, or nearly all, participants?	Y	
5.4 **If N/PN/NI to 5.1, 5.2 or 5.3**: Is the result based on a complete case analysis?	NA	
5.5 **If Y/PY/NI**: Was exclusion from the analysis because of missing data (in exposure, confounders or the outcome) likely to be related to the true value of the outcome?	N	
5.6 **If N/PN to 5.5**: Were all or most predictors of missingness (in exposure, confounders or the outcome) included in the analysis model?	SY	
5.7 **If N/PN to 5.4**: Was the analysis based on imputing missing values?	NA	
5.8 **If Y/PY to 5.7**: Was imputation performed appropriately?	NA	
5.9 **If N/PN to 5.7**: Was an appropriate alternative method used to correct for bias due to missing data?	Y	
5.10 **If PN/N/NI to 5.1, 5.2 or 5.3**: Is there evidence that the result was not biased by missing data?	NA	
Risk of bias (due to missing data) in the estimated effect of exposure on the outcome	Low risk	
What is the predicted direction of bias due to missing data?	Towards null	
Is the risk of bias (due to missing data) sufficiently high, in the context of its likely direction and the magnitude of the estimated exposure effect, to threaten conclusions about whether the exposure has an important effect on the outcome?	No	

Y = Yes; PY = Probably yes; PN = Probably no; N = No; SY = Strong yes; WY = Weak yes; NA = Not applicable; NI = No information

Domain 6: Risk of bias arising from measurement of the outcome

Signalling questions	Response options	Comments
6.1 Could measurement or ascertainment of the outcome have differed between exposure groups or levels of exposure?	N	
6.2 Were outcome assessors aware of study participants' exposure history?	N	
6.3 **If Y/PY/NI to 6.2**: Could assessment of the outcome have been influenced by knowledge of participants' exposure history?	NA	
Risk of bias (arising from measurement of outcomes) in the estimated effect of exposure on the outcome	Low risk	
What is the predicted direction of bias arising from measurement of outcomes?	Towards null	
Is the risk of bias (arising from measurement of outcomes) sufficiently high, in the context of its likely direction and the magnitude of the estimated exposure effect, to threaten conclusions about whether the exposure has an important effect on the outcome?	No	

Y = Yes; PY = Probably yes; PN = Probably no; N = No; SY = Strong yes; WY = Weak yes; NA = Not applicable; NI = No information

Domain 7: Risk of bias in selection of the reported result

Signalling questions	Response options	Comments
7.1 Was the result reported in accordance with an available, pre-determined analysis plan?	Y	
7.2 **If N/PN/NI to 7.1**: Is the reported effect estimate likely to be selected, based on desirability of the magnitude (or statistical significance) of the estimated effect of exposure on outcome, from multiple *exposure measurements* within the exposure domain?	N	
7.3 Is the reported effect estimate likely to be selected, based on desirability of the magnitude (or statistical significance) of the estimated effect of exposure on outcome, from multiple *outcome measurements* within the outcome domain?	N	
7.4 Is the reported effect estimate likely to be selected, based on desirability of the magnitude (or statistical significance) of the estimated effect of exposure on outcome, from multiple *analyses* of the exposure-outcome relationship?	N	
7.5 Is the reported effect estimate likely to be selected, based on the basis of desirability of the results (e.g. statistical significance), from different *subgroups*?	N	

Signalling questions	Response options	Comments
Risk of bias (due to selection of the reported result) in the estimated effect of exposure on the outcome	Low risk	
What is the predicted direction of bias due to selection of the reported result?	Towards null	
Is the risk of bias (due to selection of the reported result) sufficiently high, in the context of its likely direction and the magnitude of the estimated exposure effect, to threaten conclusions about whether the exposure has an important effect on the outcome?	No	

Y = Yes; PY = Probably yes; PN = Probably no; N = No; NA = Not applicable; NI = No information

Overall risk of bias

	Response options	Comments
Overall risk of bias	Low risk of bias	
What is the predicted direction of bias?	Towards null	
Is the overall risk of bias sufficiently high, in the context of its likely direction and the magnitude of the estimated exposure effect, to threaten conclusions about whether the exposure has an important effect on the outcome?	No	

[44] Freeman, M.P.; Viguera, A.C.; Góez-Mogollón, L.; Young, A.V.; Caplin, P.S.; McElheny, S.A.; Church, T.R.; Chitayat, D.; Hernández-Díaz, S.; Cohen, L.S. Reproductive safety of aripiprazole: data from the Massachusetts General Hospital National Pregnancy Registry for Atypical Antipsychotics. *Arch. Womens Ment. Health* **2021**, *24*(4), 659-667. doi: 10.1007/s00737-021-01115-6.

Domain 1: Risk of bias due to confounding *variant (b): **If Y/PY to C7 and Y/PY to C8** (the analysis was based on splitting participants' follow up time according to exposure status and/or magnitude and changes in exposure status and/or magnitude likely to be related to factors that are predictive of the outcome, so both baseline and time-varying confounding need to be addressed)*

Signalling questions	Response options	Comments
1.1 Did the authors use an analysis method that was appropriate to control for time-varying as well as baseline confounding?	Y	
1.2 **If Y/PY to 1.1**: Did the authors control for all the important baseline and time-varying confounding factors for which this was necessary?	Y	
1.3 **If Y/PY/WN to 1.2**: Were confounding factors that were controlled for (and for which control was necessary) measured validly and reliably by the variables available in this study?	PY	
1.4 **If N/PN/NI to 1.1**: Did the authors control for time-varying factors or other variables measured after the start of the exposure window being studied?	N	
1.5 Did the use of negative controls, or other considerations, suggest uncontrolled confounding?	N	
Risk of bias (due to confounding) in the estimated effect of exposure on the outcome	Low risk	
What is the predicted direction of bias due to confounding?	Towards null	
Is the risk of bias (due to confounding) sufficiently high, in the context of its likely direction and the magnitude of the estimated exposure effect, to threaten conclusions about whether the exposure has an important effect on the outcome?	No	

Y = Yes; PY = Probably yes; PN = Probably no; N = No; SY = Strong yes; WY = Weak yes; SN = Strong no; WN = Weak no; NA = Not applicable; NI = No information

Domain 2: Risk of bias arising from measurement of the exposure *Variant (b): **If Y/PY to C5 and Y/PY to C6** (each individual's exposure level was estimated from measurements made at multiple time points)*

Signalling questions	Response options	Comments
**2.1 Does the measured exposure (derived from measurements at multiple time points) well-characterize the exposure metric specified to be of interest in this study? [*This was specified in the answers to D2, D3 and D4*]**	**Y**	
**2.2 Was there error in measurement, or misclassification, of the exposure, at each single time point?**	**Y**	
**2.3 If SY/WY to 2.2: Could mismeasurement or misclassification of exposure have been differential (i.e. related to the outcome or risk of the outcome)?**	**N**	
**2.4 If SY/WY to 2.2 and N/PN/WY to 2.3: Is the nature of the (non-differential) measurement error likely to bias the estimated effect of exposure on outcome?**	**PN**	
**Risk of bias (arising from measurement of exposure) in the estimated effect of exposure on the outcome**	**PN**	
**What is the predicted direction of bias arising from measurement of exposure?**	**Low risk**	
**Is the risk of bias (arising from measurement of exposure) sufficiently high, in the context of its likely direction and the magnitude of the estimated exposure effect, to threaten conclusions about whether the exposure has an important effect on the outcome?**	**Towards null**	

Y = Yes; PY = Probably yes; SN = Strong no; WN = Weak no; NA = Not applicable; NI = No information

Domain 3: Risk of bias in selection of participants into the study (or into the analysis)

Signalling questions	Response options	Comments
3.1 Did follow-up begin at (or close to) the start of the exposure window for most participants? [*The exposure window is*	Y	

Signalling questions	Response options	Comments
specified in D3]		
3.2 **If N/PN to 3.1**: Is the effect of exposure likely to be constant over the period of follow up analysed?	Y	
3.3 Was selection of participants into the study (or into the analysis) based on participant characteristics observed after the start of the exposure window being studied? [*The exposure window is specified in D3*]	N	
3.4 **If Y/PY to 3.3**: Were these characteristics likely to be influenced by exposure or a cause of exposure?	N	
3.5 **If Y/PY to 3.4**: Were these characteristics likely to be influenced by the outcome or a cause of the outcome?	PN	
3.6 **If N/PN to 3.2 or Y/PY to 3.5**: Is it likely that the analysis corrected for all of the potential selection biases identified in A and B above?	Y	
3.7 **If N/PN to 3.2 or Y/PY to 3.5**: Did sensitivity analyses demonstrate that the likely impact of the potential selection biases identified in A or B above was minimal?	PY	
Risk of bias (due to selection of participants into the study) in the estimated effect of exposure on the outcome	Low risk	
What is the predicted direction of bias due to selection of participants into the study?	Towards null	
Is the risk of bias (due to selection of participants into the study) sufficiently high, in the context of its likely direction and the magnitude of the estimated exposure effect, to threaten conclusions about whether the exposure has an important effect on the outcome?	No	

Y = Yes; PY = Probably yes; PN = Probably no; N = No; SN = Strong no; WN = Weak no; NA = Not applicable; NI = No information

Domain 4: Risk of bias due to post-exposure interventions

Signalling questions	Response options	Comments
4.1 Were there post-exposure interventions that were influenced by prior exposure during the follow-up period?	N	
4.2 **If Y/PY to 4.1**: Is it likely that the analysis corrected for the effect of post-exposure interventions that were influenced by prior exposure?	NA	
Risk of bias (due post-exposure interventions) in the estimated effect of exposure on the outcome	Low risk	
What is the predicted direction of bias due to confounding?	Towards null	
Is the risk of bias (due post-exposure interventions) sufficiently high, in the context of its likely direction and the magnitude of the estimated exposure effect, to threaten conclusions about whether the exposure has an important effect on the outcome?	No	

Y = Yes; PY = Probably yes; PN = Probably no; N = No; NA = Not applicable; NI = No information

Domain 5: Risk of bias due to missing data

Signalling questions	Response options	Comments
5.1 Were complete data on exposure status available for all, or nearly all, participants?	Y	
5.2 Were complete data on the outcome available for all, or nearly all, participants?	Y	
5.3 Were complete data on confounding variables available for all, or nearly all, participants?	Y	
5.4 **If N/PN/NI to 5.1, 5.2 or 5.3**: Is the result based on a complete case analysis?	Y	
5.5 **If Y/PY/NI**: Was exclusion from the analysis because of missing data (in exposure, confounders or the outcome) likely to be related to the true value of the outcome?	N	
5.6 **If N/PN to 5.5**: Were all or most predictors of missingness (in exposure, confounders or the outcome) included in the analysis model?	SY	
5.7 **If N/PN to 5.4**: Was the analysis based on imputing missing values?	Y	
5.8 **If Y/PY to 5.7**: Was imputation performed appropriately?	Y	
5.9 **If N/PN to 5.7**: Was an appropriate alternative method used to correct for bias due to missing data?	Y	
5.10 **If PN/N/NI to 5.1, 5.2 or 5.3**: Is there evidence that the result was not biased by missing data?	Y	
Risk of bias (due to missing data) in the estimated effect of exposure on the outcome	Low risk	
What is the predicted direction of bias due to missing data?	Towards null	
Is the risk of bias (due to missing data) sufficiently high, in the context of its likely direction and the magnitude of the estimated exposure effect, to threaten conclusions about whether the exposure has an important effect on the outcome?	No	

Y = Yes; PY = Probably yes; PN = Probably no; N = No; SY = Strong yes; WY = Weak yes; NA = Not applicable; NI = No information

Domain 6: Risk of bias arising from measurement of the outcome

Signalling questions	Response options	Comments
6.1 Could measurement or ascertainment of the outcome have differed between exposure groups or levels of exposure?	N	
6.2 Were outcome assessors aware of study participants' exposure history?	PN	
6.3 **If Y/PY/NI to 6.2**: Could assessment of the outcome have been influenced by knowledge of participants' exposure history?	N	
Risk of bias (arising from measurement of outcomes) in the estimated effect of exposure on the outcome	Low risk	
What is the predicted direction of bias arising from measurement of outcomes?	Towards null	
Is the risk of bias (arising from measurement of outcomes) sufficiently high, in the context of its likely direction and the magnitude of the estimated exposure effect, to threaten conclusions about whether the exposure has an important effect on the outcome?	No	

Y = Yes; PY = Probably yes; PN = Probably no; N = No; SY = Strong yes; WY = Weak yes; NA = Not applicable; NI = No information

Domain 7: Risk of bias in selection of the reported result

Signalling questions	Response options	Comments
7.1 Was the result reported in accordance with an available, pre-determined analysis plan?	Y	
7.2 **If N/PN/NI to 7.1**: Is the reported effect estimate likely to be selected, based on desirability of the magnitude (or statistical significance) of the estimated effect of exposure on outcome, from multiple *exposure measurements* within the exposure domain?	N	
7.3 Is the reported effect estimate likely to be selected, based on desirability of the magnitude (or statistical significance) of the estimated effect of exposure on outcome, from multiple *outcome measurements* within the outcome domain?	N	
7.4 Is the reported effect estimate likely to be selected, based on desirability of the magnitude (or statistical significance) of the estimated effect of exposure on outcome, from multiple *analyses* of the exposure-outcome relationship?	N	
7.5 Is the reported effect estimate likely to be selected, based on the basis of desirability of the results (e.g. statistical significance), from different *subgroups*?	N	
Risk of bias (due to selection of the reported result) in the estimated effect of exposure on the outcome	Low risk	
What is the predicted direction of bias due to selection of the reported result?	Towards null	
Is the risk of bias (due to selection of the reported result) sufficiently high, in the context of its likely direction and the magnitude of the estimated exposure effect, to threaten conclusions about whether the exposure has an important effect on the outcome?	No	

Y = Yes; PY = Probably yes; PN = Probably no; N = No; NA = Not applicable; NI = No information

Overall risk of bias

	Response options	Comments
Overall risk of bias	Low risk of bias	
What is the predicted direction of bias?	Towards null	
Is the overall risk of bias sufficiently high, in the context of its likely direction and the magnitude of the estimated exposure effect, to threaten conclusions about whether the exposure has an important effect on the outcome?	No	

[45] Viguera, A.C.; Freeman, M.P.; Góez-Mogollón, L.; Sosinsky, A.Z.; McElheny, S.A.; Church, T.R.; Young, A.V.; Caplin, P.S.; Chitayat, D.; Hernández-Díaz, S.; Cohen, L.S. Reproductive safety of second-generation antipsychotics: Updated data from the Massachusetts General Hospital National Pregnancy Registry for Atypical Antipsychotics. *J. Clin. Psychiatry* **2021**, *82*(4), 20m13745. doi: 10.4088/JCP.20m13745. Erratum in: *J. Clin. Psychiatry* 2021, 82(5).

Domain 1: Risk of bias due to confounding *variant (b): **If Y/PY to C7 and Y/PY to C8** (the analysis was based on splitting participants' follow up time according to exposure status and/or magnitude and changes in exposure status and/or magnitude likely to be related to factors that are predictive of the outcome, so both baseline and time-varying confounding need to be addressed)*

Signalling questions	Response options	Comments
1.1 Did the authors use an analysis method that was appropriate to control for time-varying as well as baseline confounding?	Y	
1.2 **If Y/PY to 1.1**: Did the authors control for all the important baseline and time-varying confounding factors for which this was necessary?	Y	
1.3 **If Y/PY/WN to 1.2**: Were confounding factors that were controlled for (and for which control was necessary) measured validly and reliably by the variables available in this study?	Y	
1.4 **If N/PN/NI to 1.1**: Did the authors control for time-varying factors or other variables measured after the start of the exposure window being studied?	N	
1.5 Did the use of negative controls, or other considerations, suggest uncontrolled confounding?	N	
Risk of bias (due to confounding) in the estimated effect of exposure on the outcome	Low risk	
What is the predicted direction of bias due to confounding?	Towards null	
Is the risk of bias (due to confounding) sufficiently high, in the context of its likely direction and the magnitude of the estimated exposure effect, to threaten conclusions about whether the exposure has an important effect on the outcome?	No	

Y = Yes; PY = Probably yes; PN = Probably no; N = No; SY = Strong yes; WY = Weak yes; SN = Strong no; WN = Weak no; NA = Not applicable; NI = No information

Domain 2: Risk of bias arising from measurement of the exposure *Variant (b): **If Y/PY to C5 and Y/PY to C6** (each individual's exposure level was estimated from measurements made at multiple time points)*

Signalling questions	Response options	Comments
**2.1 Does the measured exposure (derived from measurements at multiple time points) well-characterize the exposure metric specified to be of interest in this study? [*This was specified in the answers to D2, D3 and D4*]**	Y	
**2.2 Was there error in measurement, or misclassification, of the exposure, at each single time point?**	Y	

Signalling questions	Response options	Comments
**2.3 If SY/WY to 2.2: Could mismeasurement or misclassification of exposure have been differential (i.e. related to the outcome or risk of the outcome)?**	**N**	
**2.4 If SY/WY to 2.2 and N/PN/WY to 2.3: Is the nature of the (non-differential) measurement error likely to bias the estimated effect of exposure on outcome?**	**N**	
**Risk of bias (arising from measurement of exposure) in the estimated effect of exposure on the outcome**	**PN**	
**What is the predicted direction of bias arising from measurement of exposure?**	**Low risk**	
**Is the risk of bias (arising from measurement of exposure) sufficiently high, in the context of its likely direction and the magnitude of the estimated exposure effect, to threaten conclusions about whether the exposure has an important effect on the outcome?**	**Towards null**	

Y = Yes; PY = Probably yes; SN = Strong no; WN = Weak no; NA = Not applicable; NI = No information

Domain 3: Risk of bias in selection of participants into the study (or into the analysis)

Signalling questions	Response options	Comments
3.1 Did follow-up begin at (or close to) the start of the exposure window for most participants? [*The exposure window is specified in D3*]	Y	
3.2 **If N/PN to 3.1**: Is the effect of exposure likely to be constant over the period of follow up analysed?	Y	
3.3 Was selection of participants into the study (or into the analysis) based on participant characteristics observed after the start of the exposure window being studied? [*The exposure window is specified in D3*]	N	
3.4 **If Y/PY to 3.3**: Were these characteristics likely to be influenced by exposure or a cause of exposure?	N	
3.5 **If Y/PY to 3.4**: Were these characteristics likely to be influenced by the outcome or a cause of the outcome?	PN	
3.6 **If N/PN to 3.2 or Y/PY to 3.5**: Is it likely that the analysis corrected for all of the potential selection biases identified in A and B above?	Y	
3.7 **If N/PN to 3.2 or Y/PY to 3.5**: Did sensitivity analyses demonstrate that the likely impact of the potential selection biases identified in A or B above was minimal?	PY	
Risk of bias (due to selection of participants into the study) in the estimated effect of exposure on the outcome	Low risk	
What is the predicted direction of bias due to selection of participants into the study?	Towards null	
Is the risk of bias (due to selection of participants into the study) sufficiently high, in the context of its likely direction and the magnitude of the estimated exposure effect, to threaten conclusions about whether the exposure has an important effect on the outcome?	No	

Y = Yes; PY = Probably yes; PN = Probably no; N = No; SN = Strong no; WN = Weak no; NA = Not applicable; NI = No information

Domain 4: Risk of bias due to post-exposure interventions

Signalling questions	Response options	Comments
4.1 Were there post-exposure interventions that were influenced by prior exposure during the follow-up period?	N	
4.2 **If Y/PY to 4.1**: Is it likely that the analysis corrected for the effect of post-exposure interventions that were influenced by prior exposure?	NA	
Risk of bias (due post-exposure interventions) in the estimated effect of exposure on the outcome	Low risk	
What is the predicted direction of bias due to confounding?	Towards null	
Is the risk of bias (due post-exposure interventions) sufficiently high, in the context of its likely direction and the magnitude of the estimated exposure effect, to threaten conclusions about whether the exposure has an important effect on the outcome?	No	

Y = Yes; PY = Probably yes; PN = Probably no; N = No; NA = Not applicable; NI = No information

Domain 5: Risk of bias due to missing data

Signalling questions	Response options	Comments
5.1 Were complete data on exposure status available for all, or nearly all, participants?	Y	
5.2 Were complete data on the outcome available for all, or nearly all, participants?	Y	
5.3 Were complete data on confounding variables available for all, or nearly all, participants?	Y	
5.4 **If N/PN/NI to 5.1, 5.2 or 5.3**: Is the result based on a complete case analysis?	Y	
5.5 **If Y/PY/NI**: Was exclusion from the analysis because of missing data (in exposure, confounders or the outcome) likely to be related to the true value of the outcome?	N	
5.6 **If N/PN to 5.5**: Were all or most predictors of missingness (in exposure, confounders or the outcome) included in the analysis model?	SY	
5.7 **If N/PN to 5.4**: Was the analysis based on imputing missing values?	Y	
5.8 **If Y/PY to 5.7**: Was imputation performed appropriately?	Y	
5.9 **If N/PN to 5.7**: Was an appropriate alternative method used to correct for bias due to missing data?	Y	
5.10 **If PN/N/NI to 5.1, 5.2 or 5.3**: Is there evidence that the result was not biased by missing data?	Y	
Risk of bias (due to missing data) in the estimated effect of exposure on the outcome	Low risk	
What is the predicted direction of bias due to missing data?	Towards null	
Is the risk of bias (due to missing data) sufficiently high, in the context of its likely direction and the magnitude of the estimated exposure effect, to threaten conclusions about whether the exposure has an important effect on the outcome?	No	

Y = Yes; PY = Probably yes; PN = Probably no; N = No; SY = Strong yes; WY = Weak yes; NA = Not applicable; NI = No information

Domain 6: Risk of bias arising from measurement of the outcome

Signalling questions	Response options	Comments
6.1 Could measurement or ascertainment of the outcome have differed between exposure groups or levels of exposure?	N	
6.2 Were outcome assessors aware of study participants' exposure history?	N	
6.3 **If Y/PY/NI to 6.2**: Could assessment of the outcome have been influenced by knowledge of participants' exposure history?	N	
Risk of bias (arising from measurement of outcomes) in the estimated effect of exposure on the outcome	Low risk	
What is the predicted direction of bias arising from measurement of outcomes?	Towards null	
Is the risk of bias (arising from measurement of outcomes) sufficiently high, in the context of its likely direction and the magnitude of the estimated exposure effect, to threaten conclusions about whether the exposure has an important effect on the outcome?	No	

Y = Yes; PY = Probably yes; PN = Probably no; N = No; SY = Strong yes; WY = Weak yes; NA = Not applicable; NI = No information

Domain 7: Risk of bias in selection of the reported result

Signalling questions	Response options	Comments
7.1 Was the result reported in accordance with an available, pre-determined analysis plan?	Y	
7.2 **If N/PN/NI to 7.1**: Is the reported effect estimate likely to be selected, based on desirability of the magnitude (or statistical significance) of the estimated effect of exposure on outcome, from multiple *exposure measurements* within the exposure domain?	N	
7.3 Is the reported effect estimate likely to be selected, based on desirability of the magnitude (or statistical significance) of the estimated effect of exposure on outcome, from multiple *outcome measurements* within the outcome domain?	N	
7.4 Is the reported effect estimate likely to be selected, based on desirability of the magnitude (or statistical significance) of the estimated effect of exposure on outcome, from multiple *analyses* of the exposure-outcome relationship?	N	
7.5 Is the reported effect estimate likely to be selected, based on the basis of desirability of the results (e.g. statistical significance), from different *subgroups*?	N	
Risk of bias (due to selection of the reported result) in the estimated effect of exposure on the outcome	Low risk	
What is the predicted direction of bias due to selection of the reported result?	Towards null	
Is the risk of bias (due to selection of the reported result) sufficiently high, in the context of its likely direction and the magnitude of the estimated exposure effect, to threaten conclusions about whether the exposure has an important effect on the outcome?	No	

Y = Yes; PY = Probably yes; PN = Probably no; N = No; NA = Not applicable; NI = No information

Overall risk of bias

	Response options	Comments
Overall risk of bias	Low risk of bias	
What is the predicted direction of bias?	Towards null	
Is the overall risk of bias sufficiently high, in the context of its likely direction and the magnitude of the estimated exposure effect, to threaten conclusions about whether the exposure has an important effect on the outcome?	No	

[46] Nguyen, T.; Frayne, J.; Watson, S.; Lebedevs, T.; Teoh, S.; Galbally, M. Long-acting injectable antipsychotic treatment during pregnancy: Outcomes for women at a tertiary maternity hospital. *Psychiatry Res.* **2022**, *313*, 114614. doi: 10.1016/j.psychres.2022.114614.

Domain 1: Risk of bias due to confounding *variant (b): **If Y/PY to C7 and Y/PY to C8** (the analysis was based on splitting participants' follow up time according to exposure status and/or magnitude and changes in exposure status and/or magnitude likely to be related to factors that are predictive of the outcome, so both baseline and time-varying confounding need to be addressed)*

Signalling questions	Response options	Comments
1.1 Did the authors use an analysis method that was appropriate to control for time-varying as well as baseline confounding?	Y	
1.2 **If Y/PY to 1.1**: Did the authors control for all the important baseline and time-varying confounding factors for which this was necessary?	Y	
1.3 **If Y/PY/WN to 1.2**: Were confounding factors that were controlled for (and for which control was necessary) measured validly and reliably by the variables available in this study?	Y	
1.4 **If N/PN/NI to 1.1**: Did the authors control for time-varying factors or other variables measured after the start of the exposure window being studied?	N	
1.5 Did the use of negative controls, or other considerations, suggest uncontrolled confounding?	N	
Risk of bias (due to confounding) in the estimated effect of exposure on the outcome	Low risk	
What is the predicted direction of bias due to confounding?	Towards null	
Is the risk of bias (due to confounding) sufficiently high, in the context of its likely direction and the magnitude of the estimated exposure effect, to threaten conclusions about whether the exposure has an important effect on the outcome?	No	

Y = Yes; PY = Probably yes; PN = Probably no; N = No; SY = Strong yes; WY = Weak yes; SN = Strong no; WN = Weak no; NA = Not applicable; NI = No information

***Domain 2: Risk of bias arising from measurement of the exposure** Variant (b): **If Y/PY to C5 and Y/PY to C6** (each individual's exposure level was estimated from measurements made at multiple time points)*

Signalling questions	Response options	Comments
**2.1 Does the measured exposure (derived from measurements at multiple time points) well-characterize the exposure metric specified to be of interest in this study? [*This was specified in the answers to D2, D3 and D4*]**	**Y**	
**2.2 Was there error in measurement, or misclassification, of the exposure, at each single time point?**	**Y**	
**2.3 If SY/WY to 2.2: Could mismeasurement or misclassification of exposure have been differential (i.e. related to the outcome or risk of the outcome)?**	**N**	
**2.4 If SY/WY to 2.2 and N/PN/WY to 2.3: Is the nature of the (non-differential) measurement error likely to bias the estimated effect of exposure on outcome?**	**N**	
**Risk of bias (arising from measurement of exposure) in the estimated effect of exposure on the outcome**	**PN**	
**What is the predicted direction of bias arising from measurement of exposure?**	**Low risk**	
**Is the risk of bias (arising from measurement of exposure) sufficiently high, in the context of its likely direction and the magnitude of the estimated exposure effect, to threaten conclusions about whether the exposure has an important effect on the outcome?**	**Towards null**	

Y = Yes; PY = Probably yes; SN = Strong no; WN = Weak no; NA = Not applicable; NI = No information

Domain 3: Risk of bias in selection of participants into the study (or into the analysis)

Signalling questions	Response options	Comments
3.1 Did follow-up begin at (or close to) the start of the exposure window for most participants? [*The exposure window is specified in D3*]	Y	
3.2 **If N/PN to 3.1**: Is the effect of exposure likely to be constant over the period of follow up analysed?	Y	
3.3 Was selection of participants into the study (or into the analysis) based on participant characteristics observed after the start of the exposure window being studied? [*The exposure window is specified in D3*]	N	
3.4 **If Y/PY to 3.3**: Were these characteristics likely to be influenced by exposure or a cause of exposure?	N	
3.5 **If Y/PY to 3.4**: Were these characteristics likely to be influenced by the outcome or a cause of the outcome?	PN	
3.6 **If N/PN to 3.2 or Y/PY to 3.5**: Is it likely that the analysis corrected for all of the potential selection biases identified in A and B above?	Y	
3.7 **If N/PN to 3.2 or Y/PY to 3.5**: Did sensitivity analyses demonstrate that the likely impact of the potential selection biases identified in A or B above was minimal?	PY	
Risk of bias (due to selection of participants into the study) in the estimated effect of exposure on the outcome	Low risk	
What is the predicted direction of bias due to selection of participants into the study?	Towards null	
Is the risk of bias (due to selection of participants into the study) sufficiently high, in the context of its likely direction and the magnitude of the estimated exposure effect, to threaten conclusions about whether the exposure has an important effect on the outcome?	No	

Y = Yes; PY = Probably yes; PN = Probably no; N = No; SN = Strong no; WN = Weak no; NA = Not applicable; NI = No information

Domain 4: Risk of bias due to post-exposure interventions

Signalling questions	Response options	Comments
4.1 Were there post-exposure interventions that were influenced by prior exposure during the follow-up period?	N	
4.2 **If Y/PY to 4.1**: Is it likely that the analysis corrected for the effect of post-exposure interventions that were influenced by prior exposure?	NA	
Risk of bias (due post-exposure interventions) in the estimated effect of exposure on the outcome	Low risk	
What is the predicted direction of bias due to confounding?	Towards null	
Is the risk of bias (due post-exposure interventions) sufficiently high, in the context of its likely direction and the magnitude of the estimated exposure effect, to threaten conclusions about whether the exposure has an important effect on the outcome?	No	

Y = Yes; PY = Probably yes; PN = Probably no; N = No; NA = Not applicable; NI = No information

Domain 5: Risk of bias due to missing data

Signalling questions	Response options	Comments
5.1 Were complete data on exposure status available for all, or nearly all, participants?	Y	
5.2 Were complete data on the outcome available for all, or nearly all, participants?	Y	
5.3 Were complete data on confounding variables available for all, or nearly all, participants?	Y	
5.4 **If N/PN/NI to 5.1, 5.2 or 5.3**: Is the result based on a complete case analysis?	Y	
5.5 **If Y/PY/NI**: Was exclusion from the analysis because of missing data (in exposure, confounders or the outcome) likely to be related to the true value of the outcome?	N	
5.6 **If N/PN to 5.5**: Were all or most predictors of missingness (in exposure, confounders or the outcome) included in the analysis model?	SY	
5.7 **If N/PN to 5.4**: Was the analysis based on imputing missing values?	Y	

Signalling questions	Response options	Comments
5.8 **If Y/PY to 5.7**: Was imputation performed appropriately?	Y	
5.9 **If N/PN to 5.7**: Was an appropriate alternative method used to correct for bias due to missing data?	Y	
5.10 **If PN/N/NI to 5.1, 5.2 or 5.3**: Is there evidence that the result was not biased by missing data?	Y	
Risk of bias (due to missing data) in the estimated effect of exposure on the outcome	Low risk	
What is the predicted direction of bias due to missing data?	Towards null	
Is the risk of bias (due to missing data) sufficiently high, in the context of its likely direction and the magnitude of the estimated exposure effect, to threaten conclusions about whether the exposure has an important effect on the outcome?	No	

Y = Yes; PY = Probably yes; PN = Probably no; N = No; SY = Strong yes; WY = Weak yes; NA = Not applicable; NI = No information

Domain 6: Risk of bias arising from measurement of the outcome

Signalling questions	Response options	Comments
6.1 Could measurement or ascertainment of the outcome have differed between exposure groups or levels of exposure?	N	
6.2 Were outcome assessors aware of study participants' exposure history?	N	
6.3 **If Y/PY/NI to 6.2**: Could assessment of the outcome have been influenced by knowledge of participants' exposure history?	N	
Risk of bias (arising from measurement of outcomes) in the estimated effect of exposure on the outcome	Low risk	
What is the predicted direction of bias arising from measurement of outcomes?	Towards null	
Is the risk of bias (arising from measurement of outcomes) sufficiently high, in the context of its likely direction and the magnitude of the estimated exposure effect, to threaten conclusions about whether the exposure has an important effect on the outcome?	No	

Y = Yes; PY = Probably yes; PN = Probably no; N = No; SY = Strong yes; WY = Weak yes; NA = Not applicable; NI = No information

Domain 7: Risk of bias in selection of the reported result

Signalling questions	Response options	Comments
7.1 Was the result reported in accordance with an available, pre-determined analysis plan?	Y	
7.2 **If N/PN/NI to 7.1**: Is the reported effect estimate likely to be selected, based on desirability of the magnitude (or statistical significance) of the estimated effect of exposure on outcome, from multiple *exposure measurements* within the exposure domain?	N	
7.3 Is the reported effect estimate likely to be selected, based on desirability of the magnitude (or statistical significance) of the estimated effect of exposure on outcome, from multiple *outcome measurements* within the outcome domain?	N	
7.4 Is the reported effect estimate likely to be selected, based on desirability of the magnitude (or statistical significance) of the estimated effect of exposure on outcome, from multiple *analyses* of the exposure-outcome relationship?	N	
7.5 Is the reported effect estimate likely to be selected, based on the basis of desirability of the results (e.g. statistical significance), from different *subgroups*?	N	
Risk of bias (due to selection of the reported result) in the estimated effect of exposure on the outcome	Low risk	
What is the predicted direction of bias due to selection of the reported result?	Towards null	
Is the risk of bias (due to selection of the reported result) sufficiently high, in the context of its likely direction and the magnitude of the estimated exposure effect, to threaten conclusions about whether the exposure has an important effect on the outcome?	No	

Y = Yes; PY = Probably yes; PN = Probably no; N = No; NA = Not applicable; NI = No information

Overall risk of bias

	Response options	Comments
Overall risk of bias	Low risk of bias	
What is the predicted direction of bias?	Towards null	
Is the overall risk of bias sufficiently high, in the context of its likely direction and the magnitude of the estimated exposure effect, to threaten conclusions about whether the exposure has an important effect on the outcome?	No	

[47] Yakuwa, N.; Takahashi, K.; Anzai, T.; Ito, N.; Goto, M.; Koinuma, S.; Uno, C.; Suzuki, T.; Watanabe, O.; Yamatani, A.; Murashima, A. Pregnancy outcomes with exposure to second-generation antipsychotics during the first trimester. *J. Clin. Psychiatry* **2022**, *83*(4), 21m14081. doi: 10.4088/JCP.21m14081.

Domain 1: Risk of bias due to confounding *variant (b): **If Y/PY to C7 and Y/PY to C8** (the analysis was based on splitting participants' follow up time according to exposure status and/or magnitude and changes in exposure status and/or magnitude likely to be related to factors that are predictive of the outcome, so both baseline and time-varying confounding need to be addressed)*

Signalling questions	Response options	Comments
1.1 Did the authors use an analysis method that was appropriate to control for time-varying as well as baseline confounding?	Y	
1.2 **If Y/PY to 1.1**: Did the authors control for all the important baseline and time-varying confounding factors for which this was necessary?	Y	
1.3 **If Y/PY/WN to 1.2**: Were confounding factors that were controlled for (and for which control was necessary) measured validly and reliably by the variables available in this study?	Y	

Signalling questions	Response options	Comments
1.4 **If N/PN/NI to 1.1**: Did the authors control for time-varying factors or other variables measured after the start of the exposure window being studied?	N	
1.5 Did the use of negative controls, or other considerations, suggest uncontrolled confounding?	N	
Risk of bias (due to confounding) in the estimated effect of exposure on the outcome	Low risk	
What is the predicted direction of bias due to confounding?	Towards null	
Is the risk of bias (due to confounding) sufficiently high, in the context of its likely direction and the magnitude of the estimated exposure effect, to threaten conclusions about whether the exposure has an important effect on the outcome?	No	

Y = Yes; PY = Probably yes; PN = Probably no; N = No; SY = Strong yes; WY = Weak yes; SN = Strong no; WN = Weak no; NA = Not applicable; NI = No information

***Domain 2: Risk of bias arising from measurement of the exposure** Variant (b): **If Y/PY to C5 and Y/PY to C6** (each individual's exposure level was estimated from measurements made at multiple time points)*

Signalling questions	Response options	Comments
**2.1 Does the measured exposure (derived from measurements at multiple time points) well-characterize the exposure metric specified to be of interest in this study? [*This was specified in the answers to D2, D3 and D4*]**	**Y**	
**2.2 Was there error in measurement, or misclassification, of the exposure, at each single time point?**	**Y**	
**2.3 If SY/WY to 2.2: Could mismeasurement or misclassification of exposure have been differential (i.e. related to the outcome or risk of the outcome)?**	**N**	
**2.4 If SY/WY to 2.2 and N/PN/WY to 2.3: Is the nature of the (non-differential) measurement error likely to bias the estimated effect of exposure on outcome?**	**N**	
**Risk of bias (arising from measurement of exposure) in the estimated effect of exposure on the outcome**	**PN**	
**What is the predicted direction of bias arising from measurement of exposure?**	**Low risk**	
**Is the risk of bias (arising from measurement of exposure) sufficiently high, in the context of its likely direction and the magnitude of the estimated exposure effect, to threaten conclusions about whether the exposure has an important effect on the outcome?**	**Towards null**	

Y = Yes; PY = Probably yes; SN = Strong no; WN = Weak no; NA = Not applicable; NI = No information

Domain 3: Risk of bias in selection of participants into the study (or into the analysis)

Signalling questions	Response options	Comments
3.1 Did follow-up begin at (or close to) the start of the exposure window for most participants? [*The exposure window is specified in D3*]	Y	
3.2 **If N/PN to 3.1**: Is the effect of exposure likely to be constant over the period of follow up analysed?	Y	
3.3 Was selection of participants into the study (or into the analysis) based on participant characteristics observed after the start of the exposure window being studied? [*The exposure window is specified in D3*]	N	
3.4 **If Y/PY to 3.3**: Were these characteristics likely to be influenced by exposure or a cause of exposure?	N	
3.5 **If Y/PY to 3.4**: Were these characteristics likely to be influenced by the outcome or a cause of the outcome?	PN	
3.6 **If N/PN to 3.2 or Y/PY to 3.5**: Is it likely that the analysis corrected for all of the potential selection biases identified in A and B above?	Y	
3.7 **If N/PN to 3.2 or Y/PY to 3.5**: Did sensitivity analyses demonstrate that the likely impact of the potential selection biases identified in A or B above was minimal?	PY	
Risk of bias (due to selection of participants into the study) in the estimated effect of exposure on the outcome	Low risk	
What is the predicted direction of bias due to selection of participants into the study?	Towards null	
Is the risk of bias (due to selection of participants into the study) sufficiently high, in the context of its likely direction and the magnitude of the estimated exposure effect, to threaten conclusions about whether the exposure has an important effect on the outcome?	No	

Y = Yes; PY = Probably yes; PN = Probably no; N = No; SN = Strong no; WN = Weak no; NA = Not applicable; NI = No information

Domain 4: Risk of bias due to post-exposure interventions

Signalling questions	Response options	Comments
4.1 Were there post-exposure interventions that were influenced by prior exposure during the follow-up period?	N	
4.2 **If Y/PY to 4.1**: Is it likely that the analysis corrected for the effect of post-exposure interventions that were influenced by prior exposure?	NA	
Risk of bias (due post-exposure interventions) in the estimated effect of exposure on the outcome	Low risk	
What is the predicted direction of bias due to confounding?	Towards null	
Is the risk of bias (due post-exposure interventions) sufficiently high, in the context of its likely direction and the magnitude of the estimated exposure effect, to threaten conclusions about whether the exposure has an important effect on the outcome?	No	

Y = Yes; PY = Probably yes; PN = Probably no; N = No; NA = Not applicable; NI = No information

Domain 5: Risk of bias due to missing data

Signalling questions	Response options	Comments
5.1 Were complete data on exposure status available for all, or nearly all, participants?	Y	
5.2 Were complete data on the outcome available for all, or nearly all, participants?	Y	
5.3 Were complete data on confounding variables available for all, or nearly all, participants?	Y	
5.4 **If N/PN/NI to 5.1, 5.2 or 5.3**: Is the result based on a complete case analysis?	Y	
5.5 **If Y/PY/NI**: Was exclusion from the analysis because of missing data (in exposure, confounders or the outcome) likely to be related to the true value of the outcome?	N	
5.6 **If N/PN to 5.5**: Were all or most predictors of missingness (in exposure, confounders or the outcome) included in the analysis model?	SY	
5.7 **If N/PN to 5.4**: Was the analysis based on imputing missing values?	Y	
5.8 **If Y/PY to 5.7**: Was imputation performed appropriately?	Y	
5.9 **If N/PN to 5.7**: Was an appropriate alternative method used to correct for bias due to missing data?	Y	
5.10 **If PN/N/NI to 5.1, 5.2 or 5.3**: Is there evidence that the result was not biased by missing data?	Y	
Risk of bias (due to missing data) in the estimated effect of exposure on the outcome	Low risk	
What is the predicted direction of bias due to missing data?	Towards null	
Is the risk of bias (due to missing data) sufficiently high, in the context of its likely direction and the magnitude of the estimated exposure effect, to threaten conclusions about whether the exposure has an important effect on the outcome?	No	

Y = Yes; PY = Probably yes; PN = Probably no; N = No; SY = Strong yes; WY = Weak yes; NA = Not applicable; NI = No information

Domain 6: Risk of bias arising from measurement of the outcome

Signalling questions	Response options	Comments
6.1 Could measurement or ascertainment of the outcome have differed between exposure groups or levels of exposure?	N	
6.2 Were outcome assessors aware of study participants' exposure history?	N	
6.3 **If Y/PY/NI to 6.2**: Could assessment of the outcome have been influenced by knowledge of participants' exposure history?	N	
Risk of bias (arising from measurement of outcomes) in the estimated effect of exposure on the outcome	Low risk	
What is the predicted direction of bias arising from measurement of outcomes?	Towards null	
Is the risk of bias (arising from measurement of outcomes) sufficiently high, in the context of its likely direction and the magnitude of the estimated exposure effect, to threaten conclusions about whether the exposure has an important effect on the outcome?	No	

Y = Yes; PY = Probably yes; PN = Probably no; N = No; SY = Strong yes; WY = Weak yes; NA = Not applicable; NI = No information

Domain 7: Risk of bias in selection of the reported result

Signalling questions	Response options	Comments
7.1 Was the result reported in accordance with an available, pre-determined analysis plan?	Y	
7.2 **If N/PN/NI to 7.1**: Is the reported effect estimate likely to be selected, based on desirability of the magnitude (or statistical significance) of the estimated effect of exposure on outcome, from multiple *exposure measurements* within the exposure domain?	N	
7.3 Is the reported effect estimate likely to be selected, based on desirability of the magnitude (or statistical significance) of the estimated effect of exposure on outcome, from multiple *outcome measurements* within the outcome domain?	N	
7.4 Is the reported effect estimate likely to be selected, based on desirability of the magnitude (or statistical significance) of the estimated effect of exposure on outcome, from multiple *analyses* of the exposure-outcome relationship?	N	
7.5 Is the reported effect estimate likely to be selected, based on the basis of desirability of the results (e.g. statistical significance), from different *subgroups*?	N	
Risk of bias (due to selection of the reported result) in the estimated effect of exposure on the outcome	Low risk	
What is the predicted direction of bias due to selection of the reported result?	Towards null	
Is the risk of bias (due to selection of the reported result) sufficiently high, in the context of its likely direction and the magnitude of the estimated exposure effect, to threaten conclusions about whether the exposure has an important effect on the outcome?	No	

Y = Yes; PY = Probably yes; PN = Probably no; N = No; NA = Not applicable; NI = No information

Overall risk of bias

	Response options	Comments
Overall risk of bias	Low risk of bias	
What is the predicted direction of bias?	Towards null	
Is the overall risk of bias sufficiently high, in the context of its likely direction and the magnitude of the estimated exposure effect, to threaten conclusions about whether the exposure has an important effect on the outcome?	No	

[48] Hálfdánarson, Ó.; Cohen, J.M.; Karlstad, Ø.; Cesta, C.E.; Bjørk, M.H.; Håberg, S.E.; Einarsdóttir, K.; Furu, K.; Gissler, M.; Hjellvik, V.; Kieler, H.; Leinonen, M.K.; Nørgaard, M.; Öztürk, Essen, B.; Ulrichsen, S.P.; Reutfors, J.; Zoega, H. Antipsychotic use in pregnancy and risk of attention/deficit-hyperactivity disorder and autism spectrum disorder: a Nordic cohort study. *Evid. Based Ment. Health* **2022**, *25*(2), 54-62. doi: 10.1136/ebmental-2021-300311. Epub 2021 Nov 22.

Domain 1: Risk of bias due to confounding *variant (b): **If Y/PY to C7 and Y/PY to C8** (the analysis was based on splitting participants' follow up time according to exposure status and/or magnitude and changes in exposure status and/or magnitude likely to be related to factors that are predictive of the outcome, so both baseline and time-varying confounding need to be addressed)*

Signalling questions	Response options	Comments
1.1 Did the authors use an analysis method that was appropriate to control for time-varying as well as baseline confounding?	Y	
1.2 **If Y/PY to 1.1**: Did the authors control for all the important baseline and time-varying confounding factors for which this was necessary?	Y	
1.3 **If Y/PY/WN to 1.2**: Were confounding factors that were controlled for (and for which control was necessary) measured validly and reliably by the variables available in this study?	Y	
1.4 **If N/PN/NI to 1.1**: Did the authors control for time-varying factors or other variables measured after the start of the exposure window being studied?	N	
1.5 Did the use of negative controls, or other considerations, suggest uncontrolled confounding?	N	
Risk of bias (due to confounding) in the estimated effect of exposure on the outcome	Low risk	
What is the predicted direction of bias due to confounding?	Towards null	
Is the risk of bias (due to confounding) sufficiently high, in the context of its likely direction and the magnitude of the estimated exposure effect, to threaten conclusions about whether the exposure has an important effect on the outcome?	No	

Y = Yes; PY = Probably yes; PN = Probably no; N = No; SY = Strong yes; WY = Weak yes; SN = Strong no; WN = Weak no; NA = Not applicable; NI = No information

Domain 2: Risk of bias arising from measurement of the exposure *Variant (b): **If Y/PY to C5 and Y/PY to C6** (each individual's exposure level was estimated from measurements made at multiple time points)*

Signalling questions	Response options	Comments
**2.1 Does the measured exposure (derived from measurements at multiple time points) well-characterize the exposure metric specified to be of interest in this study? [*This was specified in the answers to D2, D3 and D4*]**	**Y**	
**2.2 Was there error in measurement, or misclassification, of the exposure, at each single time point?**	**Y**	
**2.3 If SY/WY to 2.2: Could mismeasurement or misclassification of exposure have been differential (i.e. related to the outcome or risk of the outcome)?**	**N**	
**2.4 If SY/WY to 2.2 and N/PN/WY to 2.3: Is the nature of the (non-differential) measurement error likely to bias the estimated effect of exposure on outcome?**	**N**	
**Risk of bias (arising from measurement of exposure) in the estimated effect of exposure on the outcome**	**PN**	
**What is the predicted direction of bias arising from measurement of exposure?**	**Low risk**	
**Is the risk of bias (arising from measurement of exposure) sufficiently high, in the context of its likely direction and the magnitude of the estimated exposure effect, to threaten conclusions about whether the exposure has an important effect on the outcome?**	**Towards null**	

Y = Yes; PY = Probably yes; SN = Strong no; WN = Weak no; NA = Not applicable; NI = No information

Domain 3: Risk of bias in selection of participants into the study (or into the analysis)

Signalling questions	Response options	Comments
3.1 Did follow-up begin at (or close to) the start of the exposure window for most participants? [*The exposure window is specified in D3*]	Y	
3.2 **If N/PN to 3.1**: Is the effect of exposure likely to be constant over the period of follow up analysed?	Y	
3.3 Was selection of participants into the study (or into the analysis) based on participant characteristics observed after the start of the exposure window being studied? [*The exposure window is specified in D3*]	N	
3.4 **If Y/PY to 3.3**: Were these characteristics likely to be influenced by exposure or a cause of exposure?	N	
3.5 **If Y/PY to 3.4**: Were these characteristics likely to be influenced by the outcome or a cause of the outcome?	PN	
3.6 **If N/PN to 3.2 or Y/PY to 3.5**: Is it likely that the analysis corrected for all of the potential selection biases identified in A and B above?	Y	
3.7 **If N/PN to 3.2 or Y/PY to 3.5**: Did sensitivity analyses demonstrate that the likely impact of the potential selection biases identified in A or B above was minimal?	PY	
Risk of bias (due to selection of participants into the study) in the estimated effect of exposure on the outcome	Low risk	
What is the predicted direction of bias due to selection of participants into the study?	Towards null	
Is the risk of bias (due to selection of participants into the study) sufficiently high, in the context of its likely direction and the magnitude of the estimated exposure effect, to threaten conclusions about whether the exposure has an important effect on the outcome?	No	

Y = Yes; PY = Probably yes; PN = Probably no; N = No; SN = Strong no; WN = Weak no; NA = Not applicable; NI = No information

Domain 4: Risk of bias due to post-exposure interventions

Signalling questions	Response options	Comments
4.1 Were there post-exposure interventions that were influenced by prior exposure during the follow-up period?	N	
4.2 **If Y/PY to 4.1**: Is it likely that the analysis corrected for the effect of post-exposure interventions that were influenced	NA	

by prior exposure?		
Risk of bias (due post-exposure interventions) in the estimated effect of exposure on the outcome	Low risk	
What is the predicted direction of bias due to confounding?	Towards null	
Is the risk of bias (due post-exposure interventions) sufficiently high, in the context of its likely direction and the magnitude of the estimated exposure effect, to threaten conclusions about whether the exposure has an important effect on the outcome?	No	

Y = Yes; PY = Probably yes; PN = Probably no; N = No; NA = Not applicable; NI = No information

Domain 5: Risk of bias due to missing data

Signalling questions	Response options	Comments
5.1 Were complete data on exposure status available for all, or nearly all, participants?	Y	
5.2 Were complete data on the outcome available for all, or nearly all, participants?	Y	
5.3 Were complete data on confounding variables available for all, or nearly all, participants?	Y	
5.4 **If N/PN/NI to 5.1, 5.2 or 5.3**: Is the result based on a complete case analysis?	Y	
5.5 **If Y/PY/NI**: Was exclusion from the analysis because of missing data (in exposure, confounders or the outcome) likely to be related to the true value of the outcome?	N	
5.6 **If N/PN to 5.5**: Were all or most predictors of missingness (in exposure, confounders or the outcome) included in the analysis model?	SY	
5.7 **If N/PN to 5.4**: Was the analysis based on imputing missing values?	Y	
5.8 **If Y/PY to 5.7**: Was imputation performed appropriately?	Y	
5.9 **If N/PN to 5.7**: Was an appropriate alternative method used to correct for bias due to missing data?	Y	
5.10 **If PN/N/NI to 5.1, 5.2 or 5.3**: Is there evidence that the result was not biased by missing data?	Y	
Risk of bias (due to missing data) in the estimated effect of exposure on the outcome	Low risk	
What is the predicted direction of bias due to missing data?	Towards null	
Is the risk of bias (due to missing data) sufficiently high, in the context of its likely direction and the magnitude of the estimated exposure effect, to threaten conclusions about whether the exposure has an important effect on the outcome?	No	

Y = Yes; PY = Probably yes; PN = Probably no; N = No; SY = Strong yes; WY = Weak yes; NA = Not applicable; NI = No information

Domain 6: Risk of bias arising from measurement of the outcome

Signalling questions	Response options	Comments
6.1 Could measurement or ascertainment of the outcome have differed between exposure groups or levels of exposure?	N	
6.2 Were outcome assessors aware of study participants' exposure history?	N	
6.3 **If Y/PY/NI to 6.2**: Could assessment of the outcome have been influenced by knowledge of participants' exposure history?	N	
Risk of bias (arising from measurement of outcomes) in the estimated effect of exposure on the outcome	Low risk	
What is the predicted direction of bias arising from measurement of outcomes?	Towards null	
Is the risk of bias (arising from measurement of outcomes) sufficiently high, in the context of its likely direction and the magnitude of the estimated exposure effect, to threaten conclusions about whether the exposure has an important effect on the outcome?	No	

Y = Yes; PY = Probably yes; PN = Probably no; N = No; SY = Strong yes; WY = Weak yes; NA = Not applicable; NI = No information

Domain 7: Risk of bias in selection of the reported result

Signalling questions	Response options	Comments
7.1 Was the result reported in accordance with an available, pre-determined analysis plan?	Y	
7.2 **If N/PN/NI to 7.1**: Is the reported effect estimate likely to be selected, based on desirability of the magnitude (or statistical significance) of the estimated effect of exposure on outcome, from multiple *exposure measurements* within the exposure domain?	N	
7.3 Is the reported effect estimate likely to be selected, based on desirability of the magnitude (or statistical significance) of the estimated effect of exposure on outcome, from multiple *outcome measurements* within the outcome domain?	N	
7.4 Is the reported effect estimate likely to be selected, based on desirability of the magnitude (or statistical significance) of the estimated effect of exposure on outcome, from multiple *analyses* of the exposure-outcome relationship?	N	
7.5 Is the reported effect estimate likely to be selected, based on the basis of desirability of the results (e.g. statistical significance), from different *subgroups*?	N	
Risk of bias (due to selection of the reported result) in the estimated effect of exposure on the outcome	Low risk	
What is the predicted direction of bias due to selection of the reported result?	Towards null	
Is the risk of bias (due to selection of the reported result) sufficiently high, in the context of its likely direction and the magnitude of the estimated exposure effect, to threaten conclusions about whether the exposure has an important effect on the outcome?	No	

Y = Yes; PY = Probably yes; PN = Probably no; N = No; NA = Not applicable; NI = No information

Overall risk of bias

	Response options	Comments
Overall risk of bias	Low risk of bias	
What is the predicted direction of bias?	Towards null	
Is the overall risk of bias sufficiently high, in the context of its likely direction and the magnitude of the estimated exposure effect, to threaten conclusions about whether the exposure has an important effect on the outcome?	No	

[49] Straub, L.; Hernández-Díaz, S.; Bateman, B.T.; Wisner, K.L.; Gray, K.J.; Pennell, P.B.; Lester, B.; McDougle, C.J.; Suarez, E.A.; Zhu, Y.; Zakoul, H.; Mogun, H.; Huybrechts, K.F. Association of antipsychotic drug exposure in pregnancy with risk of neurodevelopmental disorders: A National Birth Cohort Study. *JAMA Intern. Med.* **2022**, *182*(5), 522-533. doi: 10.1001/jamainternmed.2022.0375.

Domain 1: Risk of bias due to confounding *variant (b): **If Y/PY to C7 and Y/PY to C8** (the analysis was based on splitting participants' follow up time according to exposure status and/or magnitude and changes in exposure status and/or magnitude likely to be related to factors that are predictive of the outcome, so both baseline and time-varying confounding need to be addressed)*

Signalling questions	Response options	Comments
1.1 Did the authors use an analysis method that was appropriate to control for time-varying as well as baseline confounding?	Y	
1.2 **If Y/PY to 1.1**: Did the authors control for all the important baseline and time-varying confounding factors for which this was necessary?	Y	
1.3 **If Y/PY/WN to 1.2**: Were confounding factors that were controlled for (and for which control was necessary) measured validly and reliably by the variables available in this study?	Y	
1.4 **If N/PN/NI to 1.1**: Did the authors control for time-varying factors or other variables measured after the start of the exposure window being studied?	N	
1.5 Did the use of negative controls, or other considerations, suggest uncontrolled confounding?	N	
Risk of bias (due to confounding) in the estimated effect of exposure on the outcome	Low risk	
What is the predicted direction of bias due to confounding?	Towards null	
Is the risk of bias (due to confounding) sufficiently high, in the context of its likely direction and the magnitude of the estimated exposure effect, to threaten conclusions about whether the exposure has an important effect on the outcome?	No	

Y = Yes; PY = Probably yes; PN = Probably no; N = No; SY = Strong yes; WY = Weak yes; SN = Strong no; WN = Weak no; NA = Not applicable; NI = No information

Domain 2: Risk of bias arising from measurement of the exposure *Variant (b): **If Y/PY to C5 and Y/PY to C6** (each individual's exposure level was estimated from measurements made at multiple time points)*

Signalling questions	Response options	Comments
**2.1 Does the measured exposure (derived from measurements at multiple time points) well-characterize the exposure metric specified to be of interest in this study? [*This was specified in the answers to D2, D3 and D4*]**	**Y**	
**2.2 Was there error in measurement, or misclassification, of the exposure, at each single time point?**	**Y**	
**2.3 If SY/WY to 2.2: Could mismeasurement or misclassification of exposure have been differential (i.e. related to the outcome or risk of the outcome)?**	**N**	
**2.4 If SY/WY to 2.2 and N/PN/WY to 2.3: Is the nature of the (non-differential) measurement error likely to bias the estimated effect of exposure on outcome?**	**N**	
**Risk of bias (arising from measurement of exposure) in the estimated effect of exposure on the outcome**	**PN**	
**What is the predicted direction of bias arising from measurement of exposure?**	**Low risk**	
**Is the risk of bias (arising from measurement of exposure) sufficiently high, in the context of its likely direction and the magnitude of the estimated exposure effect, to threaten conclusions about whether the exposure has an important effect on the outcome?**	**Towards null**	

Y = Yes; PY = Probably yes; SN = Strong no; WN = Weak no; NA = Not applicable; NI = No information

Domain 3: Risk of bias in selection of participants into the study (or into the analysis)

Signalling questions	Response options	Comments
3.1 Did follow-up begin at (or close to) the start of the exposure window for most participants? [*The exposure window is specified in D3*]	Y	
3.2 **If N/PN to 3.1**: Is the effect of exposure likely to be constant over the period of follow up analysed?	Y	
3.3 Was selection of participants into the study (or into the analysis) based on participant characteristics observed after the start of the exposure window being studied? [*The exposure window is specified in D3*]	N	
3.4 **If Y/PY to 3.3**: Were these characteristics likely to be influenced by exposure or a cause of exposure?	N	
3.5 **If Y/PY to 3.4**: Were these characteristics likely to be influenced by the outcome or a cause of the outcome?	PN	
3.6 **If N/PN to 3.2 or Y/PY to 3.5**: Is it likely that the analysis corrected for all of the potential selection biases identified in A and B above?	Y	

Signalling questions	Response options	Comments
3.7 **If N/PN to 3.2 or Y/PY to 3.5**: Did sensitivity analyses demonstrate that the likely impact of the potential selection biases identified in A or B above was minimal?	PY	
Risk of bias (due to selection of participants into the study) in the estimated effect of exposure on the outcome	Low risk	
What is the predicted direction of bias due to selection of participants into the study?	Towards null	
Is the risk of bias (due to selection of participants into the study) sufficiently high, in the context of its likely direction and the magnitude of the estimated exposure effect, to threaten conclusions about whether the exposure has an important effect on the outcome?	No	

Y = Yes; PY = Probably yes; PN = Probably no; N = No; SN = Strong no; WN = Weak no; NA = Not applicable; NI = No information

Domain 4: Risk of bias due to post-exposure interventions

Signalling questions	Response options	Comments
4.1 Were there post-exposure interventions that were influenced by prior exposure during the follow-up period?	N	
4.2 **If Y/PY to 4.1**: Is it likely that the analysis corrected for the effect of post-exposure interventions that were influenced by prior exposure?	NA	
Risk of bias (due post-exposure interventions) in the estimated effect of exposure on the outcome	Low risk	
What is the predicted direction of bias due to confounding?	Towards null	
Is the risk of bias (due post-exposure interventions) sufficiently high, in the context of its likely direction and the magnitude of the estimated exposure effect, to threaten conclusions about whether the exposure has an important effect on the outcome?	No	

Y = Yes; PY = Probably yes; PN = Probably no; N = No; NA = Not applicable; NI = No information

Domain 5: Risk of bias due to missing data

Signalling questions	Response options	Comments
5.1 Were complete data on exposure status available for all, or nearly all, participants?	Y	
5.2 Were complete data on the outcome available for all, or nearly all, participants?	Y	
5.3 Were complete data on confounding variables available for all, or nearly all, participants?	Y	
5.4 **If N/PN/NI to 5.1, 5.2 or 5.3**: Is the result based on a complete case analysis?	Y	
5.5 **If Y/PY/NI**: Was exclusion from the analysis because of missing data (in exposure, confounders or the outcome) likely to be related to the true value of the outcome?	N	
5.6 **If N/PN to 5.5**: Were all or most predictors of missingness (in exposure, confounders or the outcome) included in the analysis model?	SY	
5.7 **If N/PN to 5.4**: Was the analysis based on imputing missing values?	Y	
5.8 **If Y/PY to 5.7**: Was imputation performed appropriately?	Y	
5.9 **If N/PN to 5.7**: Was an appropriate alternative method used to correct for bias due to missing data?	Y	
5.10 **If PN/N/NI to 5.1, 5.2 or 5.3**: Is there evidence that the result was not biased by missing data?	Y	
Risk of bias (due to missing data) in the estimated effect of exposure on the outcome	Low risk	
What is the predicted direction of bias due to missing data?	Towards null	
Is the risk of bias (due to missing data) sufficiently high, in the context of its likely direction and the magnitude of the estimated exposure effect, to threaten conclusions about whether the exposure has an important effect on the outcome?	No	

Y = Yes; PY = Probably yes; PN = Probably no; N = No; SY = Strong yes; WY = Weak yes; NA = Not applicable; NI = No information

Domain 6: Risk of bias arising from measurement of the outcome

Signalling questions	Response options	Comments
6.1 Could measurement or ascertainment of the outcome have differed between exposure groups or levels of exposure?	N	
6.2 Were outcome assessors aware of study participants' exposure history?	N	
6.3 **If Y/PY/NI to 6.2**: Could assessment of the outcome have been influenced by knowledge of participants' exposure history?	N	
Risk of bias (arising from measurement of outcomes) in the estimated effect of exposure on the outcome	Low risk	
What is the predicted direction of bias arising from measurement of outcomes?	Towards null	
Is the risk of bias (arising from measurement of outcomes) sufficiently high, in the context of its likely direction and the magnitude of the estimated exposure effect, to threaten conclusions about whether the exposure has an important effect on the outcome?	No	

Y = Yes; PY = Probably yes; PN = Probably no; N = No; SY = Strong yes; WY = Weak yes; NA = Not applicable; NI = No information

Domain 7: Risk of bias in selection of the reported result

Signalling questions	Response options	Comments
7.1 Was the result reported in accordance with an available, pre-determined analysis plan?	Y	
7.2 **If N/PN/NI to 7.1**: Is the reported effect estimate likely to be selected, based on desirability of the magnitude (or statistical significance) of the estimated effect of exposure on outcome, from multiple *exposure measurements* within the exposure domain?	N	
7.3 Is the reported effect estimate likely to be selected, based on desirability of the magnitude (or statistical significance)	N	

Signalling questions	Response options	Comments
of the estimated effect of exposure on outcome, from multiple *outcome measurements* within the outcome domain?		
7.4 Is the reported effect estimate likely to be selected, based on desirability of the magnitude (or statistical significance) of the estimated effect of exposure on outcome, from multiple *analyses* of the exposure-outcome relationship?	N	
7.5 Is the reported effect estimate likely to be selected, based on the basis of desirability of the results (e.g. statistical significance), from different *subgroups*?	N	
Risk of bias (due to selection of the reported result) in the estimated effect of exposure on the outcome	Low risk	
What is the predicted direction of bias due to selection of the reported result?	Towards null	
Is the risk of bias (due to selection of the reported result) sufficiently high, in the context of its likely direction and the magnitude of the estimated exposure effect, to threaten conclusions about whether the exposure has an important effect on the outcome?	No	

Y = Yes; PY = Probably yes; PN = Probably no; N = No; NA = Not applicable; NI = No information

Overall risk of bias

	Response options	Comments
Overall risk of bias	Low risk of bias	
What is the predicted direction of bias?	Towards null	
Is the overall risk of bias sufficiently high, in the context of its likely direction and the magnitude of the estimated exposure effect, to threaten conclusions about whether the exposure has an important effect on the outcome?	No	

[50] Huybrechts, K.F.; Straub, L.; Karlsson, P.; Pazzagli, L.; Furu, K.; Gissler, M.; Hernandez-Diaz, S.; Nørgaard, M.; Zoega, H.; Bateman, B.T.; Cesta, C.E.; Cohen, J.M.; Leinonen, M.K.; Reutfors, J.; Selmer, R.M.; Suarez, E.A.; Ulrichsen, S.P.; Kieler, H. Association of in utero antipsychotic medication exposure with risk of congenital malformations in Nordic countries and the US. *JAMA Psychiatry* **2023**, *80*(2), 156-166. doi: 10.1001/jamapsychiatry.2022.4109.

Domain 1: Risk of bias due to confounding *variant (b): **If Y/PY to C7 and Y/PY to C8** (the analysis was based on splitting participants' follow up time according to exposure status and/or magnitude and changes in exposure status and/or magnitude likely to be related to factors that are predictive of the outcome, so both baseline and time-varying confounding need to be addressed)*

Signalling questions	Response options	Comments
1.1 Did the authors use an analysis method that was appropriate to control for time-varying as well as baseline confounding?	Y	
1.2 **If Y/PY to 1.1**: Did the authors control for all the important baseline and time-varying confounding factors for which this was necessary?	Y	
1.3 **If Y/PY/WN to 1.2**: Were confounding factors that were controlled for (and for which control was necessary) measured validly and reliably by the variables available in this study?	Y	
1.4 **If N/PN/NI to 1.1**: Did the authors control for time-varying factors or other variables measured after the start of the exposure window being studied?	N	
1.5 Did the use of negative controls, or other considerations, suggest uncontrolled confounding?	N	
Risk of bias (due to confounding) in the estimated effect of exposure on the outcome	Low risk	
What is the predicted direction of bias due to confounding?	Towards null	
Is the risk of bias (due to confounding) sufficiently high, in the context of its likely direction and the magnitude of the estimated exposure effect, to threaten conclusions about whether the exposure has an important effect on the outcome?	No	

Y = Yes; PY = Probably yes; PN = Probably no; N = No; SY = Strong yes; WY = Weak yes; SN = Strong no; WN = Weak no; NA = Not applicable; NI = No information

***Domain 2: Risk of bias arising from measurement of the exposure** Variant (b): **If Y/PY to C5 and Y/PY to C6** (each individual's exposure level was estimated from measurements made at multiple time points)*

Signalling questions	Response options	Comments
**2.1 Does the measured exposure (derived from measurements at multiple time points) well-characterize the exposure metric specified to be of interest in this study? [*This was specified in the answers to D2, D3 and D4*]**	**Y**	
**2.2 Was there error in measurement, or misclassification, of the exposure, at each single time point?**	**Y**	
**2.3 If SY/WY to 2.2: Could mismeasurement or misclassification of exposure have been differential (i.e. related to the outcome or risk of the outcome)?**	**N**	
**2.4 If SY/WY to 2.2 and N/PN/WY to 2.3: Is the nature of the (non-differential) measurement error likely to bias the estimated effect of exposure on outcome?**	**N**	
**Risk of bias (arising from measurement of exposure) in the estimated effect of exposure on the outcome**	**PN**	
**What is the predicted direction of bias arising from measurement of exposure?**	**Low risk**	
**Is the risk of bias (arising from measurement of exposure) sufficiently high, in the context of its likely direction and the magnitude of the estimated exposure effect, to threaten conclusions about whether the exposure has an important effect on the outcome?**	**Towards null**	

Y = Yes; PY = Probably yes; SN = Strong no; WN = Weak no; NA = Not applicable; NI = No information

Domain 3: Risk of bias in selection of participants into the study (or into the analysis)

Signalling questions	Response options	Comments
3.1 Did follow-up begin at (or close to) the start of the exposure window for most participants? [*The exposure window is specified in D3*]	Y	
3.2 **If N/PN to 3.1**: Is the effect of exposure likely to be constant over the period of follow up analysed?	Y	
3.3 Was selection of participants into the study (or into the analysis) based on participant characteristics observed after the start of the exposure window being studied? [*The exposure window is specified in D3*]	N	
3.4 **If Y/PY to 3.3**: Were these characteristics likely to be influenced by exposure or a cause of exposure?	N	
3.5 **If Y/PY to 3.4**: Were these characteristics likely to be influenced by the outcome or a cause of the outcome?	PN	
3.6 **If N/PN to 3.2 or Y/PY to 3.5**: Is it likely that the analysis corrected for all of the potential selection biases identified in A and B above?	Y	
3.7 **If N/PN to 3.2 or Y/PY to 3.5**: Did sensitivity analyses demonstrate that the likely impact of the potential selection biases identified in A or B above was minimal?	PY	
Risk of bias (due to selection of participants into the study) in the estimated effect of exposure on the outcome	Low risk	
What is the predicted direction of bias due to selection of participants into the study?	Towards null	
Is the risk of bias (due to selection of participants into the study) sufficiently high, in the context of its likely direction and the magnitude of the estimated exposure effect, to threaten conclusions about whether the exposure has an important effect on the outcome?	No	

Y = Yes; PY = Probably yes; PN = Probably no; N = No; SN = Strong no; WN = Weak no; NA = Not applicable; NI = No information

Domain 4: Risk of bias due to post-exposure interventions

Signalling questions	Response options	Comments
4.1 Were there post-exposure interventions that were influenced by prior exposure during the follow-up period?	N	
4.2 **If Y/PY to 4.1**: Is it likely that the analysis corrected for the effect of post-exposure interventions that were influenced by prior exposure?	NA	
Risk of bias (due post-exposure interventions) in the estimated effect of exposure on the outcome	Low risk	
What is the predicted direction of bias due to confounding?	Towards null	
Is the risk of bias (due post-exposure interventions) sufficiently high, in the context of its likely direction and the magnitude of the estimated exposure effect, to threaten conclusions about whether the exposure has an important effect on the outcome?	No	

Y = Yes; PY = Probably yes; PN = Probably no; N = No; NA = Not applicable; NI = No information

Domain 5: Risk of bias due to missing data

Signalling questions	Response options	Comments
5.1 Were complete data on exposure status available for all, or nearly all, participants?	Y	
5.2 Were complete data on the outcome available for all, or nearly all, participants?	Y	
5.3 Were complete data on confounding variables available for all, or nearly all, participants?	Y	
5.4 **If N/PN/NI to 5.1, 5.2 or 5.3**: Is the result based on a complete case analysis?	Y	
5.5 **If Y/PY/NI**: Was exclusion from the analysis because of missing data (in exposure, confounders or the outcome) likely to be related to the true value of the outcome?	N	
5.6 **If N/PN to 5.5**: Were all or most predictors of missingness (in exposure, confounders or the outcome) included in the analysis model?	SY	
5.7 **If N/PN to 5.4**: Was the analysis based on imputing missing values?	Y	
5.8 **If Y/PY to 5.7**: Was imputation performed appropriately?	Y	
5.9 **If N/PN to 5.7**: Was an appropriate alternative method used to correct for bias due to missing data?	Y	
5.10 **If PN/N/NI to 5.1, 5.2 or 5.3**: Is there evidence that the result was not biased by missing data?	Y	
Risk of bias (due to missing data) in the estimated effect of exposure on the outcome	Low risk	
What is the predicted direction of bias due to missing data?	Towards null	
Is the risk of bias (due to missing data) sufficiently high, in the context of its likely direction and the magnitude of the estimated exposure effect, to threaten conclusions about whether the exposure has an important effect on the outcome?	No	

Y = Yes; PY = Probably yes; PN = Probably no; N = No; SY = Strong yes; WY = Weak yes; NA = Not applicable; NI = No information

Domain 6: Risk of bias arising from measurement of the outcome

Signalling questions	Response options	Comments
6.1 Could measurement or ascertainment of the outcome have differed between exposure groups or levels of exposure?	N	
6.2 Were outcome assessors aware of study participants' exposure history?	N	
6.3 **If Y/PY/NI to 6.2**: Could assessment of the outcome have been influenced by knowledge of participants' exposure history?	N	
Risk of bias (arising from measurement of outcomes) in the estimated effect of exposure on the outcome	Low risk	
What is the predicted direction of bias arising from measurement of outcomes?	Towards null	
Is the risk of bias (arising from measurement of outcomes) sufficiently high, in the context of its likely direction and the magnitude of the estimated exposure effect, to threaten conclusions about whether the exposure has an important effect on the outcome?	No	

Y = Yes; PY = Probably yes; PN = Probably no; N = No; SY = Strong yes; WY = Weak yes; NA = Not applicable; NI = No information

Domain 7: Risk of bias in selection of the reported result

Signalling questions	Response options	Comments
7.1 Was the result reported in accordance with an available, pre-determined analysis plan?	Y	
7.2 **If N/PN/NI to 7.1**: Is the reported effect estimate likely to be selected, based on desirability of the magnitude (or statistical significance) of the estimated effect of exposure on outcome, from multiple *exposure measurements* within the exposure domain?	N	
7.3 Is the reported effect estimate likely to be selected, based on desirability of the magnitude (or statistical significance) of the estimated effect of exposure on outcome, from multiple *outcome measurements* within the outcome domain?	N	
7.4 Is the reported effect estimate likely to be selected, based on desirability of the magnitude (or statistical significance) of the estimated effect of exposure on outcome, from multiple *analyses* of the exposure-outcome relationship?	N	
7.5 Is the reported effect estimate likely to be selected, based on the basis of desirability of the results (e.g. statistical significance), from different *subgroups*?	N	
Risk of bias (due to selection of the reported result) in the estimated effect of exposure on the outcome	Low risk	
What is the predicted direction of bias due to selection of the reported result?	Towards null	
Is the risk of bias (due to selection of the reported result) sufficiently high, in the context of its likely direction and the magnitude of the estimated exposure effect, to threaten conclusions about whether the exposure has an important effect on the outcome?	No	

Y = Yes; PY = Probably yes; PN = Probably no; N = No; NA = Not applicable; NI = No information

Overall risk of bias

	Response options	Comments
Overall risk of bias	Low risk of bias	
What is the predicted direction of bias?	Towards null	
Is the overall risk of bias sufficiently high, in the context of its likely direction and the magnitude of the estimated exposure effect, to threaten conclusions about whether the exposure has an important effect on the outcome?	No	

[51] Cohen, L.S.; Church, T.R.; Freeman, M.P.; Gaccione, P.; Caplin, P.S.; Kobylski, L.A.; Arakelian, M.; Rossa, E.T.; Chitayat, D.; Hernández-Díaz, S.; Viguera, A.C. Reproductive safety of lurasidone and quetiapine: Update from the National Pregnancy Registry for Psychiatric Medications. *J. Womens Health (Larchmt.)* **2023**, *32*(4), 452-462. doi: 10.1089/jwh.2022.0310.

Domain 1: Risk of bias due to confounding *variant (b): **If Y/PY to C7 and Y/PY to C8** (the analysis was based on splitting participants' follow up time according to exposure status and/or magnitude and changes in exposure status and/or magnitude likely to be related to factors that are predictive of the outcome, so both baseline and time-varying confounding need to be addressed)*

Signalling questions	Response options	Comments
1.1 Did the authors use an analysis method that was appropriate to control for time-varying as well as baseline confounding?	Y	
1.2 **If Y/PY to 1.1**: Did the authors control for all the important baseline and time-varying confounding factors for which this was necessary?	Y	
1.3 **If Y/PY/WN to 1.2**: Were confounding factors that were controlled for (and for which control was necessary) measured validly and reliably by the variables available in this study?	Y	
1.4 **If N/PN/NI to 1.1**: Did the authors control for time-varying factors or other variables measured after the start of the exposure window being studied?	N	
1.5 Did the use of negative controls, or other considerations, suggest uncontrolled confounding?	N	
Risk of bias (due to confounding) in the estimated effect of exposure on the outcome	Low risk	
What is the predicted direction of bias due to confounding?	Towards null	
Is the risk of bias (due to confounding) sufficiently high, in the context of its likely direction and the magnitude of the estimated exposure effect, to threaten conclusions about whether the exposure has an important effect on the outcome?	No	

Y = Yes; PY = Probably yes; PN = Probably no; N = No; SY = Strong yes; WY = Weak yes; SN = Strong no; WN = Weak no; NA = Not applicable; NI = No information

Domain 2: Risk of bias arising from measurement of the exposure *Variant (b): **If Y/PY to C5 and Y/PY to C6** (each individual's exposure level was estimated from measurements made at multiple time points)*

Signalling questions	Response options	Comments
**2.1 Does the measured exposure (derived from measurements at multiple time points) well-**	Y	

Signalling questions	Response options	Comments
**characterize the exposure metric specified to be of interest in this study? [*This was specified in the answers to D2, D3 and D4*]**		
**2.2 Was there error in measurement, or misclassification, of the exposure, at each single time point?**	**Y**	
**2.3 If SY/WY to 2.2: Could mismeasurement or misclassification of exposure have been differential (i.e. related to the outcome or risk of the outcome)?**	**N**	
**2.4 If SY/WY to 2.2 and N/PN/WY to 2.3: Is the nature of the (non-differential) measurement error likely to bias the estimated effect of exposure on outcome?**	**N**	
**Risk of bias (arising from measurement of exposure) in the estimated effect of exposure on the outcome**	**PN**	
**What is the predicted direction of bias arising from measurement of exposure?**	**Low risk**	
**Is the risk of bias (arising from measurement of exposure) sufficiently high, in the context of its likely direction and the magnitude of the estimated exposure effect, to threaten conclusions about whether the exposure has an important effect on the outcome?**	**Towards null**	

Y = Yes; PY = Probably yes; SN = Strong no; WN = Weak no; NA = Not applicable; NI = No information

Domain 3: Risk of bias in selection of participants into the study (or into the analysis)

Signalling questions	Response options	Comments
3.1 Did follow-up begin at (or close to) the start of the exposure window for most participants? [*The exposure window is specified in D3*]	Y	
3.2 **If N/PN to 3.1**: Is the effect of exposure likely to be constant over the period of follow up analysed?	Y	
3.3 Was selection of participants into the study (or into the analysis) based on participant characteristics observed after the start of the exposure window being studied? [*The exposure window is specified in D3*]	N	
3.4 **If Y/PY to 3.3**: Were these characteristics likely to be influenced by exposure or a cause of exposure?	N	
3.5 **If Y/PY to 3.4**: Were these characteristics likely to be influenced by the outcome or a cause of the outcome?	PN	
3.6 **If N/PN to 3.2 or Y/PY to 3.5**: Is it likely that the analysis corrected for all of the potential selection biases identified in A and B above?	Y	
3.7 **If N/PN to 3.2 or Y/PY to 3.5**: Did sensitivity analyses demonstrate that the likely impact of the potential selection biases identified in A or B above was minimal?	PY	
Risk of bias (due to selection of participants into the study) in the estimated effect of exposure on the outcome	Low risk	
What is the predicted direction of bias due to selection of participants into the study?	Towards null	
Is the risk of bias (due to selection of participants into the study) sufficiently high, in the context of its likely direction and the magnitude of the estimated exposure effect, to threaten conclusions about whether the exposure has an important effect on the outcome?	No	

Y = Yes; PY = Probably yes; PN = Probably no; N = No; SN = Strong no; WN = Weak no; NA = Not applicable; NI = No information

Domain 4: Risk of bias due to post-exposure interventions

Signalling questions	Response options	Comments
4.1 Were there post-exposure interventions that were influenced by prior exposure during the follow-up period?	N	
4.2 **If Y/PY to 4.1**: Is it likely that the analysis corrected for the effect of post-exposure interventions that were influenced by prior exposure?	NA	
Risk of bias (due post-exposure interventions) in the estimated effect of exposure on the outcome	Low risk	
What is the predicted direction of bias due to confounding?	Towards null	
Is the risk of bias (due post-exposure interventions) sufficiently high, in the context of its likely direction and the magnitude of the estimated exposure effect, to threaten conclusions about whether the exposure has an important effect on the outcome?	No	

Y = Yes; PY = Probably yes; PN = Probably no; N = No; NA = Not applicable; NI = No information

Domain 5: Risk of bias due to missing data

Signalling questions	Response options	Comments
5.1 Were complete data on exposure status available for all, or nearly all, participants?	Y	
5.2 Were complete data on the outcome available for all, or nearly all, participants?	Y	
5.3 Were complete data on confounding variables available for all, or nearly all, participants?	Y	
5.4 **If N/PN/NI to 5.1, 5.2 or 5.3**: Is the result based on a complete case analysis?	Y	
5.5 **If Y/PY/NI**: Was exclusion from the analysis because of missing data (in exposure, confounders or the outcome) likely to be related to the true value of the outcome?	N	
5.6 **If N/PN to 5.5**: Were all or most predictors of missingness (in exposure, confounders or the outcome) included in the analysis model?	SY	
5.7 **If N/PN to 5.4**: Was the analysis based on imputing missing values?	Y	
5.8 **If Y/PY to 5.7**: Was imputation performed appropriately?	Y	
5.9 **If N/PN to 5.7**: Was an appropriate alternative method used to correct for bias due to missing data?	Y	
5.10 **If PN/N/NI to 5.1, 5.2 or 5.3**: Is there evidence that the result was not biased by missing data?	Y	
Risk of bias (due to missing data) in the estimated effect of exposure on the outcome	Low risk	
What is the predicted direction of bias due to missing data?	Towards null	

Signalling questions	Response options	Comments
Is the risk of bias (due to missing data) sufficiently high, in the context of its likely direction and the magnitude of the estimated exposure effect, to threaten conclusions about whether the exposure has an important effect on the outcome?	No	

Y = Yes; PY = Probably yes; PN = Probably no; N = No; SY = Strong yes; WY = Weak yes; NA = Not applicable; NI = No information

Domain 6: Risk of bias arising from measurement of the outcome

Signalling questions	Response options	Comments
6.1 Could measurement or ascertainment of the outcome have differed between exposure groups or levels of exposure?	N	
6.2 Were outcome assessors aware of study participants' exposure history?	N	
6.3 **If Y/PY/NI to 6.2**: Could assessment of the outcome have been influenced by knowledge of participants' exposure history?	N	
Risk of bias (arising from measurement of outcomes) in the estimated effect of exposure on the outcome	Low risk	
What is the predicted direction of bias arising from measurement of outcomes?	Towards null	
Is the risk of bias (arising from measurement of outcomes) sufficiently high, in the context of its likely direction and the magnitude of the estimated exposure effect, to threaten conclusions about whether the exposure has an important effect on the outcome?	No	

Y = Yes; PY = Probably yes; PN = Probably no; N = No; SY = Strong yes; WY = Weak yes; NA = Not applicable; NI = No information

Domain 7: Risk of bias in selection of the reported result

Signalling questions	Response options	Comments
7.1 Was the result reported in accordance with an available, pre-determined analysis plan?	Y	
7.2 **If N/PN/NI to 7.1**: Is the reported effect estimate likely to be selected, based on desirability of the magnitude (or statistical significance) of the estimated effect of exposure on outcome, from multiple *exposure measurements* within the exposure domain?	N	
7.3 Is the reported effect estimate likely to be selected, based on desirability of the magnitude (or statistical significance) of the estimated effect of exposure on outcome, from multiple *outcome measurements* within the outcome domain?	N	
7.4 Is the reported effect estimate likely to be selected, based on desirability of the magnitude (or statistical significance) of the estimated effect of exposure on outcome, from multiple *analyses* of the exposure-outcome relationship?	N	
7.5 Is the reported effect estimate likely to be selected, based on the basis of desirability of the results (e.g. statistical significance), from different *subgroups*?	N	
Risk of bias (due to selection of the reported result) in the estimated effect of exposure on the outcome	Low risk	
What is the predicted direction of bias due to selection of the reported result?	Towards null	
Is the risk of bias (due to selection of the reported result) sufficiently high, in the context of its likely direction and the magnitude of the estimated exposure effect, to threaten conclusions about whether the exposure has an important effect on the outcome?	No	

Y = Yes; PY = Probably yes; PN = Probably no; N = No; NA = Not applicable; NI = No information

Overall risk of bias

	Response options	Comments
Overall risk of bias	Low risk of bias	
What is the predicted direction of bias?	Towards null	
Is the overall risk of bias sufficiently high, in the context of its likely direction and the magnitude of the estimated exposure effect, to threaten conclusions about whether the exposure has an important effect on the outcome?	No	

[52] Viguera, A.C.; Freeman, M.P.; Kobylski, L.A.; Rossa, E.T.; Gaccione, P.; Chitayat, D.; Hernández-Díaz, S.; Cohen, L.S. Risk of major malformations following first-trimester exposure to olanzapine: Preliminary data from the Massachusetts General Hospital National Pregnancy Registry for Psychiatric Medications. *J. Clin. Psychopharmacol.* **2023**, *43*(2), 106-112. doi: 10.1097/JCP.0000000000001665.

Domain 1: Risk of bias due to confounding *variant (b): **If Y/PY to C7 and Y/PY to C8** (the analysis was based on splitting participants' follow up time according to exposure status and/or magnitude and changes in exposure status and/or magnitude likely to be related to factors that are predictive of the outcome, so both baseline and time-varying confounding need to be addressed)*

Signalling questions	Response options	Comments
1.1 Did the authors use an analysis method that was appropriate to control for time-varying as well as baseline confounding?	Y	
1.2 **If Y/PY to 1.1**: Did the authors control for all the important baseline and time-varying confounding factors for which	Y	

Signalling questions	Response options	Comments
this was necessary?		
1.3 **If** Y/PY/WN **to 1.2**: Were confounding factors that were controlled for (and for which control was necessary) measured validly and reliably by the variables available in this study?	Y	
1.4 **If** N/PN/NI **to 1.1**: Did the authors control for time-varying factors or other variables measured after the start of the exposure window being studied?	N	
1.5 Did the use of negative controls, or other considerations, suggest uncontrolled confounding?	N	
Risk of bias (due to confounding) in the estimated effect of exposure on the outcome	Low risk	
What is the predicted direction of bias due to confounding?	Towards null	
Is the risk of bias (due to confounding) sufficiently high, in the context of its likely direction and the magnitude of the estimated exposure effect, to threaten conclusions about whether the exposure has an important effect on the outcome?	No	

Y = Yes; PY = Probably yes; PN = Probably no; N = No; SY = Strong yes; WY = Weak yes; SN = Strong no; WN = Weak no; NA = Not applicable; NI = No information

***Domain 2: Risk of bias arising from measurement of the exposure** Variant (b): **If Y/PY to C5 and Y/PY to C6** (each individual's exposure level was estimated from measurements made at multiple time points)*

Signalling questions	Response options	Comments
**2.1 Does the measured exposure (derived from measurements at multiple time points) well-characterize the exposure metric specified to be of interest in this study? [*This was specified in the answers to D2, D3 and D4*]**	**Y**	
**2.2 Was there error in measurement, or misclassification, of the exposure, at each single time point?**	**Y**	
**2.3 If SY/WY to 2.2: Could mismeasurement or misclassification of exposure have been differential (i.e. related to the outcome or risk of the outcome)?**	**N**	
**2.4 If SY/WY to 2.2 and N/PN/WY to 2.3: Is the nature of the (non-differential) measurement error likely to bias the estimated effect of exposure on outcome?**	**N**	
**Risk of bias (arising from measurement of exposure) in the estimated effect of exposure on the outcome**	**PN**	
**What is the predicted direction of bias arising from measurement of exposure?**	**Low risk**	
**Is the risk of bias (arising from measurement of exposure) sufficiently high, in the context of its likely direction and the magnitude of the estimated exposure effect, to threaten conclusions about whether the exposure has an important effect on the outcome?**	**Towards null**	

Y = Yes; PY = Probably yes; SN = Strong no; WN = Weak no; NA = Not applicable; NI = No information

Domain 3: Risk of bias in selection of participants into the study (or into the analysis)

Signalling questions	Response options	Comments
3.1 Did follow-up begin at (or close to) the start of the exposure window for most participants? [*The exposure window is specified in D3*]	Y	
3.2 **If** N/PN **to 3.1**: Is the effect of exposure likely to be constant over the period of follow up analysed?	Y	
3.3 Was selection of participants into the study (or into the analysis) based on participant characteristics observed after the start of the exposure window being studied? [*The exposure window is specified in D3*]	N	
3.4 **If** Y/PY **to 3.3**: Were these characteristics likely to be influenced by exposure or a cause of exposure?	N	
3.5 **If** Y/PY **to 3.4**: Were these characteristics likely to be influenced by the outcome or a cause of the outcome?	PN	
3.6 **If** N/PN **to 3.2 or** Y/PY **to 3.5**: Is it likely that the analysis corrected for all of the potential selection biases identified in A and B above?	Y	
3.7 **If** N/PN **to 3.2 or** Y/PY **to 3.5**: Did sensitivity analyses demonstrate that the likely impact of the potential selection biases identified in A or B above was minimal?	PY	
Risk of bias (due to selection of participants into the study) in the estimated effect of exposure on the outcome	Low risk	
What is the predicted direction of bias due to selection of participants into the study?	Towards null	
Is the risk of bias (due to selection of participants into the study) sufficiently high, in the context of its likely direction and the magnitude of the estimated exposure effect, to threaten conclusions about whether the exposure has an important effect on the outcome?	No	

Y = Yes; PY = Probably yes; PN = Probably no; N = No; SN = Strong no; WN = Weak no; NA = Not applicable; NI = No information

Domain 4: Risk of bias due to post-exposure interventions

Signalling questions	Response options	Comments
4.1 Were there post-exposure interventions that were influenced by prior exposure during the follow-up period?	N	
4.2 **If** Y/PY **to 4.1**: Is it likely that the analysis corrected for the effect of post-exposure interventions that were influenced by prior exposure?	NA	
Risk of bias (due post-exposure interventions) in the estimated effect of exposure on the outcome	Low risk	
What is the predicted direction of bias due to confounding?	Towards null	
Is the risk of bias (due post-exposure interventions) sufficiently high, in the context of its likely direction and the magnitude of the estimated exposure effect, to threaten conclusions about whether the exposure has an important effect on the outcome?	No	

Y = Yes; PY = Probably yes; PN = Probably no; N = No; NA = Not applicable; NI = No information

Domain 5: Risk of bias due to missing data

Signalling questions	Response options	Comments
5.1 Were complete data on exposure status available for all, or nearly all, participants?	Y	
5.2 Were complete data on the outcome available for all, or nearly all, participants?	Y	
5.3 Were complete data on confounding variables available for all, or nearly all, participants?	Y	
5.4 **If N/PN/NI to 5.1, 5.2 or 5.3**: Is the result based on a complete case analysis?	Y	
5.5 **If Y/PY/NI**: Was exclusion from the analysis because of missing data (in exposure, confounders or the outcome) likely to be related to the true value of the outcome?	N	
5.6 **If N/PN to 5.5**: Were all or most predictors of missingness (in exposure, confounders or the outcome) included in the analysis model?	SY	
5.7 **If N/PN to 5.4**: Was the analysis based on imputing missing values?	Y	
5.8 **If Y/PY to 5.7**: Was imputation performed appropriately?	Y	
5.9 **If N/PN to 5.7**: Was an appropriate alternative method used to correct for bias due to missing data?	Y	
5.10 **If PN/N/NI to 5.1, 5.2 or 5.3**: Is there evidence that the result was not biased by missing data?	Y	
Risk of bias (due to missing data) in the estimated effect of exposure on the outcome	Low risk	
What is the predicted direction of bias due to missing data?	Towards null	
Is the risk of bias (due to missing data) sufficiently high, in the context of its likely direction and the magnitude of the estimated exposure effect, to threaten conclusions about whether the exposure has an important effect on the outcome?	No	

Y = Yes; PY = Probably yes; PN = Probably no; N = No; SY = Strong yes; WY = Weak yes; NA = Not applicable; NI = No information

Domain 6: Risk of bias arising from measurement of the outcome

Signalling questions	Response options	Comments
6.1 Could measurement or ascertainment of the outcome have differed between exposure groups or levels of exposure?	N	
6.2 Were outcome assessors aware of study participants' exposure history?	N	
6.3 **If Y/PY/NI to 6.2**: Could assessment of the outcome have been influenced by knowledge of participants' exposure history?	N	
Risk of bias (arising from measurement of outcomes) in the estimated effect of exposure on the outcome	Low risk	
What is the predicted direction of bias arising from measurement of outcomes?	Towards null	
Is the risk of bias (arising from measurement of outcomes) sufficiently high, in the context of its likely direction and the magnitude of the estimated exposure effect, to threaten conclusions about whether the exposure has an important effect on the outcome?	No	

Y = Yes; PY = Probably yes; PN = Probably no; N = No; SY = Strong yes; WY = Weak yes; NA = Not applicable; NI = No information

Domain 7: Risk of bias in selection of the reported result

Signalling questions	Response options	Comments
7.1 Was the result reported in accordance with an available, pre-determined analysis plan?	Y	
7.2 **If N/PN/NI to 7.1**: Is the reported effect estimate likely to be selected, based on desirability of the magnitude (or statistical significance) of the estimated effect of exposure on outcome, from multiple *exposure measurements* within the exposure domain?	N	
7.3 Is the reported effect estimate likely to be selected, based on desirability of the magnitude (or statistical significance) of the estimated effect of exposure on outcome, from multiple *outcome measurements* within the outcome domain?	N	
7.4 Is the reported effect estimate likely to be selected, based on desirability of the magnitude (or statistical significance) of the estimated effect of exposure on outcome, from multiple *analyses* of the exposure-outcome relationship?	N	
7.5 Is the reported effect estimate likely to be selected, based on the basis of desirability of the results (e.g. statistical significance), from different *subgroups*?	N	
Risk of bias (due to selection of the reported result) in the estimated effect of exposure on the outcome	Low risk	
What is the predicted direction of bias due to selection of the reported result?	Towards null	
Is the risk of bias (due to selection of the reported result) sufficiently high, in the context of its likely direction and the magnitude of the estimated exposure effect, to threaten conclusions about whether the exposure has an important effect on the outcome?	No	

Y = Yes; PY = Probably yes; PN = Probably no; N = No; NA = Not applicable; NI = No information

Overall risk of bias

	Response options	Comments
Overall risk of bias	Low risk of bias	
What is the predicted direction of bias?	Towards null	
Is the overall risk of bias sufficiently high, in the context of its likely direction and the magnitude of the estimated exposure effect, to threaten conclusions about whether the exposure has an important effect on the outcome?	No	

[53] Liu, X.; Kolding, L.; Momen, N.; Gasse, C.; Pedersen, L.H. Maternal antipsychotic use during pregnancy and congenital malformations. *Am. J. Obstet. Gynecol. MFM* **2023**, *5*(6), 100950. doi: 10.1016/j.ajogmf.2023.100950.

Domain 1: Risk of bias due to confounding *variant (b): **If Y/PY to C7 and Y/PY to C8** (the analysis was based on splitting participants' follow up time according to exposure status and/or magnitude and changes in exposure status and/or magnitude likely to be related to factors that are predictive of the outcome, so both baseline and time-varying confounding need to be addressed)*

Signalling questions	Response options	Comments
1.1 Did the authors use an analysis method that was appropriate to control for time-varying as well as baseline confounding?	Y	
1.2 **If Y/PY to 1.1**: Did the authors control for all the important baseline and time-varying confounding factors for which this was necessary?	Y	
1.3 **If Y/PY/WN to 1.2**: Were confounding factors that were controlled for (and for which control was necessary) measured validly and reliably by the variables available in this study?	Y	
1.4 **If N/PN/NI to 1.1**: Did the authors control for time-varying factors or other variables measured after the start of the exposure window being studied?	N	
1.5 Did the use of negative controls, or other considerations, suggest uncontrolled confounding?	N	
Risk of bias (due to confounding) in the estimated effect of exposure on the outcome	Low risk	
What is the predicted direction of bias due to confounding?	Towards null	
Is the risk of bias (due to confounding) sufficiently high, in the context of its likely direction and the magnitude of the estimated exposure effect, to threaten conclusions about whether the exposure has an important effect on the outcome?	No	

Y = Yes; PY = Probably yes; PN = Probably no; N = No; SY = Strong yes; WY = Weak yes; SN = Strong no; WN = Weak no; NA = Not applicable; NI = No information

Domain 2: Risk of bias arising from measurement of the exposure *Variant (b): **If Y/PY to C5 and Y/PY to C6** (each individual's exposure level was estimated from measurements made at multiple time points)*

Signalling questions	Response options	Comments
**2.1 Does the measured exposure (derived from measurements at multiple time points) well-characterize the exposure metric specified to be of interest in this study? [*This was specified in the answers to D2, D3 and D4*]**	**Y**	
**2.2 Was there error in measurement, or misclassification, of the exposure, at each single time point?**	**Y**	
**2.3 If SY/WY to 2.2: Could mismeasurement or misclassification of exposure have been differential (i.e. related to the outcome or risk of the outcome)?**	**N**	
**2.4 If SY/WY to 2.2 and N/PN/WY to 2.3: Is the nature of the (non-differential) measurement error likely to bias the estimated effect of exposure on outcome?**	**N**	
**Risk of bias (arising from measurement of exposure) in the estimated effect of exposure on the outcome**	**PN**	
**What is the predicted direction of bias arising from measurement of exposure?**	**Low risk**	
**Is the risk of bias (arising from measurement of exposure) sufficiently high, in the context of its likely direction and the magnitude of the estimated exposure effect, to threaten conclusions about whether the exposure has an important effect on the outcome?**	**Towards null**	

Y = Yes; PY = Probably yes; SN = Strong no; WN = Weak no; NA = Not applicable; NI = No information

Domain 3: Risk of bias in selection of participants into the study (or into the analysis)

Signalling questions	Response options	Comments
3.1 Did follow-up begin at (or close to) the start of the exposure window for most participants? [*The exposure window is specified in D3*]	Y	
3.2 **If N/PN to 3.1**: Is the effect of exposure likely to be constant over the period of follow up analysed?	Y	
3.3 Was selection of participants into the study (or into the analysis) based on participant characteristics observed after the start of the exposure window being studied? [*The exposure window is specified in D3*]	N	
3.4 **If Y/PY to 3.3**: Were these characteristics likely to be influenced by exposure or a cause of exposure?	N	
3.5 **If Y/PY to 3.4**: Were these characteristics likely to be influenced by the outcome or a cause of the outcome?	PN	
3.6 **If N/PN to 3.2 or Y/PY to 3.5**: Is it likely that the analysis corrected for all of the potential selection biases identified in A and B above?	Y	
3.7 **If N/PN to 3.2 or Y/PY to 3.5**: Did sensitivity analyses demonstrate that the likely impact of the potential selection biases identified in A or B above was minimal?	PY	
Risk of bias (due to selection of participants into the study) in the estimated effect of exposure on the outcome	Low risk	
What is the predicted direction of bias due to selection of participants into the study?	Towards null	
Is the risk of bias (due to selection of participants into the study) sufficiently high, in the context of its likely direction and the magnitude of the estimated exposure effect, to threaten conclusions about whether the exposure has an important effect on the outcome?	No	

Y = Yes; PY = Probably yes; PN = Probably no; N = No; SN = Strong no; WN = Weak no; NA = Not applicable; NI = No information

Domain 4: Risk of bias due to post-exposure interventions

Signalling questions	Response options	Comments
4.1 Were there post-exposure interventions that were influenced by prior exposure during the follow-up period?	N	
4.2 **If Y/PY to 4.1**: Is it likely that the analysis corrected for the effect of post-exposure interventions that were influenced	NA	

by prior exposure?		
Risk of bias (due post-exposure interventions) in the estimated effect of exposure on the outcome	Low risk	
What is the predicted direction of bias due to confounding?	Towards null	
Is the risk of bias (due post-exposure interventions) sufficiently high, in the context of its likely direction and the magnitude of the estimated exposure effect, to threaten conclusions about whether the exposure has an important effect on the outcome?	No	

Y = Yes; PY = Probably yes; PN = Probably no; N = No; NA = Not applicable; NI = No information

Domain 5: Risk of bias due to missing data

Signalling questions	Response options	Comments
5.1 Were complete data on exposure status available for all, or nearly all, participants?	Y	
5.2 Were complete data on the outcome available for all, or nearly all, participants?	Y	
5.3 Were complete data on confounding variables available for all, or nearly all, participants?	Y	
5.4 **If N/PN/NI to 5.1, 5.2 or 5.3**: Is the result based on a complete case analysis?	Y	
5.5 **If Y/PY/NI**: Was exclusion from the analysis because of missing data (in exposure, confounders or the outcome) likely to be related to the true value of the outcome?	N	
5.6 **If N/PN to 5.5**: Were all or most predictors of missingness (in exposure, confounders or the outcome) included in the analysis model?	SY	
5.7 **If N/PN to 5.4**: Was the analysis based on imputing missing values?	Y	
5.8 **If Y/PY to 5.7**: Was imputation performed appropriately?	Y	
5.9 **If N/PN to 5.7**: Was an appropriate alternative method used to correct for bias due to missing data?	Y	
5.10 **If PN/N/NI to 5.1, 5.2 or 5.3**: Is there evidence that the result was not biased by missing data?	Y	
Risk of bias (due to missing data) in the estimated effect of exposure on the outcome	Low risk	
What is the predicted direction of bias due to missing data?	Towards null	
Is the risk of bias (due to missing data) sufficiently high, in the context of its likely direction and the magnitude of the estimated exposure effect, to threaten conclusions about whether the exposure has an important effect on the outcome?	No	

Y = Yes; PY = Probably yes; PN = Probably no; N = No; SY = Strong yes; WY = Weak yes; NA = Not applicable; NI = No information

Domain 6: Risk of bias arising from measurement of the outcome

Signalling questions	Response options	Comments
6.1 Could measurement or ascertainment of the outcome have differed between exposure groups or levels of exposure?	N	
6.2 Were outcome assessors aware of study participants' exposure history?	N	
6.3 **If Y/PY/NI to 6.2**: Could assessment of the outcome have been influenced by knowledge of participants' exposure history?	N	
Risk of bias (arising from measurement of outcomes) in the estimated effect of exposure on the outcome	Low risk	
What is the predicted direction of bias arising from measurement of outcomes?	Towards null	
Is the risk of bias (arising from measurement of outcomes) sufficiently high, in the context of its likely direction and the magnitude of the estimated exposure effect, to threaten conclusions about whether the exposure has an important effect on the outcome?	No	

Y = Yes; PY = Probably yes; PN = Probably no; N = No; SY = Strong yes; WY = Weak yes; NA = Not applicable; NI = No information

Domain 7: Risk of bias in selection of the reported result

Signalling questions	Response options	Comments
7.1 Was the result reported in accordance with an available, pre-determined analysis plan?	Y	
7.2 **If N/PN/NI to 7.1**: Is the reported effect estimate likely to be selected, based on desirability of the magnitude (or statistical significance) of the estimated effect of exposure on outcome, from multiple *exposure measurements* within the exposure domain?	N	
7.3 Is the reported effect estimate likely to be selected, based on desirability of the magnitude (or statistical significance) of the estimated effect of exposure on outcome, from multiple *outcome measurements* within the outcome domain?	N	
7.4 Is the reported effect estimate likely to be selected, based on desirability of the magnitude (or statistical significance) of the estimated effect of exposure on outcome, from multiple *analyses* of the exposure-outcome relationship?	N	
7.5 Is the reported effect estimate likely to be selected, based on the basis of desirability of the results (e.g. statistical significance), from different *subgroups*?	N	
Risk of bias (due to selection of the reported result) in the estimated effect of exposure on the outcome	Low risk	
What is the predicted direction of bias due to selection of the reported result?	Towards null	
Is the risk of bias (due to selection of the reported result) sufficiently high, in the context of its likely direction and the magnitude of the estimated exposure effect, to threaten conclusions about whether the exposure has an important effect on the outcome?	No	

Y = Yes; PY = Probably yes; PN = Probably no; N = No; NA = Not applicable; NI = No information

Overall risk of bias

	Response options	Comments
Overall risk of bias	Low risk of bias	

What is the predicted direction of bias?	Towards null	
Is the overall risk of bias sufficiently high, in the context of its likely direction and the magnitude of the estimated exposure effect, to threaten conclusions about whether the exposure has an important effect on the outcome?	No	

[54] Schrijver, L.; Robakis, T.K.; Kamperman, A.M.; Bijma, H.; Honig, A.; van Kamp, I.L.; Hoogendijk, W.J.G.; Bergink, V.; Poels, E.M.P. Neurodevelopment in school-aged children after intrauterine exposure to antipsychotics. *Acta Psychiatr. Scand.* **2023**, *147*(1), 43-53. doi: 10.1111/acps.13517. Epub 2022 Nov 10.

Domain 1: Risk of bias due to confounding *variant (b): **If Y/PY to C7 and Y/PY to C8** (the analysis was based on splitting participants' follow up time according to exposure status and/or magnitude and changes in exposure status and/or magnitude likely to be related to factors that are predictive of the outcome, so both baseline and time-varying confounding need to be addressed)*

Signalling questions	Response options	Comments
1.1 Did the authors use an analysis method that was appropriate to control for time-varying as well as baseline confounding?	Y	
1.2 **If Y/PY to 1.1**: Did the authors control for all the important baseline and time-varying confounding factors for which this was necessary?	Y	
1.3 **If Y/PY/WN to 1.2**: Were confounding factors that were controlled for (and for which control was necessary) measured validly and reliably by the variables available in this study?	Y	
1.4 **If N/PN/NI to 1.1**: Did the authors control for time-varying factors or other variables measured after the start of the exposure window being studied?	N	
1.5 Did the use of negative controls, or other considerations, suggest uncontrolled confounding?	N	
Risk of bias (due to confounding) in the estimated effect of exposure on the outcome	Low risk	
What is the predicted direction of bias due to confounding?	Towards null	
Is the risk of bias (due to confounding) sufficiently high, in the context of its likely direction and the magnitude of the estimated exposure effect, to threaten conclusions about whether the exposure has an important effect on the outcome?	No	

Y = Yes; PY = Probably yes; PN = Probably no; N = No; SY = Strong yes; WY = Weak yes; SN = Strong no; WN = Weak no; NA = Not applicable; NI = No information

Domain 2: Risk of bias arising from measurement of the exposure *Variant (b): **If Y/PY to C5 and Y/PY to C6** (each individual's exposure level was estimated from measurements made at multiple time points)*

Signalling questions	Response options	Comments
**2.1 Does the measured exposure (derived from measurements at multiple time points) well-characterize the exposure metric specified to be of interest in this study? [*This was specified in the answers to D2, D3 and D4*]**	**Y**	
**2.2 Was there error in measurement, or misclassification, of the exposure, at each single time point?**	**N**	
**2.3 If SY/WY to 2.2: Could mismeasurement or misclassification of exposure have been differential (i.e. related to the outcome or risk of the outcome)?**	**N**	
**2.4 If SY/WY to 2.2 and N/PN/WY to 2.3: Is the nature of the (non-differential) measurement error likely to bias the estimated effect of exposure on outcome?**	**PN**	
**Risk of bias (arising from measurement of exposure) in the estimated effect of exposure on the outcome**	**Low risk**	
**What is the predicted direction of bias arising from measurement of exposure?**	**Towards null**	
**Is the risk of bias (arising from measurement of exposure) sufficiently high, in the context of its likely direction and the magnitude of the estimated exposure effect, to threaten conclusions about whether the exposure has an important effect on the outcome?**	**No**	

Y = Yes; PY = Probably yes; SN = Strong no; WN = Weak no; NA = Not applicable; NI = No information

Domain 3: Risk of bias in selection of participants into the study (or into the analysis)

Signalling questions	Response options	Comments
3.1 Did follow-up begin at (or close to) the start of the exposure window for most participants? [*The exposure window is specified in D3*]	Y	
3.2 **If N/PN to 3.1**: Is the effect of exposure likely to be constant over the period of follow up analysed?	Y	
3.3 Was selection of participants into the study (or into the analysis) based on participant characteristics observed after the start of the exposure window being studied? [*The exposure window is specified in D3*]	N	
3.4 **If Y/PY to 3.3**: Were these characteristics likely to be influenced by exposure or a cause of exposure?	N	
3.5 **If Y/PY to 3.4**: Were these characteristics likely to be influenced by the outcome or a cause of the outcome?	PN	
3.6 **If N/PN to 3.2 or Y/PY to 3.5**: Is it likely that the analysis corrected for all of the potential selection biases identified in A and B above?	Y	
3.7 **If N/PN to 3.2 or Y/PY to 3.5**: Did sensitivity analyses demonstrate that the likely impact of the potential selection biases identified in A or B above was minimal?	PY	
Risk of bias (due to selection of participants into the study) in the estimated effect of exposure on the outcome	Low risk	
What is the predicted direction of bias due to selection of participants into the study?	Towards null	
Is the risk of bias (due to selection of participants into the study) sufficiently high, in the context of its likely direction and the magnitude of the estimated exposure effect, to threaten conclusions about whether the exposure has an important effect on the outcome?	No	

Y = Yes; PY = Probably yes; PN = Probably no; N = No; SN = Strong no; WN = Weak no; NA = Not applicable; NI = No information

Domain 4: Risk of bias due to post-exposure interventions

**Signalling questions**	**Response options**	**Comments**
4.1 Were there post-exposure interventions that were influenced by prior exposure during the follow-up period?	N	
4.2 **If Y/PY to 4.1**: Is it likely that the analysis corrected for the effect of post-exposure interventions that were influenced by prior exposure?	NA	
Risk of bias (due post-exposure interventions) in the estimated effect of exposure on the outcome	Low risk	
What is the predicted direction of bias due to confounding?	Towards null	
Is the risk of bias (due post-exposure interventions) sufficiently high, in the context of its likely direction and the magnitude of the estimated exposure effect, to threaten conclusions about whether the exposure has an important effect on the outcome?	No	

Y = Yes; PY = Probably yes; PN = Probably no; N = No; NA = Not applicable; NI = No information

Domain 5: Risk of bias due to missing data

**Signalling questions**	**Response options**	**Comments**
5.1 Were complete data on exposure status available for all, or nearly all, participants?	Y	
5.2 Were complete data on the outcome available for all, or nearly all, participants?	Y	
5.3 Were complete data on confounding variables available for all, or nearly all, participants?	Y	
5.4 **If N/PN/NI to 5.1, 5.2 or 5.3**: Is the result based on a complete case analysis?	Y	
5.5 **If Y/PY/NI**: Was exclusion from the analysis because of missing data (in exposure, confounders or the outcome) likely to be related to the true value of the outcome?	N	
5.6 **If N/PN to 5.5**: Were all or most predictors of missingness (in exposure, confounders or the outcome) included in the analysis model?	SY	
5.7 **If N/PN to 5.4**: Was the analysis based on imputing missing values?	Y	
5.8 **If Y/PY to 5.7**: Was imputation performed appropriately?	Y	
5.9 **If N/PN to 5.7**: Was an appropriate alternative method used to correct for bias due to missing data?	Y	
5.10 **If PN/N/NI to 5.1, 5.2 or 5.3**: Is there evidence that the result was not biased by missing data?	Y	
Risk of bias (due to missing data) in the estimated effect of exposure on the outcome	Low risk	
What is the predicted direction of bias due to missing data?	Towards null	
Is the risk of bias (due to missing data) sufficiently high, in the context of its likely direction and the magnitude of the estimated exposure effect, to threaten conclusions about whether the exposure has an important effect on the outcome?	No	

Y = Yes; PY = Probably yes; PN = Probably no; N = No; SY = Strong yes; WY = Weak yes; NA = Not applicable; NI = No information

Domain 6: Risk of bias arising from measurement of the outcome

**Signalling questions**	**Response options**	**Comments**
6.1 Could measurement or ascertainment of the outcome have differed between exposure groups or levels of exposure?	N	
6.2 Were outcome assessors aware of study participants' exposure history?	N	
6.3 **If Y/PY/NI to 6.2**: Could assessment of the outcome have been influenced by knowledge of participants' exposure history?	N	
Risk of bias (arising from measurement of outcomes) in the estimated effect of exposure on the outcome	Low risk	
What is the predicted direction of bias arising from measurement of outcomes?	Towards null	
Is the risk of bias (arising from measurement of outcomes) sufficiently high, in the context of its likely direction and the magnitude of the estimated exposure effect, to threaten conclusions about whether the exposure has an important effect on the outcome?	No	

Y = Yes; PY = Probably yes; PN = Probably no; N = No; SY = Strong yes; WY = Weak yes; NA = Not applicable; NI = No information

Domain 7: Risk of bias in selection of the reported result

**Signalling questions**	**Response options**	**Comments**
7.1 Was the result reported in accordance with an available, pre-determined analysis plan?	Y	
7.2 **If N/PN/NI to 7.1**: Is the reported effect estimate likely to be selected, based on desirability of the magnitude (or statistical significance) of the estimated effect of exposure on outcome, from multiple *exposure measurements* within the exposure domain?	N	
7.3 Is the reported effect estimate likely to be selected, based on desirability of the magnitude (or statistical significance) of the estimated effect of exposure on outcome, from multiple *outcome measurements* within the outcome domain?	N	
7.4 Is the reported effect estimate likely to be selected, based on desirability of the magnitude (or statistical significance) of the estimated effect of exposure on outcome, from multiple *analyses* of the exposure-outcome relationship?	N	
7.5 Is the reported effect estimate likely to be selected, based on the basis of desirability of the results (e.g. statistical significance), from different *subgroups*?	N	
Risk of bias (due to selection of the reported result) in the estimated effect of exposure on the outcome	Low risk	
What is the predicted direction of bias due to selection of the reported result?	Towards null	
Is the risk of bias (due to selection of the reported result) sufficiently high, in the context of its likely direction and the magnitude of the estimated exposure effect, to threaten conclusions about whether the exposure has an important effect on the outcome?	No	

Y = Yes; PY = Probably yes; PN = Probably no; N = No; NA = Not applicable; NI = No information

Overall risk of bias

	Response options	Comments
Overall risk of bias	Low risk of bias	
What is the predicted direction of bias?	Towards null	
Is the overall risk of bias sufficiently high, in the context of its likely direction and the magnitude of the estimated exposure effect, to threaten conclusions about whether the exposure has an important effect on the outcome?	No	

Table S3. The PRISMA 2020 Checklist

PRISMA 2020 Checklist

Section and Topic	Item #	Checklist item	Location where item is reported
**TITLE**			1
Title	1	Identify the report as a systematic review.	1
**ABSTRACT**			2
Abstract	2	See the PRISMA 2020 for Abstracts checklist.	2
**INTRODUCTION**			1-3
Rationale	3	Describe the rationale for the review in the context of existing knowledge.	2-3
Objectives	4	Provide an explicit statement of the objective(s) or question(s) the review addresses.	2-3
**METHODS**			3-4
Eligibility criteria	5	Specify the inclusion and exclusion criteria for the review and how studies were grouped for the syntheses.	3
Information sources	6	Specify all databases, registers, websites, organisations, reference lists and other sources searched or consulted to identify studies. Specify the date when each source was last searched or consulted.	3
Search strategy	7	Present the full search strategies for all databases, registers and websites, including any filters and limits used.	3-5, Fig. 1
Selection process	8	Specify the methods used to decide whether a study met the inclusion criteria of the review, including how many reviewers screened each record and each report retrieved, whether they worked independently, and if applicable, details of automation tools used in the process.	3
Data collection process	9	Specify the methods used to collect data from reports, including how many reviewers collected data from each report, whether they worked independently, any processes for obtaining or confirming data from study investigators, and if applicable, details of automation tools used in the process.	3-4
Data items	10a	List and define all outcomes for which data were sought. Specify whether all results that were compatible with each outcome domain in each study were sought (e.g. for all measures, time points, analyses), and if not, the methods used to decide which results to collect.	3
	10b	List and define all other variables for which data were sought (e.g. participant and intervention characteristics, funding sources). Describe any assumptions made about any missing or unclear information.	3
Study risk of bias assessment	11	Specify the methods used to assess risk of bias in the included studies, including details of the tool(s) used, how many reviewers assessed each study and whether they worked independently, and if applicable, details of automation tools used in the process.	4
Effect measures	12	Specify for each outcome the effect measure(s) (e.g. risk ratio, mean difference) used in the synthesis or	4-5

Section and Topic	Item #	Checklist item	Location where item is reported
		presentation of results.	
Synthesis methods	13a	Describe the processes used to decide which studies were eligible for each synthesis (e.g. tabulating the study intervention characteristics and comparing against the planned groups for each synthesis (item #5)).	3-5
	13b	Describe any methods required to prepare the data for presentation or synthesis, such as handling of missing summary statistics, or data conversions.	3-5
	13c	Describe any methods used to tabulate or visually display results of individual studies and syntheses.	Table 7-16
	13d	Describe any methods used to synthesize results and provide a rationale for the choice(s). If meta-analysis was performed, describe the model(s), method(s) to identify the presence and extent of statistical heterogeneity, and software package(s) used.	Table 7-27
	13e	Describe any methods used to explore possible causes of heterogeneity among study results (e.g. subgroup analysis, meta-regression).	Subgroup analysis
	13f	Describe any sensitivity analyses conducted to assess robustness of the synthesized results.	NA
Reporting bias assessment	14	Describe any methods used to assess risk of bias due to missing results in a synthesis (arising from reporting biases).	3 Supplement
Certainty assessment	15	Describe any methods used to assess certainty (or confidence) in the body of evidence for an outcome.	NA
**RESULTS**			7-16
Study selection	16a	Describe the results of the search and selection process, from the number of records identified in the search to the number of studies included in the review, ideally using a flow diagram.	Figure 1, 7
	16b	Cite studies that might appear to meet the inclusion criteria, but which were excluded, and explain why they were excluded.	Supplement
Study characteristics	17	Cite each included study and present its characteristics.	4-18, Table 1
Risk of bias in studies	18	Present assessments of risk of bias for each included study.	Supplement
Results of individual studies	19	For all outcomes, present, for each study: (a) summary statistics for each group (where appropriate) and (b) an effect estimate and its precision (e.g. confidence/credible interval), ideally using structured tables or plots.	6-18
Results of syntheses	20a	For each synthesis, briefly summarise the characteristics and risk of bias among contributing studies.	NA
	20b	Present results of all statistical syntheses conducted. If meta-analysis was done, present for each the summary estimate and its precision (e.g. confidence/credible interval) and measures of statistical heterogeneity. If comparing groups, describe the direction of the effect.	NA
	20c	Present results of all investigations of possible causes of heterogeneity among study results.	NA
	20d	Present results of all sensitivity analyses conducted to assess the robustness of the synthesized results.	NA
Reporting biases	21	Present assessments of risk of bias due to missing results (arising from reporting biases) for each synthesis assessed.	Supplement
Certainty of evidence	22	Present assessments of certainty (or confidence) in the body of evidence for each outcome assessed.	NA
**DISCUSSION**			16-18
Discussion	23a	Provide a general interpretation of the results in the context of other evidence.	16-18
	23b	Discuss any limitations of the evidence included in the review.	17-18
	23c	Discuss any limitations of the review processes used.	18
	23d	Discuss implications of the results for practice, policy, and future research.	18
**OTHER INFORMATION**			
Registration and protocol	24a	Provide registration information for the review, including register name and registration number, or state that the review was not registered.	4
	24b	Indicate where the review protocol can be accessed, or state that a protocol was not prepared.	4
	24c	Describe and explain any amendments to information provided at registration or in the protocol.	NA
Support	25	Describe sources of financial or non-financial support for the review, and the role of the funders or sponsors in the review.	18
Competing interests	26	Declare any competing interests of review authors.	18
Availability of data, code and other materials	27	Report which of the following are publicly available and where they can be found: template data collection forms; data extracted from included studies; data used for all analyses; analytic code; any other materials used in the review.	NA

From: [14] Page, M.J.; McKenzie, J.E.; Bossuyt, P.M.; Boutron, I.; Hoffmann, T.C.; Mulrow, C.D.; et al. The PRISMA 2020 statement: an updated guideline for reporting systematic reviews. *B.M.J.* **2021**, *372*, n71. doi: 10.1136/bmj.n71.

For more information, visit: http://www.prisma-statement.org/
